# Skeletal Rearrangement of Biaryls via Rhodium-Azirine Intermediate: A Route to Solid-State Emissive Polycyclic Sulfamates

**DOI:** 10.1021/acscatal.5c06179

**Published:** 2025-12-19

**Authors:** Nil Insa-Carreras, Àlex Díaz-Jiménez, Andrea Pinto, Laura Rodríguez, Albert Poater, Anna Roglans, Anna Pla-Quintana

**Affiliations:** † Institut de Química Computacional i Catàlisi (IQCC) and Departament de Química, 16738 Universitat de Girona (UdG), C/Maria Aurèlia Capmany, 69, E-17003 Girona, Spain; ‡ Departament de Química Inorgànica i Orgànica, Secció de Química Inorgànica, 16724 Universitat de Barcelona, Martí i Franquès 1-11, E-08028 Barcelona, Spain; § Institut de Nanociència i Nanotecnologia (IN2UB), Universitat de Barcelona, 08028 Barcelona, Spain

**Keywords:** Nitrene, Rhodium, Sulfamates, Solid-state fluorescence, Skeletal rearrangement

## Abstract

We report a rhodium-catalyzed cascade transformation of biaryl alkynylsulfamates that enables access to rigid, spirocyclic cycloheptatrienes with strong solid-state fluorescence. Building on prior work confirming selective 7-endo nitrene–alkyne cyclization, our design leverages skeletal rigidity to promote a thermally driven electrocyclic ring opening. Detailed mechanistic studies, supported by density functional theory (DFT) calculations, reveal a key rhodium-bound azirine intermediate that undergoes a dearomative single-carbon insertion into the biaryl framework. This reaction proceeds via a distinct, noncarbene-based pathway, expanding the scope of nitrene-mediated rearrangements beyond classical Büchner-type mechanisms. The method provides a modular route to structurally complex and photoactive scaffolds through rationally designed cascade processes.

## Graphical abstract

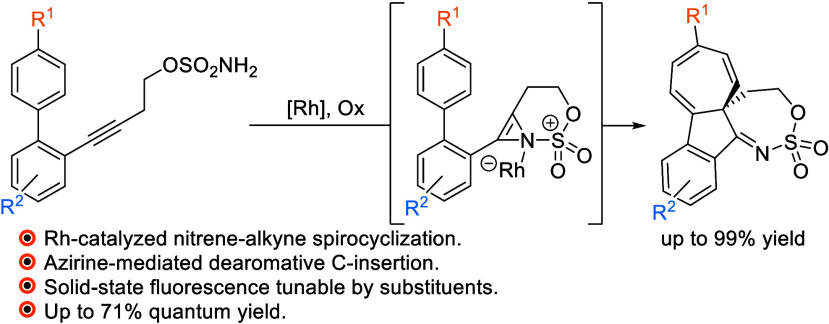

## Introduction

The development of innovative methodologies for the selective synthesis of complex molecules remains a central challenge in organic chemistry. Skeletal rearrangement reactions^1^ hold great potential due to their ability to construct complex molecular architectures in a step-economical manner. These reactions involve a fundamental reorganization of molecular frameworks through the cleavage and reformation of bonds, and they are especially powerful when catalyzed by transition-metal complexes. One of the most common driving forces in transition-metal-catalyzed skeletal rearrangement is aromaticity gain.^2^ However, processes that proceed through dearomatization also represent a plausible pathway.

Despite the challenges of breaking aromaticity in 6-membered rings, carbenes have long enabled access to nonaromatic 7-membered systems.^3^ The earliest example dates back to 1885, when Büchner et al. reported the ring expansion of benzene using thermally generated carbenes.^4^ Since then, a wide range of methods employing diverse carbene precursors and catalytic systems,^5^ including more recent enantioselective variants,^6^ have been developed.

The Büchner reaction (Scheme 1A) holds strong potential for skeletal rearrangement, especially when the reactive electrophilic intermediate arises from complex bond-forming processes. Transition-metal-catalyzed alkyne cyclizations have proven effective for generating carbenes or carbocations in situ, enabling Büchner-type cycloadditions. These tandem strategies allow rapid construction of complex frameworks, even in intermolecular settings.^7^ Key precedents highlighting the utility of this approach are shown in Schemes 1B–E. In 2014, Panek et al.^8^ developed a rhodium-catalyzed cascade involving a 7-endo nitrene-alkyne cyclization to generate an α-iminocarbene, which underwent arene cyclopropanation to yield tetracyclic norcaradienes (Scheme 1B). Interestingly, the ring expansion did not occur but required nucleophilic opening of the sulfamate moiety, followed by Lewis or Brønsted acid-promoted electrocyclization. In 2019, Xu et al.^9^ reported a copper-catalyzed cascade of alkyne-diazo compounds to form dihydrocyclohepta­[*b*]­indoles via a 5-exo carbene/alkyne metathesis (CAM) and Büchner-type ring expansion (Scheme 1C). In 2022, Zhu et al.^10^ disclosed a rhodium-catalyzed enantioselective Büchner reaction, with the carbene intermediate formed in situ via a 5-exo cyclization of aryloxyenynones (Scheme 1D). More recently, Ye et al.^11^ described a copper-catalyzed cyclization of *N*-propargyl ynamides via vinyl cation intermediates, delivering chiral tricyclic cycloheptatrienes in high yields and enantioselectivities (Scheme 1E).

**Scheme 1:**
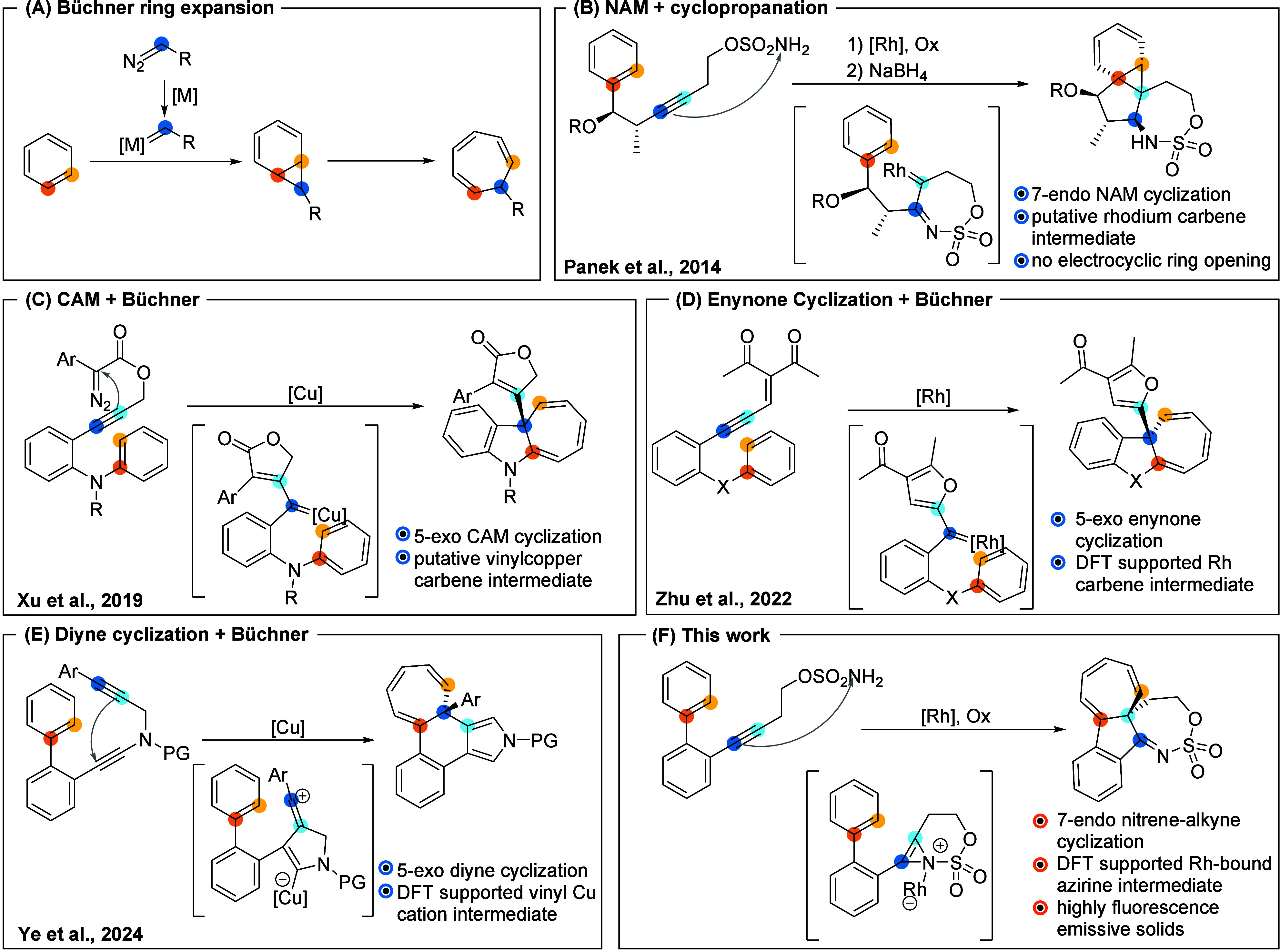
Dearomative Skeletal Rearrangements: (A) Traditional Büchner Ring Expansion, (B) Nitrene–Alkyne Metathesis and Cyclopropanation, (C) Carbene–Alkyne Metathesis and Büchner Ring Expansion, (D) Enynone Cyclization and Büchner Ring Expansion, (E) Diyne Cyclization and Büchner Ring Expansion, and (F) Nitrene–Alkyne Cyclization and Dearomative Single Carbon Atom Insertion

Noting that endocyclized intermediates yield intriguing spirocyclic structures, we aimed to develop a skeletal rearrangement reaction that, unlike Panek’s example (Scheme 1B), proceeds with electrocyclic ring opening. Landmark studies of Blakey et al.^12^ and subsequent related work^13^ demonstrated that alkynylsulfamic esters exhibit excellent selectivity for 7-endo nitrene-alkyne cyclization. Building on these findings and drawing from our experience in transition metal-catalyzed cascade reactions,^14^ we sought to modulate skeletal rigidity to access rigid spirocyclic cycloheptatrienes.

We report here the skeletal rearrangement of biaryl scaffolds via a rhodium-nitrene-alkyne initiated cascade process, affording highly solid-state fluorescent tetracyclic sulfamates (Scheme 1F). An in-depth mechanistic study, supported by DFT calculations, reveals the formation of a key rhodium-bound azirine intermediate that triggers an unprecedented dearomative single-carbon insertion of the biaryl framework.

## Results and Discussion

We started by developing a synthesis of model compound **1a** (R^1^=R^2^=H), which features a rigid biphenyl moiety directly linked to a homopropargylic sulfamic ester. It is well-established that sulfamates, in the presence of oxidants and transition-metal complexes, generate metal–nitrene intermediates. After some optimization (see the for full details), we found that the linear substrate was quantitatively converted to tetracyclic sulfamate **2a** using 1.2 equiv of PhI­(OAc)_2_, under [Rh_2_(*R*-BTPCP)_4_] catalysis in dichloromethane (DCM) at room temperature (Scheme 2). A key parameter in the optimization was the choice of oxidant—PhI­(OAc)_2_ proved optimal—although, interestingly, the reaction showed very low sensitivity to its amount, proceeding equally efficiently with 1.2 and 4.8 equiv. During the optimization process, we also tested silver catalysts, which proved ineffective, and evaluated the enantioselectivity using chiral Rh paddlewheel catalysts.^15^ An enantiomeric excess (ee) of 58% was achieved with Rh_2_(*R*-*p*-Ph-TPCP)_4_, albeit with a yield of only 77%. Conversely, Rh_2_(*R*-BTPCP)_4_ provided a moderate 40% ee but an excellent 99% yield and was therefore chosen as the optimized catalyst. Notably, the reaction can be carried out without strictly anhydrous conditions. The transformation involves the construction of an all-carbon quaternary center, which is spirocyclic to two seven-membered rings, one formed by cyclization of the sulfamic ester to the alkyne and the other resulting from a single-carbon insertion into the upper aryl ring as can be unambiguously determined upon X-ray diffraction. Of note, aromatic substitution has never been observed to compete. Interestingly, the compound exhibited fluorescence in both the solid and solution states.

**Scheme 2:**
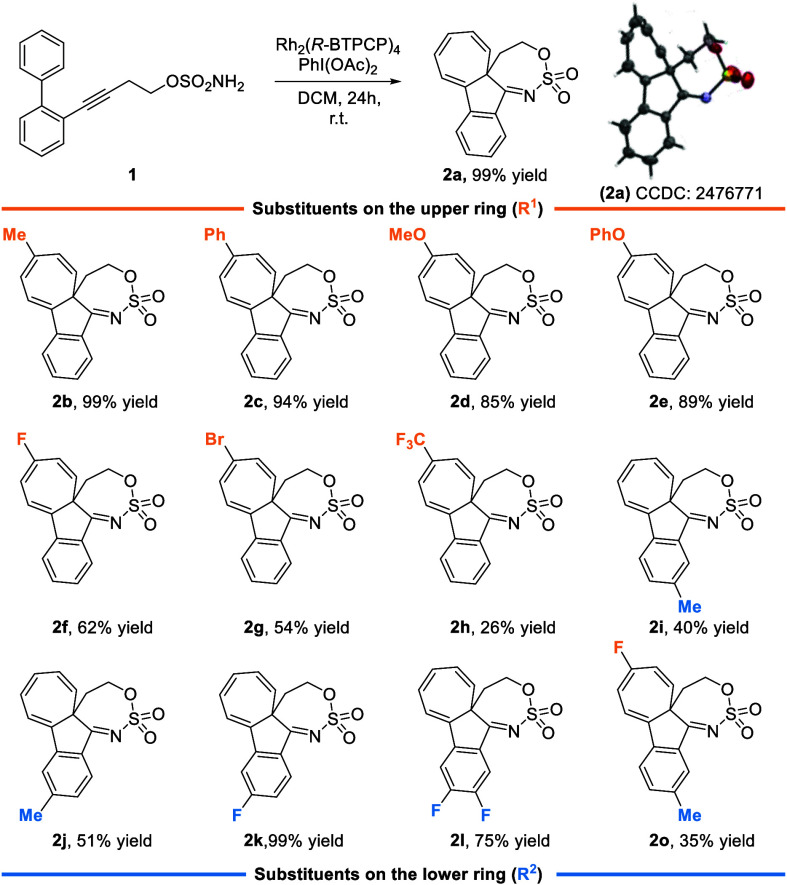
Optimized Reaction Conditions and Substrate Scope^a^

a Reaction conditions: sulfamate **1** (0.05 mmol), iodine oxidant (0.06 mmol), and Rh_2_(*R*-BTPCP)_4_ (2.5 mol %) in DCM (1 mL). The reactions were stirred for 24 h. Isolated yields.

Given the relevance of the transformation and the value of the resulting scaffold, we proceeded to evaluate the scope of the reaction with substrates bearing substituents on either the upper (R^1^) or lower (R^2^) aryl ring of the biphenyl moiety (Scheme 2). Introduction of mildly electron-donating groups, such as methyl or phenyl, to the upper ring provided cyclized products **2b** and **2c** in excellent yields of 99% and 94%, respectively. Increasing the electron-donating character resulted in only a slight decrease in yield, with MeO (**2d**) affording an 85% yield and PhO (**2e**) an 89% yield. However, the presence of a dimethylamino group completely inhibited the reaction. In contrast, the introduction of electron-withdrawing groups led to a clear decline in cyclization efficiency as expected in a process involving a Büchner rearrangement, when the nucleophilicity of the aryl ring is decreased. Specifically, halogen substituents such as fluorine (**2f**) or bromine (**2g**), resulted in reduced yields of 62% and 54%, respectively, while the strongly withdrawing CF_3_ group afforded the cyclized product **2h** in only 26% yield.

Interestingly, the opposite trend was observed for substitution on the lower ring. Introduction of an electron-donating methyl group at the *para*- (**2i**) or *meta*- (**2j**) position led to moderate yields of 40% and 51%, respectively. In contrast, electron-withdrawing substituents such as *m*-fluoro (**2k**) or *p*,*m*-difluoro resulted in significantly improved yields, reaching 99% and 75%, respectively. To further explore substitution effects, we also synthesized the analogue **2o** bearing one substituent on each phenyl ring. The reaction afforded the target compound in modest yield, which can be attributed to the electron-poor nature of the upper ring involved in the Büchner rearrangement.

Compounds that exhibit solid-state fluorescence are of great importance due to their ability to emit light efficiently in the condensed phase, a property that is often suppressed by aggregation in many fluorescent materials. This makes them highly valuable in a variety of practical applications.^16^

In this context, we decided to characterize the photophysical properties of our compounds. We recorded their absorption and emission spectra in 10^–5^ M dichloromethane solutions (see Figure 1 and the ). All compounds exhibit absorption bands near 275, 330, and 400 nm, corresponding to π–π*/*n*–π* transitions involving the sulfamate unit. The low-energy band red-shifts with increasing electron-donating character of the substituents, consistent with reduced HOMO–LUMO gaps (see the ).

**Figure 1:**
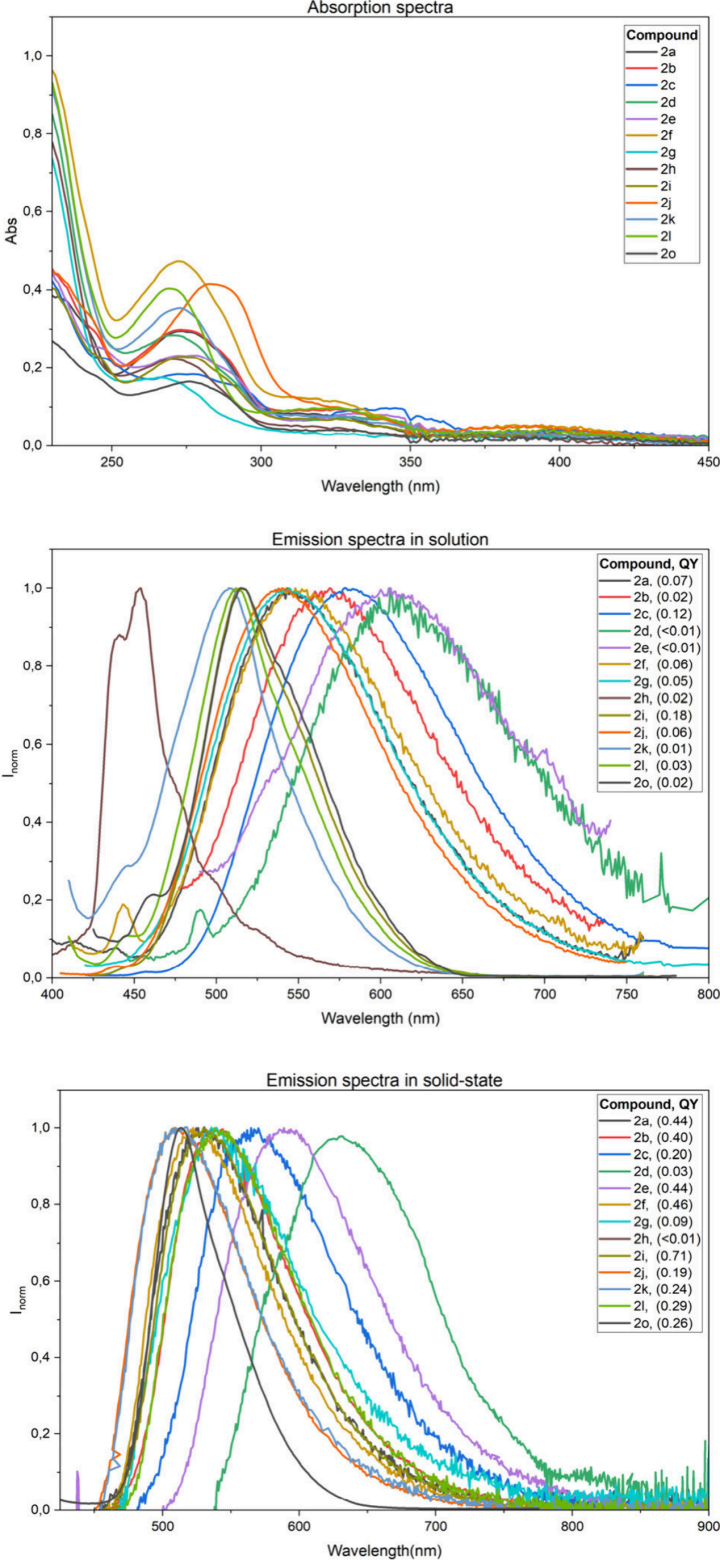
UV-Vis absorption and fluorescence emission spectra of compounds **2a**–**2o** in solution and in the solid state.

Emission spectra show bands between 450 nm and 610 nm in solution, and 515–630 nm in the solid state. Both emission quantum yields and lifetimes increase in the solid state, suggesting that molecular packing reduces nonradiative decay (see the ). An exception is CF_3_-substituted **2h**, which emits at 454 nm in solution, consistent with its large HOMO–LUMO gap, but is nearly nonemissive in the solid state. Since the calculated frontier molecular orbitals do not provide a clear rationale for the absence of solid-state emission in compound **2h**, this behavior may instead arise from specific molecular packing effects in the solid state.

Substituent electronics and position critically influence emission. Electron-donating groups such as OMe (**2d**) and OPh (**2e**) on the cycloheptatriene ring lead to red-shifted emission, whereas methylation at the electron-rich 6-membered ring (**2i** and **2j**) enhances emission more than methylation at the seven-membered ring (**2b**). Notably, compound **2i**, bearing a methyl group at the 4-position of the aryl ring exhibits an impressive quantum yield of 71%. Fluorination of the 7-membered ring (**2f**) also improves emission efficiency, compared to fluorination on the 6-membered ring (**2k** and **2l**).

We finally examined the disubstituted analogue (**2o**) bearing a fluoro substituent on the seven-membered ring (R^1^) and a *para*-methyl substituent on the phenyl ring (R^2^), as these groups individually afforded the highest quantum yields (46% for **2f** and 71% for **2i**). Interestingly, the quantum yield of **2o** (26%) was markedly lower than those of either monosubstituted analogue, suggesting that the combined electronic effects of the substituents may perturb the excited-state electronic structure, thereby diminishing the emissive efficiency.

HOMO–LUMO analysis suggests that efficient IL transitions correlate with electron-rich aryl rings adjacent to the LUMO-localized sulfamate moiety. Supporting this, chemical reduction of the imine in **2a** with NaBH_4_ diastereoselectively provides product **3a** (Scheme 3a),^17^ with entirely abolished fluorescence.

**Scheme 3:**
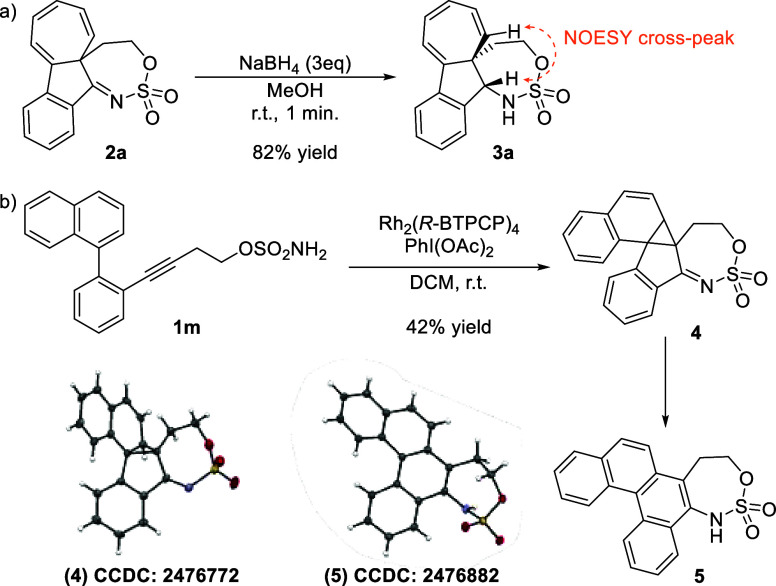
Mechanistic Experiments: (a) Reduction of the Sulfonylimine Moiety in **2a**, and (b) Reaction of the Naphthoyl Substrate **1m**, Supporting the Involvement of a Norcaradiene Intermediate

Given the novelty of this transformation, we investigated the underlying reaction mechanism. The reaction is initiated by the formation of a rhodium–nitrene complex, putatively via substrate–oxidant interaction that generates small amounts of iminoiodinane. This intermediate rapidly reacts with the catalyst to form the active Rh-nitrene species.^18^ The subsequent steps were explored by DFT (M06-D3/6–311+G**∼SDD) using Rh_2_(formate)_4_ as a simplified catalyst model.^19^ The rhodium-nitrene species can exist in three electronic states: closed-shell singlet, open-shell singlet, and open-shell triplet. Among these, the triplet state **A-t** is the ground state, with both singlet states located at least 13.3 kcal/mol higher in energy, consistent with previous DFT studies on Rh paddlewheel-catalyzed nitrene reactions,^20^ and was taken as the reference point for energy calculations (Figure 2). Nitrenes exhibit notable reactivity toward alkynes in a reaction proposed to proceed either via a singlet concerted pathway or a triplet stepwise mechanism.^21^ Both pathways were examined, and only the triplet transition state (**TSAB-t**) could be located. The corresponding closed-shell and open-shell singlet structures could not be optimized, but single-point calculations estimate them to lie 9.0 kcal/mol (closed-shell) and 2.7 kcal/mol (open-shell) above **TSAB-t**. Although this confirms that the triplet stepwise process is preferred, the subsequent intermediate, featuring a newly formed N–C bond and lying 25.4 kcal/mol below the initial **A-t** structure, appears to be fictitious, as it does not evolve to the azirine intermediate **B** (see the for details). In contrast, both the closed- and open-shell singlet analogues of **A-t** could not be located, because they directly evolve toward **B**. These results suggest that spin crossing likely occurs at or immediately after **TSAB-t**, a process that is highly favorable since the rhodium-bound azirine intermediate **B** is stabilized by 47.0 kcal/mol, relative to **A-t**. From intermediate **B**, all attempts to model rhodium migration to the azirine, followed by ring opening to form an α-iminorhodium carbene via either 7-endo or 6-exo cyclization, were unsuccessful. Conversely, we found that a nucleophilic attack by the aryl ring on the azirine, proceeding through a noncarbene pathway, occurs with an activation barrier of 12.4 kcal/mol via transition state **TSBC**, resulting in the formation of norcaradiene intermediate **C** upon rhodium dissociation.

**Figure 2:**
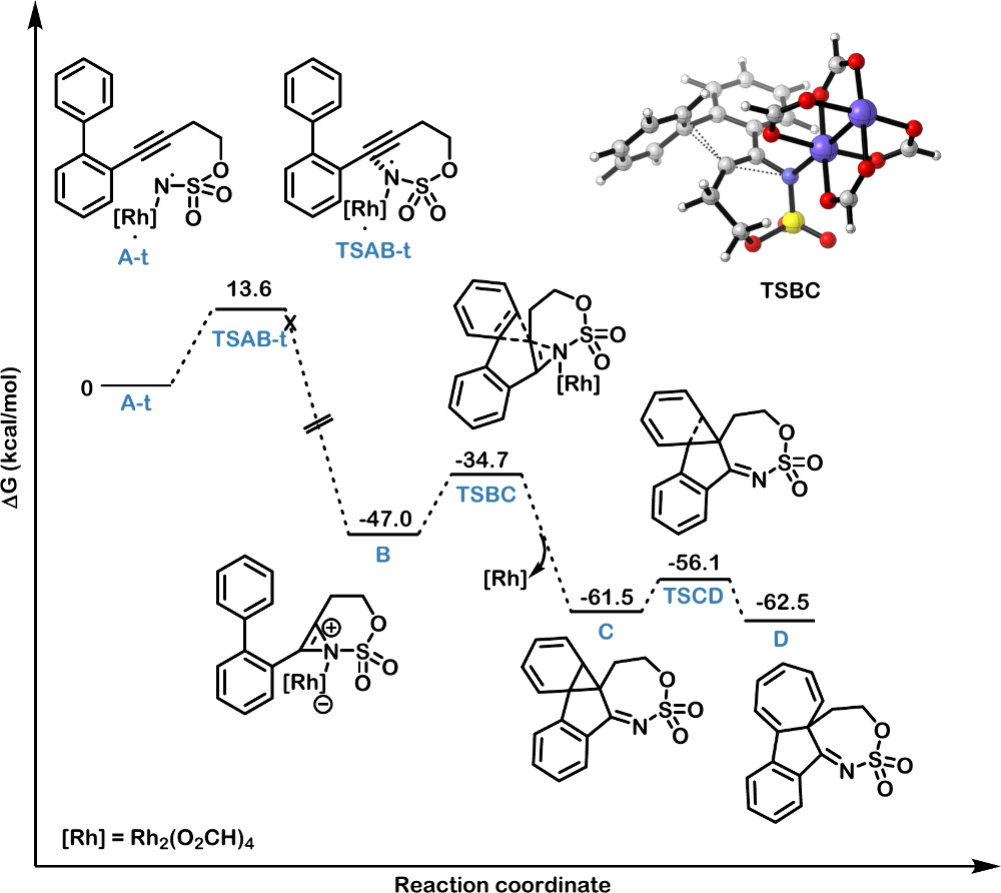
DFT-computed free-energy diagram for the nitrene–alkyne cyclization/Büchner reaction. Energies obtained at the M06-D3/6–311+G**∼sdd level of theory (with solvent corrections included).

The formation of the seven-membered sulfamate ring can be rationalized by a preferential nucleophilic attack at the carbon that ultimately becomes β to the nitrogen center initially forming the nitrene. This process resembles a conjugate-type addition to the vinylogous position of a putative vinylnitrene (or vinylcarbene) intermediate. The subsequent electrocyclic ring opening of intermediate **C** to form **D** is only slightly exergonic, yet proceeds readily due to a low 5.4 kcal/mol kinetic barrier to surpass **TSCD**. Interestingly, this contrasts with Panek’s precedent (Scheme 1B),^8^ where the norcaradiene intermediate was calculated to be 37 kcal/mol more stable than the rearranged cycloheptatriene, which failed to form—highlighting the unique energetic landscape and reactivity in our system.

Experimental evidence argues against the involvement of rhodium–carbene intermediates. Water had a negligible effect on the yield during optimization, and no Si–H insertion products were observed in the presence of a silane. To support the formation of norcaradiene intermediate **C**, we leveraged the known reluctance of naphthalene rings to undergo Büchner-type expansion.^11^ Sulfamic ester **1m** was synthesized and subjected to standard conditions, affording norcaradiene **4** in 42% yield (Scheme 3b). Its structure, confirmed by single-crystal X-ray diffraction, supports a mechanism involving nucleophilic attack of the aryl ring on the azirine intermediate. Single-point energy calculations for the experimentally observed product **4** and the putative Büchner-dearomatized naphthalene product indicate that **4** is 25.3 kcal/mol more stable than the corresponding dearomatized species, thereby explaining the observed reactivity. Interestingly, compound **4** spontaneously rearranged in solution to benzo­[c]­phenanthrene derivative **5**, further expanding the accessible structural diversity. Additional single-point energy calculations again rationalize this transformation, showing that **5** is 25.5 kcal/mol more stable than **4**.

## Methods

### General Procedure for Single-Carbon Insertion via Nitrene Cascade Reaction

The corresponding sulfamate **1** (5 mmol, 1 equiv) and diacetoxyiodo benzene (6 mmol, 1.2 equiv) were added to a vial containing the rhodium catalyst (0.125 mmol, 2.5% mol), followed by the addition of dichloromethane (1 mL). The reaction was left to stir at room temperature for 24 h. Upon completion of the reaction (TLC monitoring), the crude was concentrated under rotatory evaporation and was purified by column chromatography on silica gel (hexane/EtOAc) to afford the corresponding product **2**.

### Computational Details

All DFT static calculations were performed with the Gaussian 16 software package.^22^ Geometry optimizations were performed without symmetry constraints and with analytical frequency calculations for the characterization of the located stationary points. These frequencies were used to calculate unscaled zero-point energies (ZPEs) as well as thermal corrections and entropy effects at 298 K. For this calculations, we used the M06 hybrid functional of Truhlar and Zhao,^23^ together with the Grimme D3 correction term to the electronic energy.^24^ The electronic configuration of the molecular systems was described with the 6–311G,^25^ including diffuse functions (“+” keyword in Gaussian)^26^ and single first polarization functions (** notation in Gaussian),^27^ whereas for Rh atoms, the small-core quasi-relativistic Stuttgart/Dresden effective core potential, with an associated valence basis set (standard SDD keywords in Gaussian 16) were employed.^28^ Energies were obtained by single-point calculations on the optimized geometries with the B3LYP functional,^29^ with the Grimme D3 correction term, coupled with the 6–31G­(d) basis set.^25,30^ Solvent corrections were considered using the universal solvation model SMD of Cramer and Truhlar,^31^ using dichloromethane as the solvent. The reported free energies in this work include energies obtained at the M06-D3/6–311+G**∼SDD level of theory (with solvent corrections included) corrected with zero-point energies, thermal corrections and entropy effects evaluated at 298 K, achieved at B3LYP-D3/6–31G­(d)∼SDD level.

## Conclusion

In summary, we have developed a rhodium-catalyzed nitrene–alkyne cascade that enables the skeletal rearrangement of biaryl alkynylsulfamates into spirocyclic cycloheptatrienes with strong solid-state fluorescence. Emission properties are highly sensitive to both the nature and position of the substituents; notably, compound **2i**, bearing a methyl group on the aryl ring, showed a remarkably high quantum yield of 71%, underscoring the scaffold’s potential as a solid-state emitter. Mechanistic studies support a key rhodium-bound azirine intermediate that undergoes a dearomative single-carbon insertion and electrocyclic ring opening. This noncarbene-based pathway broadens the scope of nitrene-mediated rearrangements.

## Supplementary Material











## Notes

Experimental procedures, characterization data, X-ray crystallographic data, photophysical characterization and NMR spectra for all new compounds (PDF)
Cartesian coordinates of all optimized intermediates and transition states from the DFT study (XYZ)
CIF data for compound **2a** (CIF)
CIF data for compound **4** (CIF)
CIF data for compound **5** (CIF)

## Notes

Crystallographic data for compounds **2a** (CCDC-2476771), **4** (CCDC-2476772), and **5** (CCDC-2476882) can be downloaded free of charge from the Cambridge Crystallographic Data Centre (www.ccdc.cam.ac.uk).
