# Supplementary material for: Skeletal Rearrangement of Biaryls via Rhodium-Azirine Intermediate: A Route to Solid-State Emissive Polycyclic Sulfamates
Source: ACS Catal. 2025 Dec 19;16(1):189–96. doi: 10.1021/acscatal.5c06179 (PMC12772131; doi:10.1021/acscatal.5c06179)

Supplementary information

Skeletal Rearrangement of Biaryls via Rhodium-Azirine Intermediate: A Route to Solid-State Emissive Polycyclic Sulfamates

Nil Insa-Carreras[a], Àlex Díaz-Jiménez[a*], Andrea Pinto[b], Laura Rodríguez[b], Albert Poater[a], Anna Roglans[a], Anna Pla-Quintana[a*]

[a] Institut de Química Computacional i Catàlisi (IQCC) and Departament de Química, Universitat de Girona (UdG), C/ Maria Aurèlia Capmany, 69, E-17003, Girona, Spain.

[b] Departament de Química Inorgànica i Orgànica, Secció de Química Inorgànica, Universitat de Barcelona, Martí i Franquès 1-11, E-08028 Barcelona and Institut de Nanociència i Nanotecnologia (IN2UB). Universitat de Barcelona, 08028 Barcelona, Spain.

Corresponding author e-mail: anna.plaq@udg.edu

CONTENTS

S1. General materials and methods ... 3

S2. Optimization table ... 4

S3. General procedure for the synthesis of amino-biphenyls ... 5

S4. General procedure for the synthesis of iodo-biphenyls ... 9

S5. General procedure for the synthesis of biphenyl alcohols ... 13

S5.1. Procedure A ... 13

S5.2. Procedure B: ... 14

S6. Experimental procedure for the synthesis of sulfamates ... 20

S6.1. Procedure A: ... 20

S6.2. Procedure B: ... 21

S7. General procedure for single-carbon insertion via nitrene cascade reaction 28

S8. Reduction of the sulfamate 2a. ... 35

S9. Mechanistic study. ... 36

S10. Crystal structure of compund 2a with probability level of 50% ... 36

S11. Crystal structure of compound 4 with probability level of 50% ... 41

S12. Crystal structure of compound 5 with probability level of 50% ... 46

S13. Computational details ... 51

S13.1. Insight into the formation of intermediate B ... 51

S13.2. XYZ coordinates ... 52

S13.3. Insight into the thermodynamic evolution of 4 to 5 ... 59

S14. Photophysical characterization ... 62

S14.1. HOMO-LUMO orbitals ... 63

S15. References ... 69

S16. NMR spectra ... 71

S1. General materials and methods

Unless otherwise noted, materials were obtained from commercial suppliers and used without further purification. Reaction progress during the preparation of all compounds was monitored using thin layer chromatography on Macherey-Nagel Xtra SIL G/UV254 silica gel plates. Solvents were removed under reduced pressure with a rotary evaporator. Reaction mixtures were chromatographed on silica gel using an automated purification instrument Interchim PuriFlash XS 520 Plus equipped with a quaternary gradient pump (up to 300 ml/min, 20 bar) and an UV-Vis 200-800 nm diode array detector. All ^{1}H and ^{13}C NMR spectra were recorded on a Bruker ASCEND 400 spectrometer equipped with a 5 mm BBFO probe using $CDCl_3$ a deuterated solvent. Chemical shifts for ^{1}H and ^{13}C NMR are reported in ppm (δ) relative to residual solvent signals ($CDCl_3$: 7.26 ppm for ^{1}H, 77,16 ppm for ^{13}C). Coupling constants are given in Hertz (Hz). ^{1}H and ^{13}C NMR signals were assigned based on ^{2}D-NMR: HSQC, HMBC, COSY and TOCSY experiments. Electrospray ionization high-resolution mass spectrometry was performed using a Bruker microTOF-Q II instrument operated in the positive ESI (+) ion mode. IR spectra were recorded on an Agilent Cary 630 FT-IR spectrometer equipped with an ATR sampling accessory. Melting points were measured in a SMP10 apparatus from Stuart without any correction. Absorption spectra have been recorded on a Varian Cary 100 Bio UV spectrophotometer, and emission spectra have been recorded on a Horiba Jobin-Yvon SPEX Nanolog spectrofluorimeter. The enantiopurity of the compounds was determined on an Agilent 1260 Infinity HPLC, equipped with a Daicel Corporation Chiralpak IC chromatography column. Quantum yields have been recorded on a Hamamatsu Absolute PL Quantum Yield Spectrometer C11347. Luminescence lifetimes were measured on JYF-DELTAPRO-NL equipment upon excitation of the samples with a 390 nm NanoLED and collecting the decays through a band pass filter of 500, 550 and 600 nm.

S2. Optimization table

Table S1: Reaction optimization

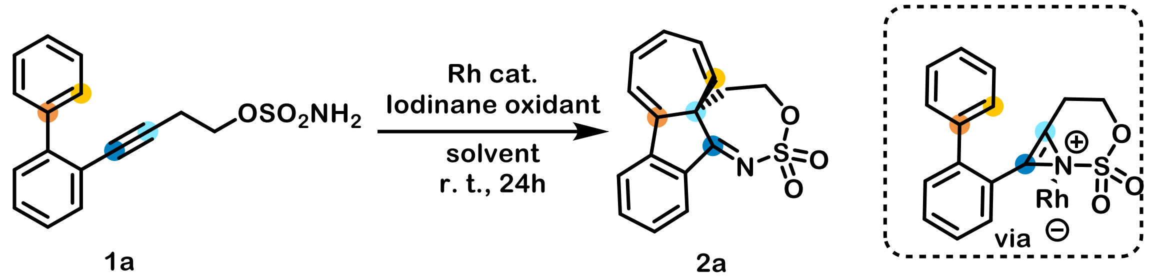

Entry	Catalyst	Cat. Loading (% mol)	Solvent	Iodinane oxidant (equiv.)	Yield (%)	e.e. (%)
**1**	Tp(CF_3)$_2$BrAg(THF)	5	DCM_{anh}	PhI(OAc)$_2$ (4.8)	n.r.	-
**2**	Rh_2(esp)$_2$	5	DCE_{anh}	PhI(OAc)$_2$ (4.8)	25	-
**3**	Rh_2(esp)$_2$	5	DCM_{anh}	PhI(OAc)$_2$ (4.8)	86	-
**4**	Rh_2(*S*-PTTL)$_4$	5	DCM_{anh}	PhI(OAc)$_2$ (4.8)	32	6
**5**	Rh_2(*R*-DOSP)$_4$	5	DCM_{anh}	PhI(OAc)$_2$ (4.8)	47	10
**6**	Rh_2(*R*-*p*-Ph-TPCP)$_4$	5	DCM_{anh}	PhI(OAc)$_2$ (4.8)	56	52
**7**[a]	Rh_2(*R*-*p*-Ph-TPCP)$_4$	5	DCM_{anh}	PhI(OAc)$_2$ (4.8)	77	58
**8**[a]	Rh_2(*R*-*p*-Ph-TPCP)$_4$	5	DCM_{anh}	PhI(OCOCF$_3$)$_2$ (4.8)	46	36
**9**	Rh_2(*R*-BTPCP)$_4$	5	DCM_{anh}	PhI(OAc)$_2$ (4.8)	97	44
**10**	Rh_2(*R*-BTPCP)$_4$	2.5	DCM	PhI(OAc)$_2$ (4.8)	99	40
**11**	**Rh_2(*R*-BTPCP)$_4$**	**2.5**	**DCM**	**PhI(OAc)$_2$ (1.2)**	**99**	**40**

a) CaO was added as additive.

We started the screening of the reaction conditions by using Tp(CF_3)$_2$BrAg(THF); however, it proved ineffective and failed to promote the desired transformation (Entry 1). We then continued the screening of the reaction conditions by using Rh_2(esp)$_2$ (5% mol) and PhI(OAc)$_2$ (4.8 eq) in anhydrous 1,2-dichloroethane (DCE) under a nitrogen atmosphere, affording a 25% yield of the desired product (Entry 2). Changing the solvent to anhydrous dichloromethane (DCM) under identical conditions led to significantly improved yield of 86% (Entry 3). Encouraged by this result, we evaluated asymmetric dirhodium catalysts to maximize both the yield and the enantioselectivity of this transformation. We first evaluated a phthalimido-derived dirhodium catalyst (Entry 4), which resulted in low yield and e.e.. Switching to a sulfonyl-based catalyst slightly improved both parameters but still failed to provide satisfactory results (Entry 5). Subsequently, a triphenylcyclopropyl (TPCP)-derived catalyst was used significantly enhancing the yield and the e.e. (Entry 6). Inspired by the work of Shi et al.[1], we added calcium oxide to quench the acetic acid formed in-situ, which further improved the yield to 77% and the e.e. to 58% (Entry 7). Testing an alternative iodinane oxidant proposed by Dauban et al.[2] led to a decreased yield and enantiomeric excess (Entry 8). Remarkably, after changing to Rh_2(*R*-BTPCP)$_4$, a 97% yield

was obtained, although the e.e. dropped to 44% (Entry 9). Further investigation was carried out, and the removal of the anhydrous conditions resulted in an increase in the yield without affecting the e.e. (Entry 10). Finally, diminishing the oxidant loading from 4.8 to 1.2 equiv. had no detrimental effect on the yield (Entry 11). Given that the e.e. could not be improved beyond 58%, we established the conditions maximizing yield as the optimized reaction conditions for this transformation (Entry 11).

S3. General procedure for the synthesis of amino-biphenyls

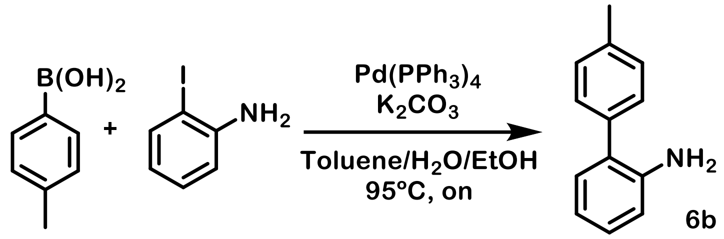

6b

A mixture of 2-iodoaniline (1.10 g, 5 mmol), (*p*-methylphenyl)boronic acid (0.75 g, 5.5 mmol), $Pd(PPh_3)_4$ (0.29 g, 0.25 mmol, 5% mol), and potassium carbonate (2.07 g, 15 mmol) was added to a 250 mL double-necked round-bottom flask containing a previously degassed mixture of toluene (60 mL), H_2O (40 mL), and ethanol (20 mL) under nitrogen atmosphere. The reaction mixture was then heated to 95°C and stirred overnight. Upon completion of the reaction (TLC and GC-MS monitoring), the crude product was washed with aqueous NH_4Cl (100 mL) and the resulting aqueous phase was further extracted with CH_2Cl_2 (3 x 20 mL). The organic phases were combined and washed with H_2O (10 mL) and saturated $NaHCO_3$ solution (10 mL), dried over anhydrous Na_2SO_4, and concentrated under reduced pressure. The crude product was purified by column chromatography on silica gel (Hexane/AcOEt = 90:10 to 75:25) to afford **6b** as an orange oil (0.82 g, 90% yield).[3]

^{1}H-NMR (400 MHz, $CDCl_3$) δ (ppm): 7.39 – 7.34 (m, 2H), 7.29 – 7.27 (m, 2H), 7.18 – 7.11 (m, 2H), 6.83 (td, *J* = 7.8, 1.2 Hz, 1H), 6.77 (dd, *J* = 7.8, 1.2 Hz, 1H), 3.75 (bs, 2H), 2.42 (s, 3H).[3]

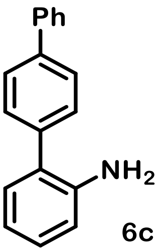

6c

^{1}H-NMR (400 MHz, $CDCl_3$) δ(ppm): 7.67 – 7.64 (m, 2H), 7.63 – 7.57 (m, 2H), 7.55 – 7.52 (m, 2H), 7.48 – 7.43 (m, 2H), 7.39 – 7.35 (m, 1H), 7.21 (dd, *J* = 7.6, 1.6 Hz, 1H), 7.11 (t, *J* = 7.6 Hz, 1H), 6.96 – 6.88 (m, 2H). [4]

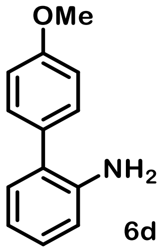

6d

^{1}H-NMR (400 MHz, $CDCl_3$) δ(ppm): 7.36 – 7.41 (m, 2H), 7.09 – 7.17 (m, 2H), 6.95 – 7.01 (m, 2H), 6.81 (td, *J* = 7.5, 1.2 Hz, 1H), 6.76 (dd, *J* = 7.9, 1.2 Hz, 1H), 3.85 (s, 3H).[3]

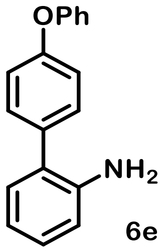

6e

^{1}H-NMR (400 MHz, $CDCl_3$) δ (ppm): 7.46 – 7.30 (m, 4H), 7.20 – 7.10 (m, 3H), 7.10 – 7.03 (m, 4H), 6.83 (td, *J* = 7.4, 1.2 Hz, 1H), 6.78 (dd, *J* = 8.0, 1.0 Hz, 1H), 3.80 (bs, 2H). [4]

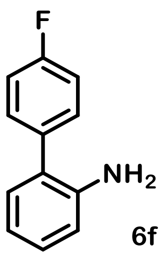

6f

^{1}H-NMR (400 MHz, $CDCl_3$) δ (ppm): 7.39 – 7.46 (m, 2H), 7.08 – 7.20 (m, 4H), 6.83 (td, *J* = 7.4, 1.2 Hz, 1H), 6.78 (dd, *J* = 8.0, 1.2 Hz, 1H), 3.77 (bs, 2H).[3]

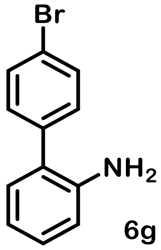

6g

^{1}H-NMR (400 MHz, $CDCl_3$) δ (ppm): 7.61 – 7.53 (m, 2H), 7.39 – 7.31 (m, 2H), 7.22 – 7.14 (m, 1H), 7.09 (dd, *J* = 7.6, 1.6 Hz, 1H), 6.83 (td, *J* = 7.5, 1.3 Hz, 1H), 6.80 – 6.74 (m, 1H), 3.72 (bs, 2H).[5]

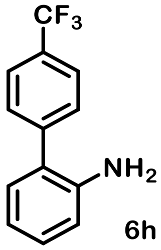

6h

^{1}H-NMR (400 MHz, $CDCl_3$) δ(ppm): 7.70 (d, *J* = 8.4 Hz, 2H), 7.60 (d, *J* = 8.4 Hz, 2H), 7.21 (td, *J* = 7.7, 1.6 Hz, 1H), 7.12 (dd, *J* = 7.6, 1.6 Hz, 1H), 6.87 (td, *J* = 7.5, 1.2 Hz, 1H), 6.81 (dd, *J* = 8.0, 1.2 Hz, 1H), 4.01 (bs, 2H). [3]

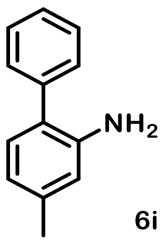

6i

^{1}H-NMR (400 MHz, $CDCl_3$) δ (ppm): 7.49 – 7.38 (m, 4H), 7.38 – 7.28 (m, 1H), 7.04 (d, *J* = 7.6 Hz, 1H), 6.66 (d, *J* = 7.6 Hz, 1H), 6.61 (s, 1H), 3.72 (bs, 2H), 2.32 (s, 3H).[4]

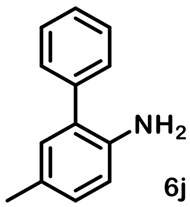

6j

^{1}H-NMR (400 MHz, $CDCl_3$) δ (ppm): 7.48 – 7.41 (m, 4H), 7.38 – 7.31 (m, 1H), 7.01 – 6.94 (m, 2H), 6.70 (d, *J* = 7.9 Hz, 1H), 3.64 (bs, 2H), 2.28 (s, 3H).[4]

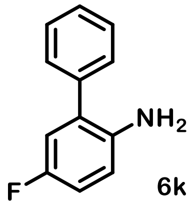

6k

^{1}H-NMR (400 MHz, $CDCl_3$) δ (ppm): 7.50 – 7.41 (m, 4H), 7.40 – 7.32 (m, 1H), 6.91 – 6.84 (m, 2H), 6.74 – 6.68 (m, 1H), 3.70 (bs, 2H).[3]

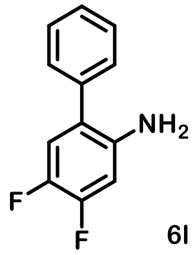

6l

^{1}H-NMR (400 MHz, CDCl$_3$) δ (ppm): 7.49 – 7.42 (m, 2H), 7.42 – 7.32 (m, 3H), 6.94 (dd, *J* = 11.0, 8.7 Hz, 1H), 6.55 (dd, *J* = 11.9, 7.0 Hz, 1H), 3.76 (bs, 2H).[7]

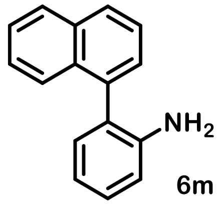

6m

^{1}H-NMR (400 MHz, CDCl$_3$) δ (ppm): 7.96 – 7.86 (m, 2H), 7.69 – 7.63 (m, 1H), 7.56 (dd, *J* = 8.3, 7.0 Hz, 1H), 7.50 (ddd, *J* = 8.3, 6.8, 1.5 Hz, 1H), 7.44 (dtd, *J* = 8.3, 6.5, 1.4 Hz, 2H), 7.27 (td, *J* = 7.6, 1.7 Hz, 1H), 7.17 (dd, *J* = 7.5, 1.7 Hz, 1H), 6.89 (td, *J* = 7.5, 1.2 Hz, 1H), 6.84 (dd, *J* = 8.0, 1.2 Hz, 1H), 3.48 (bs, 2H).[14]

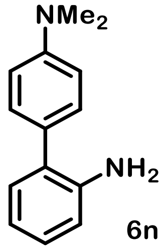

6n

^{1}H-NMR (400 MHz, CDCl$_3$) δ (ppm): 7.38 – 7.32 (m, 2H), 7.15 – 7.09 (m, 2H), 6.86 – 6.73 (m, 4H), 3.84 (bs, 2H), 3.00 (s, 6H).[6]

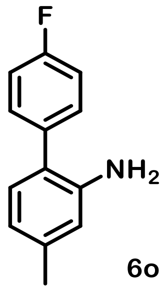

6o

^{1}H-NMR (400 MHz, $CDCl_3$) δ (ppm): 7.44 – 7.36 (m, 2H), 7.16 – 7.07 (m, 2H), 6.99 (d, *J* = 7.7 Hz, 1H), 6.65 (ddd, *J* = 7.7, 2.0, 0.8z Hz, 1H), 6.60 (d, *J* = 2.0 Hz, 1H), 3.65 (bs, 2H), 2.31 (s, 3H).[26]

S4. General procedure for the synthesis of iodo-biphenyls

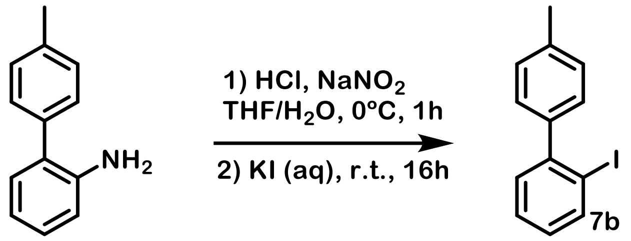

A solution of aqueous HCl 37% (1.58 mL, 19.2 mmol) was added dropwise to a 50 mL round-bottom flask immersed in an ice-water bath containing a solution of **4'-methyl-[1,1'-biphenyl]-2-amine 6b** (0.74 g, 4 mmol) in THF (4 mL) and H_2O (8 mL). Then, a solution of $NaNO_2$ (0.42 g, 6 mmol) in H_2O (2 mL) was added dropwise at the same temperature and stirred for 1h. A solution of KI (1.59 g, 9.6 mmol) in H_2O (2 mL) was then added dropwise at the same temperature. The reaction mixture was slowly warmed to room temperature and stirred for 16h. Upon completion of the reaction (TLC monitoring), the crude mixture was treated with a 1M $Na_2S_2O_3$ solution (100 mL) and extracted with EtOAc (3 x 20 mL). The organic phases were combined, dried over anhydrous Na_2SO_4, and concentrated under reduced pressure. The crude product was purified by column chromatography on silica gel (Hexane/AcOEt = 90:10 to 80:20) to afford **7b** as an orange oil (1.01 g, 86% yield).[8]

^{1}H-NMR (400 MHz, $CDCl_3$) δ (ppm): 7.95 (dd, *J* = 7.6, 1.2 Hz, 1H), 7.38 (td, *J* = 7.6, 1.2 Hz, 1H), 7.30 (dd, *J* = 7.6, 1.8 Hz, 1H), 7.24 (s, 4H), 7.02 (td, *J* = 7.6, 1.8 Hz, 1H), 2.42 (s, 3H).[8]

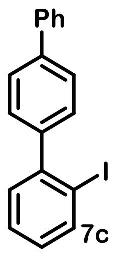

^{1}H-NMR (400 MHz, $CDCl_3$) δ(ppm): 7.98 (dd, *J* = 7.9, 1.2 Hz, 1H), 7.69 – 7.63 (m, 4H), 7.50 – 7.41 (m, 5H), 7.40 – 7.33 (m, 2H), 7.04 (td, J = 7.2, 2.0 Hz, 1H).[8]

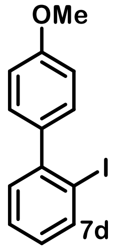

7d

^{1}H-NMR (400 MHz, $CDCl_3$) δ(ppm): 7.95 (dd, *J* = 8.0, 1.2 Hz, 1H), 7.37 (td, *J* = 7.6, 1.2 Hz, 1H), 7.26 – 7.31 (m, 3H), 7.01 (td, *J* = 7.6, 2.0 Hz, 1H), 6.94 – 6.98 (m, 2H), 3.87 (s, 3H).[8]

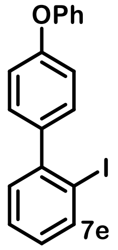

7e

^{1}H-NMR (400 MHz, $CDCl_3$) δ(ppm): 7.96 (dd, *J* = 7.9, 1.2 Hz, 1H), 7.43 – 7.35 (m, 3H), 7.35 – 7.28 (m, 3H), 7.17 – 7.13 (m, 1H), 7.12 – 7.02 (m, 5H).[9]

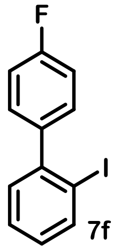

7f

^{1}H-NMR (400 MHz, $CDCl_3$) δ(ppm): 7.95 (dd, *J* = 8.0, 1.6 Hz, 1H), 7.39 (td, *J* = 7.2, 1.2 Hz, 1H), 7.26 – 7.33 (m, 3H), 7.08 – 7.15 (m, 2H), 7.04 (td, *J* = 7.6, 2.0 Hz, 1H). [10]

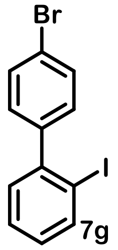

7g

^{1}H-NMR (400 MHz, $CDCl_3$) δ(ppm): 7.96 (dd, *J* = 8.0, 1.2 Hz, 1H), 7.60 – 7.49 (m, 2H), 7.42 – 7.37 (m, 1H), 7.28 – 7.26 (m, 1H), 7.24 – 7.20 (m, 2H), 7.05 (td, *J* = 7.7, 1.7 Hz, 1H).[11]

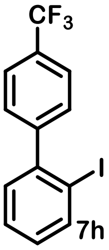

7h

^{1}H-NMR (400 MHz, $CDCl_3$) δ(ppm): 7.98 (dd, *J* = 7.7, 1.2 Hz, 1H), 7.69 (d, *J* = 8.1 Hz, 2H), 7.47 (d, *J* = 8.1 Hz, 2H), 7.42 (td, *J* = 7.7, 1.2 Hz, 1H), 7.29 (dd, *J* = 7.6, 1.7 Hz, 1H), 7.08 (td, *J* = 7.6, 1.7 Hz, 1H).[10]

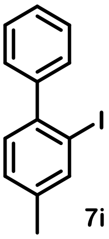

7i

^{1}H-NMR (400 MHz, $CDCl_3$) δ(ppm): 7.80 (s, 1H), 7.45 – 7.31 (m, 5H), 7.20 (s, 2H), 2.36 (s, 3H).[8]

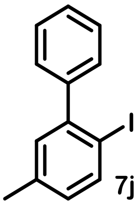

7j

^{1}H-NMR (400 MHz, $CDCl_3$) δ(ppm): 7.82 (d, *J* = 8.4 Hz, 1H), 7.48 – 7.29 (m, 5H), 7.14 (d, *J* = 2.2 Hz, 1H), 6.87 (dd, *J* = 8.4, 2.2 Hz, 1H), 2.33 (s, 3H).[8]

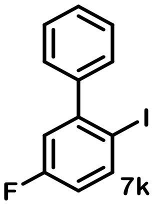

7k

^{1}H-NMR (400 MHz, $CDCl_3$) δ (ppm): 7.88 (dd, *J* = 8.7, 5.6 Hz, 1H), 7.47 – 7.39 (m, 3H), 7.35 – 7.29 (m, 2H), 7.06 (dd, *J* = 9.3, 3.0 Hz, 1H), 6.81 (td, *J* = 8.4, 3.0 Hz, 1H).[8]

Following the **Procedure** and starting from **4,5-difluoro-[1,1'-biphenyl]-2-amine 6l** (0.74 g, 3.63 mmol), compound **4,5-difluoro-2-iodo-1,1'-biphenyl 7l** was obtained as an orange oil (1.04 g, 91 % yield).

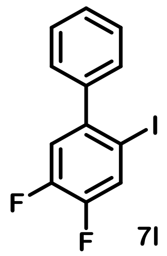

7l

Formula: $C_{12}H_7F_2I$; **MW:** 316.09 g/mol; **^{1}H-NMR (400 MHz, $CDCl_3$) δ (ppm):** 7.74 (dd, J = 9.2, 8.3 Hz, 1H), 7.48 – 7.39 (m, 3H), 7.33 – 7.27 (m, 2H), 7.15 (dd, J = 10.1, 8.8 Hz, 1H); **^{13}C-NMR (101 MHz, $CDCl_3$) δ (ppm):** 150.3 (dd, $^1J_{C\text{-}F}$ = 253.4, $^2J_{C\text{-}F}$ = 14.3 Hz), 149.3 (dd, $^1J_{C\text{-}F}$ = 256.0, $^2J_{C\text{-}F}$ = 14.3 Hz), 143.7 (t, $^3J_{C\text{-}F}$ = $^4J_{C\text{-}F}$ = 4.9 Hz), 142.6, 129.3, 128.4, 128.3, 127.9 (dd, $^2J_{C\text{-}F}$ = 11.3, $^3J_{C\text{-}F}$ = 8.4 Hz), 118.5 (dd, $^2J_{C\text{-}F}$ = 14.7, $^3J_{C\text{-}F}$ = 3.8 Hz), 90.1 (t, $^3J_{C\text{-}F}$ = $^4J_{C\text{-}F}$ = 4.8 Hz).; **^{19}F-NMR (377 MHz, $CDCl_3$) δ (ppm):** -138.79, -138.78; **HRMS (ESI) m/z:** This product did not ionize in the HRMS instrument .

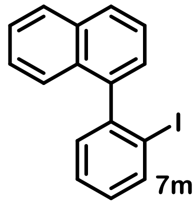

7m

^{1}H-NMR (400 MHz, $CDCl_3$) δ (ppm): 8.02 (dd, J = 7.9, 1.2 Hz, 1H), 7.95 – 7.89 (m, 2H), 7.59 – 7.38 (m, 5H), 7.34 (ddd, J = 10.5, 7.2, 1.5 Hz, 2H), 7.17 – 7.11 (m, 1H).[15]

Following the **Procedure** and starting from **N4',N4'-dimethyl-[1,1'-biphenyl]-2,4'-diamine 6n** (0.94 g, 4.44 mmol), **2-iodo-N,N-dimethyl-[1,1'-biphenyl]-4-amine 7n** was obtained as a yellow oil (0.97 g, 68% yield).

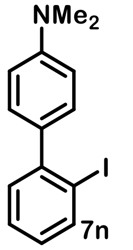

7n

Formula: $C_{14}H_{14}IN$; **MW:** 323.18 g/mol; **^{1}H-NMR (400 MHz, $CDCl_3$) δ (ppm):** 7.94 (dd, J = 7.8, 1.3 Hz, 1H), 7.39 – 7.29 (m, 2H), 7.28 – 7.21 (m, 2H), 6.97 (ddd, J = 7.8, 7.1, 2.0 Hz, 1H), 6.78 (d, J = 8.3 Hz, 2H), 3.02 (s, 6H); **^{13}C-NMR (101**

MHz, CDCl3) δ (ppm): 146.7, 139.5, 130.3, 130.1 , 128.7, 128.1, 128.0, 126.3, 111.5, 99.5, 40.5; **HRMS (ESI) m/z:** found: 324.0245, calculated for $[M + H]^+$ = 324.0244.

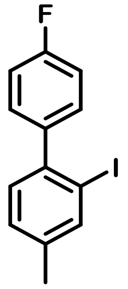

7o

^{1}H-NMR (400 MHz, $CDCl_3$) δ (ppm): 7.79 (s, 1H), 7.34 – 7.27 (m, 2H), 7.22 – 7.13 (m, 2H), 7.13 – 7.05 (m, 2H), 2.35 (s, 3H).

S5. General procedure for the synthesis of biphenyl alcohols

S5.1. Procedure A:

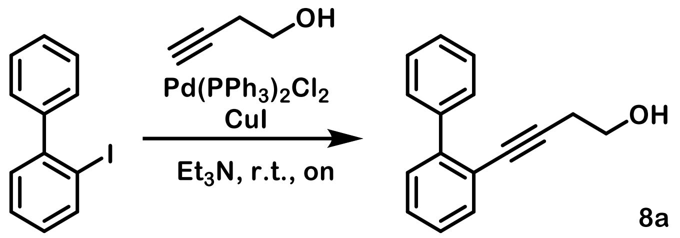

3-Butyn-1-ol (0.38 mL, 5.11 mmol, 1.2 eq) was added dropwise to a 100 mL round-bottom flask equipped with a magnetic stirrer containing a mixture of **2-iodo-1,1-biphenyl** (0.75 mL, 4.26 mmol, 1 eq), $Pd(PPh_3)_2Cl_2$ (74.8 mg, 0.106 mmol, 2.5% mol), CuI (32.5 mg, 0.17 mmol, 4% mol) and triethylamine (25 mL), under a nitrogen atmosphere. The solution was then stirred at room temperature for 16h. Upon completion (TLC monitoring), the crude was filtered through a Celite pad and concentrated under reduced pressure. The crude product was then purified by column chromatography on silica gel (Hexane/AcOEt = 95:5 to 85:15) to afford **4-[(1,1-biphenyl)-2-yl]-but-3-yn-1-ol 8a** as an orange oil (0.55 g, 58% yield).

^{1}H-NMR (400 MHz, $CDCl_3$) δ(ppm): 2.55 (t, J = 6.1 Hz, 2H), 3.61 (q, J = 6.1 Hz, 2H), 7.27 – 7.32 (m, 1H), 7.34 – 7.37 (m, 2H), 7.37 – 7.46 (m, 3H), 7.54 (m, 3H).[12]

S5.2. Procedure B:

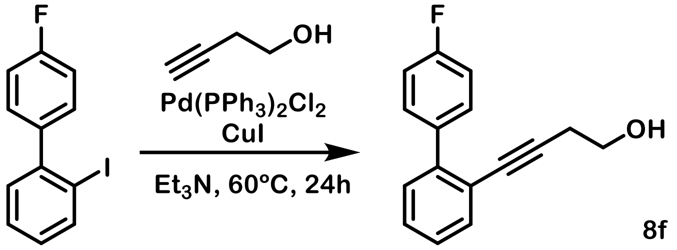

8f

3-Butyn-1-ol (0.21 mL, 2.77 mmol, 1.2 eq) was added dropwise to a 100 mL round-bottom flask equipped with a magnetic stirrer containing a mixture of **4'-fluoro-2-iodo-1,1-biphenyl 7f** (0.70 g, 2.35 mmol, 1 eq), $Pd(PPh_3)_2Cl_2$ (41.4 mg, 0.059 mmol, 2.5% mol), CuI (17.8 mg, 0.093 mmol, 4% mol) and triethylamine (25 mL) under a nitrogen atmosphere. The solution was then stirred at 60ºC for 24h. Upon completion (TLC monitoring), the crude was filtered through a Celite pad and concentrated under reduced pressure. The crude product was then purified by column chromatography on silica gel (Hexane/AcOEt = 95:5 to 85:15) to afford **4-[4'-fluoro-(1,1'-biphenyl)-2-yl]but-3-yn-1-ol 8f** as a yellow oil (0.55 g, 97% yield).

Formula: $C_{16}H_{13}OF$; **MW:** 240.28 g/mol; **^{1}H-NMR (400 MHz, $CDCl_3$) δ (ppm):** 7.50 – 7.56 (m, 3H), 7.27 – 7.39 (m, 3H), 7.08 – 7.15 (m, 2H), 3.65 (q, *J* = 6.1 Hz, 2H), 2.57 (t, *J* = 6.1 Hz, 2H); **^{13}C-NMR (101 MHz, $CDCl_3$) δ (ppm):** 162.5 (d, $^1J_{C-F}$ = 247.5 Hz), 143.0, 136.9 (d, $^4J_{C-F}$ = 3.3 Hz), 133.10, 130.9 (d, $^3J_{C-F}$ = 7.9 Hz), 129.5, 128.4, 127.3, 121.8, 115.0 (d, $^2J_{C-F}$ = 21.4 Hz), 89.8, 82.2, 61.1, 24.1; **^{19}F-NMR (377 MHz, $CDCl_3$) δ (ppm):** -115.81; **HRMS (ESI) m/z:** found: 241.1026, calculated for $[M + H]^+$ = 241.1023**; IR (ATR) υ (cm^{-1}):** 3320 (broad).

Following **Procedure A** and starting from **2-iodo-4'-methyl-1,1'-biphenyl 7b** (0.8 g, 2.72 mmol), compound **4-[4'-methyl-(1,1'-biphenyl)-2-yl]but-3-yn-1-ol 8b** was obtained as an orange oil (0.56 g, 87% yield).

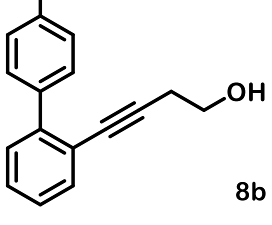

8b

Formula: $C_{17}H_{16}O$; **MW:** 236.31 g/mol; **^{1}H-NMR (400 MHz, $CDCl_3$) δ(ppm):** 7.51 (d, *J* = 8.0 Hz, 1H), 7.46 (d, *J* = 8.0 Hz, 2H), 7.35 (d, *J* = 4.0 Hz, 2H), 7.30 – 7.26 (m, 1H), 7.25 – 7.22 (m, 2H), 3.64 (q, *J* = 6.2 Hz, 2H), 2.57 (t, *J* = 6.2 Hz, 2H), 2.41 (s, 3H); **^{13}C-NMR (101 MHz, $CDCl_3$) δ (ppm):** 144.1, 138.1, 137.4, 132.9, 129.5, 129.1, 128.8, 128.3, 127.0, 121.8, 89.5, 82.6, 61.2, 24.2, 21.4; **HRMS**

(ESI) m/z: found: 237.1271, calculated for $[M + H]^+$ = 237.1274; **IR (ATR) υ (cm^{-1}):** 3335 (broad).

Following **Procedure A** and starting from **2-iodo-4'-phenyl-1,1'-biphenyl 7c** (0.51 g, 1.43 mmol), compound **4-[4'-phenyl-(1,1'-biphenyl)-2-yl]but-3-yn-1-ol 8c** was obtained as a yellow solid (0.37 g, 87% yield).

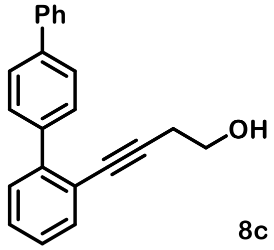

8c

Formula: $C_{22}H_{18}O$; **MW:** 298.39 g/mol; **m.p.:** 88-90 ºC; **^{1}H-NMR (400 MHz, $CDCl_3$) δ(ppm):** 7.70 – 7.63 (m, 6H), 7.55 (dd, *J* = 8.0, 1.2 Hz, 1H), 7.46 (td, *J* = 6.8, 1.6 Hz, 2H), 7.44 – 7.39 (m, 1H), 7.39 – 7.34 (m, 2H), 7.30 (td, *J* = 7.4, 1.8 Hz, 1H), 3.66 (q, *J* = 6.0 Hz, 2H), 2.59 (t, *J* = 6.0 Hz, 2H); **^{13}C-NMR (101 MHz, $CDCl_3$) δ (ppm):** 143.6, 140.9, 140.5, 139.9, 133.1, 129.7, 129.5, 129.0, 128.4, 127.5, 127.3, 127.2, 126.8, 121.9, 89.8, 82.4, 61.2, 24.2; **HRMS (ESI) m/z:** found: 337.0993, calculated for $[M + K]^+$ = 337.0989; **IR (ATR) υ (cm^{-1}):** 3320 (broad).

Following **Procedure A** and starting from **2-iodo-4'-methoxy-1,1'-biphenyl 7d** (0.45 g, 1.45 mmol), compound **4-[4'-methoxy-(1,1'-biphenyl)-2-yl]but-3-yn-1-ol 8d** was obtained as an orange oil (0.35 g, 96% yield).

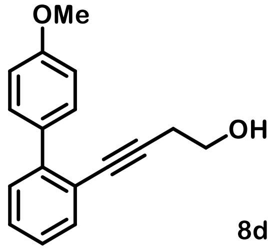

8d

Formula: $C_{17}H_{16}O_2$; **MW:** 252.31 g/mol; **^{1}H-NMR (400 MHz, $CDCl_3$) δ(ppm):** 7.54 – 7.49 (m, 3H), 7.35 – 7.32 (m, 2H), 7.28 – 7.23 (m, 1H), 6.99 – 6.94 (m, 2H), 3.86 (s, 3H), 3.66 (q, *J* = 6.0 Hz, 2H), 2.58 (t, *J* = 6.0 Hz, 2H); **^{13}C-NMR (101 MHz, $CDCl_3$) δ (ppm):** 159.3, 143.7, 133.4, 133.0, 130.4, 129.4, 128.3, 126.8, 121.7, 113.5, 89.5, 82.6, 61.2, 55.5, 24.2; **HRMS (ESI) m/z:** found: 253.1223, calculated for $[M + H]^+$ = 253.1223; **IR (ATR) υ (cm^{-1}):** 3351 (broad).

Following **Procedure A** and starting from **2-iodo-4'-phenoxy-1,1'-biphenyl 7e** (0.51 g, 1.37 mmol), compound **4-[4'-phenoxy-(1,1'-biphenyl)-2-yl]but-3-yn-1-ol 8e** was obtained as an orange oil (0.37 g, 86% yield).

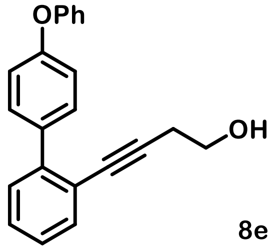

8e

Formula: $C_{22}H_{18}O_2$; **MW:** 314.38 g/mol; **^{1}H-NMR (400 MHz, $CDCl_3$) δ (ppm):** 7.55 (td, *J* = 7.0, 1.6 Hz, 3H), 7.42 – 7.34 (m, 4H), 7.31 – 7.26 (m, 1H), 7.17 – 7.05 (m, 5H), 3.67 (t, *J* = 6.1 Hz, 2H), 2.59 (t, *J* = 6.1 Hz, 2H); **^{13}C-NMR (101 MHz, $CDCl_3$) δ (ppm):** 157.1, 157.0, 143.3, 135.8, 133.0, 130.6, 129.9, 129.4, 128.3, 127.0, 123.6, 121.8, 119.3, 118.2, 89.7, 82.3, 61.1, 24.1; **HRMS (ESI) m/z:** found: 315.1385, calculated for $[M + H]^+$ = 315.1380; **IR (ATR) ʋ (cm^{-1}):** 3342 (broad).

Following **Procedure B** and starting from **2-iodo-4'-bromo-1,1'-biphenyl 7g** (1.13 g, 3.15 mmol), compound **4-[4'-bromo-(1,1'-biphenyl)-2-yl]but-3-yn-1-ol 8g** was obtained as a yellow oil (0.84 g, 88% yield).

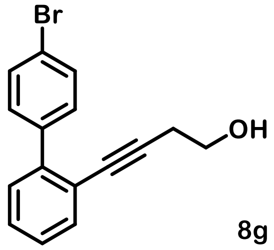

8g

Formula: $C_{16}H_{13}OBr$; **MW:** 301.18 g/mol; **^{1}H-NMR (400 MHz, $CDCl_3$) δ (ppm):** 7.57 – 7.51 (m, 3H), 7.47 – 7.43 (m, 2H), 7.37 – 7.28 (m, 3H), 3.67 (t, *J* = 6.2 Hz, 2H), 2.57 (t, *J* = 6.2 Hz, 2H); **^{13}C-NMR (101 MHz, $CDCl_3$) δ (ppm):** 142.6, 139.7, 133.2, 131.2, 131.0, 129.3, 128.4, 127.5, 121.8, 121.7, 90.0, 81.9, 61.1, 24.0; **HRMS (ESI) m/z:** found: 301.0228-303.0203, calculated for $[M + H]^+$ = 301.0223-303.0203; **IR (ATR) ʋ (cm^{-1}):** 3335 (broad).

Following **Procedure B** and starting from **2-iodo-4'-(trifluoromethyl)-1,1'-biphenyl 7h** (0.76 g, 2.18 mmol), compound **4-[4'-(trifluoromethyl)-(1,1-biphenyl)-2-yl]but-3-yn-1-ol 8h** was obtained as a red oil (0.63 g, 99% yield).

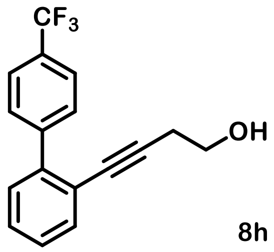

8h

Formula: $C_{17}H_{13}OF_3$; **MW:** 290.29 g/mol; **^{1}H-NMR (400 MHz, $CDCl_3$) δ(ppm):** 7.69 (s, 4H), 7.55 (d, *J* = 7.6 Hz, 1H), 7.42 – 7.30 (m, 3H), 3.66 (q, *J* = 6.2 Hz, 2H), 2.57 (t, *J* = 6.2 Hz, 2H); **^{13}C-NMR (101 MHz, $CDCl_3$) δ (ppm):** 144.5, 142.5, 133.3, 129.7, 129.7 (q, $^2J_{C-F}$ = 32.6 Hz), 129.4, 128.5, 127.9, 125.0 (q, $^3J_{C-F}$ = 3.8 Hz), 124.4 (q, $^1J_{C-F}$ = 272.7 Hz), 121.9, 90.3, 81.7, 61.1, 24.0; **^{19}F-NMR (377 MHz, $CDCl_3$) δ (ppm):** -63.4; **HRMS (ESI) m/z:** found: 291.0996, calculated for $[M + H]^+$ = 291.0991; **IR (ATR) υ (cm^{-1}):** 3353 (broad).

Following **Procedure A** and starting from **2-iodo-4-methyl-1,1'-biphenyl 7i** (1.68 g, 5.71 mmol) compound **4-[4-methyl-(1,1-biphenyl)-2-yl]but-3-yn-1-ol 8i** was obtained as an orange oil (0.71 g, 52% yield).

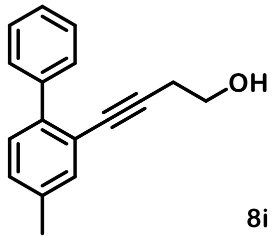

8i

Formula: $C_{17}H_{16}O$; **MW:** 236.31 g/mol; **^{1}H-NMR (400 MHz, $CDCl_3$) δ(ppm):** 7.57 – 7.52 (m, 2H), 7.45 – 7.39 (m, 2H), 7.39 – 7.33 (m, 2H), 7.27 - 7.23 (m, 1H), 7.17 (dd, *J* = 7.8, 1.1 1H), 3.62 (q, *J* = 6.0 Hz, 2H), 2.55 (t, *J* = 6.0 Hz, 2H), 2.36 (s, 3H); **^{13}C-NMR (101 MHz, $CDCl_3$) δ (ppm):** 141.3, 141.0, 137.0, 133.4, 129.4, 129.3, 129.2, 128.1, 127.4, 121.6, 89.2, 82.6, 61.1, 24.1, 21.0; **HRMS (ESI) m/z:** found: 237.1268, calculated for $[M + H]^+$ = 237.1274; **IR (ATR) υ (cm^{-1}):** 3339 (broad).

Following **Procedure A** and starting from **2-iodo-5-methyl-1,1'-biphenyl 7j** (3.97 g, 13.49 mmol), compound **4-[5-methyl-(1,1-biphenyl)-2-yl]but-3-yn-1-ol 8j** was obtained as an orange oil (2.60 g, 82% yield).

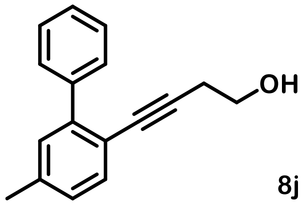

8j

Formula: $C_{17}H_{16}O$; **MW:** 236.31 g/mol; **^{1}H-NMR (400 MHz, $CDCl_3$) δ (ppm):** 7.57 – 7.52 (m, 2H), 7.46 – 7.33 (m, 4H), 7.17 (s, 1H), 7.10 (dd, *J* = 8.0, 1.8 Hz, 1H), 3.61 (q, *J* = 6.0 Hz, 2H), 2.54 (t, *J* = 6.0 Hz, 2H), 2.39 (s, 3H); **^{13}C-NMR (101 MHz, $CDCl_3$) δ (ppm):** 144.0, 141.2, 138.3, 132.7, 130.3, 129.2, 128.1, 128.0, 127.6, 118.9, 88.7, 82.5, 61.1, 24.1, 21.5; **HRMS (ESI) m/z:** found: 237.1274, calculated for $[M + H]^+$ = 237.1274; **IR (ATR) ʋ (cm^{-1}):** 3335 (broad).

Following **Procedure B** and starting from **5-fluoro-2-iodo-1,1'-biphenyl 7k** (0.60 g, 2.01 mmol), compound **4-[5-fluoro-(1,1-biphenyl)-2-yl]but-3-yn-1-ol 8k** was obtained as an orange oil (0.44 g, 91% yield).

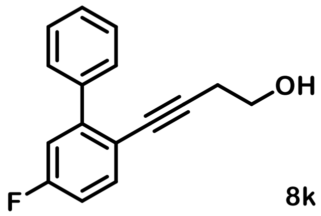

8k

Formula: $C_{16}H_{13}FO$; **MW:** 240.28 g/mol; **^{1}H-NMR (400 MHz, $CDCl_3$) δ (ppm):** 7.57 – 7.52 (m, 2H), 7.52 – 7.37 (m, 4H), 7.07 (dd, *J* = 9.5, 2.7 Hz, 1H), 6.99 (td, *J* = 8.3, 2.7 Hz, 1H), 3.61 (t, *J* = 6.0 Hz, 2H), 2.54 (t, *J* = 6.0 Hz, 2H); **^{13}C-NMR (101 MHz, $CDCl_3$) δ (ppm):** 162.2 (d, $^1J_{C-F}$ = 250.3 Hz), 146.3 (d, $^3J_{C-F}$ = 8.1 Hz), 140.0 (d, $^4J_{C-F}$ = 1.7 Hz), 134.6 (d, $^3J_{C-F}$ = 8.6 Hz), 129.1, 128.2, 128.1, 118.0 (d, $^4J_{C-F}$ = 3.2 Hz), 116.5 (d, $^2J_{C-F}$ = 22.4 Hz), 114.4 (d, $^2J_{C-F}$ = 21.9 Hz), 89.2 (d, $^5J_{C-F}$ = 1.7 Hz), 81.4, 61.1, 24.0; **^{19}F NMR (377 MHz, $CDCl_3$) δ:** -112.62; **HRMS (ESI) m/z:** found: 241.1021, calculated for $[M + H]^+$ = 241.1023; **IR(ATR) ʋ (cm^{-1}):** 3331 (broad).

Following **Procedure B** and starting from **4,5-difluoro-2-iodo-1,1'-biphenyl 7l** (0.85 g, 2.69 mmol), compound **4-[4,5-difluoro-(1,1-biphenyl)-2-yl]but-3-yn-1-ol 8l** was obtained as an orange oil (0.58 g, 83% yield).

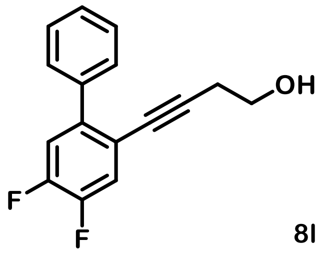

8l

Formula: $C_{16}H_{12}F_2O$; **MW:** 258.27 g/mol; **^{1}H-NMR (400 MHz, $CDCl_3$) δ (ppm):** 7.54 – 7.47 (m, 2H), 7.47 – 7.35 (m, 3H), 7.31 (dd, *J* = 10.9, 8.0 Hz, 1H), 7.16 (dd, *J* = 10.9, 8.0 Hz, 1H), 3.61 (t, *J* = 6.0 Hz, 2H), 2.53 (t, *J* = 6.0 Hz, 2H); **^{13}C-NMR (101 MHz, $CDCl_3$) δ (ppm):** 150.0 (dd, $^1J_{C-F}$ = 252.5, $^2J_{C-F}$ = 12.7 Hz) **,**149.1 (dd, $^1J_{C-F}$ = 250.1, $^2J_{C-F}$ = 13.2), 141.4 (dd, $^3J_{C-F}$ = 6.0, $^4J_{C-F}$ = 3.6 Hz), 139.2 (d,

$^{3}J_{C-F}$ = 1.5 Hz), 129.2, 128.3, 128.2, 121.3 (d, $^{2}J_{C-F}$ = 18.4 Hz), 118.5 (dd overlaped), 118.4 (d, $^{2}J_{C-F}$ = 18.3 Hz), 90.3 (d, $^{5}J_{C-F}$ = 1.9 Hz), 80.5 (t, $^{4}J_{C-F}$ = $^{5}J_{C-F}$ = 2.1 Hz), 61.0, 24.0; **^{19}F NMR (377 MHz, CDCl$_3$) δ:** -137.25 (d, *J* = 22.2 Hz), -140.69 (d, *J* = 22.0 Hz); **HRMS (ESI) m/z:** found: 259.0930, calculated for [M + H]$^+$ = 259.0929; **IR (ATR) υ (cm^{-1}):** 3346 (broad).

Following **Procedure A** and starting from **1-(2-iodophenyl)naphthalene 7m** (1.81 g, 5.5 mmol), compound **4-(2-(naphthalen-1-yl)phenyl)but-3-yn-1-ol 8m** was obtained as an orange oil (1.07 g, 72% yield).

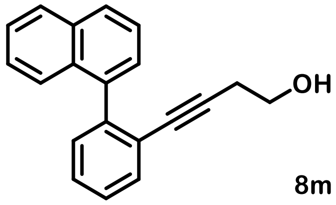

8m

Formula: $C_{20}H_{16}O$; **MW:** 272.35 g/mol; **^{1}H-NMR (400 MHz, CDCl$_3$) δ (ppm):** 7.95 – 7.88 (m, 2H), 7.61 (dd, *J* = 8.3, 1.1 Hz, 1H), 7.58 – 7.48 (m, 3H), 7.44 – 7.36 (m, 5H), 3.09 – 3.02 (m, 2H), 2.22 (t, *J* = 5.8 Hz, 2H); **^{13}C-NMR (101 MHz, CDCl$_3$) δ (ppm):** 143.3, 139.4, 133.5, 131.9, 131.8, 130.5, 128.4, 128.1, 128.1, 127.6, 127.1, 126.5, 126.0, 126.0, 125.4, 123.9, 90.4, 82.1, 60.8, 23.9; **HRMS (ESI) m/z:** found: 273.1280, calculated for [M + H]$^+$ = 273.1274; **IR (ATR) υ (cm^{-1}):** 3334 (broad).

Following **Procedure A** and starting from **2-iodo-N,N-dimethyl-[1,1'-biphenyl]-4-amine 7n** (0.79 g, 2.44 mmol), compound **4-[4'-dimethylamino-(1,1-biphenyl)-2-yl]but-3-yn-1-ol 8n** was obtained as a dark oil (0.52 g, 80% yield).

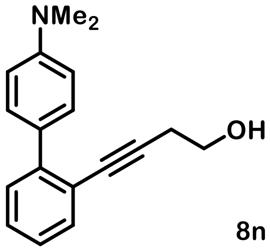

8n

Formula: $C_{18}H_{19}NO$; **MW:** 265.36 g/mol; **^{1}H-NMR (400 MHz, CDCl$_3$) δ (ppm):** 7.56 – 7.48 (m, 3H), 7.41 – 7.30 (m, 2H), 7.28 – 7.19 (m, 1H), 6.82 (d, *J* = 8.3 Hz, 2H), 3.70 (q, *J* = 6.0 Hz, 2H), 3.03 (s, 5H), 2.62 (t, *J* = 6.0 Hz, 2H); **^{13}C-NMR (101 MHz, CDCl$_3$) δ (ppm):** 150.0, 144.1, 133.0, 130.0, 129.3, 128.2, 126.2, 121.5, 112.0, 89.2, 83.0, 61.2, 40.7, 24.3; **HRMS (ESI) m/z:** found: 266.1541, calculated for [M + H]$^+$ = 266.1539; **IR (ATR) υ (cm^{-1}):** 3335 (broad).

Following **Procedure B** and starting from **4'-fluoro-2-iodo-4-methyl-1,1'-biphenyl 7o** (1.30 g, 4.16 mmol), compound **4-[4'-fluoro-(1,1'-biphenyl)-2-yl]but-3-yn-1-ol 8o** was obtained as a dark oil (0.35 g, 33% yield).

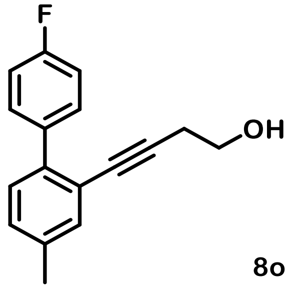

8o

Formula: $C_{17}H_{15}FO$; **MW:** 254.30 g/mol; **m.p.:** 52 - 54ºC; **^{1}H-NMR (400 MHz, $CDCl_3$) δ (ppm):** 7.55 – 7.46 (m, 2H), 7.35 (d, *J* = 2.0 Hz, 1H), 7.22 (d, *J* = 7.8 Hz, 1H), 7.16 (dd, *J* = 7.8, 2.0 Hz, 1H), 7.13 – 7.06 (m, 2H), 3.66 (t, *J* = 6.2 Hz, 2H), 2.57 (t, *J* = 6.2 Hz, 2H)z, 2.36 (s, 3H); **^{13}C-NMR (101 MHz, $CDCl_3$) δ (ppm):** 162.4 (d, $^1J_{C-F}$ = 247.1 Hz), 140.2, 137.1, 136.9 (d, $^4J_{C-F}$ = 3.2 Hz), 133.6, 130.9 (d, $^3J_{C-F}$ = 8.1 Hz), 129.4, 129.3, 121.5, 114.9 (d, $^2J_{C-F}$ = 21.4 Hz), 89.3, 82.3, 61.2, 24.1, 21.0; **^{19}F NMR (377 MHz, $CDCl_3$) δ:** -116.18; **IR (ATR) υ (cm^{-1}):** 3347 (broad).

S6. Experimental procedure for the synthesis of sulfamates

S6.1. Procedure A:

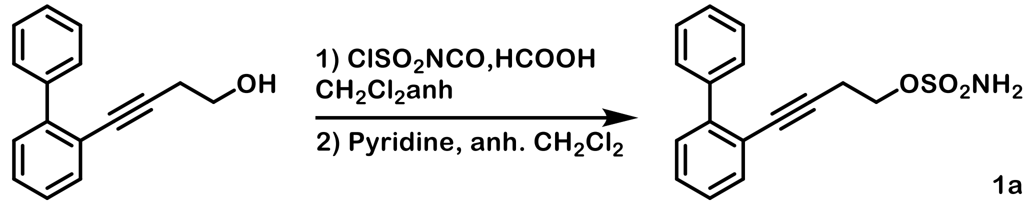

1a

Formic acid (0.17 mL, 4.51 mmol) was added dropwise to a 50 mL round-bottom flask equipped with a magnetic stirrer containing chlorosulfonyl isocyanate (0.39 mL, 4.44 mmol), at 0ºC under nitrogen atmosphere. The resulting colourless solid was stirred for 30 min and dissolved with anhydrous dichloromethane (3 mL). The solution was then slowly warmed to room temperature and a mixture of pyridine (0.36 mL, 4.44 mmol), **4-([1,1'-biphenyl]-2-yl)but-3-yn-1-ol 8a** (0.7g, 3.15 mmol) in anhydrous dichloromethane (2.5 mL) was added. The reaction mixture was stirred for 3h until completion of the reaction (TLC monitoring). The crude was quenched by the addition of water and extracted with EtOAc (3 x 20 mL). The combined organic phases were rinsed with brine, dried over Na_2SO_4, and concentrated under reduced pressure. The crude product was purified by column chromatography on silica gel (Hexane/AcOEt = 70:30) to afford **4-([1,1'-biphenyl]-2-yl)but-3-yn-1-yl sulfamate 1a** as a colourless solid (0.27 g, 28% yield).

Formula: $C_{16}H_{15}O_3NS$; **MW:** 301.36 g/mol; **m.p.:** 94 – 96 ºC; **^{1}H-NMR (400 MHz, $CDCl_3$) δ (ppm):** 2.78 (t, *J* = 6.5 Hz, 2H), 4.20 (t, *J* = 6.5 Hz, 2H), 4.41 (s, 2H), 7.29 (m, 1H), 7.34 – 7.41 (m, 3H), 7.42 – 7.48 (m, 2H), 7.50 – 7.60 (m, 3H); **^{13}C-NMR (101 MHz, $CDCl_3$) δ (ppm):** 143.8, 140.8, 133.3, 129.7, 129.5, 128.7, 128.2, 127.7, 127.3, 121.3, 87.3, 82.4, 68.7, 20.6; **HRMS (ESI) m/z:** found: 324.0663, calculated for $[M + Na]^+$ = 324.0665; **IR (ATR) υ (cm^{-1}):** 3385, 3292, 1344.

S6.2. Procedure B: [13]

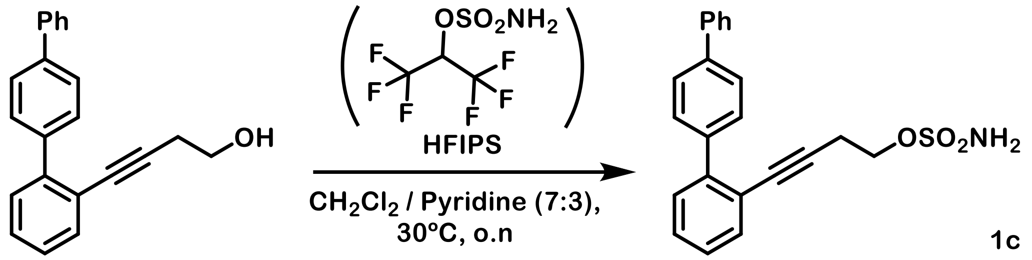

1c

HFIPS (149.4 mg, 0.60 mmol) was added to a solution of **4-(4'-phenoxy-[1,1'-biphenyl]-2-yl)but-3-yn-1-ol 8c** (150.3 mg, 0.50 mmol) in CH_2Cl_2 (3.5 mL) and pyridine (1.5 mL), and stirred at 30 °C for 16 h. Upon completion of the reaction (TLC monitoring), the crude was concentrated under reduced pressure, and co-evaporated with toluene (15 mL) to aid in pyridine removal. The crude mixture was purified by column chromatography on silica gel (Hexane/AcOEt = 80:20) to afford **4-(4'-phenyl-[1,1'-biphenyl]-2-yl)but-3-yn-1-yl sulfamate 1c** as a colourless solid (105.7 mg, 56% yield).

Formula: $C_{22}H_{19}O_3NS$; **MW:** 377.46 g/mol; **m.p.:** 101 – 103ºC; **^{1}H-NMR (400 MHz, $CDCl_3$) δ (ppm):** 7.72 – 7.65 (m, 6H), 7.55 - 7.53 (m, 1H), 7.49 - 7.45 (m, 2H), 7.44 – 7.34 (m, 3H), 7.32 - 7.28 (m, 1H), 4.42 (s, 2H), 4.21 (t, *J* = 6.6 Hz, 2H), 2.79 (t, *J* = 6.6 Hz, 2H); **^{13}C-NMR (101 MHz, $CDCl_3$) δ (ppm):** 143.4, 140.6, 140.4, 139.7, 133.4, 129.9, 129.6, 129.1, 128.7, 127.7, 127.3, 127.2 , 126.9, 121.3, 87.4, 82.5, 68.6, 20.6; **HRMS (ESI) m/z:** found: 395.1417 calculated for $[M + NH_4]^+$: 395.1424; **IR (ATR) υ (cm^{-1}):** 3366, 3272, 1346.

Following **Procedure A** and starting from **4-(4'-methyl-[1,1'-biphenyl]-2-yl)but-3-yn-1-ol 8b** (400 mg, 1.69 mmol), compound **4-(4'-methyl-[1,1'-biphenyl]-2-yl)but-3-yn-1-yl sulfamate 1b** was obtained as a colorless solid (249.8 mg, 47% yield).

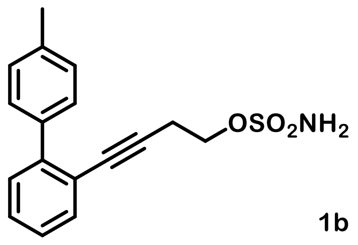

1b

Formula: $C_{17}H_{17}O_3NS$; **MW:** 315.39 g/mol; **mp:** 68 - 70ºC; **^{1}H-NMR (400 MHz, $CDCl_3$) δ (ppm):** 7.54 – 7.45 (m, 3H), 7.39 – 7.33 (m, 2H), 7.29 – 7.27 (m, 2H), 7.26-7.24 (m, 1H), 4.28 (bs, 2H), 4.21 (t, *J* = 6.4 Hz, 2H), 2.79 (t, *J* = 6.4 Hz, 2H), 2.42 (s, 3H)**; ^{13}C-NMR (101 MHz, $CDCl_3$) δ (ppm):** 143.8, 137.8, 137.6, 133.4, 129.7, 129.3, 129.0, 128.6, 127.0, 121.2, 87.3, 82.5, 68.7, 21.3, 20.6**; HRMS (ESI) m/z:** calculated for $[M + K]^+$: 354.0561, found: 354.0563; **IR (ATR) υ (cm^{-1}):** 3383, 3292, 1346.

Following **Procedure A** and starting from **4-(4'-methoxy-[1,1'-biphenyl]-2-yl)but-3-yn-1-ol 8d** (300 mg, 1.19 mmol), compound **4-(4'-methoxhy-[1,1'-biphenyl]-2-yl)but-3-yn-1-yl sulfamate 1d** was obtained as a colorless solid (135.5 mg, 35% yield).

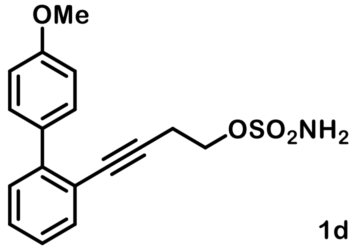

1d

Formula: $C_{17}H_{17}O_4NS$; **MW:** 331.39 g/mol; **m.p.:** 70 - 72ºC; **^{1}H-NMR (400 MHz, $CDCl_3$) δ (ppm):** 7.54 - 7.49 (m, 3H), 7.36 – 7.32 (m, 2H), 7.27 – 7.25 (m, 1H), 7.02 – 6.97 (m, 2H), 4.45 (bs, 2H), 4.20 (t, *J* = 6.5 Hz, 2H), 3.88 (s, 3H), 2.79 (t, *J* = 6.5 Hz, 2H); **^{13}C-NMR (101 MHz, $CDCl_3$) δ (ppm):** 159.2, 143.6, 133.2, 133.1, 130.6, 130.5, 129.5, 128.6, 126.8, 126.7, 121.2, 113.7, 113.5, 87.2, 82.6, 68.7, 55.5, 20.6; **HRMS (ESI) m/z:** found: 354.0768, calculated for $[M + Na]^+$ = 354.0770; **IR (ATR) υ (cm^{-1}):** 3379, 3286, 1342.

Following **Procedure B** and starting from **4-(4'-phenoxy-[1,1'-biphenyl]-2-yl)but-3-yn-1-ol 8e** (382.2 mg, 1.22 mmol), compound **4-(4'-phenoxy-[1,1'-biphenyl]-2-yl)but-3-yn-1-yl sulfamate 1e** was obtained as an orange oil (146.1 mg, 31% yield).

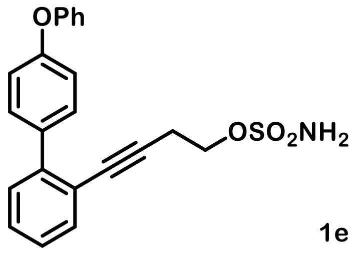

1e

Formula: $C_{22}H_{19}O_4NS$; **MW:** 393.46 g/mol; **^{1}H-NMR (400 MHz, $CDCl_3$) δ (ppm):** 7.60 – 7.48 (m, 3H), 7.41 – 7.34 (m, 4H), 7.30 – 7.23 (m, 1H), 7.18 – 7.01 (m, 5H), 4.59 (bs, 2H), 4.22 (t, *J* = 6.8 Hz, 2H), 2.79 (t, *J* = 6.8 Hz, 2H); **^{13}C-NMR (101 MHz, $CDCl_3$) δ (ppm):** 157.1, 157.0, 143.3, 135.6, 133.3, 130.8, 130.0, 129.5, 128.7, 127.1, 123.8, 121.3, 119.5, 118.1, 87.4, 82.5, 68.6, 20.6; **HRMS (ESI) m/z:** found: 411.1372, calculated for $[M + NH_4]^+$: 411.1373; **IR (ATR) ʋ (cm^{-1}):** 3379, 3279, 1362.

Following **Procedure A** and starting from **4-(4'-fluoro-[1,1'-biphenyl]-2-yl)but-3-yn-1-ol 8f** (550.0 mg, 2.29 mmol), compound **4-(4'-fluoro-[1,1'-biphenyl]-2-yl)but-3-yn-1-yl sulfamate 1f** was obtained as a colorless solid (455.3 mg, 62% yield).

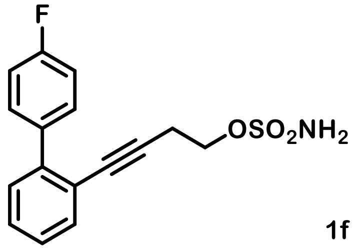

1f

Formula: $C_{16}H_{14}O_3NSF$; **MW:** 319.35 g/mol; **m.p.:** 85 - 87°C; **^{1}H-NMR (400 MHz, $CDCl_3$) δ (ppm):** 7.57 (d, *J* = 6.7 Hz, 2H), 7.53 – 7.37 (m, 4H), 7.08 (dd, *J* = 9.5, 2.8 Hz, 1H), 6.99 (td, *J* = 8.3, 2.8 Hz, 1H), 4.43 (bs, 2H), 4.19 (t, *J* = 6.6 Hz, 2H), 2.76 (t, *J* = 6.6 Hz, 2H); **^{13}C-NMR (101 MHz, $CDCl_3$) δ (ppm):** 162.5 (d, $^1J_{C-F}$ = 250.7 Hz), 146.2, 139.7, 135.1 (d, $^4J_{C-F}$ = 8.8 Hz) , 129.3, 128.3, 128.2, 117.4, 116.8 (d, $^2J_{C-F}$ = 22.4 Hz), 114.6 (d, $^3J_{C-F}$ = 21.7 Hz), 86.8, 81.5, 68.6, 20.5; **^{19}F NMR (377 MHz, $CDCl_3$) δ (ppm):** -112.03; **HRMS (ESI) m/z:** found: 337.1014 g/mol, calculated for $[M + NH_4]^+$ = 337.1017; **IR (ATR) ʋ (cm^{-1}):** 3387, 3291, 1364.

Following **Procedure B** and starting from **4-(4'-bromo-[1,1'-biphenyl]-2-yl)but-3-yn-1-ol 8g** (150.9 mg, 0.51 mmol), compound **4-(4'-bromo-[1,1'-biphenyl]-2-yl)but-3-yn-1-yl sulfamate 1g** was obtained as a colorless solid (77.1 mg, 41% yield).

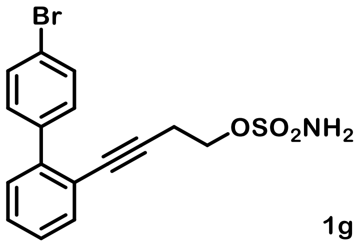

1g

Formula: $C_{16}H_{14}O_3NSBr$; **MW:** 380.26 g/mol; **m.p.:** 81 - 83°C; **^{1}H-NMR (400 MHz, $CDCl_3$) δ (ppm):** 7.61 – 7.54 (m, 2H), 7.54 – 7.49 (m, 1H), 7.48 – 7.42 (m, 2H), 7.40 – 7.35 (m, 1H), 7.34 – 7.29 (m, 2H), 4.59 (bs, 2H), 4.23 (t, *J* = 6.7 Hz, 2H), 2.79 (t, *J* = 6.7 Hz, 2H); **^{13}C-NMR (101 MHz, $CDCl_3$) δ (ppm):** 142.7, 139.6, 133.4, 131.3, 131.1, 129.5, 129.4, 128.7, 128.2, 127.6, 121.8, 121.2, 87.6, 82.2, 68.6, 20.6; **HRMS (ESI) m/z:** found: 397.0216-399.0192, calculated for $[M + NH_4]^+$ = 397.0216-399.0196; **IR (ATR) υ (cm^{-1}):** 3380, 3279, 1359.

Following **Procedure B** and starting from **4-(4'-(trifluoromethyl)-[1,1'-biphenyl]-2-yl)but-3-yn-1-ol 8h** (96.7 mg, 0.33 mmol), compound **4-(4'-(trifluoromethyl)-[1,1'-biphenyl]-2-yl)but-3-yn-1-yl sulfamate 1h** was obtained as a colorless solid (41.6 mg, 34% yield).

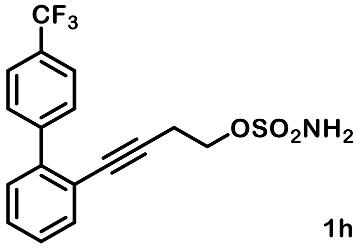

1h

Formula: $C_{17}H_{14}O_3NSF_3$; **MW:** 369.36 g/mol; **m.p.:** 75-77°C; **^{1}H-NMR (400 MHz, $CDCl_3$) δ (ppm):** 7.70 (s, 4H), 7.55 (dd, *J* = 7.8, 1.6 Hz, 1H), 7.43 – 7.30 (m, 3H), 4.62 (bs, 2H), 4.22 (t, *J* = 6.8 Hz, 2H), 2.78 (t, *J* = 6.8 Hz, 2H); **^{13}C-NMR (101 MHz, $CDCl_3$) δ (ppm):** 144.3, 142.5, 133.5, 129.8, 129.6 (q, $^2J_{C\text{-}F}$ = 32.8 Hz), 129.5, 128.8, 128.0, 125.1 (q, $^3J_{C\text{-}F}$ = 3.9 Hz), 124.4 (q, $^1J_{C\text{-}F}$ = 273.0 Hz), 121.4, 87.8, 82.0, 68.5, 20.6; **^{19}F-NMR (377 MHz, $CDCl_3$) δ:** -63.3; **HRMS (ESI) m/z:** found: 387.0976, calculated for $[M + NH_4]^+$ = 387.0985; **IR (ATR) υ (cm^{-1}):** 3366, 3290, 1319**.**

Following **Procedure B** and starting from **4-(4-methyl-[1,1'-biphenyl]-2-yl)but-3-yn-1-ol 8i** (138.5 mg, 0.59 mmol), compound **4-(4-methyl-[1,1'-biphenyl]-2-yl)but-3-yn-1-yl sulfamate 1i** was obtained as a colorless solid (109.4 mg, 59% yield).

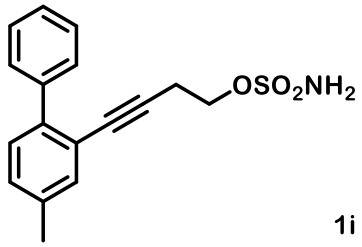

1i

Formula: $C_{17}H_{17}NO_3S$; **MW:** 315.39 g/mol; **m.p.:** 111 - 113ºC; **^{1}H-NMR (400 MHz, $CDCl_3$) δ (ppm):** 7.60 – 7.54 (m, 2H), 7.47 – 7.40 (m, 2H), 7.40 – 7.32 (m, 2H), 7.28 – 7.24 (m, 1H), 7.19 (dd, *J* = 8.0, 1.9 Hz, 1H), 4.36 (bs, 2H), 4.20 (t, *J* = 6.5 Hz, 2H), 2.77 (t, *J* = 6.5 Hz, 2H), 2.36 (s, 3H); **^{13}C-NMR (101 MHz, $CDCl_3$) δ (ppm):** 141.0, 140.7, 137.1, 133.8, 129.6, 129.6, 129.5, 128.2, 127.5, 121.0, 86.9, 82.6, 68.7, 21.0, 20.6; **HRMS (ESI) m/z:** found: 333.1269, calculated for $[M + NH_4]^+$ = 333.1267; **IR (ATR) υ (cm^{-1}):** 3378, 3286, 1341.

Following **Procedure B** and starting from **4-(5-methyl-[1,1'-biphenyl]-2-yl)but-3-yn-1-ol 8j** (271.6 mg, 1.15 mmol), compound **4-(5-methyl-[1,1'-biphenyl]-2-yl)but-3-yn-1-yl sulfamate 1j** was obtained as a colorless solid (225.5 mg, 62% yield).

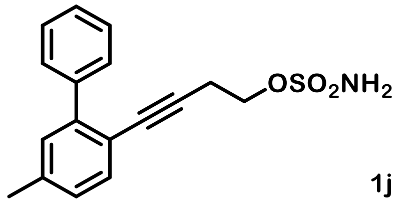

1j

Formula: $C_{17}H_{17}NO_3S$; **MW:** 315.39 g/mol; **m.p.:** 92 - 94ºC; **^{1}H-NMR (400 MHz, $CDCl_3$) δ (ppm):** 7.60 – 7.54 (m, 2H), 7.48 – 7.34 (m, 4H), 7.17 (d, *J* = 1.8 Hz, 1H), 7.10 (dd, *J* = 7.8, 1.7 Hz, 1H), 4.36 (bs, 2H), 4.19 (t, *J* = 6.5 Hz, 2H), 2.77 (t, *J* = 6.5 Hz, 2H), 2.39 (s, 3H); **^{13}C-NMR (101 MHz, $CDCl_3$) δ (ppm):** 143.7, 140.9, 138.8, 133.2, 130.5, 129.5, 128.2, 128.1, 127.6, 118.3, 86.5, 82.5, 68.8, 21.6, 20.6; **HRMS (ESI) m/z:** found: 316.1006, calculated for $[M + H]^+$ = 316.1002; **IR (ATR) υ (cm^{-1}):** 3390, 3294, 1346.

Following **Procedure B** and starting from **4-(5-fluoro-[1,1'-biphenyl]-2-yl)but-3-yn-1-ol 8k** (341.0 mg, 1.42 mmol), compound **4-(5-fluoro-[1,1'-biphenyl]-2-yl)but-3-yn-1-yl sulfamate 1k** was obtained as a colorless solid (230.2 mg, 51% yield).

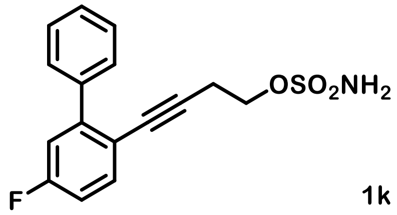

1k

Formula: $C_{16}H_{14}FNO_3S$; **MW:** 319.35 g/mol; **m.p.:** 84 - 86ºC; **^{1}H-NMR (400 MHz, $CDCl_3$) δ (ppm):** 7.61 – 7.54 (m, 2H), 7.53 – 7.37 (m, 4H), 7.08 (dd, *J* = 9.5, 2.7 Hz, 1H), 6.99 (td, *J* = 8.3, 2.7 Hz, 1H), 4.44 (bs, 2H), 4.19 (t, *J* = 6.6 Hz, 2H), 2.76 (t, *J* = 6.6 Hz, 2H); **^{13}C-NMR (101 MHz, $CDCl_3$) δ (ppm):** 162.4 (d, $^1J_{C\text{-}F}$ = 250.8 Hz), 146.1 (d, $^3J_{C\text{-}F}$ = 8.1 Hz), 139.7 (d, $^4J_{C\text{-}F}$ = 1.9 Hz), 135.1 (d, $^3J_{C\text{-}F}$ = 8.6 Hz), 129.3, 128.3, 128.2, 117.4 (d, $^4J_{C\text{-}F}$ = 3.3 Hz), 116.7 (d, $^2J_{C\text{-}F}$ = 22.4 Hz), 114.5 (d, $^2J_{C\text{-}F}$ = 21.9 Hz), 86.9, 81.5, 68.6, 20.5; **^{19}F NMR (377 MHz, $CDCl_3$) δ (ppm):** -112.0; **HRMS (ESI) m/z:** found: 337.1014, calculated for $[M + NH_4]^+$ = 337.1017; **IR (ATR) ʋ (cm^{-1}):** 3355, 3280, 1344.

Following **Procedure B** and starting from **4-(4,5-difluoro-[1,1'-biphenyl]-2-yl)but-3-yn-1-ol 8l** (63 mg, 0.24 mmol), compound **4-(4,5-difluoro-[1,1'-biphenyl]-2-yl)but-3-yn-1-yl sulfamate 1l** was obtained as a colorless solid (43.8 mg, 53% yield).

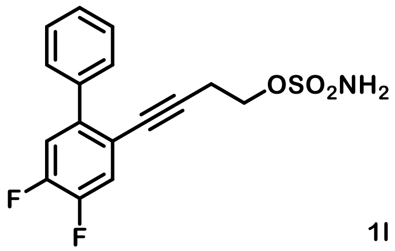

1l

Formula: $C_{16}H_{13}F_2NO_3S$; **MW:** 337.34 g/mol; **m.p.:** 88 - 90ºC; **^{1}H-NMR (400 MHz, $CDCl_3$) δ (ppm):** 7.55 – 7.50 (m, 2H), 7.49 – 7.37 (m, 3H), 7.32 (dd, *J* = 10.7, 8.0 Hz, 1H), 7.16 (dd, *J* = 11.1, 8.0 Hz, 1H), 4.42 (bs, 2H), 4.19 (t, *J* = 6.5 Hz, 2H), 2.76 (t, *J* = 6.5 Hz, 2H). **^{13}C-NMR (101 MHz, $CDCl_3$) δ (ppm):** 150.3 (dd, $^1J_{C\text{-}F}$ = 252.8, $^2J_{C\text{-}F}$ = 12.6 Hz), 149.2 (dd, $^1J_{C\text{-}F}$ = 250.4, $^2J_{C\text{-}F}$ = 13.2 Hz), 141.3 (q, $^3J_{C\text{-}F}$ = $^4J_{C\text{-}F}$ = 2.4 Hz), 138.89, 129.3, 128.4, 128.2, 121.8 (d, $^2J_{C\text{-}F}$ = 18.5 Hz), 118.6 (d, $^2J_{C\text{-}F}$ = 18.3 Hz), 117.9 (q, $^3J_{C\text{-}F}$ = $^4J_{C\text{-}F}$ = 4.0 Hz), 87.8 (d, $^5J_{C\text{-}F}$ = 2.0 Hz), 80.6 (t, $^4J_{C\text{-}F}$ = $^5J_{C\text{-}F}$ = 2.0 Hz), 68.4, 20.5; **^{19}F NMR (377 MHz, $CDCl_3$) δ (ppm):** -136.57 (d, $^3J_{F\text{-}F}$ = 22.3 Hz), -140.48 (d, $^3J_{F\text{-}F}$ = 22.3 Hz); **HRMS (ESI) m/z:** found: 360.0485, calculated for $[M + Na]^+$ = 360.0476; **IR (ATR) ʋ (cm^{-1}):** 3352, 3280, 1327.

Following **Procedure B** and starting from **4-(2-(naphthalen-1-yl)phenyl)but-3-yn-1-ol 8m** (0.99 g, 3.65 mmol), compound **4-(2-(naphthalen-1-yl)phenyl)but-3-yn-1-yl sulfamate 1m** was obtained as am orange solid (0.71 g, 56% yield).

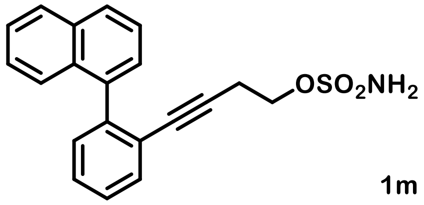

1m

Formula: $C_{20}H_{17}NO_3S$; **MW:** 351.42 g/mol; **m.p.:** 132-134 ºC; **^{1}H-NMR (400 MHz, $CDCl_3$) δ (ppm):** 7.91 (tt, *J* = 8.2, 1.0 Hz, 2H), 7.63 – 7.53 (m, 3H), 7.50 (ddd, *J* = 8.2, 6.7, 1.3 Hz, 1H), 7.45 – 7.35 (m, 5H), 4.17 (bs, 2H), 3.69 – 3.60 (m, 2H), 2.46 (t, *J* = 6.8 Hz, 2H); **^{13}C-NMR (101 MHz, $CDCl_3$) δ (ppm):** 143.1, 139.1, 133.5, 132.3, 131.9, 130.8, 128.3, 128.3, 128.0, 127.6, 127.5, 126.6, 126.0, 126.0, 125.5, 123.3, 87.8, 82.2, 68.2, 20.2; **HRMS (ESI) m/z:** found: 374.0827, calculated for $[M + Na]^+$ = 374.0821; **IR (ATR) υ (cm^{-1}):** 3382, 3297, 1349.

Following **Procedure A** and starting from **4-(4'-dimethylamino-[1,1'-biphenyl]-2-yl)but-3-yn-1-ol 8n** (501.2 mg, 1.88 mmol), compound **4-(4'-dimethylamino-[1,1'-biphenyl]-2-yl)but-3-yn-1-yl sulfamate 1n** was obtained as a colorless solid (240.7 mg, 37% yield).

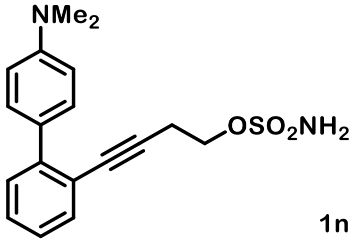

1n

Formula: $C_{18}H_{20}N_2O_3S$; **MW:** 344.43; **m.p.:** 89 - 91ºC; **^{1}H-NMR (400 MHz, $CDCl_3$) δ (ppm):** 7.55 – 7.43 (m, 3H), 7.37 – 7.30 (m, 2H), 7.24 – 7.16 (m, 1H), 6.85 (d, *J* = 8.1 Hz, 2H), 4.45 (bs, 2H), 4.17 (t, *J* = 6.3 Hz, 2H), 3.02 (s, 6H), 2.79 (t, *J* = 6.3 Hz, 2H); **^{13}C-NMR (101 MHz, $CDCl_3$) δ (ppm):** 144.0, 133.1, 130.3, 129.3, 128.6, 126.4, 121.1, 112.5, 87.3, 82.9, 68.7, 40.9 (bs, 2C), 20.7; **HRMS (ESI) m/z:** found: 345.1272, calculated for $[M + H]^+$ = 345.1267; **IR (ATR) υ (cm^{-1}):** 3366, 3303, 1345.

Following **Procedure B** and starting from **4-(4'-fluoro-4-methyl-[1,1'-biphenyl]-2-yl)but-3-yn-1-ol 8o** (270.8 mg, 1.06 mmol), compound **4-(4'-fluoro-4-methyl-[1,1'-biphenyl]-2-yl)but-3-yn-1-yl sulfamate 1o** was obtained as a colorless solid (335.0 mg, 95% yield).

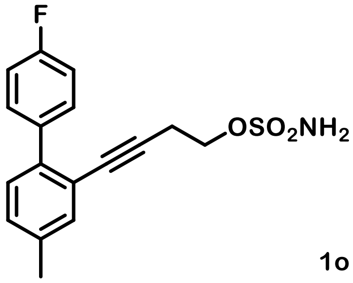

1o

Formula: $C_{17}H_{16}FNO_3S$; **MW:** 333.38; **m.p.:** 98 - 100ºC; **^{1}H-NMR (400 MHz, $CDCl_3$) δ (ppm):** 7.55 – 7.48 (m, 2H), 7.34 (d, *J* = 1.2 Hz, 1H), 7.22 (d, *J* = 7.8 Hz, 1H), 7.17 (dd, *J* = 7.8, 1.2 Hz 1H), 7.15 – 7.07 (m, 2H), 4.65 (bs, 2H), 4.22 (t,

J = 6.7 Hz, 2H), 2.77 (t, J = 6.7 Hz, 2H), 2.35 (s, 3H); **^{13}C-NMR (101 MHz, $CDCl_3$) δ (ppm):** 162.4 (d, $^{1}J_{C\text{-}F}$ = 247.0 Hz), 140.1, 137.2, 136.6 (d, $^{4}J_{C\text{-}F}$ = 3.0 Hz), 133.8, 131.0 (d, $^{3}J_{C\text{-}F}$ = 8.1 Hz), 129.6, 129.4, 121.0, 115.0 (d, $^{2}J_{C\text{-}F}$ = 21.2 Hz), 86.9, 82.5, 68.7, 21.0, 20.6; **^{19}F NMR (377 MHz, $CDCl_3$) δ (ppm):** -116.16; **IR (ATR) υ (cm^{-1}):** 3369, 3287, 1342.

S7. General procedure for single-carbon insertion via nitrene cascade reaction

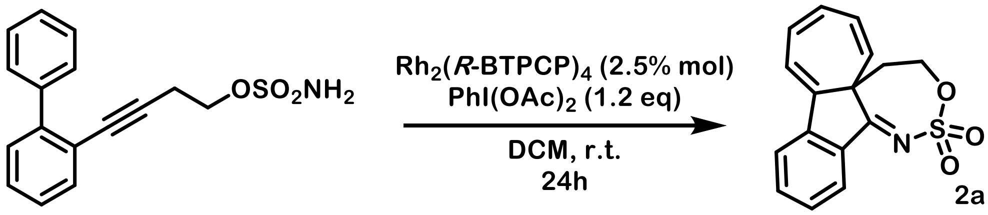

2a

The **4-([1,1'-biphenyl]-2-yl)but-3-yn-1-yl sulfamate 1a** (15.1 mg, 5 mmol, 1 eq) and the diacetoxyiodo benzene (19.3 mg, 6 mmol, 1.2 eq) were added to a vial containing the rhodium catalyst (2.2 mg, 0.125 mmol, 2.5% mol), followed by the addition of dichloromethane (1 mL). The reaction was left to stir at room temperature for 24 hours. Upon completion of the reaction (TLC monitoring), the crude was concentrated under rotatory evaporation and was purified by column chromatography on silica gel (Hexane/AcOEt = 80:20) to afford **2a** as a yellow, fluorescent solid (19.7 mg, 99% yield).

Formula: $C_{16}H_{13}NO_3S$; **MW:** 299.34; **m.p.:** 88-90 °C; **^{1}H-NMR (400 MHz, $CDCl_3$) δ (ppm):** 7.94 (d, J = 8.0 Hz, 1H), 7.68 – 7.60 (m, 2H), 7.46 – 7.42 (m, 1H), 6.88 (d, J = 6.0 Hz, 1H), 6.75 – 6.65 (m, 2H), 6.39 (dd, J = 10.0, 5.6 Hz, 1H), 5.88 (d, J = 10.0 Hz, 1H), 4.61 (ddd, J = 12.0, 11.4, 4.0 Hz, 1H), 4.31 (ddd, J = 12.0, 5.2, 2.8 Hz, 1H), 2.17 (ddd, J = 15.4, 4.0, 2.8 Hz, 1H), 1.63 (ddd, J = 15.4, 11.4, 5.2 Hz, 1H); **^{13}C-NMR (101 MHz, $CDCl_3$) δ (ppm):** 185.0, 147.0, 136.6, 136.1, 133.4, 131.9, 130.3, 129.1, 127.0, 126.5, 126.1, 122.4, 116.2, 68.3, 56.5, 24.3; **HRMS (ESI) m/z:** found: 300.0689, calculated for $[M + H]^+$: 300.0688; **IR (ATR) υ (cm^{-1}):** 1359.

According to general procedure and starting from **4-(4'-methyl-[1,1'-biphenyl]-2-yl)but-3-yn-1-yl sulfamate 1b** (15.8 mg, 0.05 mmol), compound **2b** was obtained as a yellow, fluorescent solid (15.8 mg, 99% yield).

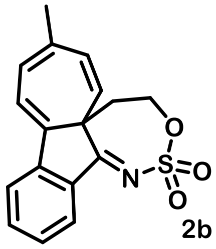

2b

Formula: $C_{17}H_{15}NO_3S$; **MW:** 313.37; **m.p.:** 160-162 °C; **^{1}H-NMR (400 MHz, $CDCl_3$) δ (ppm):** 7.92 (d, *J* = 7.8 Hz, 1H), 7.69 – 7.55 (m, 2H), 7.41 (t, *J* = 8.0 Hz, 1H), 6.77 (d, *J* = 6.7 Hz, 1H), 6.51 (d, *J* = 8.0 Hz, 1H), 6.22 (d, *J* = 10.1 Hz, 1H), 5.84 (d, *J* = 10.1 Hz, 1H), 4.61 (ddd, *J* = 12.0, 11.4, 4.0 Hz, 1H), 4.32 (ddd, *J* = 12.0, 5.2, 2.8 Hz, 1H), 2.20 (ddd, *J* = 15.0, 4.0, 2.8 Hz, 1H), 2.14 (s, 3H), 1.64 (ddd, *J* = 15.0, 11.4, 5.2 Hz, 1H); **^{13}C-NMR (101 MHz, $CDCl_3$) δ (ppm):** 185.4, 147.4, 141.8, 136.6, 135.8, 131.6, 129.8, 129.6, 126.4, 126.3, 126.0, 122.0, 116.4, 68.2, 56.2, 24.8, 24.8; **HRMS (ESI) m/z:** found: 314.0848, calculated for $[M + H]^+$: 314.0845; **IR (ATR) ʋ (cm^{-1}):** 1353.

According to general procedure and starting from **4-(4'-phenyl-[1,1'-biphenyl]-2-yl)but-3-yn-1-yl sulfamate 1c** (18.9 mg, 0.05 mmol), compound **2c** was obtained as an orange, fluorescent solid (17.8 mg, 95% yield).

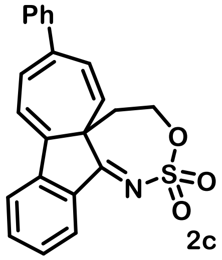

2c

Formula: $C_{22}H_{17}O_3NS$; **MW:** 375.44 g/mol; **m.p.:** 94-96 °C; **^{1}H-NMR (400 MHz, $CDCl_3$) δ (ppm):** 7.97 (d, *J* = 7.8 Hz, 1H), 7.72 – 7.63 (m, 2H), 7.56 – 7.40 (m, 5H), 7.40 – 7.33 (m, 1H), 7.10 (d, *J* = 6.9 Hz, 1H), 6.98 (d, *J* = 6.9 Hz, 1H), 6.60 (d, *J* = 10.2 Hz, 1H), 6.06 (d, *J* = 10.2 Hz, 1H), 4.69 (ddd, *J* = 12.0, 11.4, 4.0 Hz, 1H), 4.33 (ddd, *J* = 12.0, 5.2, 2.8 Hz, 1H), 2.23 (ddd, *J* = 15.4, 4.0, 2.8 Hz, 1H), 1.70 (ddd, *J* = 15.4, 11.4, 5.2 Hz, 1H); **^{13}C-NMR (101 MHz, $CDCl_3$) δ (ppm):** 185.0, 147.0, 144.3, 140.98 136.7, 136.1, 134.1, 130.3, 129.0 , 128.5, 127.9, 127.4, 126.9, 126.1, 125.9, 122.3, 116.5, 68.2, 56.2, 24.9; **HRMS (ESI) m/z:** found: 398.0818, calculated for $[M + Na]^+$: 398.0821; **IR (ATR) ʋ (cm^{-1}):1358.**

According to general procedure and starting from **4-(4'-methoxy-[1,1'-biphenyl]-2-yl)but-3-yn-1-yl sulfamate 1d** (16.5 mg, 0.05 mmol), compound **2d** was obtained as an orange, fluorescent solid (14.0 mg, 85% yield).

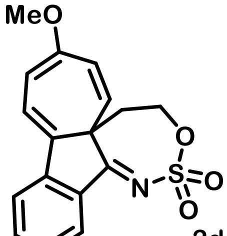

2d

Formula: $C_{17}H_{15}O_4NS$; **MW:** 329.37 g/mol; **m.p.:** 92-95 ºC; **^{1}H-NMR (400 MHz, $CDCl_3$) δ (ppm):** 7.91 (d, *J* = 8.0 Hz, 1H), 7.63 (t, *J* = 8.0 Hz, 1H), 7.56 (d, *J* = 8.0 Hz, 1H), 7.37 (t, *J* = 8.0 Hz, 1H), 6.83 (d, *J* = 7.6 Hz, 1H), 6.24 (dd, *J* = 10.8, 2.2 Hz, 1H), 6.05 (d, *J* = 10.8 Hz, 1H), 5.92 (dd, *J* = 7.6, 2.2 Hz, 1H), 4.65 (ddd, *J* = 12.2, 11.6, 4.0 Hz, 1H), 4.35 (ddd, *J* = 12.2, 5.4, 2.8 Hz, 1H), 3.74 (s, 3H), 2.32 (ddd, *J* = 15.0, 4.0, 2.8 Hz, 1H), 1.63 (ddd, *J* = 15.0, 11.6, 5.4 Hz, 1H); **^{13}C-NMR (101 MHz, $CDCl_3$) δ (ppm):** 185.5, 161.2, 147.8, 136.7, 135.3, 129.2, 129.0, 128.6, 126.0, 124.2, 121.6, 115.7, 102.3, 68.0, 56.0, 55.3, 25.0; **HRMS (ESI) m/z:** found: 330.0793, calculated for $[M + H]^+$: 330.0795; **IR (ATR) ʋ (cm^{-1}):** 1357**.**

According to general procedure and starting from **4-(4'-phenoxy-[1,1'-biphenyl]-2-yl)but-3-yn-1-yl sulfamate 1e** (19.1 mg, 0.05 mmol), compound **2e** was obtained as an orange, fluorescent solid (16.9 mg, 89% yield).

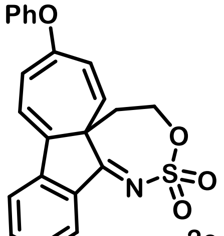

2e

Formula: $C_{22}H_{17}O_4NS$; **MW:** 391.44 g/mol; **m.p:** 158-160 ºC**; ^{1}H-NMR (400 MHz, $CDCl_3$) δ (ppm):** 7.96 (d, *J* = 7.9 Hz, 1H), 7.68 (t, *J* = 8.4 Hz, 1H), 7.60 (d, *J* = 7.9 Hz, 1H), 7.43 – 7.35 (m, 3H), 7.21 – 7.16 (m, 1H), 7.05 – 6.98 (m, 2H), 6.84 (d, *J* = 7.6 Hz, 1H), 6.37 (dd, *J* = 10.5, 1.8 Hz, 1H), 6.26 (dd, *J* = 7.6, 1.8 Hz, 1H), 6.10 (d, *J* = 10.5 Hz, 1H), 4.75 (ddd, *J* = 12.4, 11.6, 3.6 Hz, 1H), 4.44 (ddd, *J* = 12.4, 5.2, 3.0 Hz, 1H), 2.43 (ddd, *J* = 14.8, 3.6, 3.0 Hz, 1H), 1.74 (ddd, *J* = 14.8, 11.6, 5.2 Hz, 1H); **^{13}C-NMR (101 MHz, $CDCl_3$) δ (ppm):** 185.1, 159.0, 155.8, 147.3, 136.8, 135.6, 130.2, 129.8, 129.8, 129.0, 126.1, 124.7, 124.3, 122.0, 119.9, 115.1, 112.1, 68.1, 56.3, 24.9; **HRMS (ESI) m/z:** found: 392.0952, calculated for $[M + H]^+$: 392.0951; **IR (ATR) ʋ (cm^{-1}):** 1357**.**

According to general procedure and starting from **4-(4'-fluoro-[1,1'-biphenyl]-2-yl)but-3-yn-1-yl sulfamate 1f** (16.0 mg, 0.05 mmol), compound **2f** was obtained as a yellow/green, fluorescent solid (9.9 mg, 62% yield).

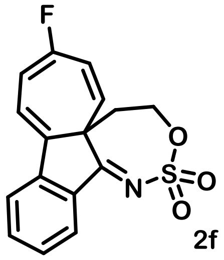

2f

Formula: $C_{16}H_{12}O_3NSF$; **MW:** 317.33 g/mol; **m.p.:** 158-160 °C; **^{1}H-NMR (400 MHz, CDCl$_3$) δ (ppm):** 7.95 (d, *J* = 8.0 Hz 1H), 7.70 – 7.65 (m, 1H), 7.61 (d, *J* = 8.0 Hz, 1H), 7.45 (t, *J* = 8.0 Hz, 1H), 6.89 – 6.78 (m, 1H), 6.48 (ddd, *J* = 15.8, 7.6, 2.0 Hz, 1H), 6.39 (ddd, *J* = 10.3, 8.1, 2.0 Hz, 1H), 6.08 (dd, *J* = 10.6, 5.2 Hz, 1H), 4.65 (ddd, J = 12.0, 11.2, 4.0 Hz 1H), 4.37 (ddd, *J* = 12.0, 5.2, 2.8 Hz, 1H), 2.26 (ddd, *J* = 15.2, 4.0, 2.8 Hz, 1H), 1.69 (ddd, *J* = 15.2, 11.2, 5.2 Hz, 1H); **^{13}C-NMR (101 MHz, CDCl$_3$) δ (ppm):** 184.3, 162.4 (d, $^1J_{C\text{-}F}$ = 251.1 Hz), 146.8, 136.9, 135.8, 130.8, 130.3, 129.8 (d, $^3J_{C\text{-}F}$ = 13.2 Hz), 126.2, 122.2, 120.9 (d, $^2J_{C\text{-}F}$ = 35.4 Hz), 113.7 (d, $^3J_{C\text{-}F}$ = 11.4 Hz), 110.6 (d, $^2J_{C\text{-}F}$ = 28.0 Hz), 67.9, 56.2, 25.0; **^{19}F NMR (377 MHz, CDCl$_3$) δ (ppm):** -95.9; **HRMS (ESI) m/z:** found: 318.0600, calculated for $[M + H]^+$: 318.0595; **IR (ATR) υ (cm^{-1}):** 1356**.**

According to general procedure and starting from **4-(4'-bromo-[1,1'-biphenyl]-2-yl)but-3-yn-1-yl sulfamate 1g** (18.7 mg, 0.05 mmol), compound **2g** was obtained as a yellow/green, fluorescent solid (10.0 mg, 54% yield).

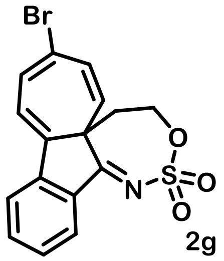

2g

Formula: $C_{16}H_{12}O_3NSBr$; **MW:** 378.24 g/mol; **m.p.:** 158-160 °C; **^{1}H-NMR (400 MHz, CDCl$_3$) δ (ppm):** 7.98 (dt, *J* = 7.9, 1.0 Hz, 1H), 7.74 – 7.68 (m, 1H), 7.64 (d, *J* = 7.2 Hz, 1H), 7.51 (m, 1H), 7.16 (d, *J* = 6.2 Hz, 1H), 6.75 (d, *J* = 7.0 Hz, 1H), 6.54 (d, *J* = 10.3, Hz, 1H), 5.83 (d, *J* = 10.3 Hz, 1H), 4.65 (ddd, *J* = 12.0, 11.4, 4.0 Hz, 1H), 4.41 (ddd, *J* = 12.0, 5.4, 2.6 Hz, 1H), 2.24 (ddd, *J* = 15.0, 4.0, 2.6 Hz, 1H), 1.74 (ddd, *J* = 15.0, 11.4, 5.4 Hz, 1H); **^{13}C-NMR (101 MHz, CDCl$_3$) δ (ppm):** 183.7, 146.4, 136.8, 136.1, 134.4, 131.4, 130.8, 130.2, 128.1, 126.2, 125.4, 122.4, 115.5, 68.0, 56.1, 25.1; **HRMS (ESI) m/z:** found: 415.9362, calculated for $[M + K]^+$: 415.9353; **IR (ATR) υ (cm^{-1}):** 1358.

According to general procedure and starting from **4-(4'-(trifluoromethyl)-[1,1'-biphenyl]-2-yl)but-3-yn-1-yl sulfamate 1h** (18.4 mg, 0.05 mmol), compound **2h** was obtained as a yellow/green, fluorescent solid (4.7 mg, 26% yield).

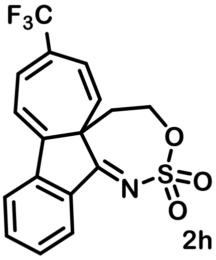

2h

Formula: $C_{17}H_{12}O_3NSF_3$; **MW:** 367.34 g/mol; **m.p.:** 113-115 ºC; **^{1}H-NMR (400 MHz, $CDCl_3$) δ (ppm):** 8.00 (d, J = 7.8 Hz, 1H), 7.75 – 7.67 (m, 2H), 7.55 (m, 1H), 7.25 – 7.20 (m, 1H), 6.95 (d, J = 6.6 Hz, 1H), 6.54 (d, J = 9.9 Hz, 1H), 6.05 (d, J = 10.0 Hz, 1H), 4.53 (ddd, J = 12.0, 11.6, 4.2 Hz, 1H), 4.35 (ddd, J = 12.0, 5.4, 2.4 Hz, 1H), 1.99 (ddd, J = 14.6, 4.2, 2.4 Hz, 1H), 1.76 (ddd, J = 14.6, 11.6, 5.4 Hz, 1H); **^{13}C-NMR (101 MHz, $CDCl_3$) δ (ppm):** 183.0, 156.7 (q, $^1J_{C-F}$ = 289.4 Hz), 145.6, 138.1, 136.9, 136.7, 132.6 (q, $^2J_{C-F}$ = 30.2 Hz), 131.6, 129.2, 128.4 (q, $^3J_{C-F}$ = 5.5 Hz), 126.3, 122.9, 121.7 (q, $^3J_{C-F}$ = 2.6 Hz), 114.2, 67.6, 55.9, 25.3; **^{19}F NMR (377 MHz, $CDCl_3$) δ (ppm):** 66.4; **HRMS (ESI) m/z:** found: 368.0567, calculated for $[M + H]^+$: 368.0563; **IR (ATR) υ (cm^{-1}):** 1363.

According to general procedure and starting from **4-(4-methyl-[1,1'-biphenyl]-2-yl)but-3-yn-1-yl sulfamate 1i** (15.8 mg, 0.05 mmol), compound **2i** was obtained as a yellow, fluorescent solid (6.3 mg, 40% yield).

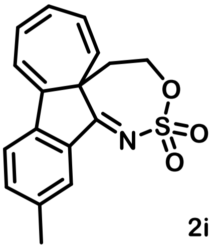

2i

Formula: $C_{17}H_{15}O_3NS$; **MW:** 313.37 g/mol; **m.p.:** 139-141ºC; **^{1}H-NMR (400 MHz, $CDCl_3$) δ (ppm):** 7.76 (s, 1H), 7.56 – 7.43 (m, 2H), 6.82 (d, J = 6.2 Hz, 1H), 6.75 – 6.60 (m, 2H), 6.37 (dd, J = 10.0, 6.2 Hz, 1H), 5.87 (d, J = 10.0 Hz, 1H), 4.61 (ddd, J = 12.0, 11.6, 4.0 Hz, 1H), 4.30 (ddd, J = 12.0, 5.2, 2.8 Hz, 1H), 2.43 (s, 3H), 2.17 (ddd, J = 15.0, 4.0, 2.8 Hz, 1H), 1.61 (ddd, J = 15.0, 11.6, 5.2 Hz, 1H); **^{13}C-NMR (101 MHz, $CDCl_3$) δ (ppm):** 185.1, 144.8, 141.0, 138.1, 136.4, 133.6, 131.5, 129.1, 127.2, 126.5, 125.8, 122.2, 115.4, 68.3, 56.9, 24.4, 21.6; **HRMS (ESI) m/z:** found: 314.0842, calculated for $[M + H]^+$: 314.0845; **IR (ATR) υ (cm^{-1}):** 1351.

According to general procedure and starting from **4-(5-methyl-[1,1'-biphenyl]-2-yl)but-3-yn-1-yl sulfamate 1j** (15.8 mg, 0.05 mmol), compound **2j** was obtained as a yellow, fluorescent solid (8.0 mg, 51% yield).

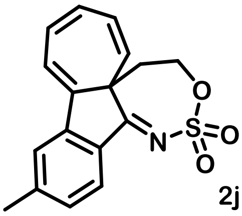

2j

Formula: $C_{17}H_{15}O_3NS$; **MW:** 313.37 g/mol; **m.p.:** 89-91ºC; **^{1}H-NMR (400 MHz, $CDCl_3$) δ (ppm):** 7.83 (d, *J* = 8.0 Hz, 1H), 7.41 (s, 1H), 7.28 (bs, 1H), 6.85 (d, *J* = 6.0 Hz, 1H), 6.75 – 6.63 (m, 2H), 6.37 (dd, *J* = 9.9, 5.9 Hz, 1H), 5.88 (d, *J* = 9.9 Hz, 1H), 4.60 (ddd, *J* = 12.0, 11.6, 3.6 Hz, 1H), 4.29 (ddd, *J* = 12.0, 5.2, 2.6 Hz, 1H), 2.47 (s, 3H), 2.15 (ddd, *J* = 14.8, 3.6, 2.6 Hz, 1H), 1.60 (ddd, *J* = 14.8, 11.6, 5.2 Hz, 1H); **^{13}C-NMR (101 MHz, $CDCl_3$) δ (ppm):** 148.4, 147.4, 133.9, 133.4, 131.9, 131.8, 129.0, 127.3, 127.0, 126.4, 125.9, 122.5, 115.9, 68.2, 56.8, 24.4, 22.5; **HRMS (ESI) m/z:** found: 314.0840, calculated for $[M + H]^+$: 314.0845; **IR (ATR) ʋ (cm^{-1}):** 1355.

According to general procedure and starting from **4-(5-fluoro-[1,1'-biphenyl]-2-yl)but-3-yn-1-yl sulfamate 1k** (16.2 mg, 0.05 mmol), compound **2k** was obtained as a yellow, fluorescent solid (16.1 mg, 99% yield).

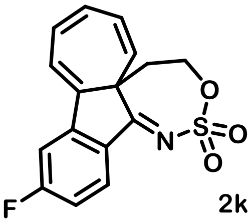

2k

Formula: $C_{16}H_{12}FNO_3S$; **MW:** 317.33 g/mol; **m.p.:** 167-170 ºC; **^{1}H-NMR (400 MHz, $CDCl_3$) δ (ppm):** 7.95 (dd, *J* = 8.7, 5.1 Hz, 1H), 7.24 (dd, *J* = 8.5, 2.1 Hz, 1H), 7.15 (td, *J* = 8.6, 2.3 Hz, 1H), 6.84 (t, *J* = 3.5 Hz, 1H), 6.77 – 6.68 (m, 2H), 6.40 (dt, *J* = 10.1, 3.3 Hz, 1H), 5.90 (d, *J* = 10.0 Hz, 1H), 4.60 (ddd, *J* = 12.0, 11.2 3.8 Hz, 1H), 4.31 (ddd, *J* = 12.0, 5.2, 2.6 Hz, 1H), 2.15 (ddd, *J* = 15.0, 3.8, 2.6 Hz, 1H), 1.63 (ddd, *J* = 15.0, 11.2, 5.2 Hz, 1H); **^{13}C-NMR (101 MHz, $CDCl_3$) δ (ppm):** 183.4, 136.6 (d, $^1J_{C\text{-}F}$ = 254.0 Hz), 132.6, 132.3, 132.1, 128.9, 128.7, 128.6, 127.2, 126.6, 119.0 ($^2J_{C\text{-}F}$ = 24.6 Hz), 117.3, 108.7 (d, $^2J_{C\text{-}F}$ = 23.5 Hz), 68.2, 56.9, 24.4; **^{19}F NMR (377 MHz, $CDCl_3$) δ (ppm):** -100.26; **HRMS (ESI) m/z:** found: 340.0414, calculated for $[M + Na]^+$: 340.0414; **IR (ATR) ʋ (cm^{-1}):** 1358.

According to general procedure and starting from **4-(4,5-difluoro-[1,1'-biphenyl]-2-yl)but-3-yn-1-yl sulfamate 1l** (16.9 mg, 0.05 mmol), compound **2l** was obtained as a yellow, fluorescent solid (12.6 mg, 75% yield).

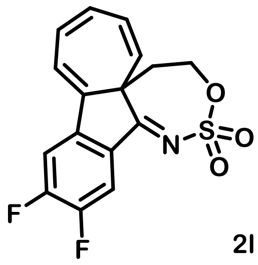

2l

Formula: $C_{16}H_{11}F_2NO_3S$; **MW:** 335.32 g/mol; **m.p.:** 155-158 °C; **^{1}H-NMR (400 MHz, $CDCl_3$) δ (ppm):** 7.71 (dd, *J* = 8.8, 7.4 Hz, 1H), 7.36 (dd, *J* = 9.3, 6.7 Hz, 1H), 6.79 – 6.69 (m, 3H), 6.43 – 6.36 (m, 1H), 5.87 (d, *J* = 9.9 Hz, 1H), 4.61 (ddd, *J* = 12.0, 11.6, 3.6 Hz, 1H), 4.32 (ddd, *J* = 12.0, 5.0, 2.6 Hz, 1H), 2.16 (ddd, *J* = 14.8, 3.6, 2.6 Hz, 1H), 1.62 (ddd, *J* = 14.8, 11.6, 5.0 Hz, 1H); **^{13}C-NMR (101 MHz, $CDCl_3$) δ (ppm):** 182.9, 157.0 (dd, $^1J_{C-F}$ = 263.6 Hz, $^2J_{C-F}$ = 15.2 Hz), 152.5 (dd, $^1J_{C-F}$ = 255.2, $^2J_{C-F}$ = 14.7 Hz), 144.3 (dd, $^4J_{C-F}$ = 2.6, $^3J_{C-F}$ = 8.0 Hz), 132.5, 131.5, 128.8, 126.9, 126.8, 117.0, 113.8 (dd, $^3J_{C-F}$ = 2.5, $^2J_{C-F}$ = 18.8 Hz), 110.6 (d, $^2J_{C-F}$ = 19.2 Hz), 68.3, 56.6, 24.3; **^{19}F NMR (377 MHz, $CDCl_3$) δ** -122.65 (d, *J* = 19.2 Hz), -131.93 (d, *J* = 19.2 Hz); **HRMS (ESI) m/z:** found: 374.0058, calculated for $[M + K]^+$: 374.0059; **IR (ATR) υ (cm^{-1}):** 1334.

According to general procedure and starting from **4-(4'-fluoro-4-methyl-[1,1'-biphenyl]-2-yl)but-3-yn-1-yl sulfamate 1o** (16.7 mg, 0.05 mmol), compound **2o** was obtained as a yellow, fluorescent solid (5.6 mg, 35% yield).

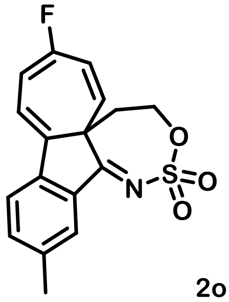

2o

Formula: $C_{17}H_{14}FNO_3S$; **MW:** 331.36 g/mol; **m.p.:** 100 - 102 °C; **^{1}H-NMR (400 MHz, $CDCl_3$) δ (ppm):** 7.76 (s, 1H), 7.53 – 7.45 (m, 2H), 6.77 (t, *J* = 7.4 Hz, 1H), 6.46 (ddd, *J* = 15.9, 7.4, 2.0 Hz, 1H), 6.37 (ddd, *J* = 10.4, 7.4, 2.0 Hz, 1H), 6.06 (dd, *J* = 10.4, 5.4 Hz, 1H), 4.65 (td, *J* = 12.0, 3.8 Hz, 1H), 4.36 (ddd, *J* = 12.0, 5.4, 3.2 Hz, 1H), 2.43 (s, 3H), 2.25 (dt, *J* = 15.2, 3.2 Hz, 1H), 1.72 – 1.65 (m, 1H).; **^{13}C-NMR (101 MHz, $CDCl_3$) δ (ppm):** 184.5, 162.2 (d, $^1J_{C-F}$ = 250.5 Hz), 144.6, 141.1, 138.4, 136.0, 130.9 (d, $^4J_{C-F}$ = 3.9 Hz), 129.8 (d, $^3J_{C-F}$ = 13.1 Hz), 125.9, 122.0, 120.8 (d, $^2J_{C-F}$ = 35.4 Hz),112.9 (d, $^3J_{C-F}$ = 10.1 Hz), 110.6 (d, $^2J_{C-F}$ = 28.3 Hz), 68.0, 56.5, 25.0, 21.6; **^{19}F NMR (377 MHz, $CDCl_3$) δ (ppm):** -96.63; **HRMS**

(ESI) m/z: found: 332.0753, calculated for $[M + H]^+$: 332.0751; **IR (ATR) ʋ (cm^{-1}):** 1353.

S8. Reduction of the sulfamate 2a.

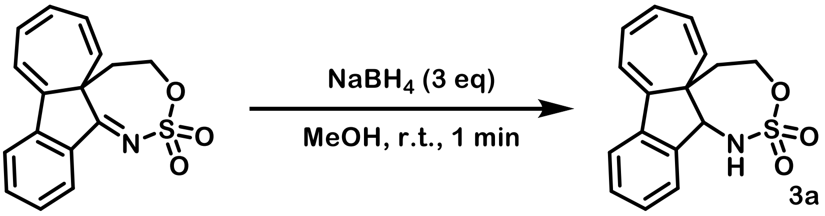

Sodium borohydride (4.5 mg, 0.12 mmol, 3 eq) was added to a vial containing **2a** (12.0 mg, 0.04 mmol) in methanol (1 mL). The reaction was stirred at room temperature for 1 minute. Upon completion of the reaction (TLC monitoring and color change of the crude from bright yellow to colorless), the crude was concentrated and was purified by column chromatography on silica gel (Hexane/AcOEt = 85:15) to afford **3a** as a colorless solid (9.8 mg, 82% yield).

Formula: $C_{16}H_{15}NO_3S$; **MW:** 301.36; **m.p.:** 165-167 °C; **^{1}H-NMR (400 MHz, $CDCl_3$) δ (ppm):** 7.53 – 7.48 (m, 1H), 7.48 – 7.43 (m, 1H), 7.40 – 7.33 (m, 2H), 6.83 (d, *J* = 6.7 Hz, 1H), 6.60 (dd, *J* = 11.0, 6.7 Hz, 1H), 6.51 (dd, *J* = 10.9, 6.4 Hz, 1H), 6.37 (dd, *J* = 10.2, 6.4 Hz, 1H), 5.89 (d, *J* = 10.2 Hz, 1H), 5.13 (d, *J* = 8.8 Hz, 1H), 4.87 (d, *J* = 8.9 Hz, 1H), 4.45 (ddd, *J* = 12.6, 10.8, 1.6 Hz, 1H), 3.94 (ddd, *J* = 12.6, 5.2, 2.0 Hz, 1H), 1.97 (ddd, *J* = 15.8, 5.2, 1.6 Hz, 1H), 1.67 (ddd, *J* = 15.8, 10.8, 2.0 Hz, 1H); **^{13}C-NMR (101 MHz, $CDCl_3$) δ (ppm):** 140.8, 140.5, 139.0, 130.2, 130.1, 130.0, 128.6, 128.5, 126.6, 125.4, 121.9, 115.4, 69.9, 66.3, 57.4, 26.4; **HRMS (ESI) m/z:** found: 319.1107, calculated for $[M + NH_4]^+$: 319.1111; **IR (ATR) ʋ (cm^{-1}):** 1359.

Analysis of the 1H-NMR spectrum of the product obtained from the imine reduction (**3a**) revealed the presence of a single diastereoisomer, indicating that the reduction proceeds in a diastereoselective manner.

To determine the relative stereochemistry of **3a**, a “Nuclear Overhauser Effect SpectroscopY” (NOESY) experiment was performed. The NOESY spectrum shows a clear correlation between the proton attached to the newly formed quaternary carbon (δ = 5.13 ppm) and the proton on the right side of the cycloheptatriene moiety (δ = 5.89 ppm). This interaction strongly suggests that the diastereoisomer formed during the reduction possesses both protons oriented in the same spatial direction.

S9. Mechanistic study.

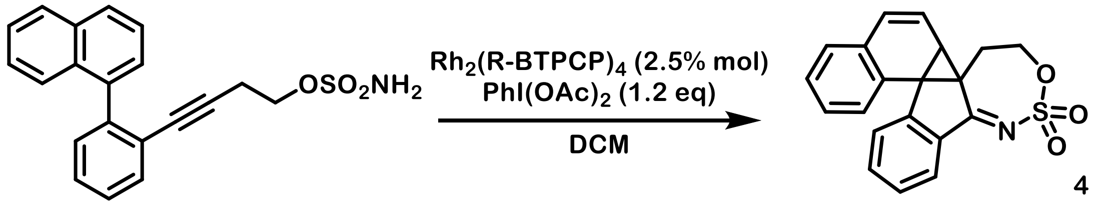

A mixture of **4-(2-(naphthalen-1-yl)phenyl)but-3-yn-1-yl sulfamate 1m** (17.5 mg, 5 mmol, 1 eq) and diacetoxyiodo benzene (19.3 mg, 6 mmol, 1.2 eq) was added to a vial containing the rhodium catalyst (2.2 mg, 0.125 mmol, 2.5% mol), followed by the addition of dichloromethane (1 mL). The reaction was stirred at room temperature for 24 hours until completion (TLC monitoring). The crude was then concentrated and was purified by column chromatography on silica gel (Hexane/AcOEt = 85:15) to afford **4** as a yellow, fluorescent solid (7.3 mg, 42% yield).

Formula: $C_{20}H_{15}NO_3S$; **MW:** 349.4; **m.p.:** 141-143 °C; **^{1}H-NMR (400 MHz, $CDCl_3$) δ (ppm):** 7.95 (d, *J* = 7.7 Hz, 1H), 7.65 – 7.58 (m, 2H), 7.49 – 7.31 (m, 5H), 6.74 (d, *J* = 9.7 Hz, 1H), 6.21 (dd, *J* = 9.7, 5.1 Hz, 1H), 4.41 (ddd, *J* = 11.0, 6.3, 1.2 Hz, 1H), 4.25 (ddd, *J* = 12.3, 11.0, 4.5 Hz, 1H), 3.05 (d, *J* = 5.1 Hz, 1H), 2.36 (ddd, *J* = 15.6, 12.3, 6.3 Hz, 1H), 1.33 – 1.29 (m, 1H); **^{13}C-NMR (101 MHz, $CDCl_3$) δ (ppm):** 185.4, 152.6, 135.0, 134.9, 132.3, 131.1, 129.0, 128.8, 128.5, 128.4, 128.0, 127.6, 126.6, 124.9, 122.3, 69.3, 56.8, 46.9, 30.7, 22.2; **HRMS (ESI) m/z:** found: 372.0675, calculated for $[M + Na]^+$: 372.0665; **IR (ATR) υ (cm^{-1}):** 1358.

S10. Crystal structure of compund 2a with probability level of 50%

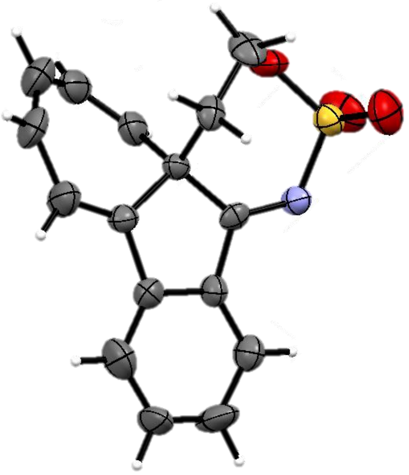

Yellow needle-like crystals of 2a were grown at 2-6 °C upon slow diffusion of pentane into a solution of 2a in chloroform.

A yellow, needle-like specimen of $C_{16}H_{13}NO_3S$, approximate dimensions 0.090 mm x 0.100 mm x 0.200 mm, was used for the X-ray crystallographic analysis. The X-ray intensity data were measured on a D8 QUEST ECO three-circle diffractometer system equipped with a Ceramic x-ray tube (Mo Kα, λ = 0.71073 Å) and a doubly curved silicon crystal Bruker Triumph monochromator. A total of 541 frames were collected. The total exposure time was 2.06 hours. The frames were integrated with the Bruker SAINT software package using a narrow-frame algorithm. The integration of the data using an orthorhombic unit cell yielded a total of 35368 reflections to a maximum θ angle of 24.00° (0.87 Å resolution), of which 4352 were independent (average redundancy 8.127, completeness = 99.8%, Rint = 13.18%, Rsig = 6.71%) and 3387 (77.83%) were greater than 2σ(F2). The final cell constants of a = 22.956(3) Å, b = 6.6292(8) Å, c = 18.175(2) Å, volume = 2765.9(6) Å3, are based upon the refinement of the XYZ-centroids of 8373 reflections above 20 σ(I) with 5.715° < 2θ < 55.93°. Data were corrected for absorption effects using the Multi-Scan method (SADABS). The ratio of minimum to maximum apparent transmission was 0.834. The calculated minimum and maximum transmission coefficients (based on crystal size) are 0.9530 and 0.9780.

The structure was solved and refined using the Bruker SHELXTL Software Package, using the space group P n a 21, with Z = 8 for the formula unit, C16H13NO3S. The final anisotropic full-matrix least-squares refinement on F2 with 379 variables converged at R1 = 7.26%, for the observed data and wR2 = 15.45% for all data. The goodness-of-fit was 1.144. The largest peak in the final difference electron density synthesis was 0.393 e-/Å3 and the largest hole was -0.248 e-/Å3 with an RMS deviation of 0.066 e-/Å3. On the basis of the final model, the calculated density was 1.438 g/cm3 and F(000), 1248 e-.

Sample and crystal data for 2a.

**Identification code**	ADII61
**Chemical formula**	$C_{16}H_{13}NO_3S$
**Formula weight**	299.33 g/mol
**Temperature**	273(2) K
**Wavelength**	0.71073 Å
**Crystal size**	0.090 x 0.100 x 0.200 mm
**Crystal habit**	yellow needle

**Crystal system**	orthorhombic
**Space group**	P n a 21
**Unit cell dimensions**	a = 22.956(3) Å α = 90°
	b = 6.6292(8) Å β = 90°
	c = 18.175(2) Å γ = 90°
**Volume**	2765.9(6) $Å^3$
**Z**	8
**Density (calculated)**	1.438 g/cm^3
**Absorption coefficient**	0.243 mm^{-1}
**F(000)**	1248

Data collection and structure refinement for 2a.

**Diffractometer**	D8 QUEST ECO three-circle diffractometer
**Radiation source**	Ceramic x-ray tube (Mo Kα, λ = 0.71073 Å)
**Theta range for data collection**	3.20 to 24.00°
**Index ranges**	-26<=h<=26, -7<=k<=7, -20<=l<=20
**Reflections collected**	35368
**Independent reflections**	4352 [R(int) = 0.1318]
**Coverage of independent reflections**	99.8%
**Absorption correction**	Multi-Scan
**Max. and min. transmission**	0.9780 and 0.9530
**Structure solution technique**	direct methods
**Structure solution program**	XT, VERSION 2018/2
**Refinement method**	Full-matrix least-squares on F^2
**Refinement program**	SHELXL-2019/1 (Sheldrick, 2019)
**Function minimized**	$\Sigma w(F_o^2 - F_c^2)^2$
**Data / restraints / parameters**	4352 / 1 / 379

**Goodness-of-fit on F^2**	1.144	
**Final R indices**	3387 data; I>2σ(I)	R1 = 0.0726, wR2 = 0.1435
	all data	R1 = 0.0981, wR2 = 0.1545
**Weighting scheme**	$w=1/[\sigma^2(F_o^2)+(0.0598P)^2+2.2171P]$ where $P=(F_o^2+2F_c^2)/3$	
**Absolute structure parameter**	-0.08(8)	
**Largest diff. peak and hole**	0.393 and -0.248 eÅ^{-3}	
**R.M.S. deviation from mean**	0.066 eÅ^{-3}	

Atomic coordinates and equivalent isotropic atomic displacement parameters (Å2) for XX.

U(eq) is defined as one third of the trace of the orthogonalized U_{ij} tensor

	**x/a**	**y/b**	**z/c**	**U(eq)**
**S1**	0.27871(11)	0.6630(4)	0.58872(13)	0.0379(6)
**S1A**	0.46636(11)	0.2692(4)	0.41348(13)	0.0351(6)
**O2**	0.3290(3)	0.5876(12)	0.6256(4)	0.064(2)
**O3**	0.2472(4)	0.8261(11)	0.6178(4)	0.061(2)
**O2A**	0.4974(3)	0.1036(11)	0.3834(4)	0.059(2)
**O4**	0.2324(3)	0.4936(10)	0.5813(4)	0.0479(18)
**O3A**	0.4158(3)	0.3355(12)	0.3780(4)	0.062(2)
**O4A**	0.5127(3)	0.4423(9)	0.4183(3)	0.0397(16)
**N21**	0.3003(3)	0.7297(11)	0.5073(4)	0.032(2)
**N21A**	0.4471(4)	0.2046(12)	0.4976(5)	0.037(2)
**C5**	0.2526(5)	0.2871(14)	0.5621(6)	0.048(3)
**C6**	0.2764(4)	0.2737(13)	0.4854(6)	0.035(2)
**C5A**	0.4927(5)	0.6491(14)	0.4377(6)	0.047(3)
**C7**	0.2548(4)	0.4421(13)	0.4333(4)	0.028(2)
**C6A**	0.4683(4)	0.6612(14)	0.5143(6)	0.040(3)

	x/a	y/b	z/c	U(eq)
**C8**	0.1894(4)	0.4768(16)	0.4389(5)	0.044(3)
**C7A**	0.4930(4)	0.4968(14)	0.5675(5)	0.031(2)
**C9**	0.1501(5)	0.3316(18)	0.4228(6)	0.052(3)
**C8A**	0.5580(4)	0.4681(16)	0.5598(5)	0.044(3)
**C10**	0.1593(5)	0.141(2)	0.3916(7)	0.067(4)
**C9A**	0.5965(5)	0.6131(17)	0.5741(7)	0.055(3)
**C11**	0.2023(5)	0.0956(19)	0.3432(7)	0.061(3)
**C10A**	0.5854(5)	0.8080(19)	0.6035(8)	0.059(4)
**C12**	0.2454(5)	0.2347(16)	0.3169(7)	0.053(3)
**C11A**	0.5432(6)	0.8490(18)	0.6525(7)	0.064(4)
**C13**	0.2655(4)	0.3969(14)	0.3530(5)	0.033(2)
**C12A**	0.5027(5)	0.7084(17)	0.6849(7)	0.049(3)
**C14**	0.3017(4)	0.5620(15)	0.3225(5)	0.037(2)
**C13A**	0.4832(4)	0.5429(14)	0.6479(5)	0.039(2)
**C15**	0.3224(6)	0.592(2)	0.2525(7)	0.068(4)
**C14A**	0.4482(4)	0.3779(16)	0.6794(6)	0.042(3)
**C16**	0.3566(6)	0.7557(19)	0.2394(7)	0.064(4)
**C15A**	0.4293(5)	0.3507(19)	0.7520(6)	0.055(3)
**C17**	0.3681(5)	0.8932(18)	0.2929(6)	0.060(3)
**C16A**	0.3975(7)	0.181(2)	0.7672(8)	0.075(4)
**C18**	0.3483(5)	0.8670(15)	0.3640(6)	0.044(3)
**C17A**	0.3816(6)	0.0428(19)	0.7128(7)	0.064(4)
**C19**	0.3149(4)	0.7002(14)	0.3781(6)	0.035(2)
**C18A**	0.4003(5)	0.0739(16)	0.6429(7)	0.055(3)
**C20**	0.2899(4)	0.6339(14)	0.4465(5)	0.029(2)
**C19A**	0.4338(4)	0.2387(14)	0.6262(6)	0.035(3)
**C20A**	0.4583(4)	0.3016(14)	0.5545(5)	0.031(2)

S11. Crystal structure of compound 4 with probability level of 50%

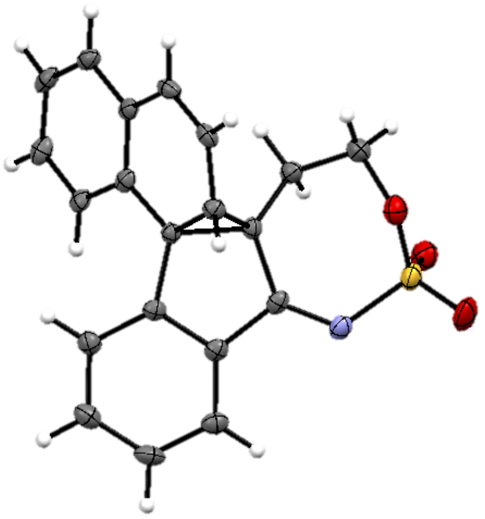

Yellow needle-like crystals of 4 were grown at 2-6 °C upon slow diffusion of pentane into a solution of 4 in chloroform.

A yellow, needle-like specimen of $C_{20}H_{15}NO_3S$, approximate dimensions 0.040 mm x 0.090 mm x 0.260 mm, was used for the X-ray crystallographic analysis. The X-ray intensity data were measured on a D8 QUEST ECO three-circle diffractometer system equipped with a Ceramic x-ray tube (Mo Kα, λ = 0.71073 Å) and a doubly curved silicon crystal Bruker Triumph monochromator. A total of 1211 frames were collected. The frames were integrated with the Bruker SAINT software package using a narrow-frame algorithm. The integration of the data using a monoclinic unit cell yielded a total of 69146 reflections to a maximum θ angle of 27.55° (0.77 Å resolution), of which 7107 were independent (average redundancy 9.729, completeness = 99.7%, Rint = 11.64%, Rsig = 6.05%) and 4900 (68.95%) were greater than 2σ(F2). The final cell constants of a = 9.9199(14) Å, b = 29.444(3) Å, c = 11.6896(15) Å, β = 115.018(4)°, volume = 3094.0(7) Å3, are based upon the refinement of the XYZ-centroids of 8488 reflections above 20 σ(I) with 5.314° < 2θ < 53.06°. Data were corrected for absorption effects using the Multi-Scan method (SADABS). The ratio of minimum to maximum apparent transmission was 0.899. The calculated minimum and maximum transmission coefficients (based on crystal size) are 0.9430 and 0.9910.

The structure was solved and refined using the Bruker SHELXTL Software Package, using the space group P 1 21/c 1, with Z = 8 for the formula unit, C20H15NO3S. The final anisotropic full-matrix least-squares refinement on F2 with 468 variables converged at R1 = 6.06%, for the observed data and wR2 = 13.70% for all data. The goodness-of-fit was 1.045. The largest peak in the final difference electron density synthesis was 0.605 e-/Å3 and the largest hole was -

0.549 e-/Å3 with an RMS deviation of 0.072 e-/Å3. On the basis of the final model, the calculated density was 1.500 g/cm3 and F(000), 1456 e-.

Sample and crystal data for 4

**Identification code**	NAM_naphthalene_grocs	
**Chemical formula**	$C_{20}H_{15}NO_3S$	
**Formula weight**	349.39 g/mol	
**Temperature**	100(2) K	
**Wavelength**	0.71073 Å	
**Crystal size**	0.040 x 0.090 x 0.260 mm	
**Crystal habit**	yellow needle	
**Crystal system**	monoclinic	
**Space group**	P 1 21/c 1	
**Unit cell dimensions**	a = 9.9199(14) Å	α = 90°
	b = 29.444(3) Å	β = 115.018(4)°
	c = 11.6896(15) Å	γ = 90°
**Volume**	3094.0(7) $Å^3$	
**Z**	8	
**Density (calculated)**	1.500 g/cm^3	
**Absorption coefficient**	0.230 mm^{-1}	
**F(000)**	1456	

Data collection and structure refinement for 4

**Diffractometer**	D8 QUEST ECO three-circle diffractometer
**Radiation source**	Ceramic x-ray tube (Mo Kα, λ = 0.71073 Å)
**Theta range for data collection**	2.04 to 27.55°
**Index ranges**	-12<=h<=12, -38<=k<=38, -15<=l<=15
**Reflections collected**	69146

**Independent reflections**	7107 [R(int) = 0.1164]
**Coverage of independent reflections**	99.7%
**Absorption correction**	Multi-Scan
**Max. and min. transmission**	0.9910 and 0.9430
**Structure solution technique**	direct methods
**Structure solution program**	XT, VERSION 2018/2
**Refinement method**	Full-matrix least-squares on F^2
**Refinement program**	SHELXL-2019/1 (Sheldrick, 2019)
**Function minimized**	$\Sigma w(F_o^2 - F_c^2)^2$
**Data / restraints / parameters**	7107 / 0 / 468
**Goodness-of-fit on F^2**	1.045
**Final R indices**	4900 data; $I>2\sigma(I)$ R1 = 0.0606, wR2 = 0.1177
	all data R1 = 0.1034, wR2 = 0.1370
**Weighting scheme**	$w=1/[\sigma^2(F_o^2)+(0.0303P)^2+6.3334P]$<br>where $P=(F_o^2+2F_c^2)/3$
**Largest diff. peak and hole**	0.605 and -0.549 eÅ^{-3}
**R.M.S. deviation from mean**	0.072 eÅ^{-3}

Atomic coordinates and equivalent isotropic atomic displacement parameters (Å2) for 4.

U(eq) is defined as one third of the trace of the orthogonalized U_{ij} tensor.

	x/a	y/b	z/c	U(eq)
N4A	0.1484(3)	0.54842(8)	0.5763(2)	0.0229(5)
S1A	0.30386(9)	0.51893(3)	0.62139(8)	0.02596(19)
O2A	0.3051(3)	0.49398(7)	0.5167(2)	0.0303(5)
O3A	0.3165(3)	0.49419(8)	0.7286(2)	0.0384(6)

	x/a	y/b	z/c	U(eq)
N25A	0.4345(2)	0.55416(7)	0.6688(2)	0.0279(5)
C5A	0.1351(3)	0.58945(9)	0.5335(3)	0.0179(6)
C6A	0.9957(3)	0.61504(9)	0.4988(3)	0.0183(6)
C7A	0.8687(3)	0.60281(10)	0.5136(3)	0.0227(6)
C8A	0.7518(3)	0.63340(11)	0.4728(3)	0.0265(7)
C9A	0.7615(3)	0.67518(11)	0.4214(3)	0.0249(7)
C10A	0.8896(3)	0.68762(10)	0.4090(3)	0.0208(6)
C11A	0.0065(3)	0.65687(9)	0.4472(3)	0.0170(6)
C12A	0.1560(3)	0.66220(9)	0.4464(2)	0.0159(5)
C13A	0.1775(3)	0.68544(9)	0.3431(3)	0.0177(6)
C14A	0.0613(3)	0.68887(9)	0.2230(3)	0.0189(6)
C15A	0.0846(3)	0.70747(9)	0.1237(3)	0.0227(6)
C16A	0.2248(4)	0.72343(9)	0.1445(3)	0.0238(7)
C17A	0.3393(3)	0.72220(9)	0.2642(3)	0.0217(6)
C18A	0.3185(3)	0.70354(9)	0.3659(3)	0.0187(6)
C19A	0.4356(3)	0.70501(10)	0.4946(3)	0.0208(6)
C20A	0.4183(3)	0.68756(9)	0.5934(3)	0.0193(6)
C21A	0.2832(3)	0.66360(9)	0.5795(3)	0.0181(6)
C22A	0.2413(3)	0.61815(9)	0.5054(3)	0.0170(6)
C23A	0.3488(3)	0.59483(10)	0.4635(3)	0.0205(6)
C24A	0.4798(3)	0.57491(10)	0.5759(3)	0.0254(7)
N4	0.6050(3)	0.32696(9)	0.0791(2)	0.0248(6)
S1	0.42812(10)	0.33949(4)	0.02969(11)	0.0237(3)
O2	0.4125(3)	0.37687(9)	0.1020(2)	0.0312(7)
O3	0.3549(3)	0.29794(10)	0.0298(3)	0.0333(7)
N25	0.3658(3)	0.35355(8)	0.8866(2)	0.0264(6)
S1'	0.4285(15)	0.3228(5)	0.9939(14)	0.060(4)

	x/a	y/b	z/c	U(eq)
O2'	0.385(3)	0.3159(10)	0.079(3)	0.046(7)
O3'	0.376(2)	0.3042(7)	0.875(2)	0.039(6)
N25'	0.395(3)	0.3815(8)	0.971(2)	0.049(7)
C5	0.6999(3)	0.35299(10)	0.0608(3)	0.0226(6)
C6	0.8588(3)	0.34367(10)	0.1256(3)	0.0211(6)
C7	0.9280(3)	0.30740(10)	0.2057(3)	0.0222(6)
C8	0.0814(3)	0.30378(10)	0.2541(3)	0.0239(6)
C9	0.1628(3)	0.33641(10)	0.2238(3)	0.0255(7)
C10	0.0941(3)	0.37311(10)	0.1461(3)	0.0223(6)
C11	0.9408(3)	0.37655(9)	0.0958(3)	0.0208(6)
C12	0.8395(3)	0.41174(10)	0.0095(3)	0.0223(6)
C13	0.8789(3)	0.43672(10)	0.9167(3)	0.0221(6)
C14	0.9801(3)	0.41908(10)	0.8743(3)	0.0251(7)
C15	0.0185(4)	0.44270(11)	0.7899(3)	0.0285(7)
C16	0.9561(4)	0.48480(11)	0.7463(3)	0.0318(8)
C17	0.8546(4)	0.50278(11)	0.7863(3)	0.0312(8)
C18	0.8151(4)	0.47981(10)	0.8722(3)	0.0268(7)
C19	0.7177(4)	0.50067(11)	0.9218(3)	0.0306(7)
C20	0.6904(4)	0.48195(10)	0.0147(3)	0.0291(7)
C21	0.7473(3)	0.43677(10)	0.0667(3)	0.0263(7)
C22	0.6812(3)	0.39460(10)	0.9818(3)	0.0242(7)
C23	0.5538(3)	0.39821(11)	0.8525(3)	0.0277(7)
C24	0.4039(4)	0.39889(11)	0.8548(3)	0.0298(7)

S12. Crystal structure of compound 5 with probability level of 50%

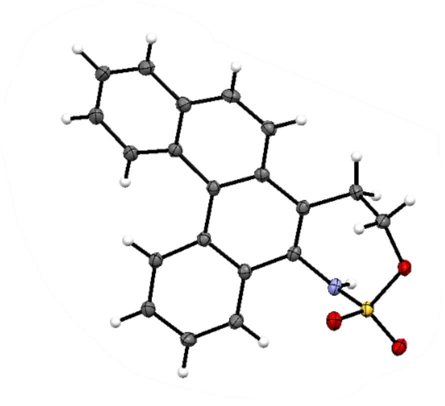

Yellow needle-like crystals of 5 were grown at 2-6 °C upon slow diffusion of pentane into a solution of 5 in chloroform.

A colorless, prism-like specimen of $C_{20}H_{15}NO_3S$, approximate dimensions 0.060 mm x 0.100 mm x 0.180 mm, was used for the X-ray crystallographic analysis. The X-ray intensity data were measured on a D8 QUEST ECO three-circle diffractometer system equipped with a Ceramic x-ray tube (Mo Kα, λ = 0.71073 Å) and a doubly curved silicon crystal Bruker Triumph monochromator. A total of 625 frames were collected. The total exposure time was 1.29 hours. The frames were integrated with the Bruker SAINT software package using a narrow-frame algorithm. The integration of the data using a triclinic unit cell yielded a total of 48752 reflections to a maximum θ angle of 27.53° (0.77 Å resolution), of which 7063 were independent (average redundancy 6.902, completeness = 99.5%, Rint = 9.63%, Rsig = 5.97%) and 5169 (73.18%) were greater than 2σ(F2). The final cell constants of a = 9.803(2) Å, b = 11.318(2) Å, c = 14.082(3) Å, α = 84.827(7)°, β = 82.553(7)°, γ = 87.067(7)°, volume = 1541.7(6) Å3, are based upon the refinement of the XYZ-centroids of 8695 reflections above 20 σ(I) with 5.427° < 2θ < 54.86°. Data were corrected for absorption effects using the Multi-Scan method (SADABS). The ratio of minimum to maximum apparent transmission was 0.898. The calculated minimum and maximum transmission coefficients (based on crystal size) are 0.9600 and 0.9860.

The structure was solved and refined using the Bruker SHELXTL Software Package, using the space group P -1, with Z = 4 for the formula unit, C20H15NO3S. The final anisotropic full-matrix least-squares refinement on F2 with 459 variables converged at R1 = 4.42%, for the observed data and wR2 = 10.94% for all data. The goodness-of-fit was 1.015. The largest peak in the final difference electron density synthesis was 0.315 e-/Å3 and the largest hole was -0.492 e-/Å3 with an RMS deviation of 0.070 e-/Å3. On the basis of the final model, the calculated density was 1.505 g/cm3 and F(000), 728 e-.

Sample and crystal data for 5.

**Identification code**	NAM_naphthalene_incolors
**Chemical formula**	$C_{20}H_{15}NO_3S$
**Formula weight**	349.39 g/mol
**Temperature**	100(2) K
**Wavelength**	0.71073 Å
**Crystal size**	0.060 x 0.100 x 0.180 mm
**Crystal habit**	colorless prism
**Crystal system**	triclinic
**Space group**	P -1
**Unit cell dimensions**	a = 9.803(2) Å α = 84.827(7)°
	b = 11.318(2) Å β = 82.553(7)°
	c = 14.082(3) Å γ = 87.067(7)°
**Volume**	1541.7(6) $Å^3$
**Z**	4
**Density (calculated)**	1.505 g/cm^3
**Absorption coefficient**	0.230 mm^{-1}
**F(000)**	728

Data collection and structure refinement for 5.

**Diffractometer**	D8 QUEST ECO three-circle diffractometer
**Radiation source**	Ceramic x-ray tube (Mo Kα, λ = 0.71073 Å)
**Theta range for data collection**	3.13 to 27.53°
**Index ranges**	-12<=h<=12, -14<=k<=14, -18<=l<=18

**Reflections collected**	48752
**Independent reflections**	7063 [R(int) = 0.0963]
**Coverage of independent reflections**	99.5%
**Absorption correction**	Multi-Scan
**Max. and min. transmission**	0.9860 and 0.9600
**Structure solution technique**	direct methods
**Structure solution program**	XT, VERSION 2018/2
**Refinement method**	Full-matrix least-squares on F^2
**Refinement program**	SHELXL-2019/1 (Sheldrick, 2019)
**Function minimized**	$\Sigma w(F_o^2 - F_c^2)^2$
**Data / restraints / parameters**	7063 / 0 / 459
**Goodness-of-fit on F^2**	1.015
**Δ/σ_{max}**	0.001
**Final R indices**	5169 data; $I>2\sigma(I)$ R1 = 0.0442, wR2 = 0.0935
	all data R1 = 0.0759, wR2 = 0.1094
**Weighting scheme**	$w=1/[\sigma^2(F_o^2)+(0.0371P)^2+1.3309P]$ where $P=(F_o^2+2F_c^2)/3$
**Largest diff. peak and hole**	0.315 and -0.492 eÅ^{-3}
**R.M.S. deviation from mean**	0.070 eÅ^{-3}

Atomic coordinates and equivalent isotropic atomic displacement parameters (Å^2) for 5.

U(eq) is defined as one third of the trace of the orthogonalized U_{ij} tensor.

	x/a	y/b	z/c	U(eq)
S1	0.78923(5)	0.66249(5)	0.53648(4)	0.01658(13)
O2	0.93354(15)	0.64204(13)	0.51452(11)	0.0216(3)
O3	0.73213(16)	0.78084(13)	0.54335(11)	0.0210(3)
O25	0.71335(16)	0.61534(13)	0.45632(10)	0.0206(3)
N4	0.74080(19)	0.58522(15)	0.63596(13)	0.0162(4)
C5	0.8019(2)	0.46695(18)	0.65191(15)	0.0155(4)
C6	0.9022(2)	0.45182(18)	0.71750(15)	0.0165(4)
C7	0.9482(2)	0.54956(19)	0.75904(15)	0.0200(5)
C8	0.0544(3)	0.5355(2)	0.81388(16)	0.0234(5)
C9	0.1208(2)	0.42395(19)	0.82749(16)	0.0216(5)
C10	0.0771(2)	0.32739(19)	0.78945(15)	0.0187(4)
C11	0.9626(2)	0.33626(18)	0.73682(14)	0.0155(4)
C12	0.9129(2)	0.23698(18)	0.69457(15)	0.0157(4)
C13	0.9470(2)	0.11376(18)	0.72451(15)	0.0164(4)
C14	0.9879(2)	0.07485(19)	0.81578(16)	0.0187(4)
C15	0.0204(2)	0.9578(2)	0.83980(17)	0.0232(5)
C16	0.0158(2)	0.8725(2)	0.77447(18)	0.0266(5)
C17	0.9692(2)	0.9049(2)	0.68804(18)	0.0233(5)
C18	0.9292(2)	0.02442(19)	0.66283(16)	0.0188(5)
C19	0.8661(2)	0.05607(19)	0.57864(16)	0.0197(5)
C20	0.8136(2)	0.16728(19)	0.55966(15)	0.0185(4)
C21	0.8316(2)	0.26043(18)	0.61887(15)	0.0153(4)
C22	0.7723(2)	0.37814(18)	0.59971(14)	0.0157(4)
C23	0.6784(2)	0.40644(19)	0.52212(15)	0.0192(5)
C24	0.7400(2)	0.48951(19)	0.43785(16)	0.0212(5)

	x/a	y/b	z/c	U(eq)
S1A	0.57082(5)	0.20149(5)	0.28568(4)	0.01705(13)
O2A	0.65621(16)	0.18777(14)	0.19740(11)	0.0216(3)
O3A	0.54376(16)	0.31650(13)	0.31991(11)	0.0212(3)
O25A	0.63797(15)	0.12896(13)	0.37011(11)	0.0197(3)
N4A	0.42513(19)	0.14420(16)	0.28011(14)	0.0175(4)
C5A	0.4214(2)	0.03488(19)	0.23444(15)	0.0169(4)
C6A	0.3653(2)	0.04314(18)	0.14494(15)	0.0164(4)
C7A	0.3249(2)	0.15294(19)	0.09940(16)	0.0206(5)
C8A	0.2856(2)	0.15982(19)	0.00891(17)	0.0227(5)
C9A	0.2906(2)	0.05730(19)	0.95965(16)	0.0205(5)
C10A	0.3260(2)	0.94897(19)	0.00330(15)	0.0177(4)
C11A	0.3583(2)	0.93667(18)	0.09917(15)	0.0158(4)
C12A	0.3970(2)	0.82392(18)	0.14760(14)	0.0153(4)
C13A	0.3657(2)	0.71032(18)	0.11638(15)	0.0171(4)
C14A	0.2606(2)	0.69575(19)	0.05898(15)	0.0198(5)
C15A	0.2345(3)	0.5862(2)	0.03232(16)	0.0254(5)
C16A	0.3137(3)	0.4853(2)	0.05995(16)	0.0278(6)
C17A	0.4115(3)	0.4944(2)	0.11928(16)	0.0244(5)
C18A	0.4364(2)	0.60533(19)	0.15112(15)	0.0193(5)
C19A	0.5262(2)	0.61257(19)	0.22183(16)	0.0199(5)
C20A	0.5374(2)	0.71583(19)	0.26146(16)	0.0192(5)
C21A	0.4695(2)	0.82410(19)	0.22794(15)	0.0169(4)
C22A	0.4766(2)	0.93229(19)	0.27377(15)	0.0170(4)
C23A	0.5382(2)	0.9324(2)	0.36736(15)	0.0208(5)
C24A	0.6686(2)	0.00174(19)	0.36126(17)	0.0210(5)

S13. Computational details

All DFT static calculations were performed with the Gaussian 16 software package.[16] Geometry optimizations were performed without symmetry constraints and with analytical frequency calculations for the characterization of the located stationary points. These frequencies were used to calculate unscaled zero-point energies (ZPEs) as well as thermal corrections and entropy effects at 298 K. For this calculations, we used the M06 hybrid functional of Truhlar and Zhao,[17] together with the Grimme D3 correction term to the electronic energy.[18] The electronic configuration of the molecular systems was described with the 6-311G,[19] including diffuse functions ("+" keyword in Gaussian)[20] and single first polarization functions (** notation in Gaussian),[21] whereas for rhodium atoms the small-core quasi-relativistic Stuttgart/Dresden effective core potential, with an associated valence basis set (standard SDD keywords in Gaussian16) were employed.[22] Energies were obtained by single-point calculations on the optimized geometries with the B3LYP functional,[23] with the Grimme D3 correction term, coupled with the 6-31G(d) basis set.[18,24] Solvent corrections were considered using the universal solvation model SMD of Cramer and Truhlar,[25] using dichloromethane as the solvent. The reported free energies in this work include energies obtained at the M06-D3/6-311+G**~sdd level of theory (with solvent corrections included) corrected with zero-point energies, thermal corrections and entropy effects evaluated at 298 K, achieved at B3LYP-D3/6-31G(d)~sdd level.

S13.1. Insight into the formation of intermediate B

Reference structure for the mechanistic scheme depicted in Figure 2 of the manuscript is the rhodium nitrene in the triplet state (**A-t**) with the arm folded so that the nitrene is positioned close to the alkyne. The corresponding singlet structure for this "folded" intermediate, whether open-shell or closed-shell, could not be located. In contrast, a conformer with the rhodium nitrene pointing away from the alkyne **(A-t (c))** lies 6.2 kcal/mol higher in energy on the triplet state and at least 13.3 kcal/mol higher than **A-t** for the open-shell and closed-shell singlet structures.

The subsequent C–N bond formation proceeds via a triple transition state (**TSAB-t**) with an energy barrier of only 13.6 kcal/mol, leading to a radical intermediate in a highly exergonic process (this intermediate is located 28.7 kcal/mol below **A-t**). For both **TSAB-t** and this intermediate, singlet states could not be located. Single-point energy calculations on the **TSAB-t** geometry in the singlet surface destabilized the structure by 9.0 kcal/mol (closed-shell) and 2.7 kcal/mol (open-shell), while for the following intermediate, the singlet was 8.2 kcal/mol (closed-shell) and 8.3 kcal/mol (open-shell) lower in energy than the triplet. In these

cases, locating the transition state or intermediate as a singlet was not feasible, as the computations directly evolve toward intermediate **B**.

Overall, these results suggest that the intersystem crossing likely occurs near the transition-state maximum **TSAB-t**, located 13.6 kcal/mol above **A-t**. Attempts to locate the exact crossing point on either multiplicity surface were unsuccessful.

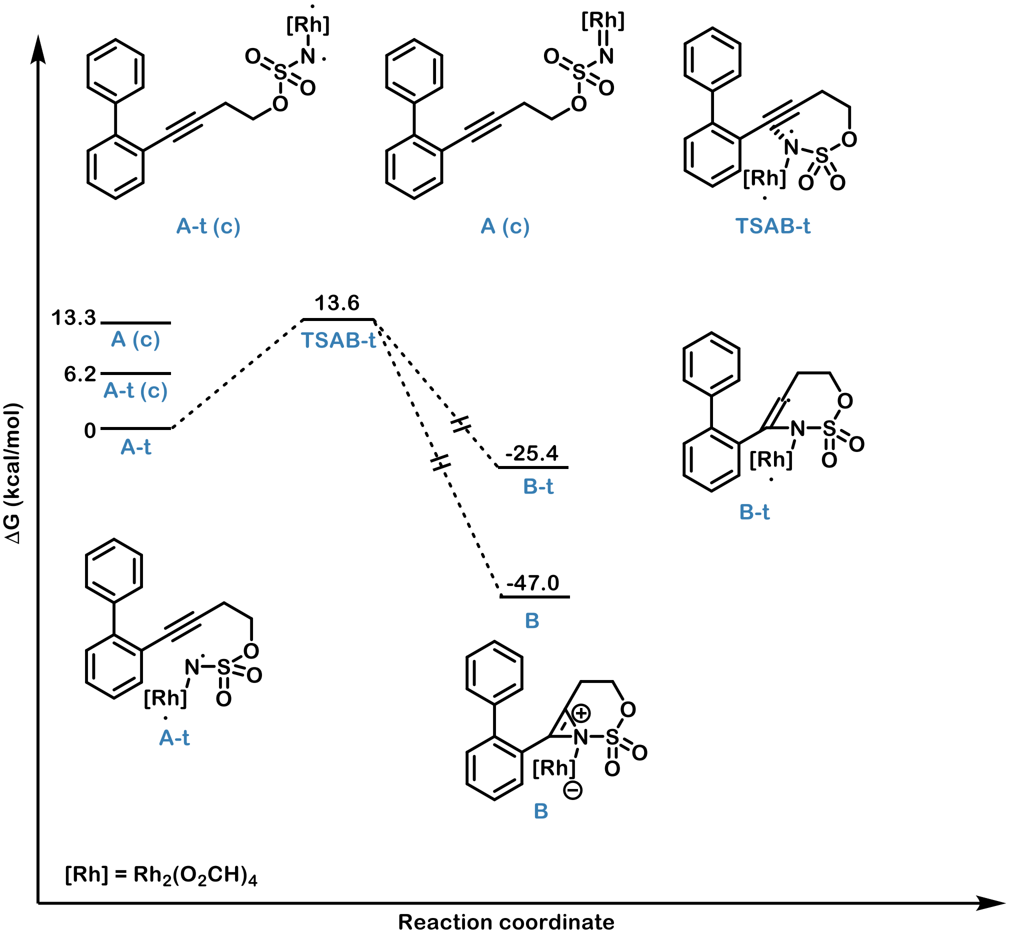

Figure S1. Insight into the formation of intermediate **B**.

S13.2. XYZ coordinates

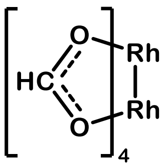

Rh ($Rh_2C_4H_4O_8$)
SCF Done = -975.628392769 a.u.

45	0.000159000	-0.000723000	-1.201521000
45	-0.000157000	0.000701000	1.201509000
8	1.463739000	-1.454787000	1.139922000
8	1.455608000	1.464301000	1.138456000
8	-1.464045000	1.456219000	1.138122000
8	-1.455969000	-1.462904000	1.139602000
8	-1.464073000	1.454418000	-1.139910000
8	1.455574000	1.463257000	-1.139575000
8	-1.455282000	-1.464600000	-1.138422000

8	1.464430000	-1.455811000	-1.138101000
6	1.847544000	1.857478000	-0.000763000
6	1.858245000	-1.846782000	0.001188000
6	-1.847552000	-1.857477000	0.000750000
6	-1.858236000	1.846781000	-0.001202000
1	2.622327000	2.636157000	-0.001128000
1	-2.637680000	2.620799000	-0.001828000
1	-2.622451000	-2.636046000	0.001110000
1	2.637853000	-2.620640000	0.001804000

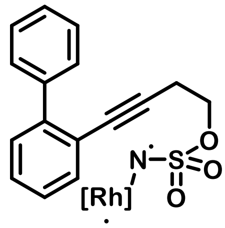

A-t ($Rh_2C_{20}NH_{17}SO_{11}$)
SCF Done = -2271.210685 a.u.

16	-1.040878000	-2.969640000	-0.208530000
7	-0.569456000	-1.496754000	0.424664000
8	-2.344509000	-3.302680000	0.718113000
8	-1.581552000	-2.808437000	-1.548948000
8	0.008170000	-3.940122000	0.086555000
6	-2.760323000	2.192890000	0.237033000
6	-2.529734000	3.535658000	-0.090958000
1	-3.012884000	3.943045000	-0.974515000
6	-1.674333000	4.336306000	0.666314000
1	-1.503273000	5.369255000	0.375545000
6	-1.031589000	3.808221000	1.791853000
1	-0.362054000	4.427086000	2.382179000
6	-1.260621000	2.487268000	2.153714000
1	-0.776244000	2.057773000	3.022834000
6	-2.118584000	1.666145000	1.392174000
6	-2.347393000	0.335495000	1.827506000
6	-2.535023000	-0.800310000	2.228720000
6	-2.936781000	-2.106282000	2.742564000
1	-2.821899000	-2.138906000	3.835108000
1	-4.001724000	-2.259478000	2.524333000
6	-2.153378000	-3.286169000	2.151276000
1	-1.089999000	-3.229602000	2.400581000
1	-2.554479000	-4.231065000	2.526466000
45	1.071162000	-0.419404000	0.066468000
45	3.098188000	0.831112000	-0.494286000
8	1.884847000	2.350750000	-1.204181000
8	3.005030000	1.665864000	1.399069000
8	4.203074000	-0.749583000	0.250719000
8	3.082762000	-0.068418000	-2.352819000
8	2.314283000	-1.885273000	0.835908000
8	1.107938000	0.514015000	1.915573000
8	1.220863000	-1.266402000	-1.812670000
8	0.003842000	1.148876000	-0.755876000
6	2.054163000	1.326515000	2.155281000
6	0.635767000	2.166752000	-1.175463000
6	2.165740000	-0.906730000	-2.579886000
6	3.569504000	-1.727879000	0.738745000
1	2.033970000	1.791116000	3.151451000
1	4.176259000	-2.553876000	1.135180000
1	2.176288000	-1.392594000	-3.565017000
1	0.004788000	2.982190000	-1.552005000
6	-3.642356000	1.368269000	-0.624135000
6	-3.197957000	0.141263000	-1.141480000
6	-4.927126000	1.823363000	-0.962004000
6	-4.024125000	-0.622024000	-1.964290000
6	-5.755111000	1.057779000	-1.783185000
6	-5.306354000	-0.167398000	-2.283558000
1	-2.193837000	-0.197721000	-0.922503000

1	-5.281361000	2.769196000	-0.559762000
1	-3.653787000	-1.567604000	-2.347096000
1	-6.751752000	1.415929000	-2.027776000
1	-5.952835000	-0.763599000	-2.922239000

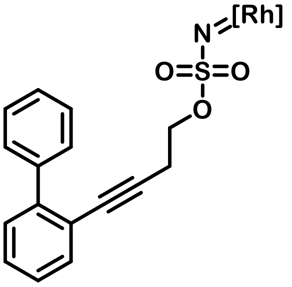

A ($Rh_2C_{20}NH_{17}SO_{11}$)

SCF Done = -2271.19050773 a.u.

16	-0.241503000	3.033568000	0.836701000
7	-1.431870000	1.963990000	1.244583000
8	0.238850000	2.841808000	-0.700703000
8	0.908574000	2.813063000	1.713409000
8	-1.003117000	4.283165000	0.846396000
6	5.253687000	-0.986294000	0.100219000
6	6.211289000	-2.005402000	0.222768000
1	5.986106000	-2.856649000	0.858679000
6	7.445777000	-1.938289000	-0.420944000
1	8.164413000	-2.743343000	-0.295170000
6	7.756828000	-0.832634000	-1.214551000
1	8.716638000	-0.768800000	-1.719222000
6	6.829237000	0.194700000	-1.353964000
1	7.055267000	1.058912000	-1.971046000
6	5.579414000	0.139367000	-0.708216000
6	4.654073000	1.210226000	-0.898110000
6	3.860919000	2.119006000	-1.023231000
6	2.697988000	3.005803000	-0.998147000
1	2.715368000	3.625419000	-0.092860000
1	2.655376000	3.679195000	-1.862098000
6	1.465913000	2.092978000	-0.947386000
1	1.599551000	1.334380000	-0.180985000
1	1.290054000	1.599984000	-1.904438000
6	3.950317000	-1.136146000	0.806664000
6	3.371335000	-0.084501000	1.540622000
6	3.283119000	-2.374903000	0.789048000
6	2.166935000	-0.261199000	2.221720000
6	2.085785000	-2.558605000	1.485648000
6	1.521391000	-1.499918000	2.200034000
1	3.870758000	0.876490000	1.584459000
1	3.702444000	-3.197038000	0.215291000
1	1.729420000	0.575525000	2.756702000
1	1.588391000	-3.524706000	1.453546000
1	0.578208000	-1.626045000	2.723321000
45	-2.096049000	0.473686000	0.239841000
45	-2.787403000	-1.655180000	-0.765638000
8	-2.835990000	-0.660253000	-2.583921000
8	-0.806946000	-2.131781000	-1.094363000
8	-2.703221000	-2.542784000	1.091047000
8	-4.741953000	-1.100329000	-0.397462000
8	-2.054272000	-0.578012000	2.048417000
8	-0.179578000	-0.137954000	-0.203255000
8	-4.097328000	0.871280000	0.538701000
8	-2.225081000	1.314737000	-1.628131000
6	0.040841000	-1.266616000	-0.749588000
6	-2.551580000	0.566784000	-2.605479000
6	-4.953334000	0.007492000	0.166644000
6	-2.367289000	-1.808478000	2.060896000
1	1.098798000	-1.492395000	-0.927231000
1	-2.336747000	-2.286491000	3.049972000
1	-6.000159000	0.273607000	0.364558000
1	-2.586766000	1.070414000	-3.580865000

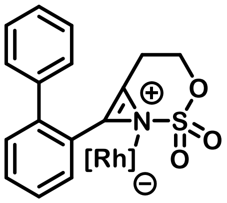

B ($Rh_2C_{20}NH_{17}SO_{11}$)
SCF Done = -2271.28906272 a.u.

16	1.118965000	-1.589232000	2.009092000
7	0.713261000	-0.530031000	0.586083000
8	1.853537000	-2.827200000	1.182287000
8	2.192975000	-0.980381000	2.781888000
8	-0.119966000	-2.044979000	2.606487000
6	3.935600000	1.635620000	0.084177000
6	4.522107000	2.867499000	0.394106000
1	5.554475000	3.042293000	0.105147000
6	3.816941000	3.843295000	1.101335000
1	4.300425000	4.786342000	1.340797000
6	2.505063000	3.604069000	1.520243000
1	1.963587000	4.357809000	2.083864000
6	1.888270000	2.397068000	1.207311000
1	0.859836000	2.202088000	1.488830000
6	2.591189000	1.425471000	0.476980000
6	1.915783000	0.219746000	0.098159000
6	1.643177000	-0.874943000	-0.530688000
6	1.772681000	-2.196140000	-1.164954000
1	1.162940000	-2.260351000	-2.073010000
1	2.823644000	-2.378665000	-1.411451000
6	1.256607000	-3.184667000	-0.097079000
1	0.164459000	-3.154086000	-0.026200000
1	1.580995000	-4.208085000	-0.291177000
45	-1.360558000	-0.073816000	0.047849000
45	-3.635016000	0.455255000	-0.661340000
8	-2.999052000	2.413644000	-0.938358000
8	-3.037933000	-0.003790000	-2.602872000
8	-4.111116000	-1.537633000	-0.335199000
8	-4.060764000	0.872386000	1.322483000
8	-1.971988000	-2.046044000	0.267737000
8	-0.916568000	-0.517480000	-1.941251000
8	-1.922643000	0.412797000	1.972546000
8	-0.875423000	1.915662000	-0.272907000
6	-1.849401000	-0.376321000	-2.794391000
6	-1.794406000	2.689872000	-0.686976000
6	-3.127222000	0.760370000	2.167364000
6	-3.191659000	-2.316814000	0.039082000
1	-1.577047000	-0.611999000	-3.834256000
1	-3.476866000	-3.368641000	0.186884000
1	-3.386326000	0.994873000	3.209834000
1	-1.492030000	3.735641000	-0.844971000
6	4.719132000	0.573855000	-0.600072000
6	4.838414000	-0.703716000	-0.024154000
6	5.373320000	0.835982000	-1.812817000
6	5.584891000	-1.696128000	-0.659435000
6	6.117979000	-0.159756000	-2.447540000
6	6.223402000	-1.428707000	-1.874545000
1	4.352717000	-0.911014000	0.925422000
1	5.280820000	1.820166000	-2.264329000
1	5.676797000	-2.675303000	-0.196940000
1	6.612092000	0.055593000	-3.391046000
1	6.804360000	-2.203270000	-2.367740000

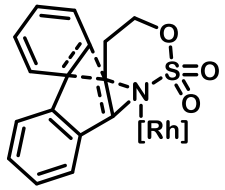

TSBC ($Rh_2C_{20}NH_{17}SO_{11}$)
SCF Done = -2270.80276224 a.u.

16	1.033769000	1.284005000	-2.527346000
7	0.705444000	0.314424000	-1.224375000
8	1.806460000	2.612749000	-1.831116000
8	2.082822000	0.721891000	-3.369362000
8	-0.231879000	1.728254000	-3.080953000
6	3.647147000	-1.590003000	0.135098000
6	4.350636000	-2.788421000	0.037129000
1	5.267104000	-2.929592000	0.603298000
6	3.863829000	-3.804329000	-0.794848000
1	4.413840000	-4.737451000	-0.877019000
6	2.684398000	-3.623824000	-1.522845000
1	2.319849000	-4.417109000	-2.168659000
6	1.976790000	-2.424012000	-1.432559000
1	1.066158000	-2.248782000	-1.995142000
6	2.461391000	-1.416332000	-0.601263000
6	1.830650000	-0.108090000	-0.491683000
6	2.186312000	0.858885000	0.340823000
6	2.152353000	2.292206000	0.583311000
1	1.703770000	2.528552000	1.554496000
1	3.183233000	2.673919000	0.569893000
6	1.342788000	3.008448000	-0.547010000
1	0.273862000	2.819913000	-0.421321000
1	1.532881000	4.082835000	-0.469162000
45	-1.210838000	0.040079000	-0.221582000
45	-3.324418000	-0.373755000	0.938904000
8	-4.099676000	-1.000211000	-0.871183000
8	-2.639994000	-2.300313000	1.315305000
8	-2.398772000	0.293573000	2.684888000
8	-3.868330000	1.573660000	0.483784000
8	-0.423050000	0.682531000	1.612175000
8	-0.669826000	-1.915173000	0.231712000
8	-1.873836000	1.979624000	-0.544221000
8	-2.120073000	-0.630706000	-1.944931000
6	-1.499318000	-2.622629000	0.882234000
6	-3.335059000	-0.984465000	-1.879305000
6	-3.033669000	2.291600000	-0.135205000
6	-1.196711000	0.662525000	2.621207000
1	-1.173653000	-3.652167000	1.095038000
1	-0.749984000	1.015186000	3.564664000
1	-3.347054000	3.323936000	-0.349310000
1	-3.778230000	-1.323151000	-2.826863000
6	4.064393000	-0.448491000	0.992763000
6	5.238126000	0.290592000	0.745424000
6	3.251439000	-0.093061000	2.105860000
6	5.604255000	1.327260000	1.595641000
6	3.662573000	0.924962000	2.988157000
6	4.822534000	1.639161000	2.724531000
1	5.844611000	0.039760000	-0.119033000
1	2.383600000	-0.700673000	2.341504000
1	6.509402000	1.893181000	1.395214000
1	3.061685000	1.147883000	3.864415000
1	5.137104000	2.435238000	3.393138000

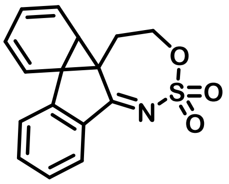

C ($C_{16}NH_{13}SO_3$)
SCF Done = -1295.66855493 a.u.

16	2.776720000	-0.874473000	-0.198341000
7	1.261784000	-1.583695000	0.041110000
8	2.957980000	0.233320000	1.000673000
8	3.762210000	-1.890281000	0.100978000
8	2.757132000	-0.174566000	-1.482750000
6	-2.137131000	-0.531726000	0.084412000
6	-3.477006000	-0.901963000	0.005960000
1	-4.262783000	-0.151940000	-0.016080000
6	-3.790811000	-2.264246000	-0.042118000
1	-4.832485000	-2.570575000	-0.086980000
6	-2.784942000	-3.241238000	-0.041640000
1	-3.056448000	-4.291895000	-0.086294000
6	-1.441662000	-2.872257000	0.013782000
1	-0.645466000	-3.610052000	0.010370000
6	-1.133454000	-1.512860000	0.092124000
6	0.189358000	-0.872115000	0.135857000
6	-0.015400000	0.625678000	0.234583000
6	0.836491000	1.448049000	1.183487000
1	0.444952000	2.468470000	1.220384000
1	0.740168000	1.034966000	2.195602000
6	2.313411000	1.517436000	0.823741000
1	2.473222000	1.867607000	-0.199735000
1	2.846246000	2.176134000	1.513855000
6	-1.531806000	0.832516000	0.134579000
6	-2.229118000	1.960777000	0.764822000
6	-0.583377000	1.196744000	-1.072646000
6	-1.962547000	3.246894000	0.437039000
6	-0.478909000	2.621751000	-1.406458000
6	-1.074469000	3.582136000	-0.659897000
1	-2.997364000	1.724423000	1.496967000
1	-0.561512000	0.509748000	-1.915571000
1	-2.487633000	4.052326000	0.943435000
1	0.043037000	2.880350000	-2.324218000
1	-0.974293000	4.626631000	-0.943021000

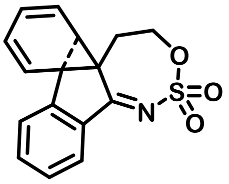

TSCD ($C_{16}NH_{13}SO_3$)
SCF Done = -1295.65925980 a.u.

16	2.683193000	-1.022335000	-0.200453000
7	1.127118000	-1.655004000	0.005252000
8	2.911138000	0.002695000	1.059080000
8	3.606008000	-2.108751000	0.044286000
8	2.710862000	-0.254977000	-1.445942000
6	-2.198242000	-0.398701000	0.101889000
6	-3.562167000	-0.686097000	0.021734000
1	-4.300783000	0.110269000	0.011525000
6	-3.958228000	-2.023100000	-0.046314000
1	-5.016815000	-2.263796000	-0.094722000
6	-3.014660000	-3.063168000	-0.064738000
1	-3.352823000	-4.093303000	-0.127309000
6	-1.651726000	-2.780901000	-0.011194000
1	-0.903336000	-3.566757000	-0.034213000
6	-1.258166000	-1.443968000	0.083267000
6	0.097174000	-0.887425000	0.114618000
6	-0.024184000	0.638484000	0.216362000
6	0.850824000	1.329788000	1.260211000
1	0.526126000	2.368596000	1.363008000

1	0.685220000	0.846548000	2.230832000
6	2.339537000	1.330332000	0.950120000
1	2.552163000	1.737346000	-0.042059000
1	2.886024000	1.910324000	1.697419000
6	-1.509659000	0.902260000	0.199748000
6	-2.119922000	2.107710000	0.645391000
6	-0.241519000	1.246581000	-1.142200000
6	-1.625973000	3.369330000	0.394187000
6	-0.146928000	2.633973000	-1.420232000
6	-0.657974000	3.628440000	-0.611165000
1	-3.072809000	2.011217000	1.161884000
1	-0.212393000	0.554727000	-1.980135000
1	-2.124758000	4.220568000	0.850641000
1	0.254264000	2.908360000	-2.393974000
1	-0.474191000	4.665350000	-0.881903000

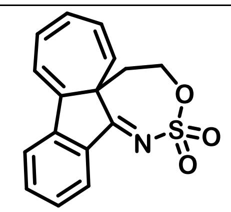

D ($C_{16}NH_{13}SO_3$)

SCF Done = -1295.67035707 a.u.

16	2.631852000	-1.110875000	-0.167152000
7	1.022146000	-1.631704000	-0.228189000
8	2.718851000	-0.274827000	1.240038000
8	2.922058000	-0.210812000	-1.283774000
8	3.428260000	-2.295867000	0.064109000
6	-2.269731000	-0.336989000	0.115555000
6	-3.636508000	-0.639993000	0.177938000
1	-4.377059000	0.139987000	0.328902000
6	-4.034797000	-1.967272000	0.040468000
1	-5.091771000	-2.214433000	0.091719000
6	-3.096943000	-2.994930000	-0.167917000
1	-3.439103000	-4.020030000	-0.275008000
6	-1.738859000	-2.703469000	-0.237383000
1	-0.993724000	-3.476611000	-0.395325000
6	-1.339666000	-1.371388000	-0.092931000
6	0.019542000	-0.829808000	-0.106765000
6	-0.068853000	0.708114000	0.072678000
6	0.708189000	1.163695000	1.343351000
1	0.443952000	2.206534000	1.542659000
1	0.366504000	0.572703000	2.201458000
6	2.226140000	1.089093000	1.248785000
1	2.608912000	1.617006000	0.371411000
1	2.679941000	1.519536000	2.144606000
6	-1.576092000	0.945392000	0.215275000
6	-2.157851000	2.158805000	0.417706000
6	0.359089000	1.448408000	-1.185849000
6	-1.538667000	3.446097000	0.275735000
6	0.251984000	2.788626000	-1.346932000
6	-0.457753000	3.728100000	-0.513292000
1	-3.220122000	2.168906000	0.659958000
1	0.829353000	0.866369000	-1.971149000
1	-2.059066000	4.285341000	0.733217000
1	0.725107000	3.212070000	-2.232335000
1	-0.186276000	4.775740000	-0.629868000

S13.3. Insight into the thermodynamic evolution of 4 to 5

DFT calculations were done to unveil the thermodynamic conversion of product **4** to product **5** and to rationalize this reactivity in front of the expected naphthalene dearomatization.

As detailed in the manuscript (**Scheme 3b**), replacing the upper phenyl moiety for a naphthyl unit led the nucleophilic attack of the aryl ring to the azirine stop at the cyclopropanated product **4**, which underwent a thermodynamically driven evolution to compound **5**. According to DFT calculations, product **5** is 25.5 Kcal/mol lower in energy than product 4. In addition, the computed free-energy of the putative dearomatized naphthalene product is 25.2 Kcal/mol higher than that of 4, and thus 50.7 Kcal/mol higher than the experimentally isolated product **5**. These results confirm the accessible structural diversity observed experimentally and highlight the thermodynamic preference for the formation of **5** over the dearomatized product.

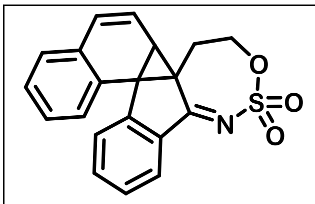

4 ($C_{20}H_{15}NSO_3$)
SCF Done = -1449.24488860 a.u.

16	3.489245000	-0.694188000	-0.149840000
7	2.581523000	0.719701000	-0.304529000
8	2.787706000	-1.801797000	-1.140149000
8	4.774262000	-0.432566000	-0.760918000
8	3.374113000	-1.162871000	1.230963000
6	-0.774771000	1.780746000	0.231879000
6	-1.631777000	2.858924000	0.444889000
1	-2.680993000	2.699139000	0.674472000
6	-1.114895000	4.154667000	0.347942000
1	-1.776243000	5.004047000	0.496908000
6	0.241101000	4.377530000	0.068724000
1	0.616983000	5.394337000	0.002334000
6	1.106692000	3.301915000	-0.118701000
1	2.162676000	3.446016000	-0.324567000
6	0.582150000	2.009542000	-0.045703000
6	1.299592000	0.729925000	-0.152188000
6	0.293901000	-0.384835000	0.025746000
6	0.361947000	-1.634004000	-0.833801000
1	-0.507885000	-2.262255000	-0.621490000
1	0.290753000	-1.342746000	-1.889354000
6	1.610948000	-2.483155000	-0.646048000
1	1.765452000	-2.766403000	0.398720000
1	1.553389000	-3.387893000	-1.256250000
6	-1.045923000	0.309105000	0.321814000
6	-2.349562000	-0.280497000	-0.101329000
6	-0.282878000	-0.395422000	1.460865000
6	-2.811629000	-1.480165000	0.497639000
6	-0.892037000	-1.605894000	2.038826000
6	-2.051154000	-2.109448000	1.573491000
1	0.247625000	0.239880000	2.166243000
1	-0.394662000	-2.058241000	2.892806000
1	-2.480925000	-2.996951000	2.032611000
6	-4.029695000	-2.034278000	0.069754000
1	-4.379309000	-2.953668000	0.533631000
6	-3.126047000	0.323656000	-1.097592000

	1 -2.775464000 1.239508000 -1.563616000<br>6 -4.331795000 -0.241891000 -1.511873000<br>1 -4.910395000 0.238564000 -2.295867000<br>6 -4.786427000 -1.425729000 -0.926185000<br>1 -5.724669000 -1.870515000 -1.245511000
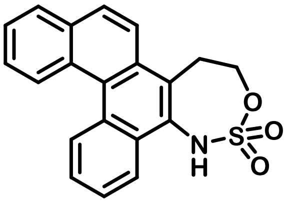	**5** ($C_{20}H_{15}NSO_3$)<br>SCF Done = -1449.28678202 a.u.<br><br>16 3.841711000 -0.127569000 0.173638000<br>7 2.633436000 0.345370000 -0.909642000<br>8 3.919930000 -1.724556000 -0.115520000<br>8 5.080002000 0.430184000 -0.335830000<br>8 3.397480000 0.062569000 1.547944000<br>6 -0.886736000 1.351564000 -0.005025000<br>6 -1.556490000 2.529520000 0.420945000<br>1 -2.579936000 2.463541000 0.764150000<br>6 -0.916417000 3.751655000 0.485689000<br>1 -1.461147000 4.625444000 0.832146000<br>6 0.443308000 3.858523000 0.145764000<br>1 0.950785000 4.817033000 0.204340000<br>6 1.146995000 2.723817000 -0.200395000<br>1 2.215592000 2.801283000 -0.371197000<br>6 0.515879000 1.457794000 -0.266103000<br>6 1.261645000 0.260172000 -0.534378000<br>6 0.706459000 -0.989963000 -0.395527000<br>6 1.555533000 -2.211795000 -0.713312000<br>1 0.934600000 -3.108836000 -0.746931000<br>1 1.969549000 -2.091500000 -1.722773000<br>6 2.738650000 -2.504067000 0.221939000<br>1 2.492383000 -2.343003000 1.275653000<br>1 3.084582000 -3.530926000 0.086360000<br>6 -1.523939000 0.046393000 -0.049835000<br>6 -2.966609000 -0.144892000 -0.063376000<br>6 -0.693126000 -1.105511000 -0.057969000<br>6 -3.504048000 -1.430004000 0.264580000<br>6 -1.275315000 -2.377892000 0.256603000<br>6 -2.611954000 -2.520580000 0.478770000<br>1 -0.633336000 -3.243252000 0.371842000<br>1 -3.022148000 -3.485698000 0.765233000<br>6 -4.905783000 -1.623594000 0.310529000<br>1 -5.283642000 -2.603781000 0.591565000<br>6 -3.892380000 0.848191000 -0.484159000<br>1 -3.526503000 1.784960000 -0.884376000<br>6 -5.255980000 0.624070000 -0.467585000<br>1 -5.929737000 1.403239000 -0.813517000<br>6 -5.775750000 -0.610120000 -0.030901000<br>1 -6.849485000 -0.772971000 -0.003525000<br>1 2.934056000 1.162713000 -1.429975000
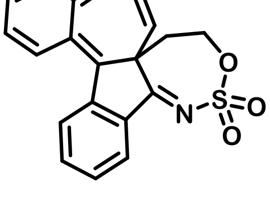	**"Putative dearomatized naphthalene"** ($C_{20}H_{15}NSO_3$)<br>SCF Done = -1449.20316772 a.u.<br><br>16 3.463974000 -0.772416000 0.346917000<br>7 2.310231000 0.267979000 0.695584000<br>8 3.706842000 -0.654387000 -1.323679000<br>8 4.733924000 -0.351826000 0.919010000<br>8 3.065324000 -2.190856000 0.547800000<br>6 -0.885282000 1.612340000 0.211801000<br>6 -1.659430000 2.755923000 0.506178000

	1	-2.727963000	2.684338000	0.677248000
	6	-1.015341000	3.976898000	0.644643000
	1	-1.593013000	4.864393000	0.886389000
	6	0.382568000	4.074188000	0.506621000
	1	0.862950000	5.041145000	0.628804000
	6	1.157610000	2.944833000	0.250451000
	1	2.240581000	2.990880000	0.199902000
	6	0.515475000	1.721467000	0.097528000
	6	1.171046000	0.383036000	-0.161429000
	6	-0.063527000	-0.567458000	0.117602000
	6	1.513823000	0.314003000	-1.699563000
	1	0.598255000	0.232657000	-2.298301000
	1	2.001909000	1.258782000	-1.961152000
	6	2.484958000	-0.816114000	-2.045838000
	1	2.054066000	-1.806957000	-1.846479000
	1	2.753403000	-0.770525000	-3.106125000
	6	-1.252567000	0.208973000	0.114152000
	6	-2.601825000	-0.275453000	-0.087728000
	6	0.084772000	-1.912702000	0.401973000
	6	-3.070931000	-1.567821000	0.338056000
	6	-0.911663000	-2.792724000	0.881644000
	6	-2.275594000	-2.627375000	0.881040000
	1	1.105609000	-2.295990000	0.386774000
	1	-0.547042000	-3.745176000	1.260139000
	1	-2.853118000	-3.465507000	1.265467000
	6	-4.458284000	-1.851611000	0.204102000
	1	-4.817687000	-2.813316000	0.558746000
	6	-3.529365000	0.587306000	-0.731431000
	1	-3.166621000	1.524167000	-1.135426000
	6	-4.857703000	0.250359000	-0.896691000
	1	-5.529181000	0.930432000	-1.412173000
	6	-5.335734000	-0.973582000	-0.394677000
	1	-6.384107000	-1.236895000	-0.499250000

S14. Photophysical characterization

Absorption and emission spectra of the compounds were recorded in 10^{-5} M dichloromethane solutions, and the results are summarized in **Table S1**.

Table S1. Absorption and emission data of the compounds recorded in 10^{-5} M dichloromethane solutions at room temperature. [a] very weak emission recorded

Compound	Absorption (nm) (10^4 ε, $cm^{-1}·M^{-1}$)	Emission in solution (nm)	Emission in solid state (nm)
**2a**	274 (2.9), 326 (0.7), 396 (0.3)	543	526
**2b** (R^1 = Me)	277 (2.9), 330 (0.9), 403 (0.4)	569	537
**2c** (R^1 = Ph)	275 (1.8), 338 (0.9), 410 (0.4)	579	565
**2d** (R^1 = OMe)	276 (2.7), 337 (0.7), 419 (0.3)	606	632
**2e** (R^1 = OPh)	275 (2.3), 336 (0.8), 415 (0.3)	605	588
**2f** (R^1 = F)	277 (4.5), 329 (1.0), 397 (0.5)	548	522
**2g** (R^1 = Br)	270 (1.7)	544	544
**2h** (R^1 = CF_3)	275 (2.1), 323 (0.4), 382 (0.2)	454	-[a]
**2i** (R^2 = 4-Me)	280 (2.2), 326 (0.7), 404 (0.3)	516	529
**2j** (R^2 = 5-Me)	288 (4.1), 391 (0.5)	540	509
**2k** (R^2 = 5-F)	274 (4.7), 328 (1.1), 396 (0.5)	512	513
**2l** (R^2 = 4,5-F,F)	275 (3.7), 328 (0.9), 390 (0.3)	508	539
**2o** (R^1=F, R^2=Me)	277 (1.6), 327 (0.4)	507	512

Quantum yields and lifetimes (**Tables S2** and **S3**) have been recorded on Absolute PL quantum yield spectrometer from Hamamatsu Photonics upon excitation the samples at the absorption maxima.

Table S2. Emission quantum yields and lifetimes recorded for the compounds in solution and in solid state. *Lifetime shorter than the IRF

Compound	QY (solution)	QY (solid)	Lifetime (ns) solution	Lifetime (ns) solid
**2a**	0.07	0.44	3.0	15.8
**2b** (R^1 = Me)	0.02	0.40	0.86	11.9
**2c** (R^1 = Ph)	0.12	0.20	2.48	14.6
**2d** (R^1 = OMe)	< 1%	0.03	2.89	10.8
**2e** (R^1 = OPh)	< 1%	0.44	1.77	9.4
**2f** (R^1 = F)	0.06	0.46	2.93	10.7
**2g** (R^1 = Br)	0.05	0.09	1.0	4.9
**2h** (R^1 = CF_3)	0.02	< 1%	*	-
**2i** (R^2 = 4-Me)	0.18	0.71	7.63	13.4
**2j** (R^2 = 5-Me)	0.06	0.19	1.63	10.5
**2k** (R^2 = 5-F)	0.01	0.24	*	12.6
**2l** (R^2 = 4,5-F,F)	0.03	0.29	1.13	14.0
**2o** (R^1=F, R^2=Me)	0.02	0.26	*	7.42

Table S3. k_r and k_{nr} values calculated for the compounds both in solution and in solid state.

Compound	Solution		Solid State	
	k_r	k_{nr}	k_r	k_{nr}
**2a**	2.33E+07	3.10E+08	2.78E+07	3.54E+07
**2b** (R^1 = Me)	2.33E+07	1.14E+09	3.36E+07	5.04E+07
**2c** (R^1 = Ph)	4.84E+07	3.55E+08	1.37E+07	5.48E+07
**2d** (R^1 = OMe)	-	-	3.19E+06	1.03E+08
**2e** (R^1 = OPh)	-	-	3.01E+07	3.84E+07
**2f** (R^1 = F)	2.05E+07	3.21E+08	4.30E+07	5.05E+07
**2g** (R^1 = Br)	1.73E+07	3.29E+08	8.33E+06	8.43E+07
**2h** (R^1 = CF_3)	2.27E+07	1.11E+09	-	-
**2i** (R^2 = 4-Me)	2.36E+07	1.07E+08	5.30E+07	2.16E+07
**2j** (R^2 = 5-Me)	3.68E+07	5.77E+08	1.81E+07	7.71E+07
**2k** (R^2 = 5-F)	-	-	1.90E+07	6.03E+07
**2l** (R^2 = 4,5-F,F)	2.65E+07	8.58E+08	2.07E+07	5.07E+07
**2o** (R^1=F, R^2=Me)	-	-	3.50E+07	9.97E+07

S14.1. HOMO-LUMO orbitals

Calculated HOMO–LUMO band gap (see **Figure S2** and **Table S4**).

Table S4. Highest occupied molecular orbital (HOMO), lowest unoccupied molecular orbital (LUMO), and band gap of the differently substituted compounds.

Compound	E_HOMO (eV)	E_LUMO (eV)	HOMO/LUMO (eV)
**2a**	-6.37	-2.47	3.90
**2b** (R^1 = Me)	-6.23	-2.40	3.83
**2c** (R^1 = Ph)	-5.94	-2.40	3.53
**2d** (R^1 = OMe)	-6.16	-2.46	3.70
**2e** (R^1 = OPh)	-6.12	-2.43	3.69
**2f** (R^1 = F)	-6.38	-2.58	3.81
**2g** (R^1 = Br)	-6.43	-2.68	3.75
**2h** (R^1 = CF_3)	-6.70	-2.77	3.92
**2i** (R^2 = 4-Me)	-6.27	-2.41	3.86
**2j** (R^2 = 5-Me)	-6.34	-2.39	3.95
**2k** (R^2 = 5-F)	-6.47	-2.74	3.73
**2l** (R^2 = 4,5-F,F)	-6.46	-2.58	3.88
**2o** (R^1 = F, R^2 = Me)	-6.00	-2.54	3.47

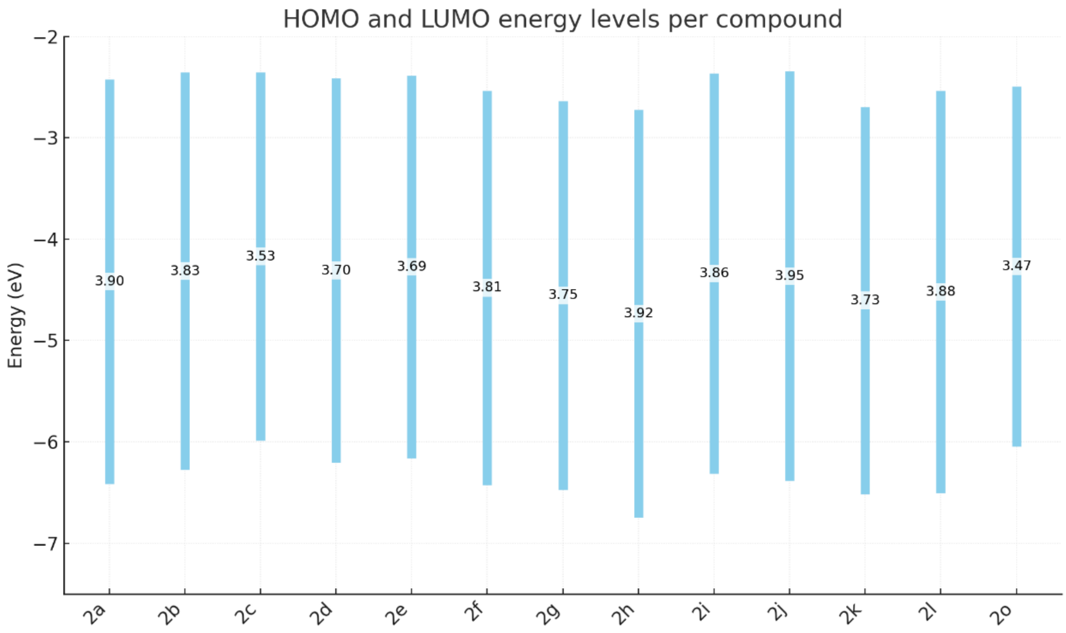

Figure S2. Graphical representation of the band gap of the differently substituted compounds.

HOMO and LUMO orbitals representations are attached to **Table S5**.

Table S5. Highest occupied molecular orbital (HOMO), lowest unoccupied molecular orbital (LUMO) graphic representations.

**2a**	**HOMO**	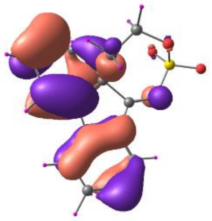
	**LUMO**	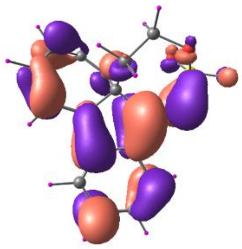

2b	HOMO	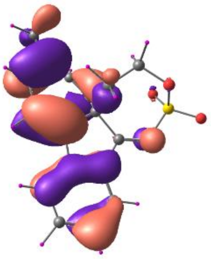
	LUMO	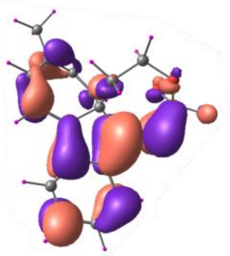
2c	HOMO	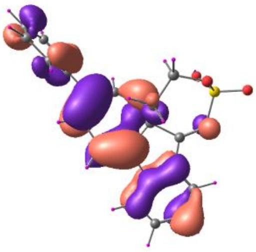
	LUMO	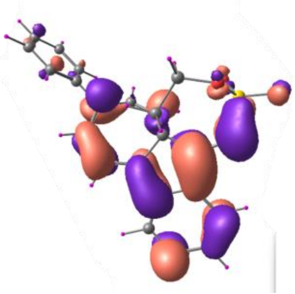
2d	HOMO	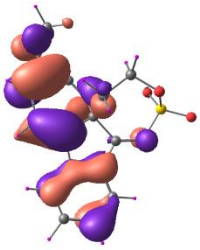
	LUMO	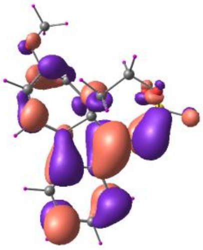

2e	HOMO	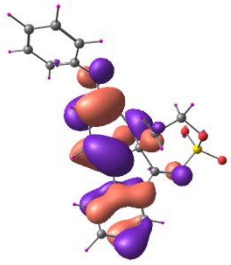
	LUMO	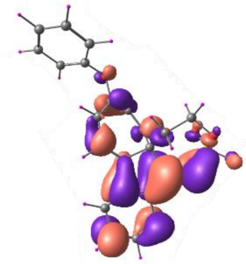
2f	HOMO	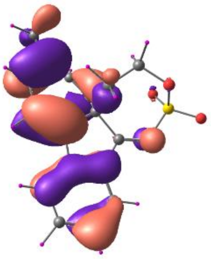
	LUMO	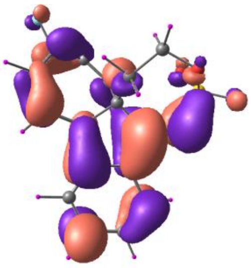
2g	HOMO	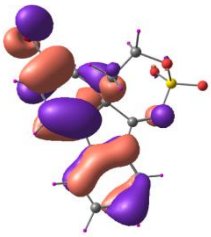
	LUMO	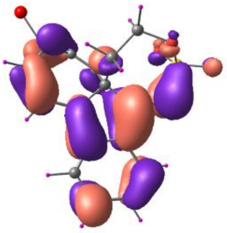

2h	HOMO	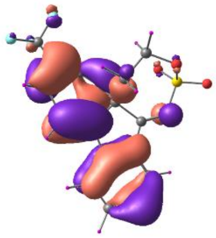
	LUMO	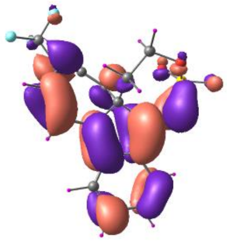
2i	HOMO	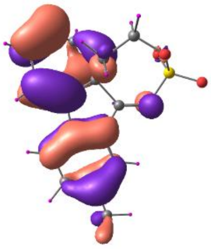
	LUMO	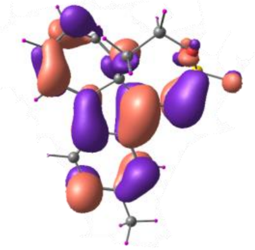
2j	HOMO	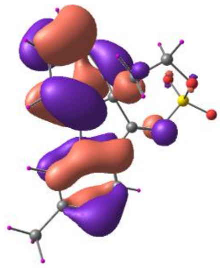
	LUMO	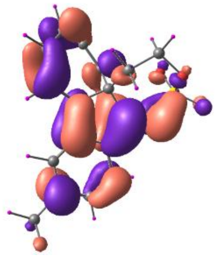

**2k**	**HOMO**	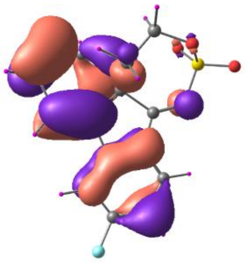
	**LUMO**	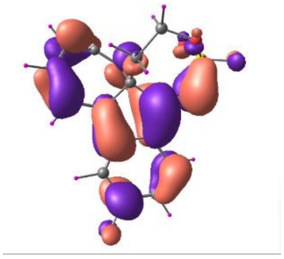
**2l**	**HOMO**	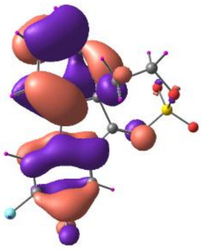
	**LUMO**	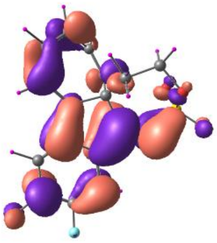
**2o**	**HOMO**	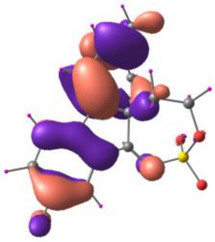
	**LUMO**	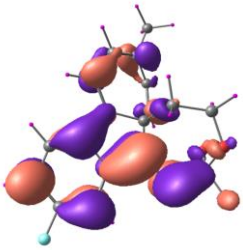

S15. References

[1] D. Pan, Y. Wei, M. Shi. *Org. Lett*. **2017**, *19*, 13, 3584–3587.

[2] E. Brunard, V. Boquet, T. Saget, E. D. S. Carrizo, M. Sircoglou, P. Dauban. *J. Am. Chem. Soc.* **2024**, *146*, 9, 5843–5854.

[3] B. J. Stokes, B. Jovanović, H. Dong, K. J. Richert, R. D. Riell, T. G. Driver. *J.Org. Chem.* **2009,** *74*, 3225-3228.

[4] Y. Cheng, T. Ding, Q. Li, J. Qu, Y. Kang. *Org. Lett*. **2023**, *25*, 2611-2615.

[5] J. Xi, Q. Dong, G. Liu, S. Wang, L. Chen, Z. Yao. *Synlett,* **2010**, 1674-1678.

[6] S. Parisien-Collette, C. Cruché, X. Abel-Snape, S. K. Collins. *Green Chem.* **2017**,*19*, 4798-4803.

[7] L. Gurskaya, L. Politanskaya, J. Wang, P. Ilyina, A. Volobueva, V. Zarubaev. *J. Flour. Chem.* **2024**, *274*.

[8] G. Shi, D. Chen, H. Jiang, Y. Zhang, Y. Zhang. *Org. Lett*. **2016**, *18*, 12, 2958 – 2961.

[9] B. Tan, L. Liu, H. Zheng, T. Cheng, D. Zhu, X. Yang, X. Luan. *Chem. Sci*. **2020**, *11*, 10198 – 10203.

[10] J. Li, H. Wang, J. Sun, Y. Yang, L. Liu. *Org. Biomol. Chem*. **2014**, *12*, 7904 – 7908.

[11] M. Jiang, J. Guo, B. Liu, Q. Tan, B. Xu. *Org. Lett.* **2019**, *21*, 20, 8328 – 8333.

[12] P. H. Patil, R. A. Fernandes. *RSC Adv.* **2015**, *5*, 54037 – 54045.

[13] M. Sguazzin, J. Johnson, J. Magolan. *Org. Lett*. **2021**, *23*, 9, 3373 – 3378.

[14] Q. Jiang, D. Duan-Mu, W. Zhong, H. Chen, H. Yan. *Chem. Eur. J*. **2013**, *19*, 1903-1907.

[15] M. Sun, X. Chen, Z. Feng, G. Deng, Y. Yang, Y. Lian. *Org. Chem. Front.* **2021**, *8*, 6535-6540.

[16] Gaussian 16, Revision C.01: M. J. Frisch, G. W. Trucks, H. B. Schlegel, G. E. Scuseria, M. A. Robb, J. R. Cheeseman, G. Scalmani, V. Barone, G. A. Petersson, H. Nakatsuji, X. Li, M. Caricato, A. V. Marenich, J. Bloino, B. G. Janesko, R. Gomperts, B. Mennucci, H. P. Hratchian, J. V. Ortiz, A. F. Izmaylov, J. L. Sonnenberg, Williams, F. Ding, F. Lipparini, F. Egidi, J. Goings, B. Peng, A. Petrone, T. Henderson, D. Ranasinghe, V. G. Zakrzewski, J. Gao, N. Rega, G. Zheng, W. Liang, M. Hada, M. Ehara, K. Toyota, R. Fukuda, J. Hasegawa, M. Ishida, T. Nakajima, Y. Honda, O. Kitao, H. Nakai, T. Vreven, K. Throssell, J. A. Montgomery Jr., J. E. Peralta, F. Ogliaro, M. J. Bearpark, J. J. Heyd, E. N. Brothers, K. N. Kudin, V. N. Staroverov, T. A. Keith, R. Kobayashi, J. Normand,

K. Raghavachari, A. P. Rendell, J. C. Burant, S. S. Iyengar, J. Tomasi, M. Cossi, J. M. Millam, M. Klene, C. Adamo, R. Cammi, J. W. Ochterski, R. L. Martin, K. Morokuma, O. Farkas, J. B. Foresman and D. J. Fox, Gaussian, Inc., Wallingford CT, **2016**.

[17] Y. Zhao, D. G. Truhlar. *Theor. Chem. Acc.* **2008**, *120*, 41-215.

[18] S. Grimme, J. Antony, S. Ehrlich, H. Krieg. *J. Chem. Phys.* **2010**, *132*, 154104.

[19] a) A. D. McLean, G. S. Chandler. *J. Chem. Phys.* **1980**, *72*, 48-5639. b) K. Raghavachari, J. S. Binkley, R. Seeger, J. A. Pople, *J. Chem. Phys.* **1980**, *72,* 54-650. c) K. Raghavachari, G. W. Trucks. *J. Chem. Phys.* **1989**, *91*, 65-1062.

[20] T. Clark, J. Chandrasekhar, G. W. Spitznagel, P. v. R. Schleyer. *J. Comp. Chem.*, **1983**, *4*, 294-301.

[21] M. J. Frisch, J. A. Pople, J. S. Binkley, *J. Chem. Phys.*, **1984**, *80,* 69-3265.

[22] M. Dolg, H. Stoll, H. Preuss, R. M. Pitzer. *J. Phys. Chem.* **1993**, *97*, 22, 5852–5859.

[23] A.D. Becke. *J. Chem. Phys.* **1993**, *98*, 5648–5652.

[24] R. Ditchfield, W. J. Hehre, J. A. Pople. *J. Chem. Phys.* **1971,** *54*, 724–728.

[25] A.V. Marenich, C.J. Cramer, D.G. Truhlar. *J. Phys. Chem.* **2009**, *113*, 6378-6396.

[26] W. Li, W. Chen, B. Zhou, Y. Xu, G. Deng, Y. Yang, Y. Liang. *Org. Lett*. **2019**, 21, 8, 2718-2722.

S16. NMR spectra

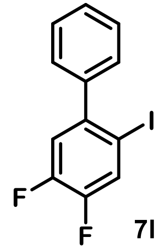

7l

^{1}H NMR (400 MHz, $CDCl_3$)

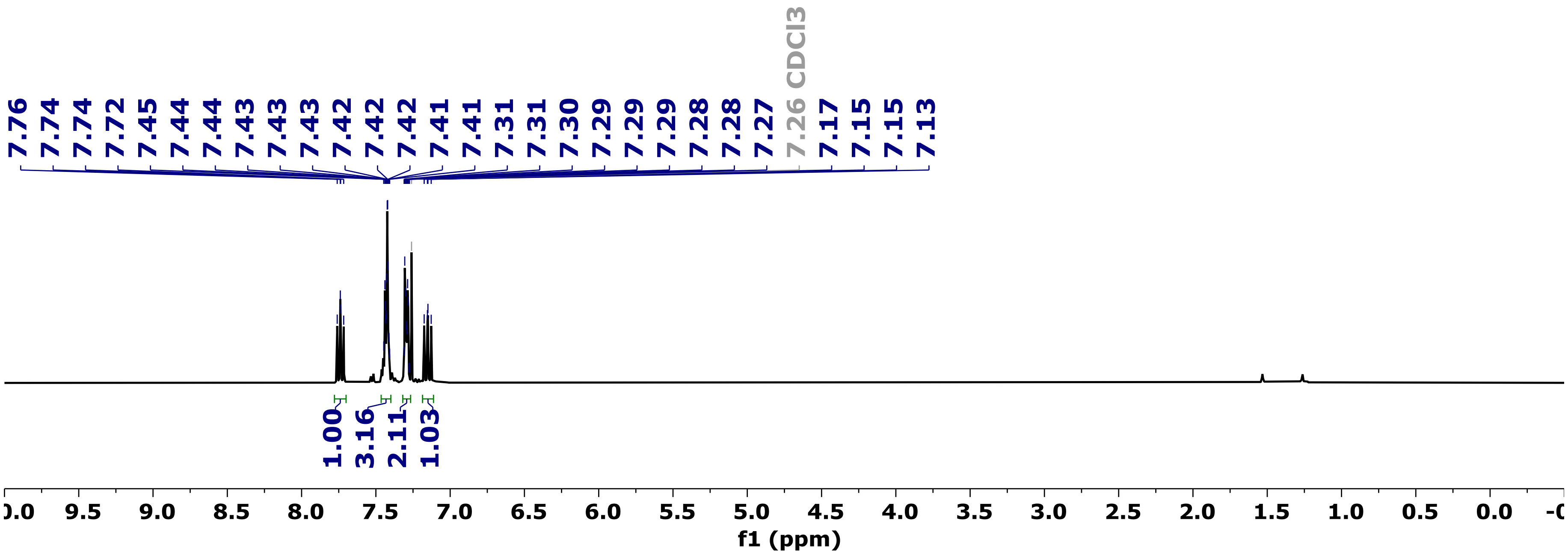

$^{13}C\{H\}$ NMR (101 MHz, $CDCl_3$)

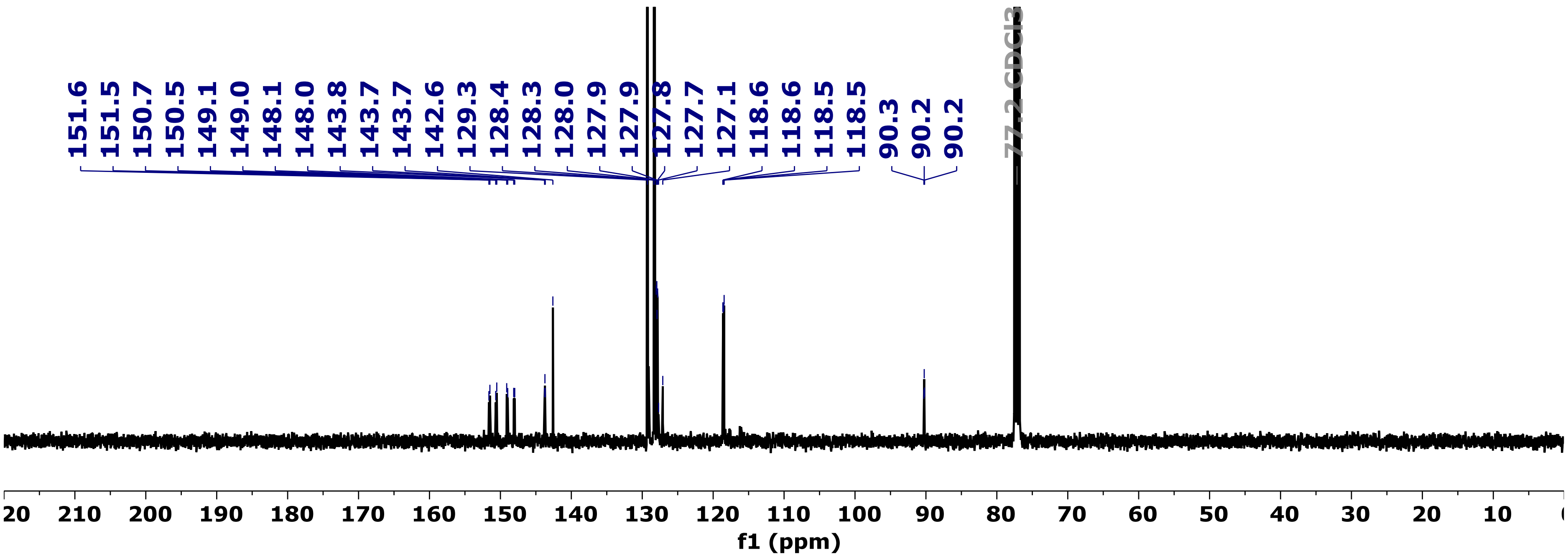

^{19}F NMR (377 MHz, $CDCl_3$)

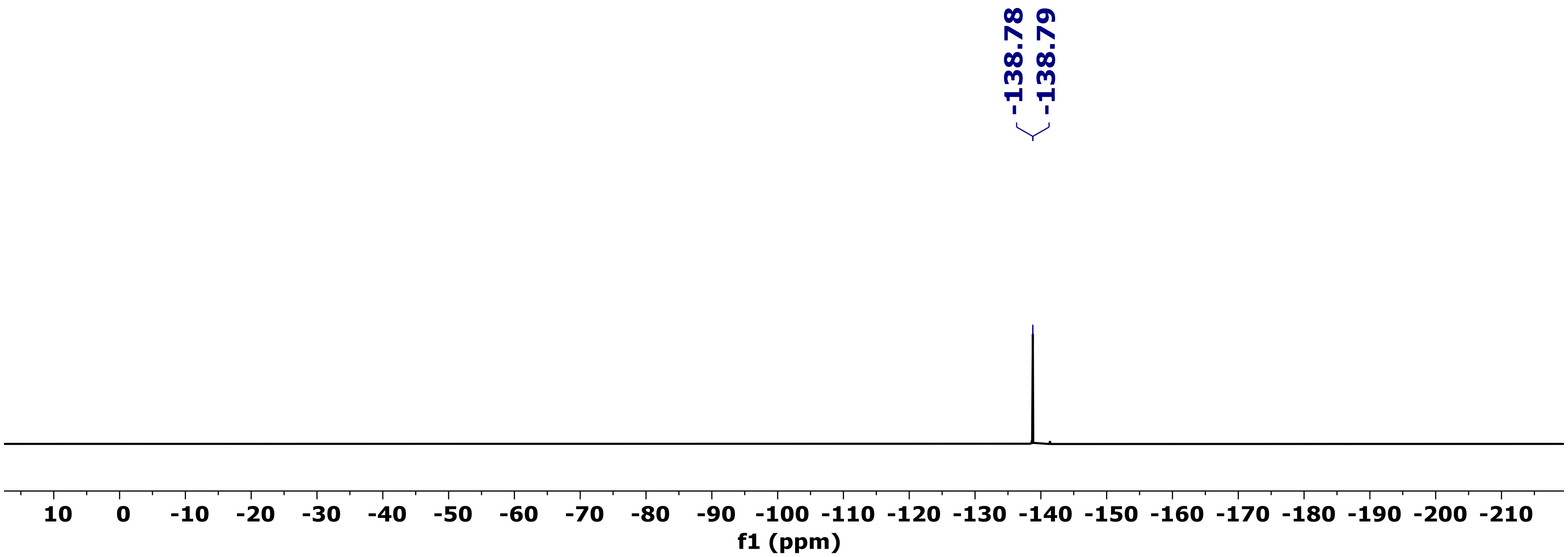

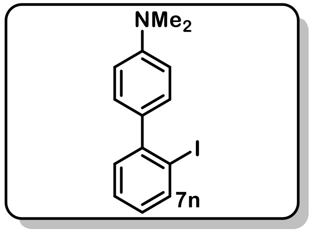

^{1}H NMR (400 MHz, $CDCl_3$)

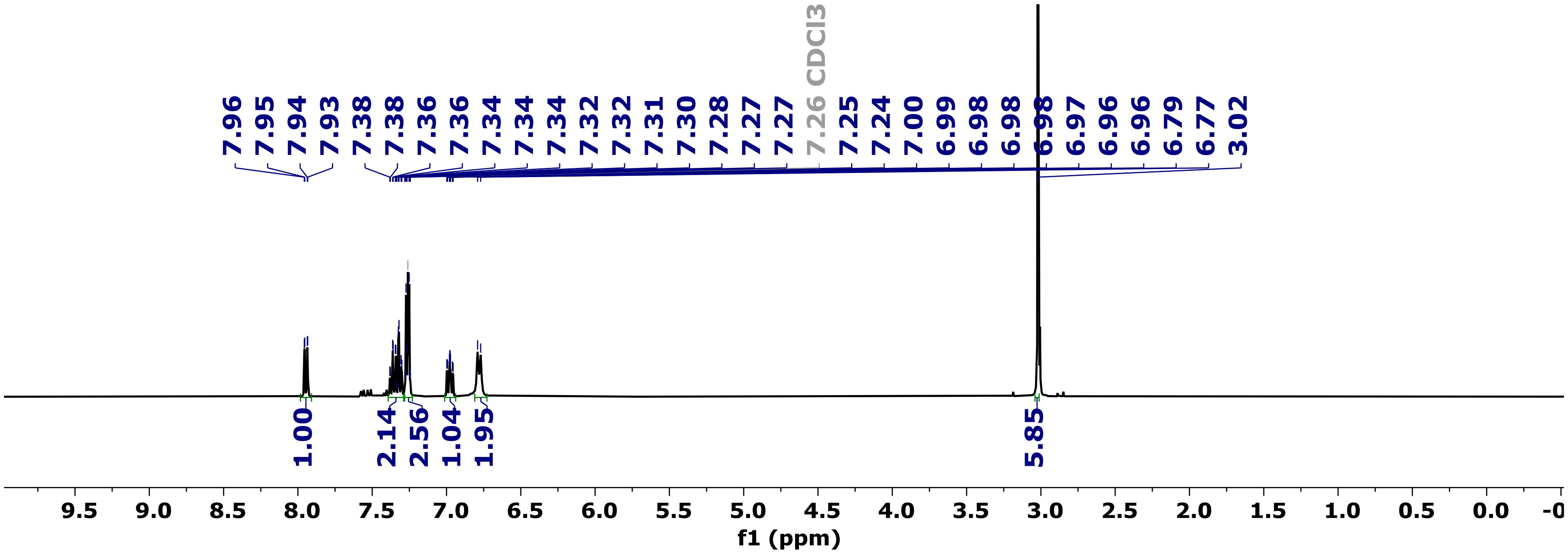

$^{13}C\{H\}$ NMR (101 MHz, $CDCl_3$)

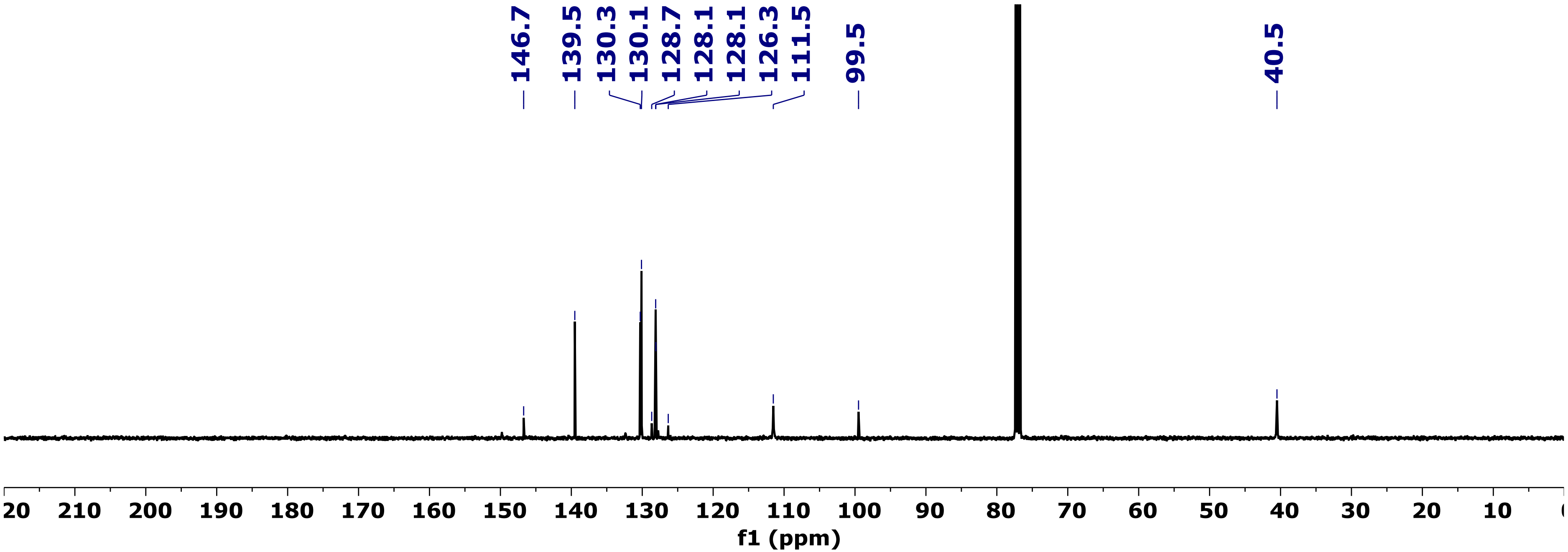

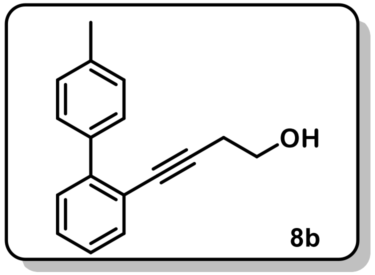

^{1}H NMR (400 MHz, $CDCl_3$)

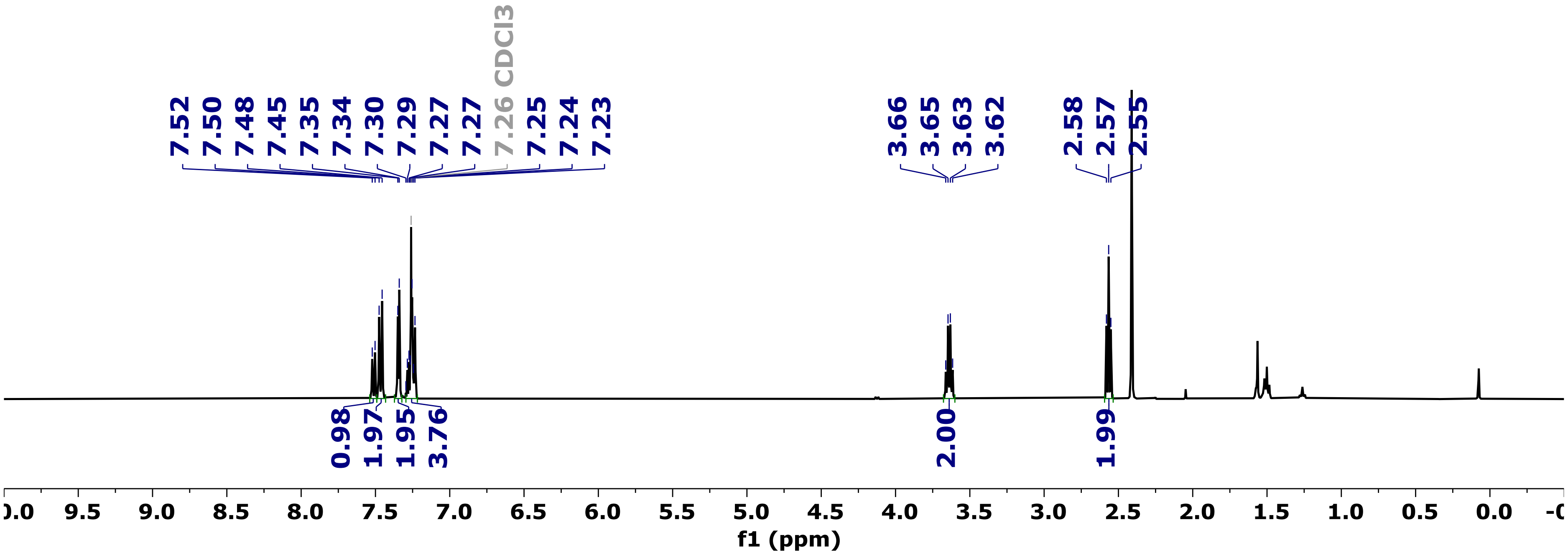

$^{13}C\{H\}$ NMR (101 MHz, $CDCl_3$)

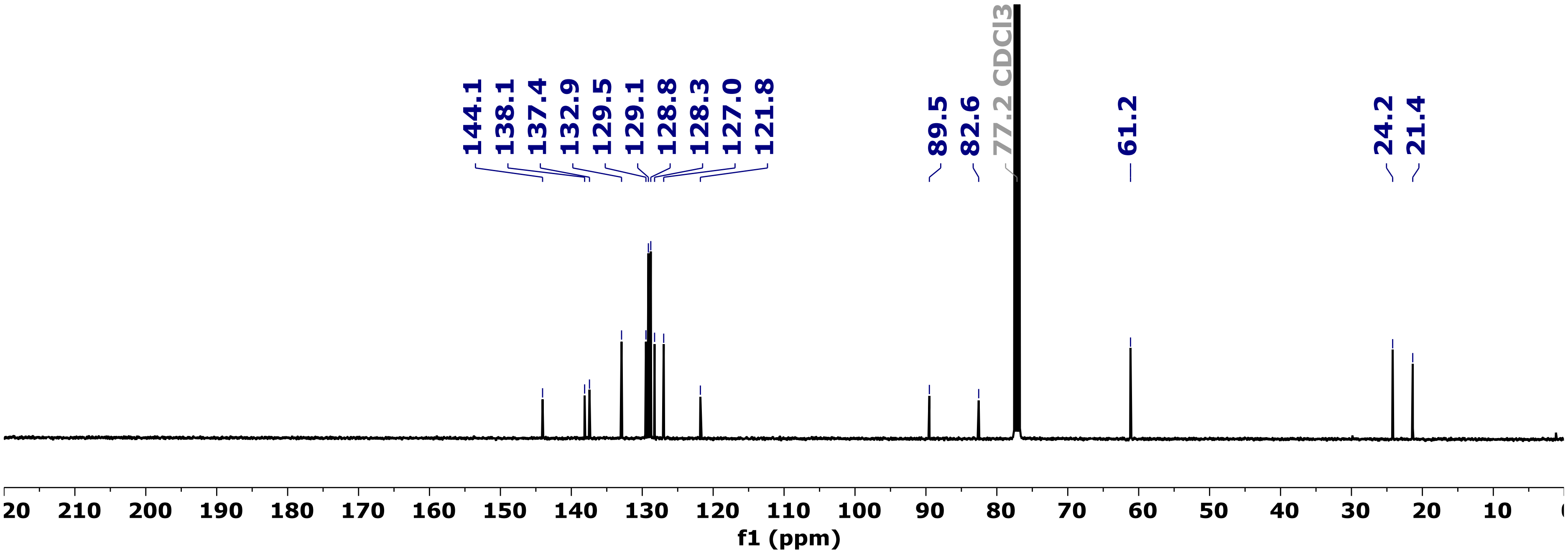

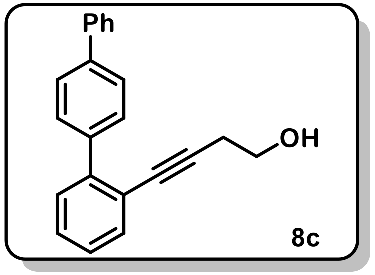

^1H NMR (400 MHz, $CDCl_3$)

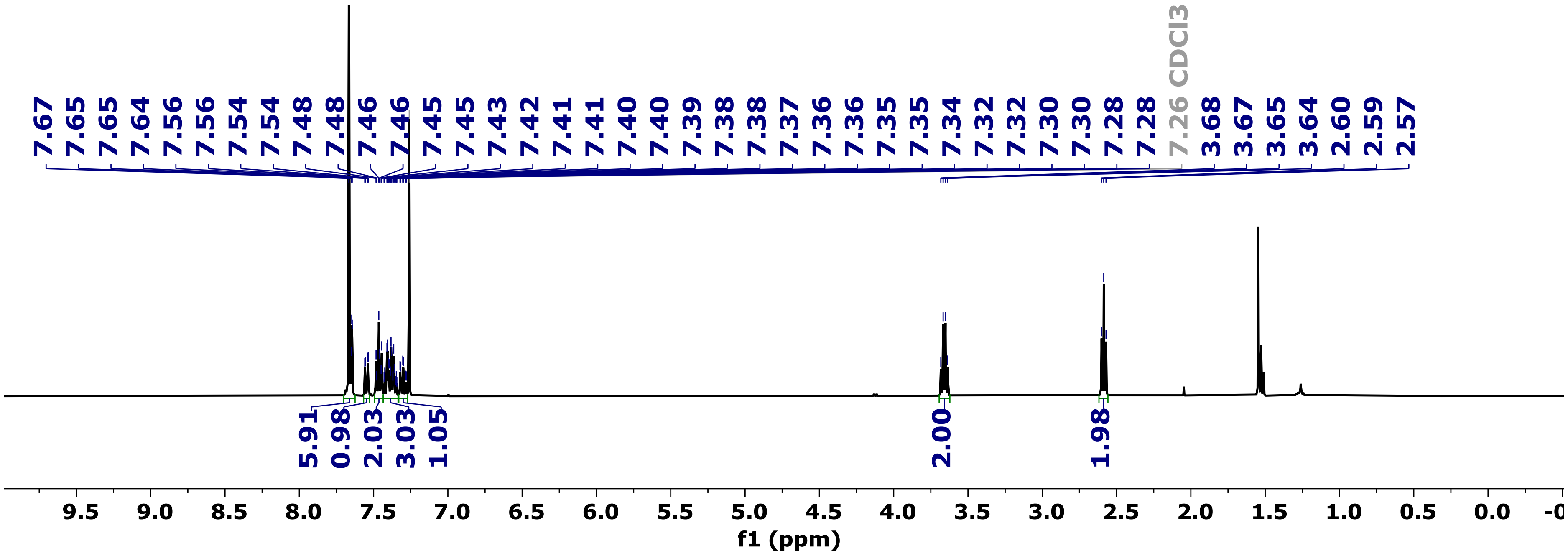

13C{H} NMR (101 MHz, CDCl3)

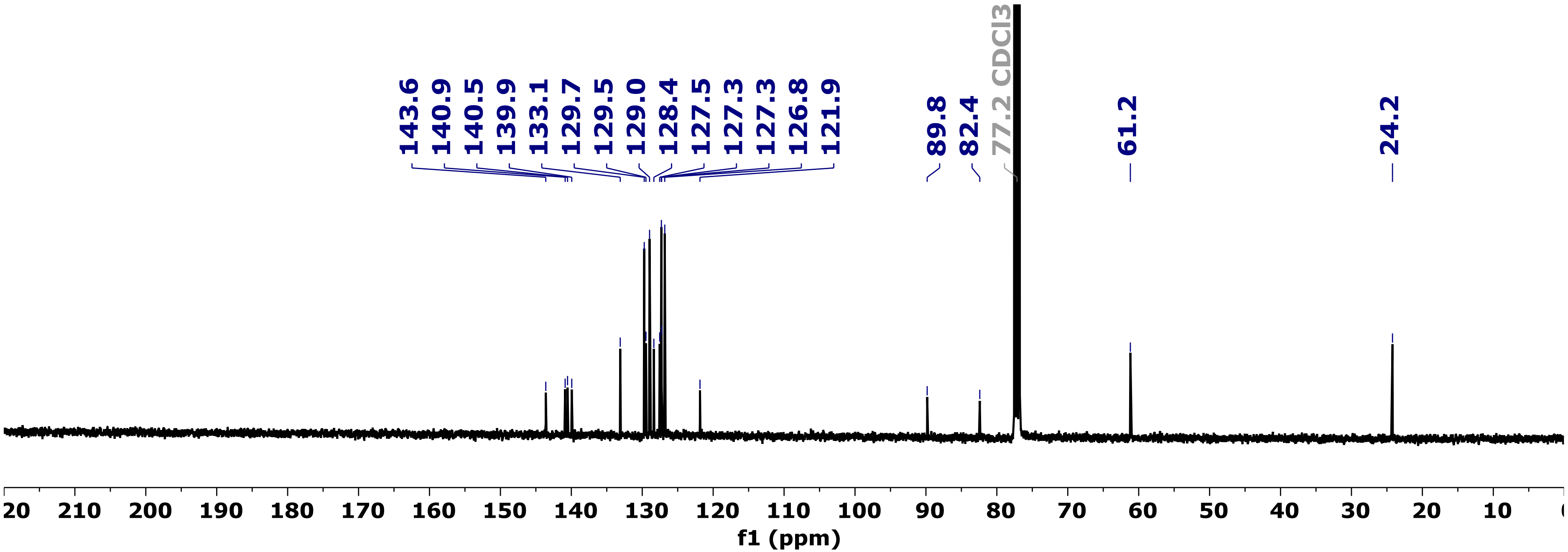

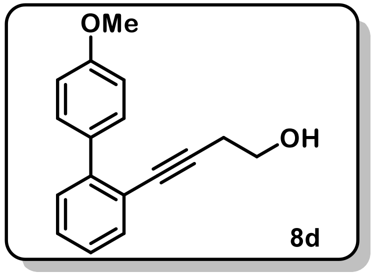

^{1}H NMR (400 MHz, $CDCl_3$)

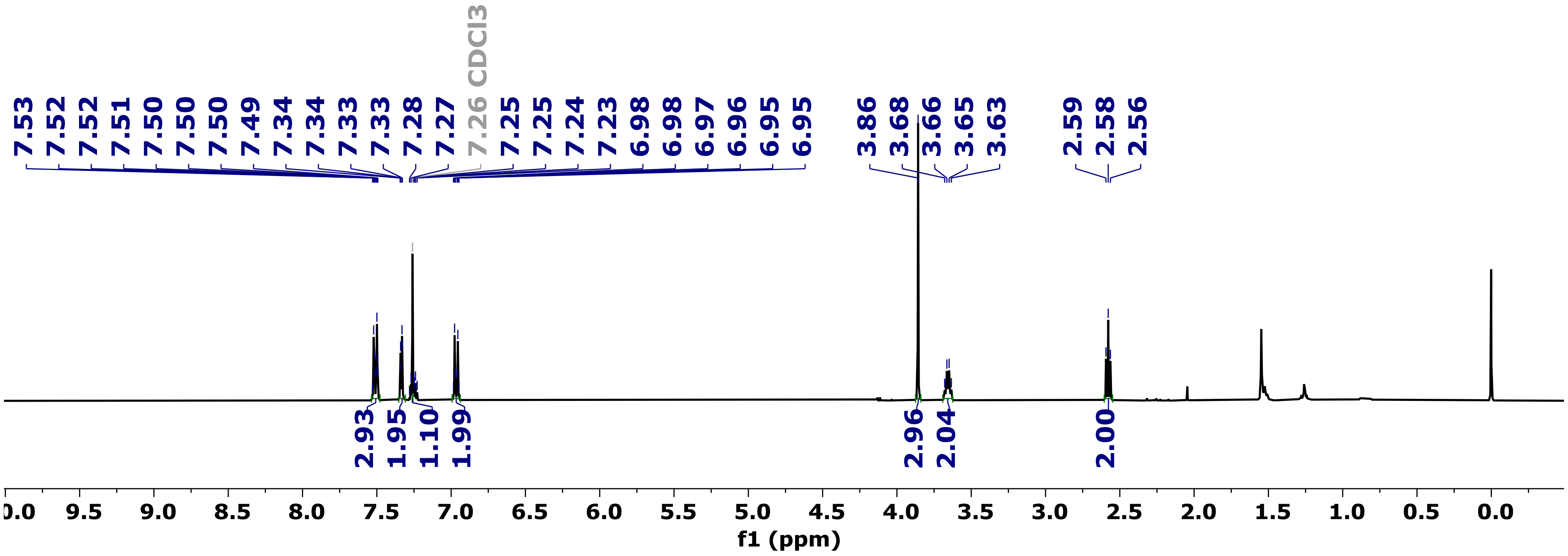

$^{13}C\{H\}$ NMR (101 MHz, $CDCl_3$)

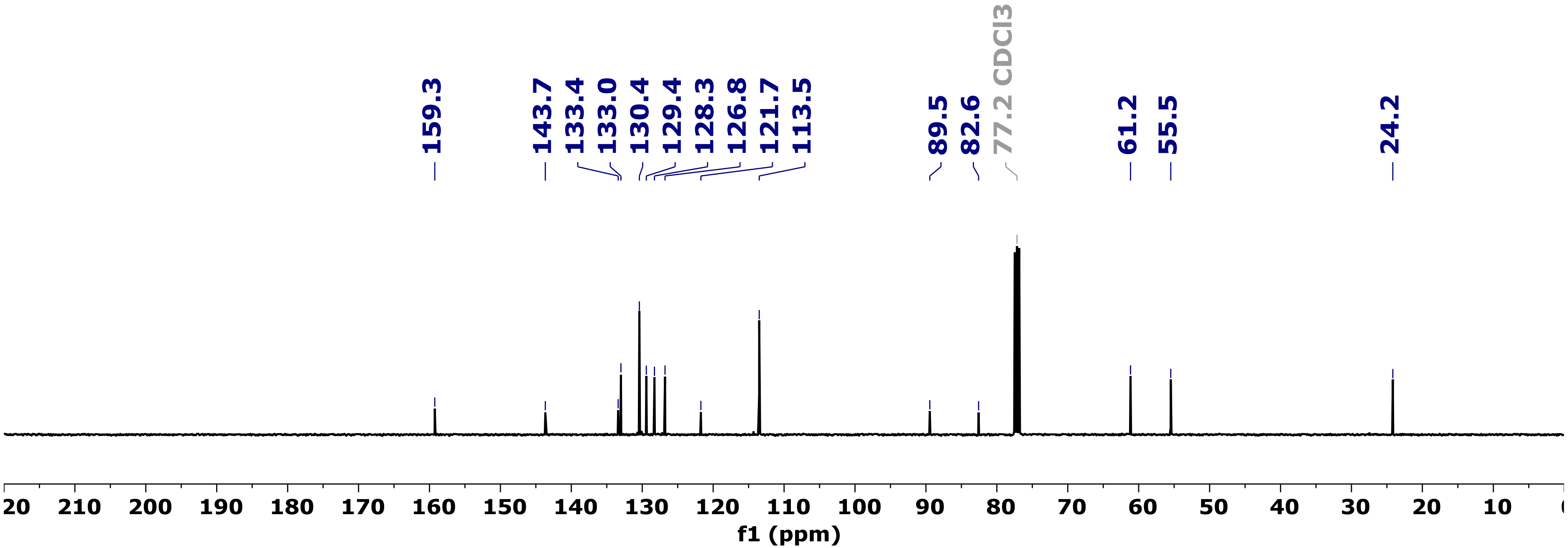

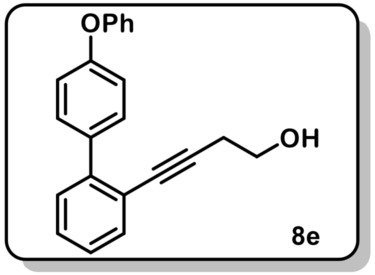

^{1}H NMR (400 MHz, $CDCl_3$)

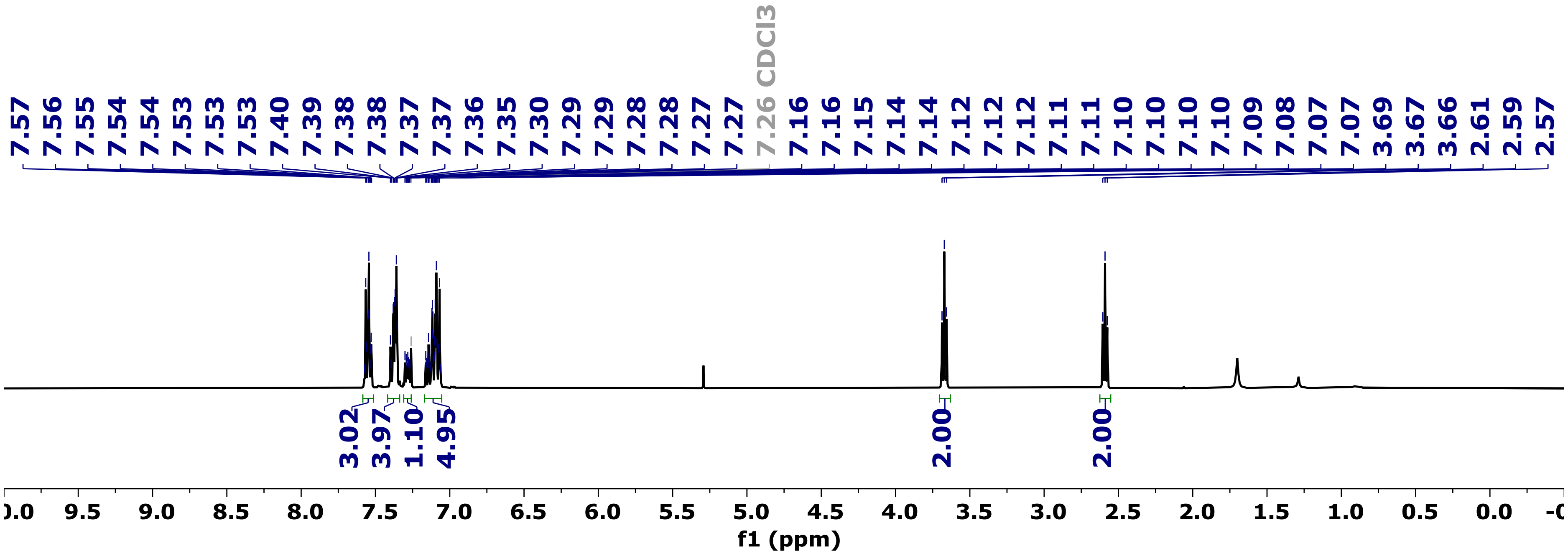

$^{13}C\{H\}$ NMR (101 MHz, $CDCl_3$)

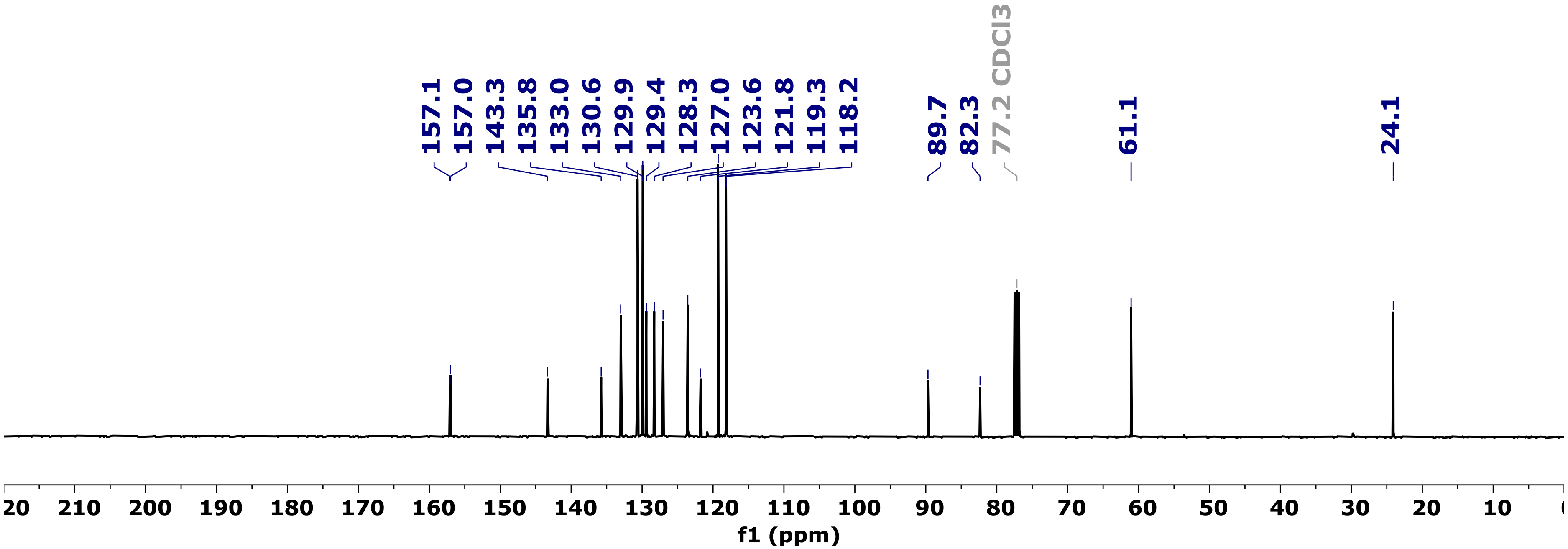

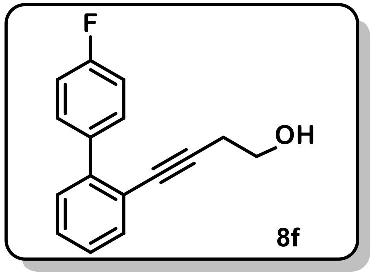

^{1}H NMR (400 MHz, $CDCl_3$)

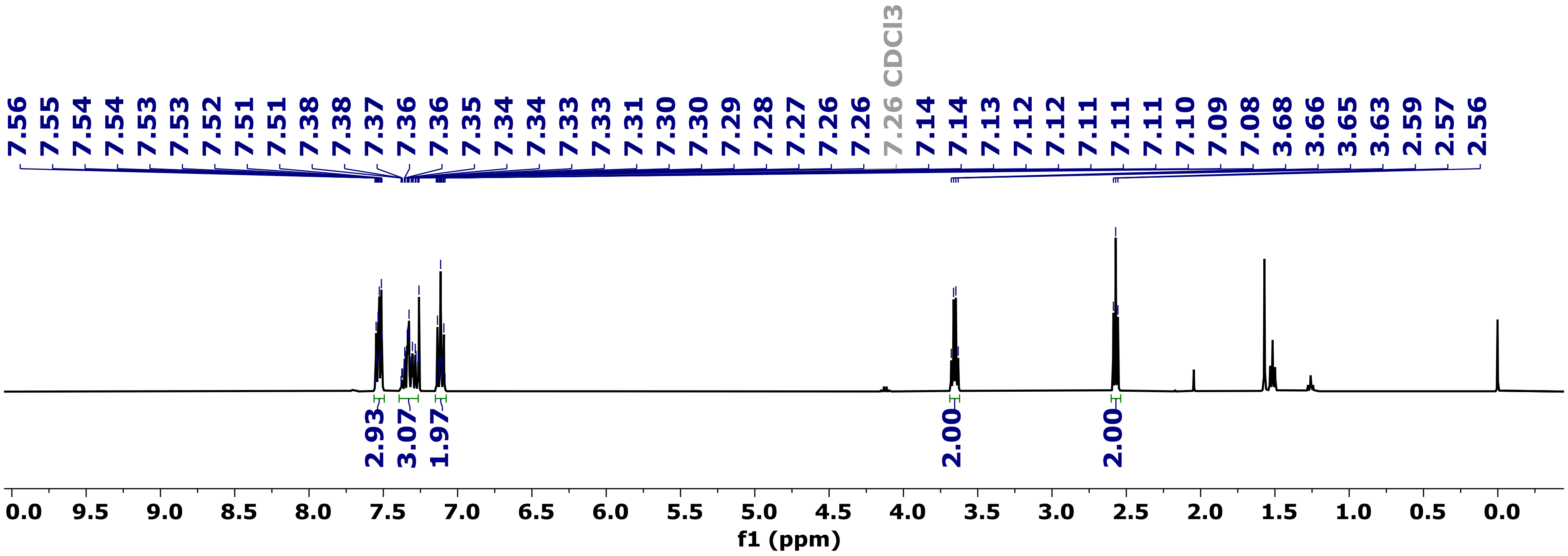

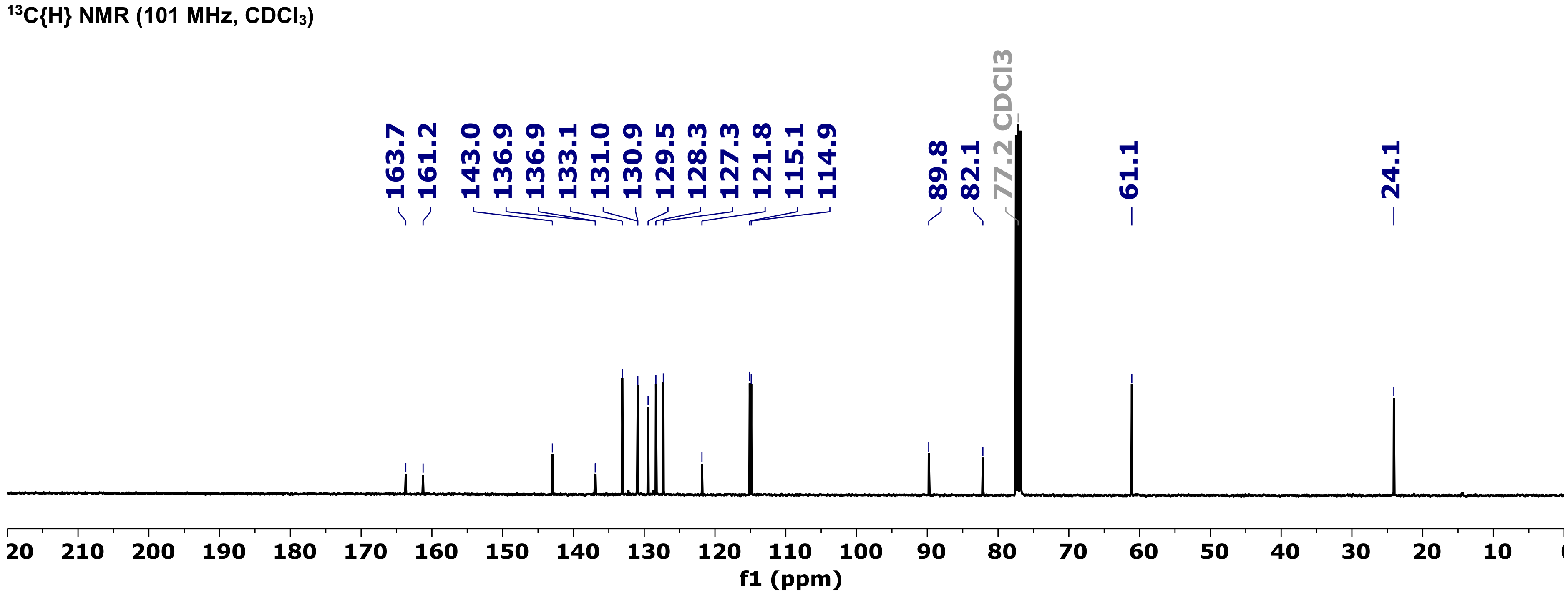

$^{13}C\{H\}$ NMR (101 MHz, $CDCl_3$)
163.7
161.2
143.0
136.9
136.9
133.1
131.0
130.9
129.5
128.3
127.3
121.8
115.1
114.9
89.8
82.1
77.2 CDCl3
61.1
24.1
f1 (ppm)

^{19}F NMR (377 MHz, $CDCl_3$)

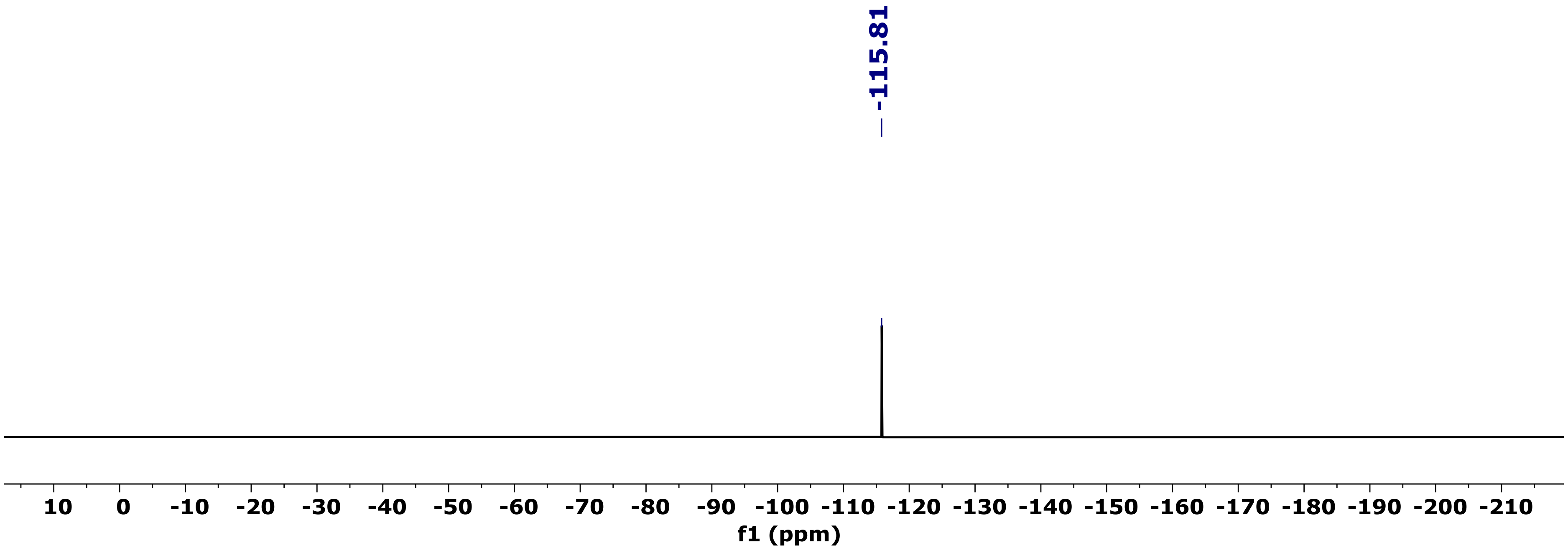

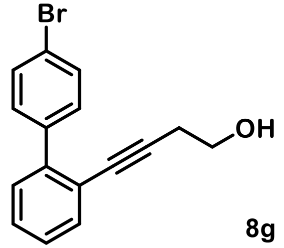

8g

^{1}H NMR (400 MHz, $CDCl_3$)

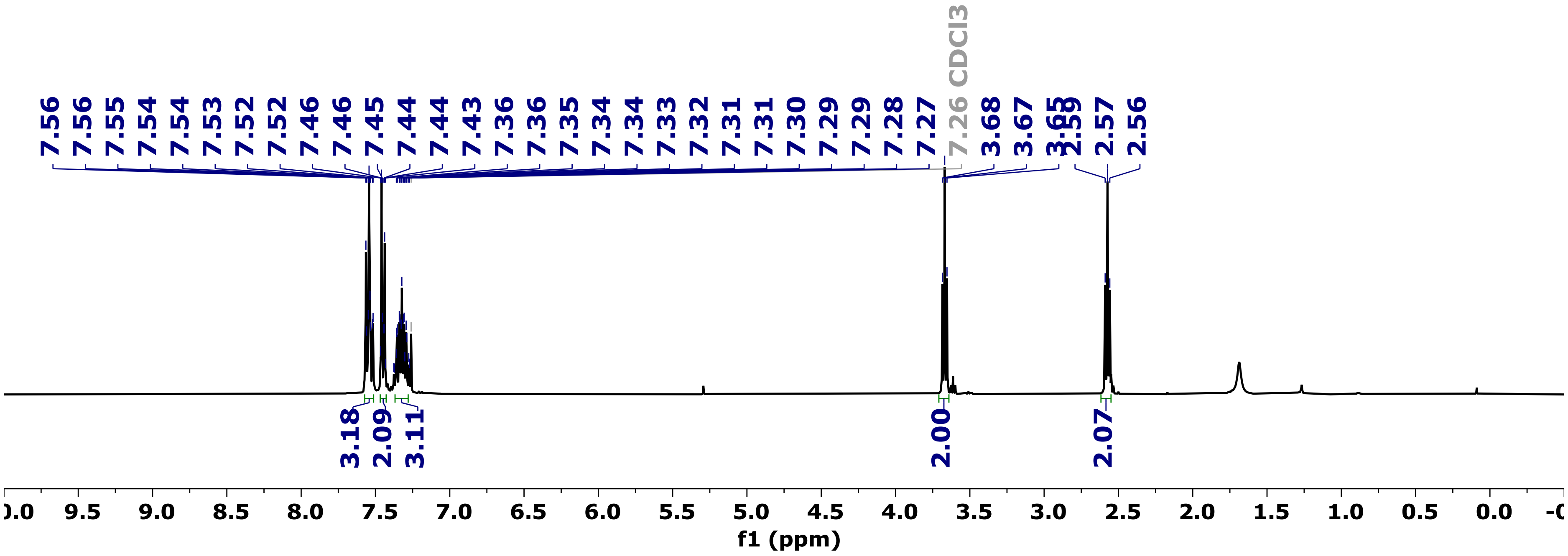

$^{13}C\{H\}$ NMR (101 MHz, $CDCl_3$)

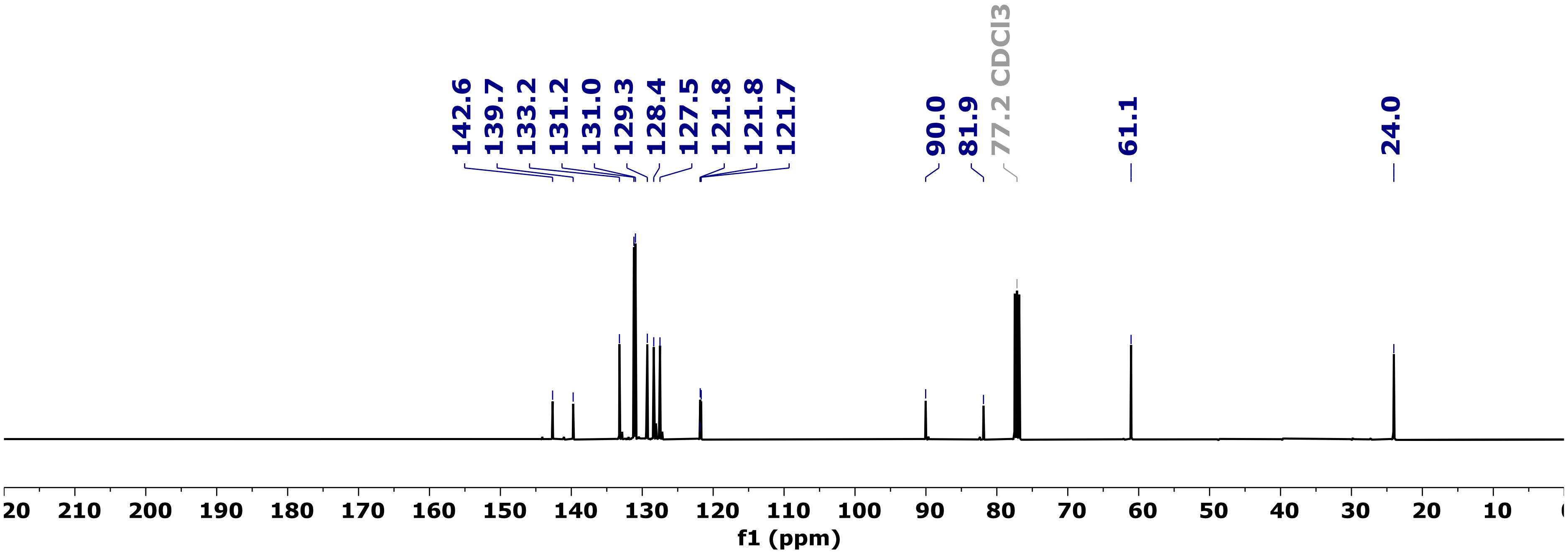

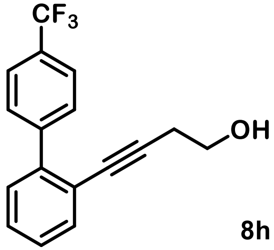

8h

^{1}H NMR (400 MHz, $CDCl_3$)

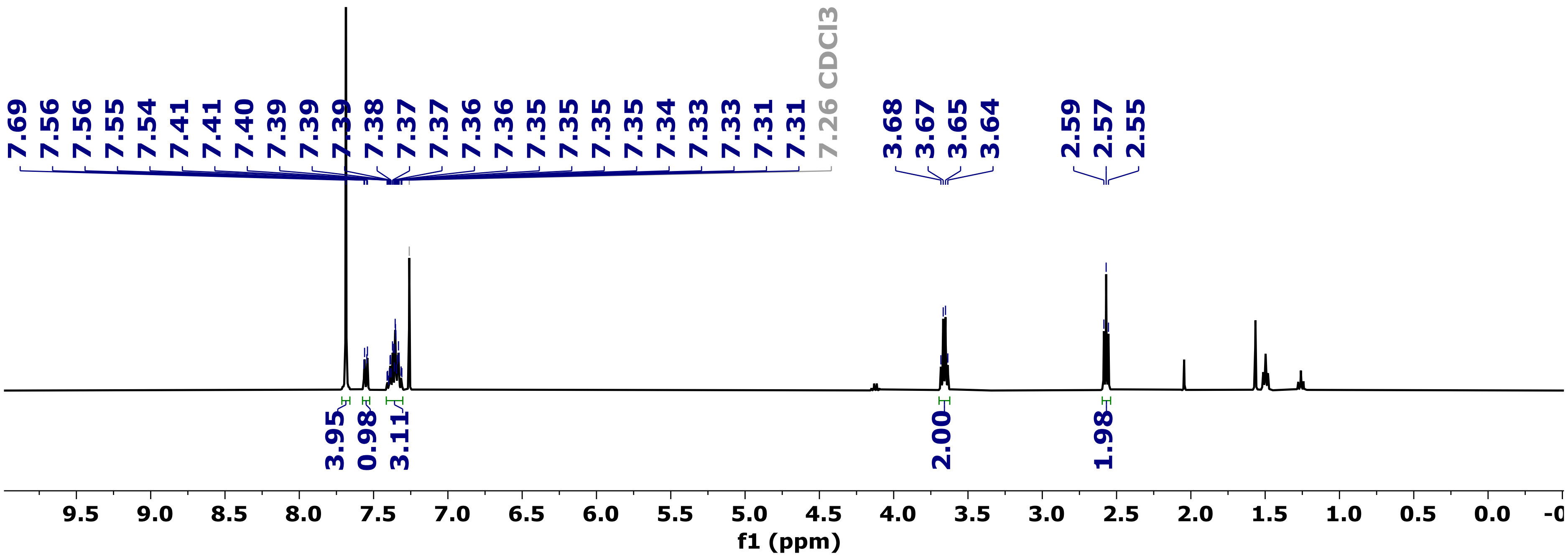

$^{13}C\{H\}$ NMR (101 MHz, $CDCl_3$)

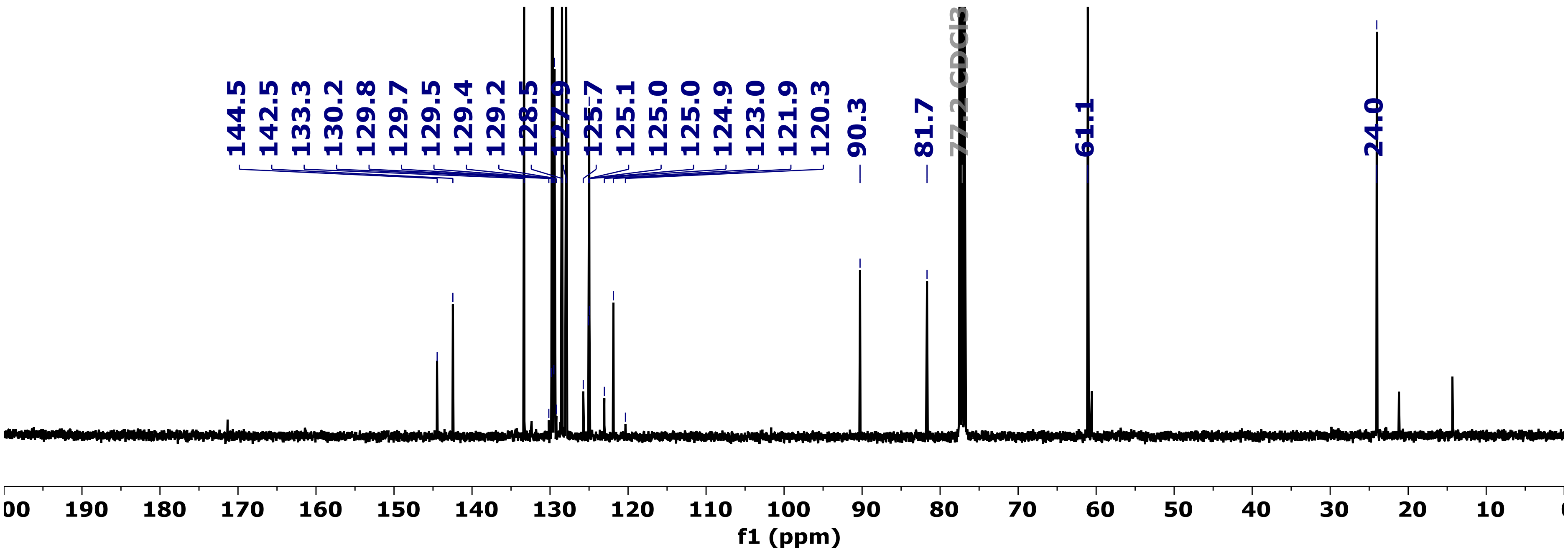

^{19}F NMR (377 MHz, $CDCl_3$)

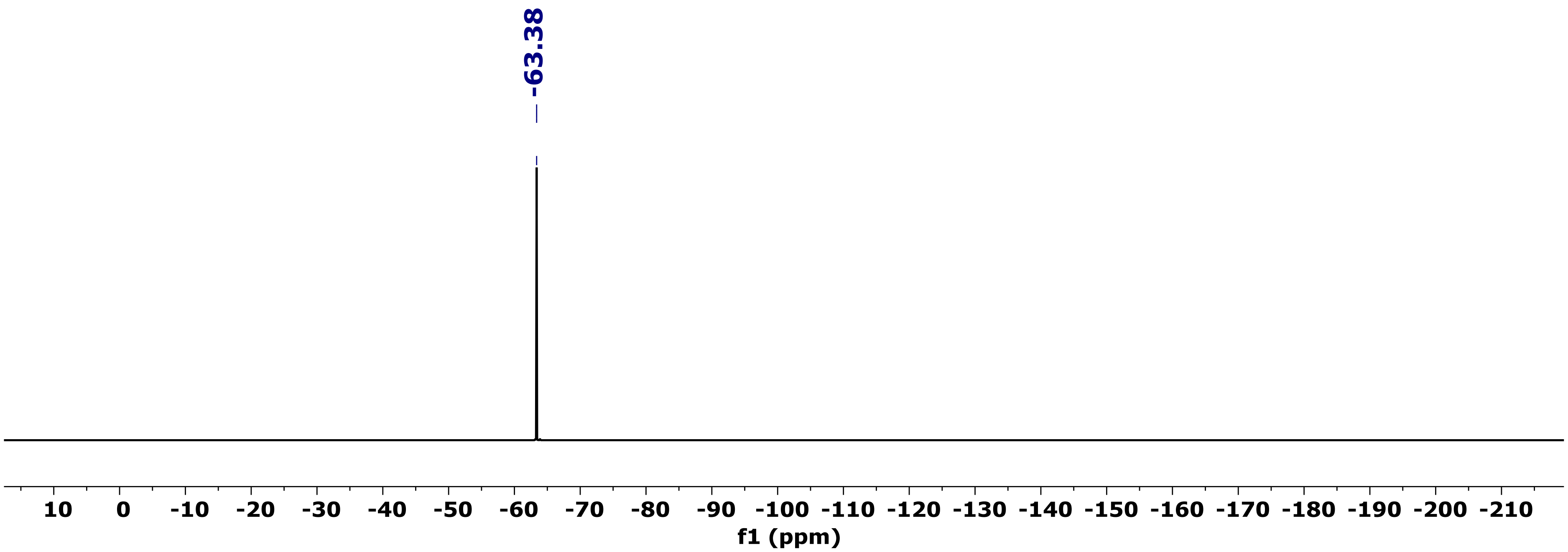

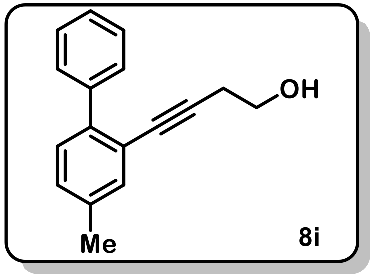

8i

^{1}H NMR (400 MHz, $CDCl_3$)

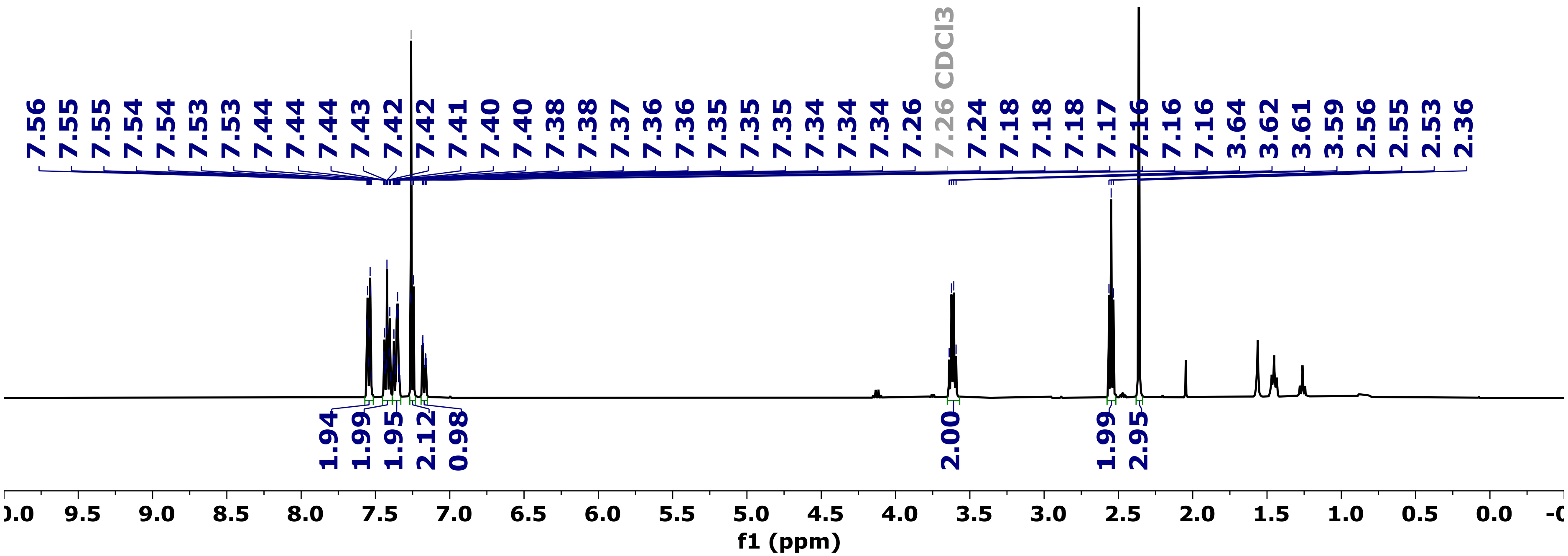

$^{13}C\{H\}$ NMR (101 MHz, $CDCl_3$)

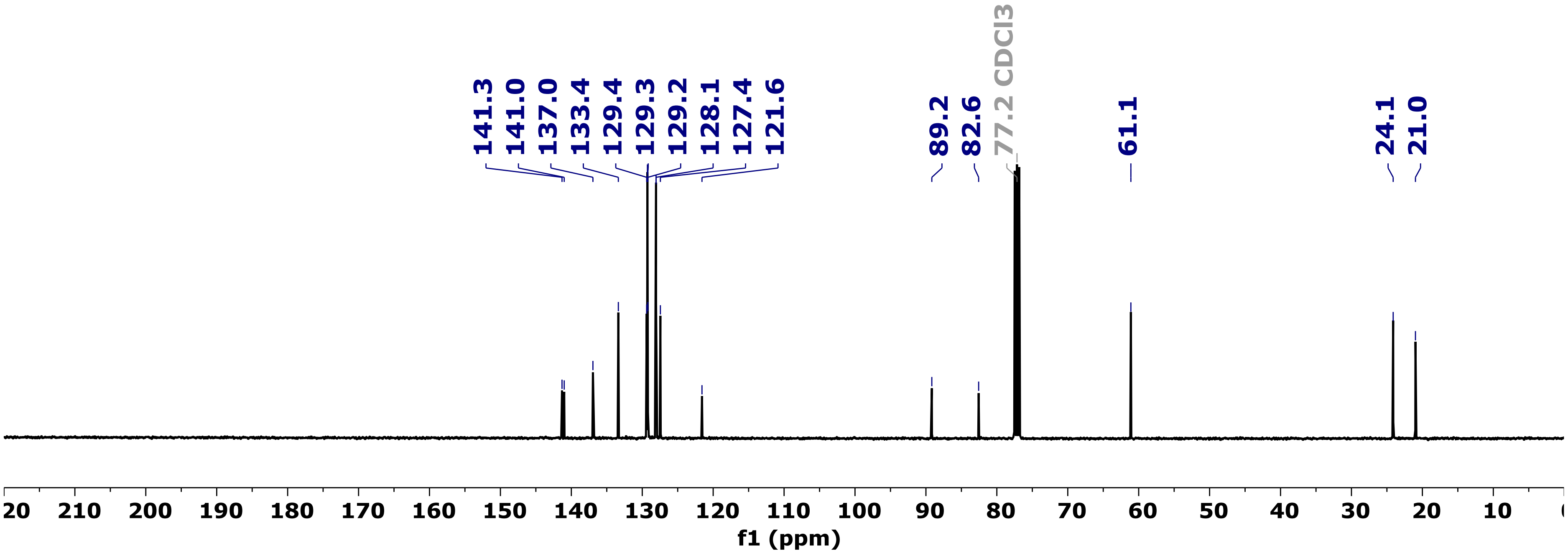

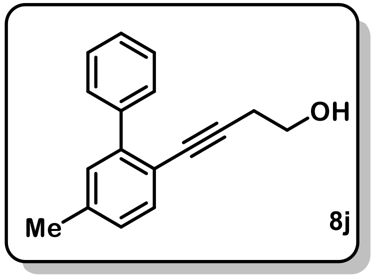

8j

^{1}H NMR (400 MHz, $CDCl_3$)

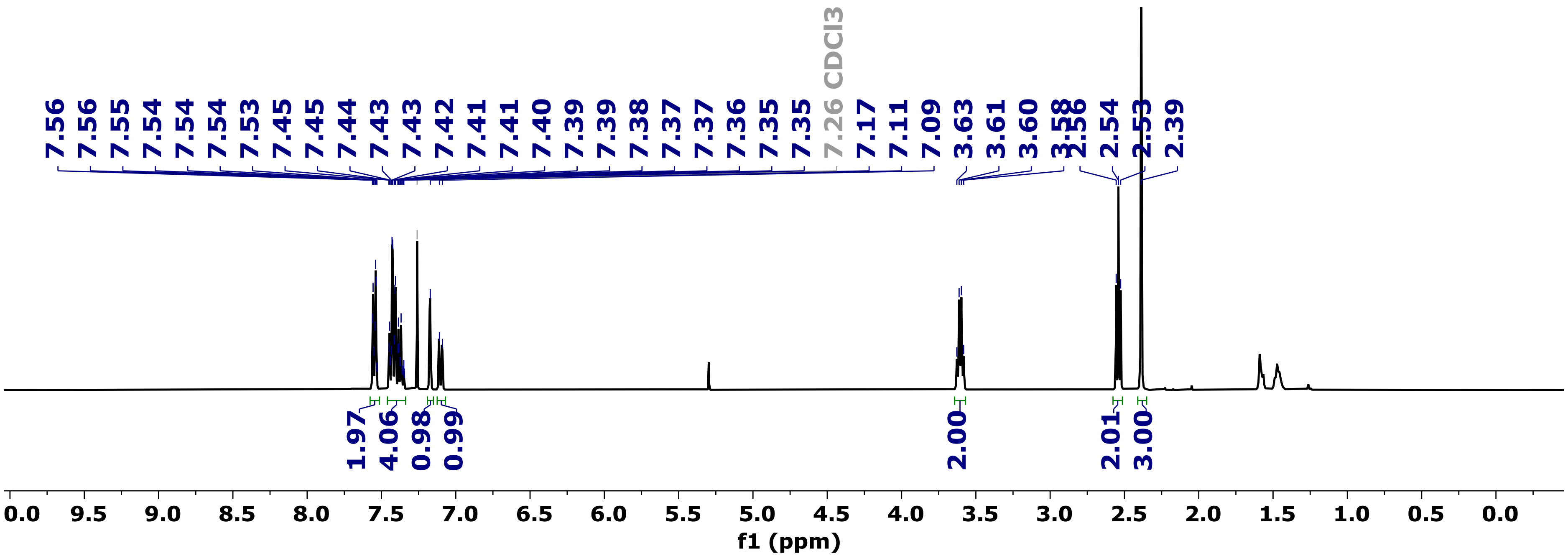

$^{13}C\{H\}$ NMR (101 MHz, $CDCl_3$)

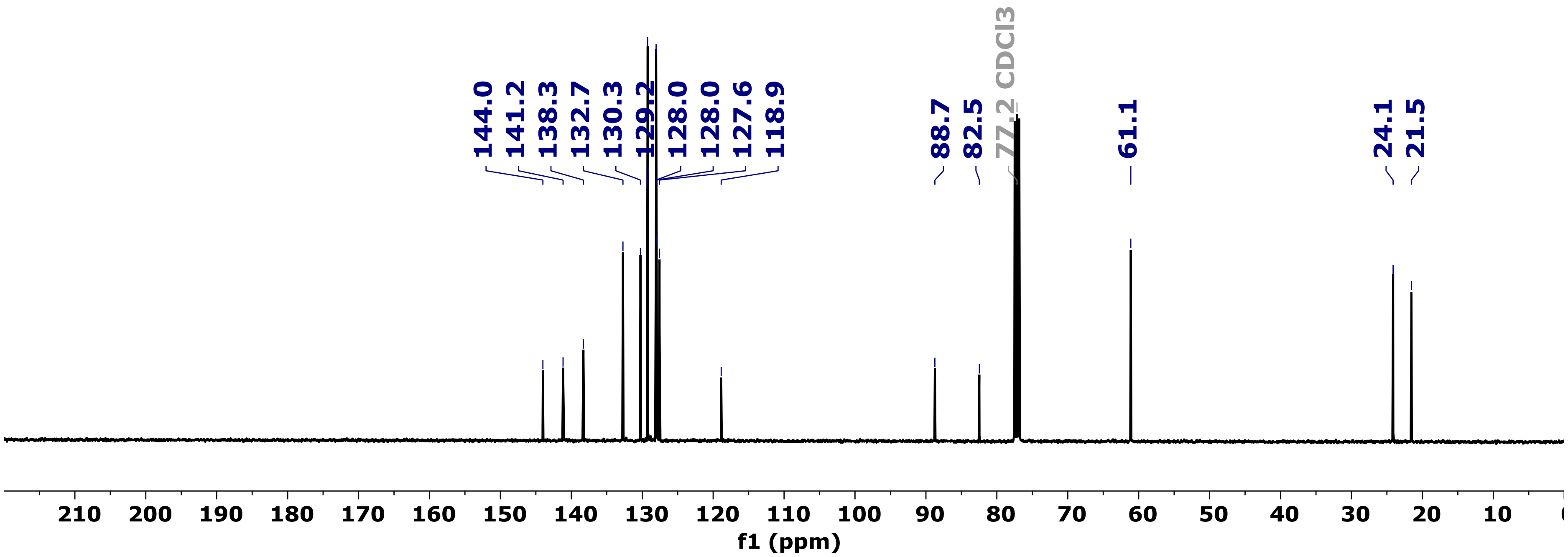

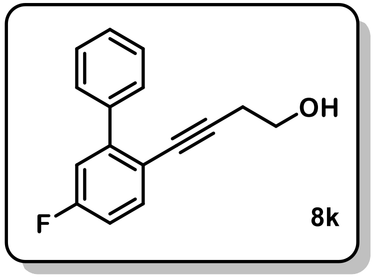

^{1}H NMR (400 MHz, $CDCl_3$)

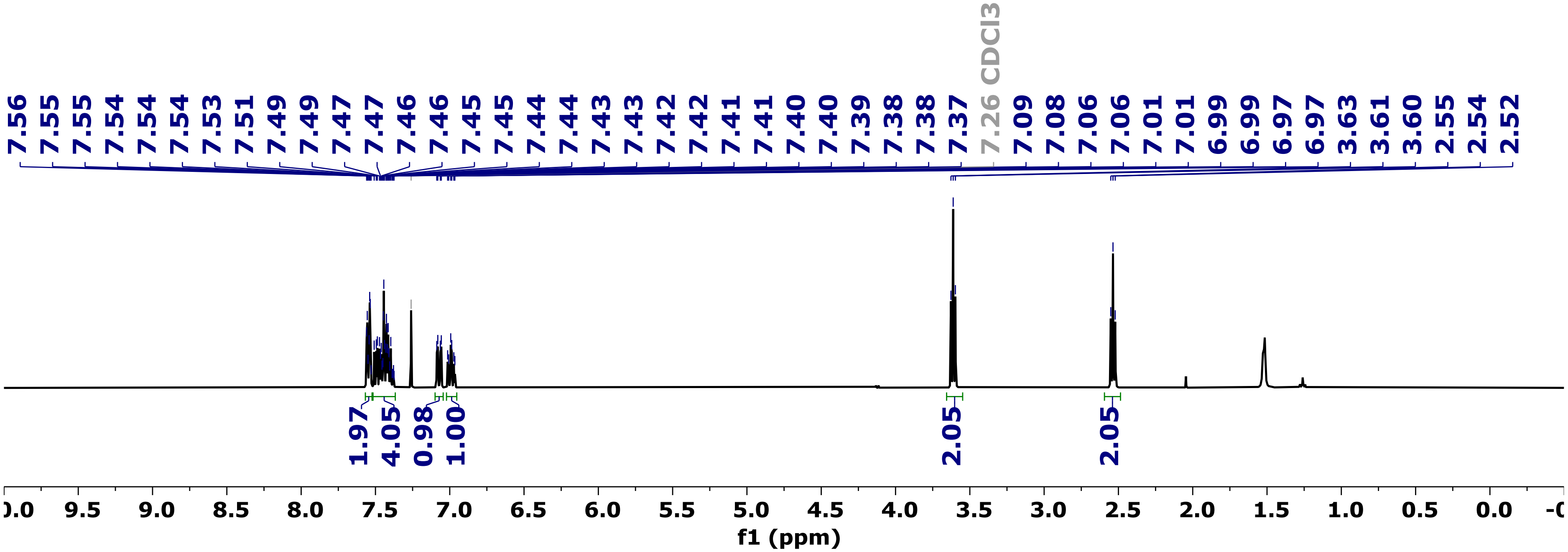

$^{13}C\{H\}$ NMR (101 MHz, $CDCl_3$)

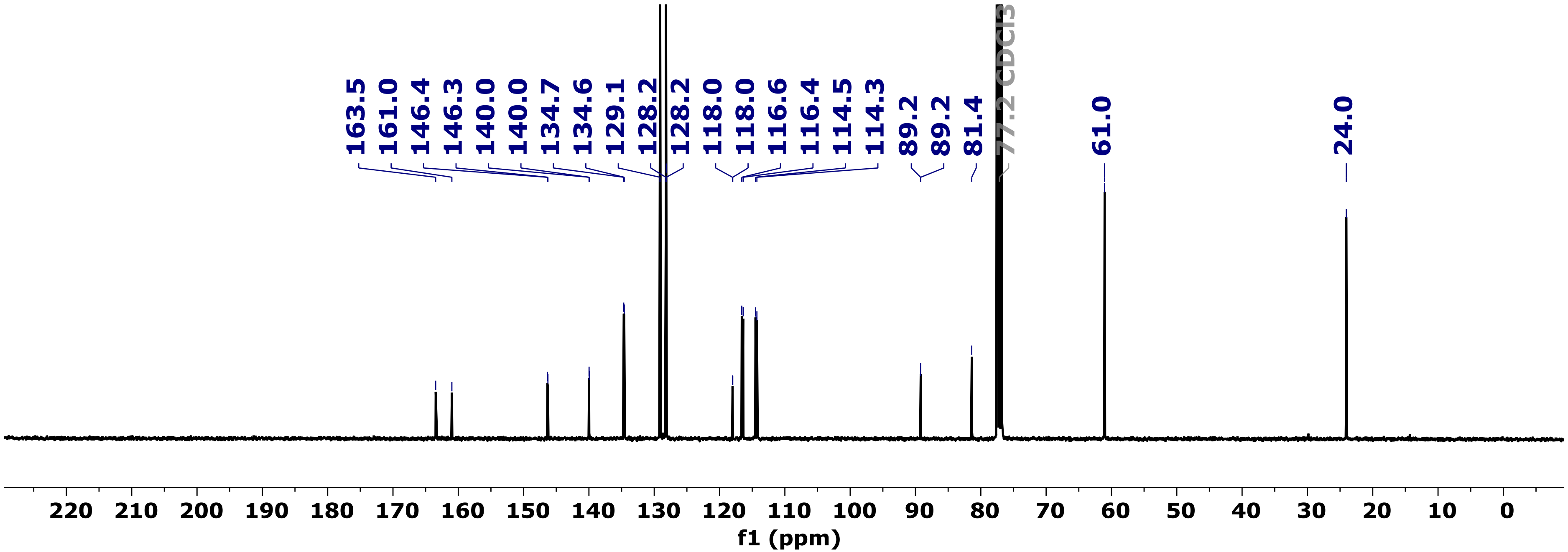

^{19}F NMR (377 MHz, $CDCl_3$)

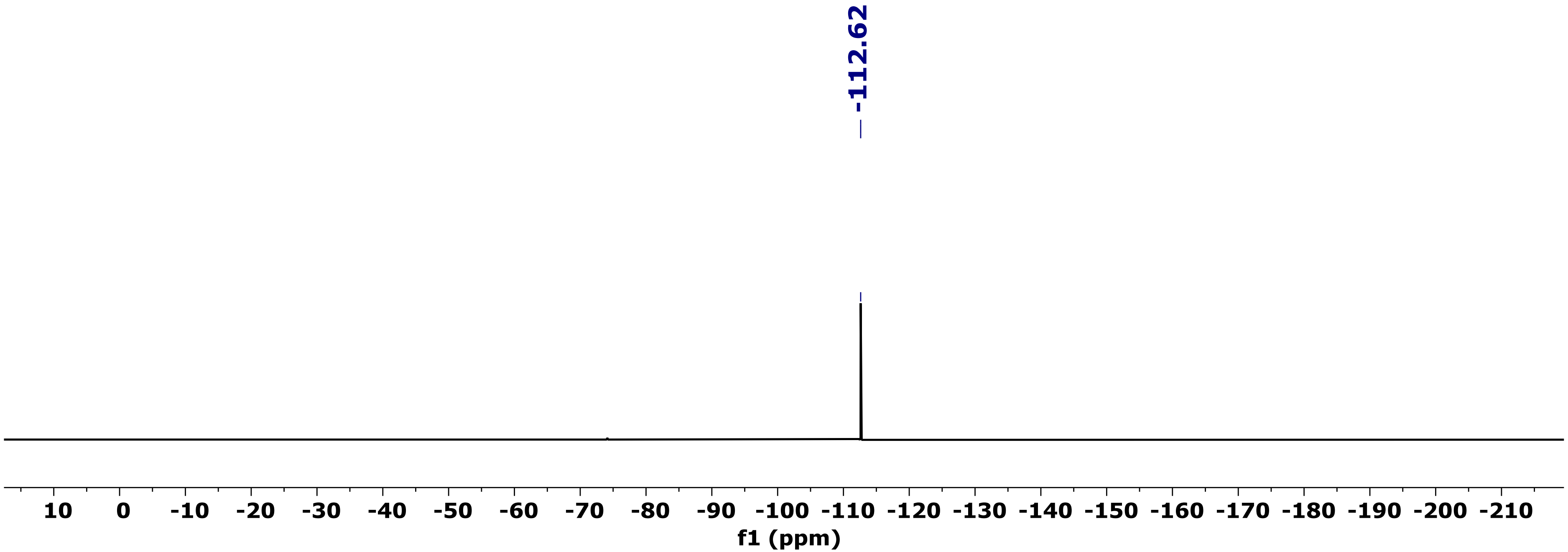

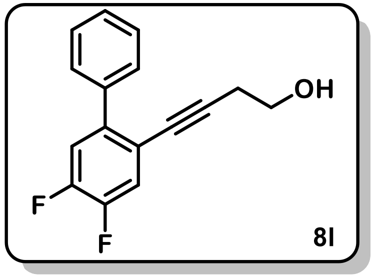

^{1}H NMR (400 MHz, $CDCl_3$)

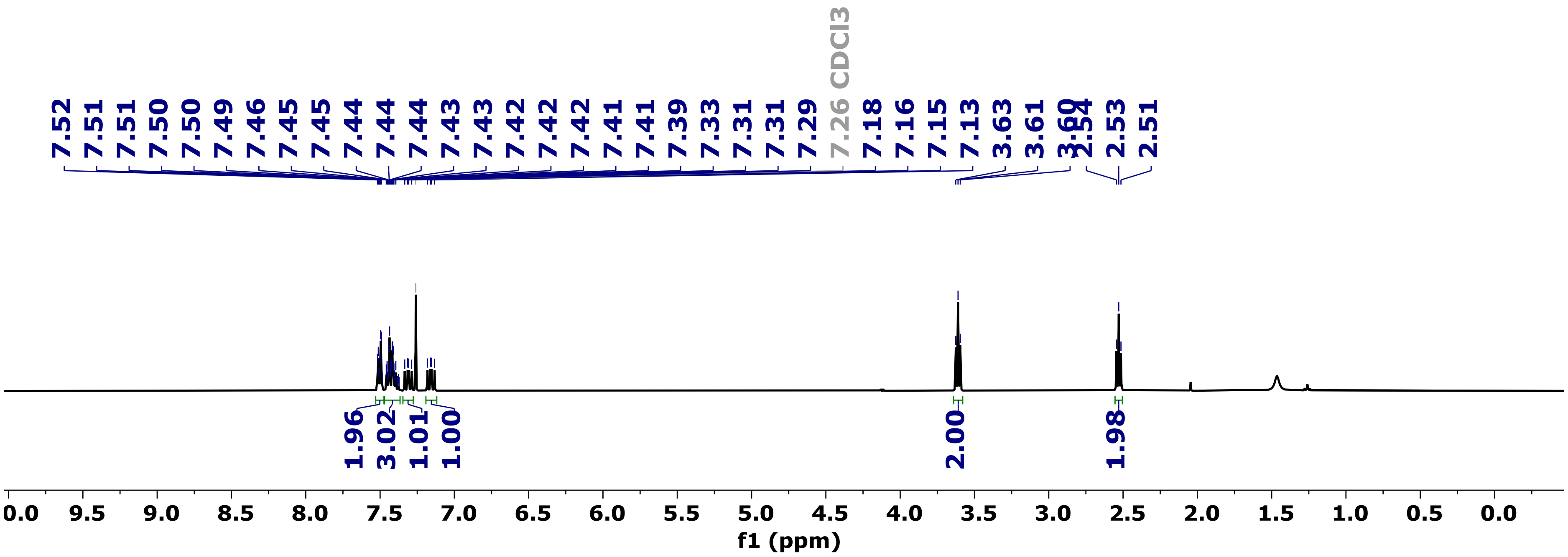

$^{13}C\{H\}$ NMR (101 MHz, $CDCl_3$)

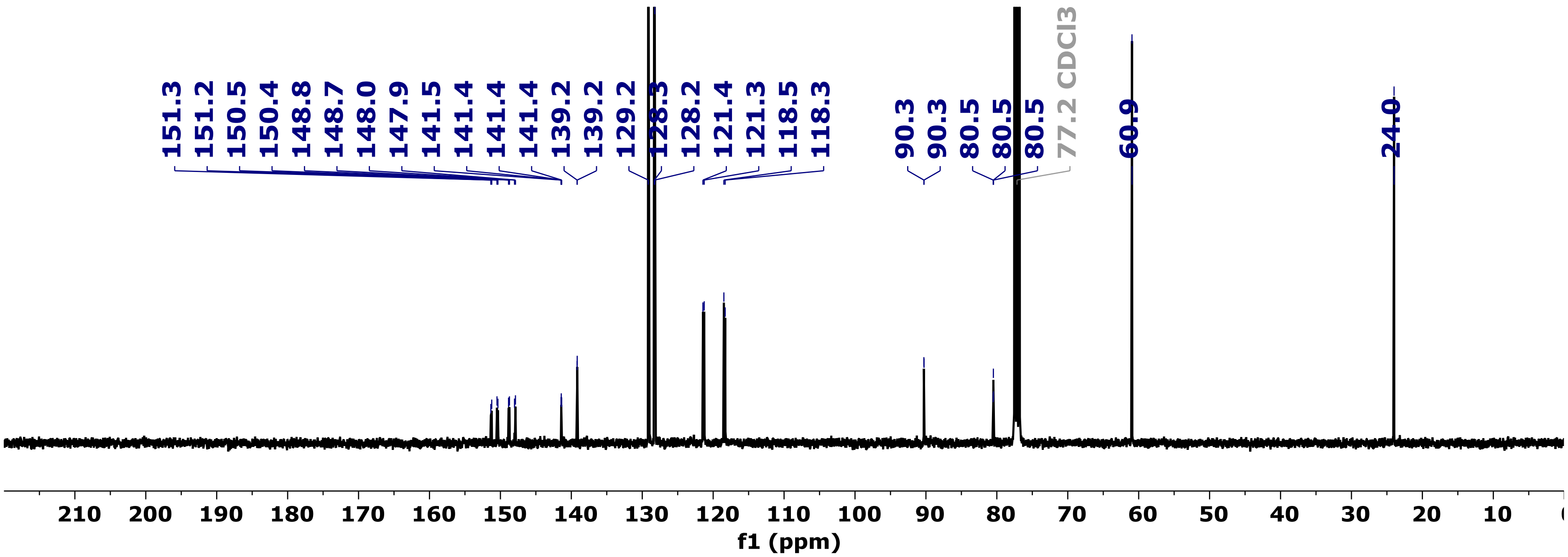

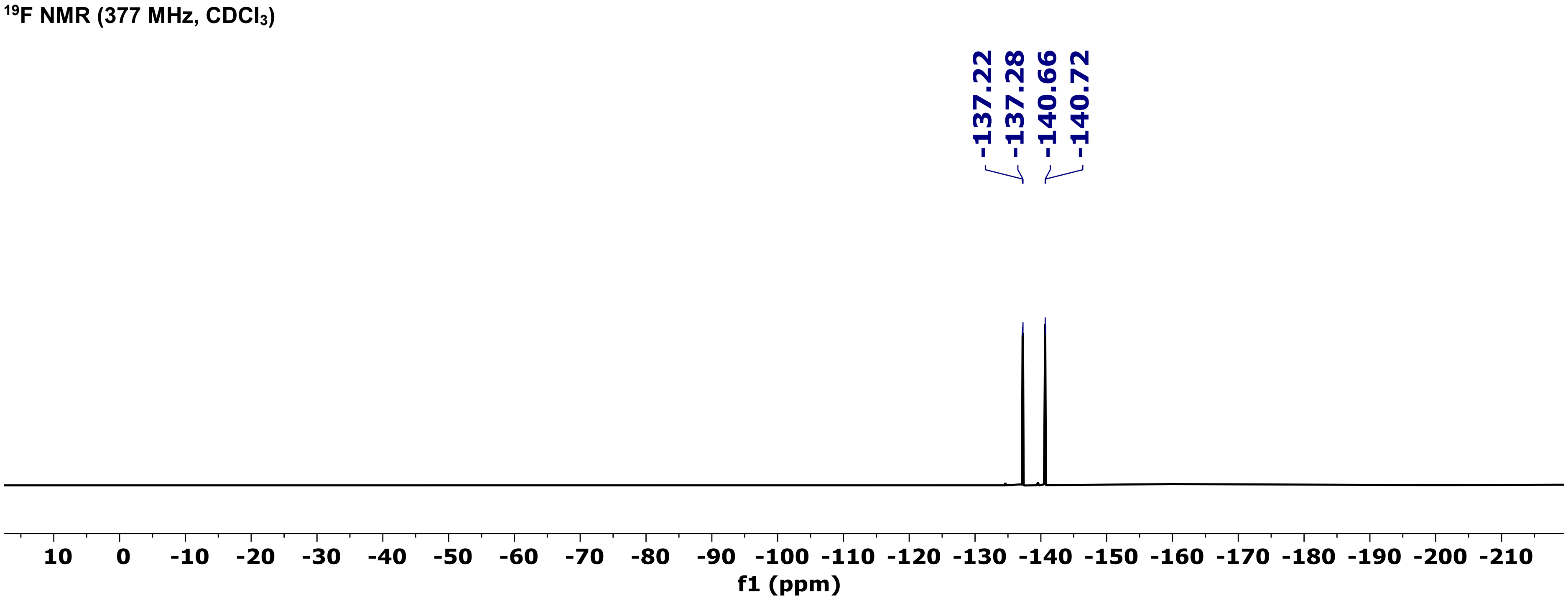
^{19}F NMR (377 MHz, $CDCl_3$)
-137.22
-137.28
-140.66
-140.72
10 0 -10 -20 -30 -40 -50 -60 -70 -80 -90 -100 -110 -120 -130 -140 -150 -160 -170 -180 -190 -200 -210
f1 (ppm)

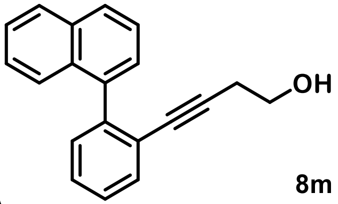

8m

^{1}H NMR (400 MHz, $CDCl_3$)

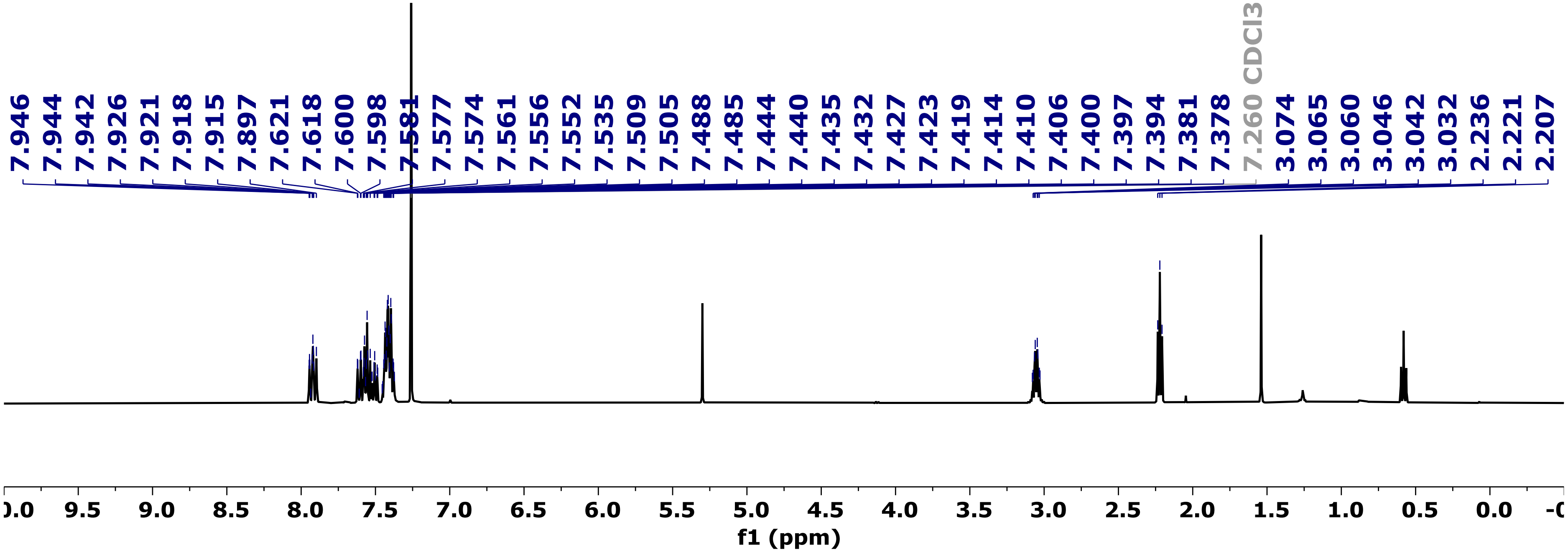

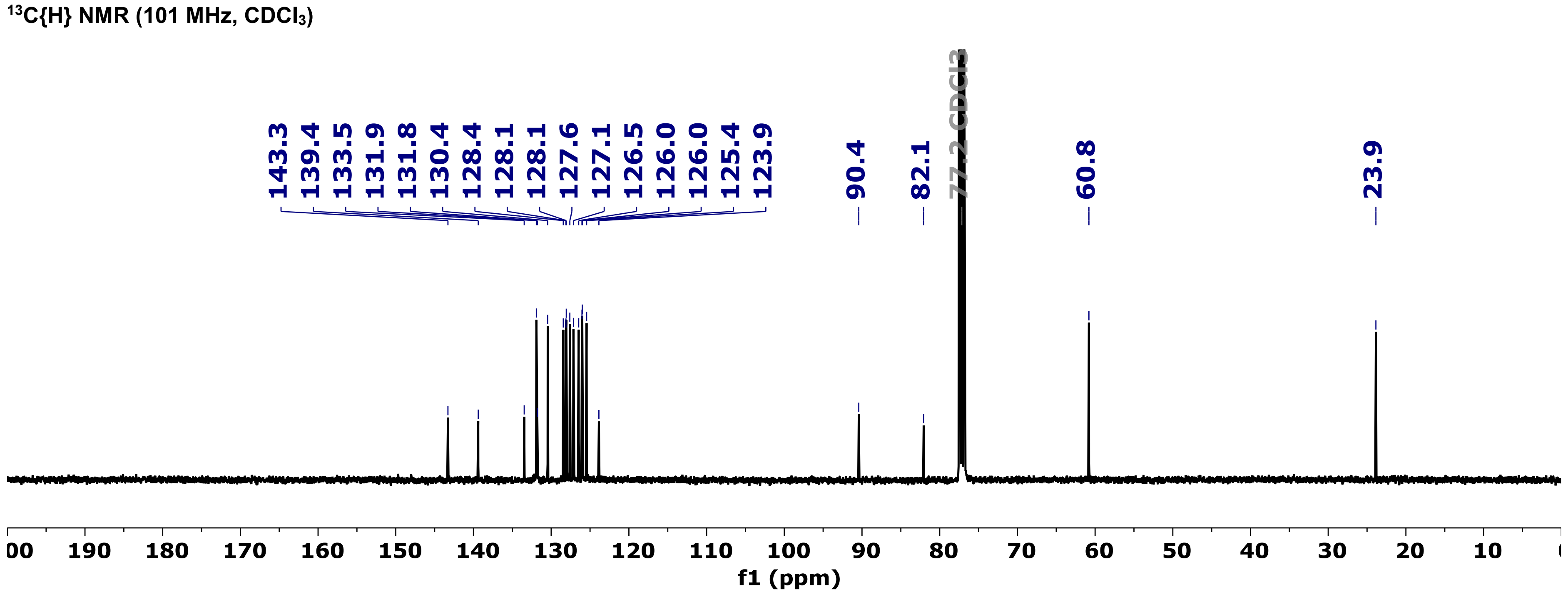

13C{H} NMR (101 MHz, CDCl3)
143.3
139.4
133.5
131.9
131.8
130.4
128.4
128.1
128.1
127.6
127.1
126.5
126.0
126.0
125.4
123.9
90.4
82.1
77.2 CDCl3
60.8
23.9
f1 (ppm)

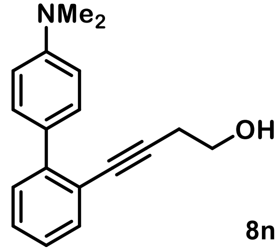

8n

^{1}H NMR (400 MHz, $CDCl_3$)

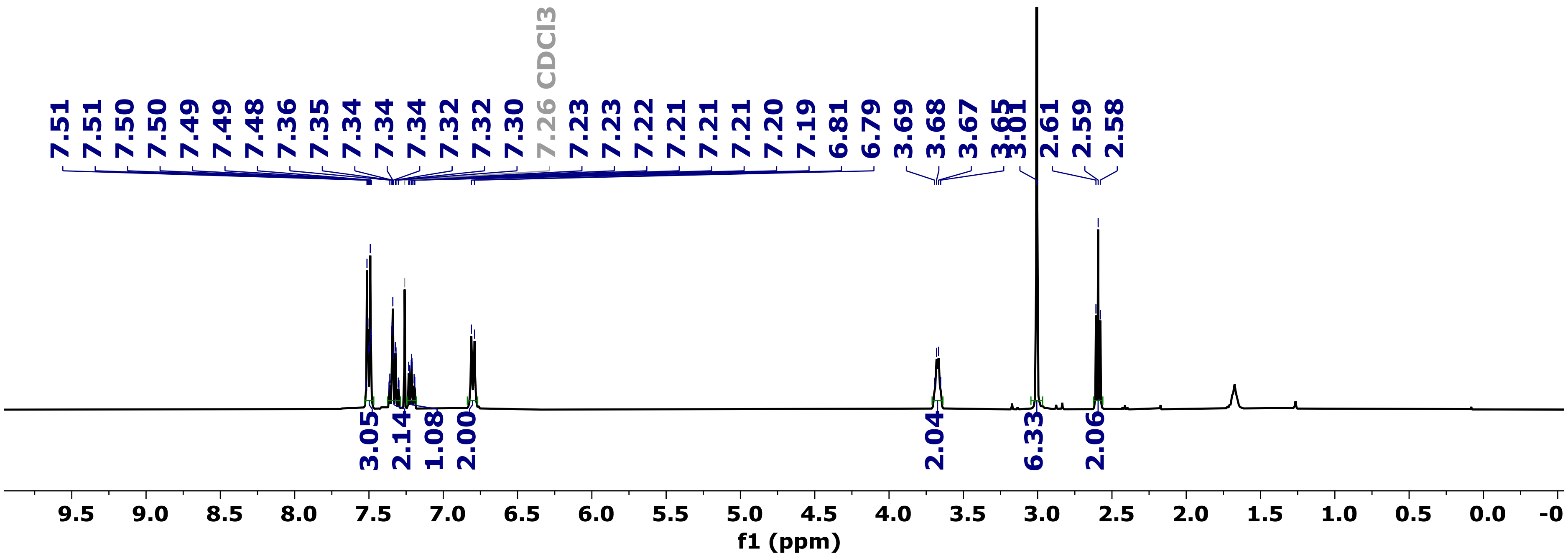

$^{13}C\{H\}$ NMR (101 MHz, $CDCl_3$)

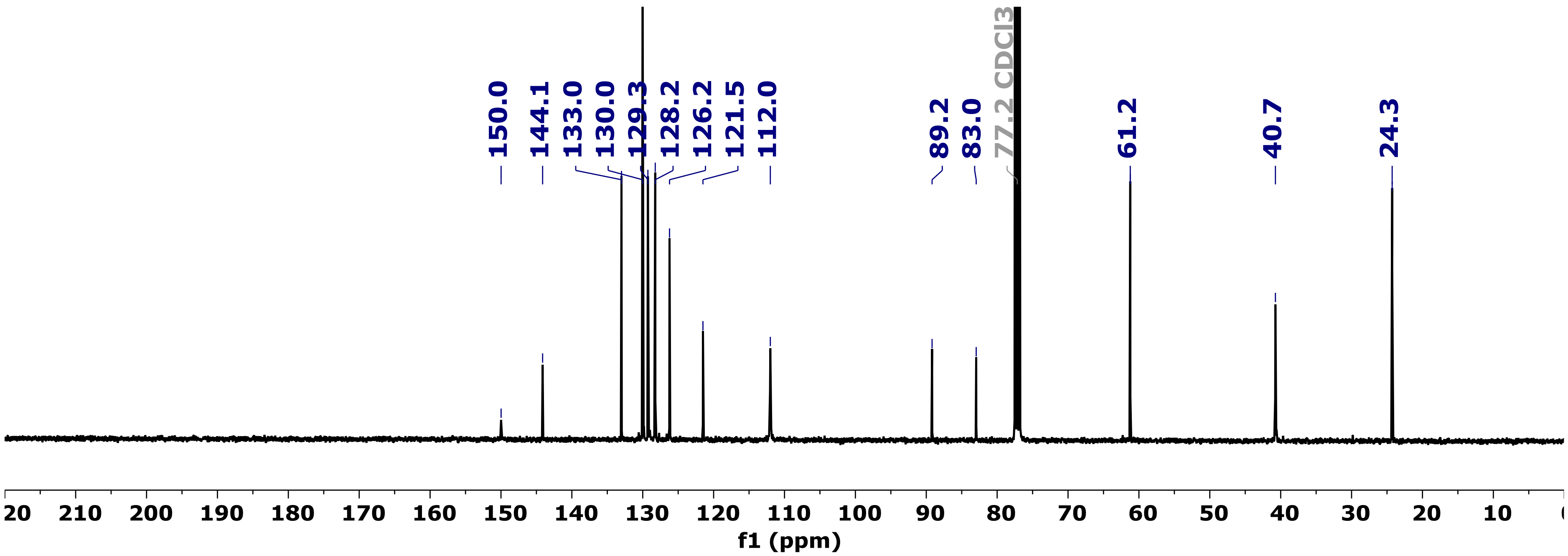

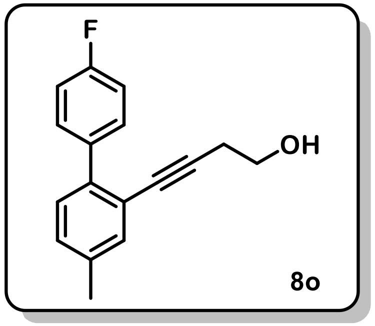

^{1}H NMR (400 MHz, $CDCl_3$)

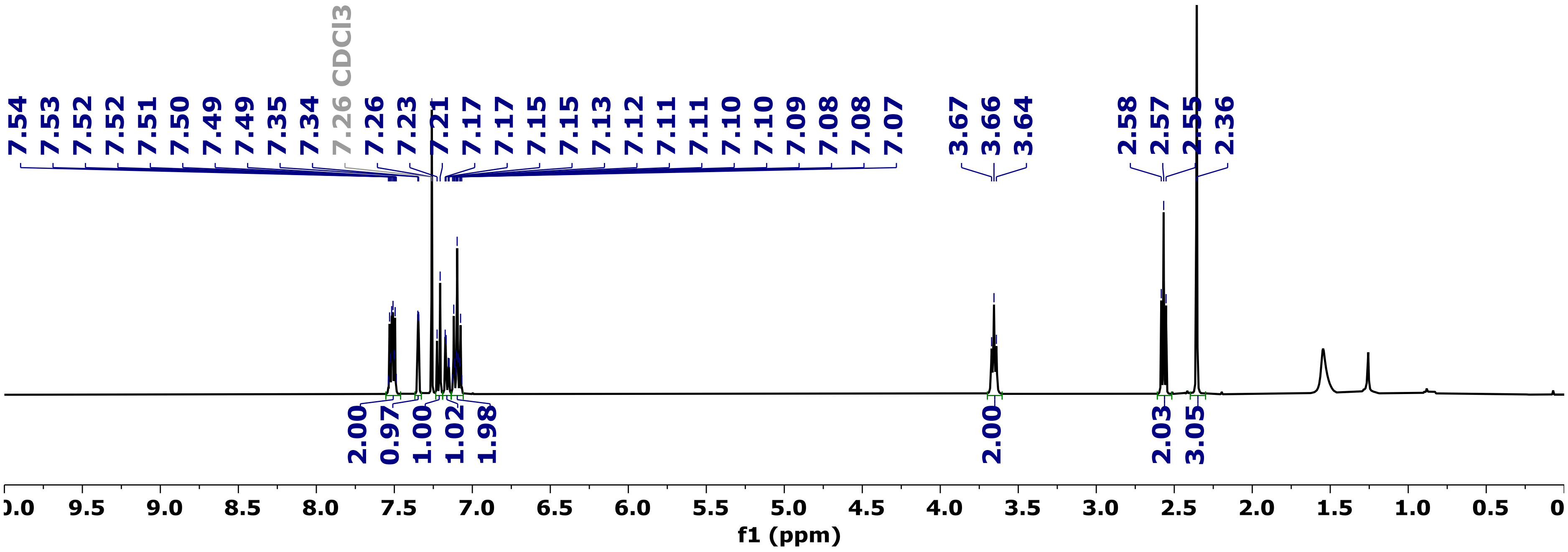

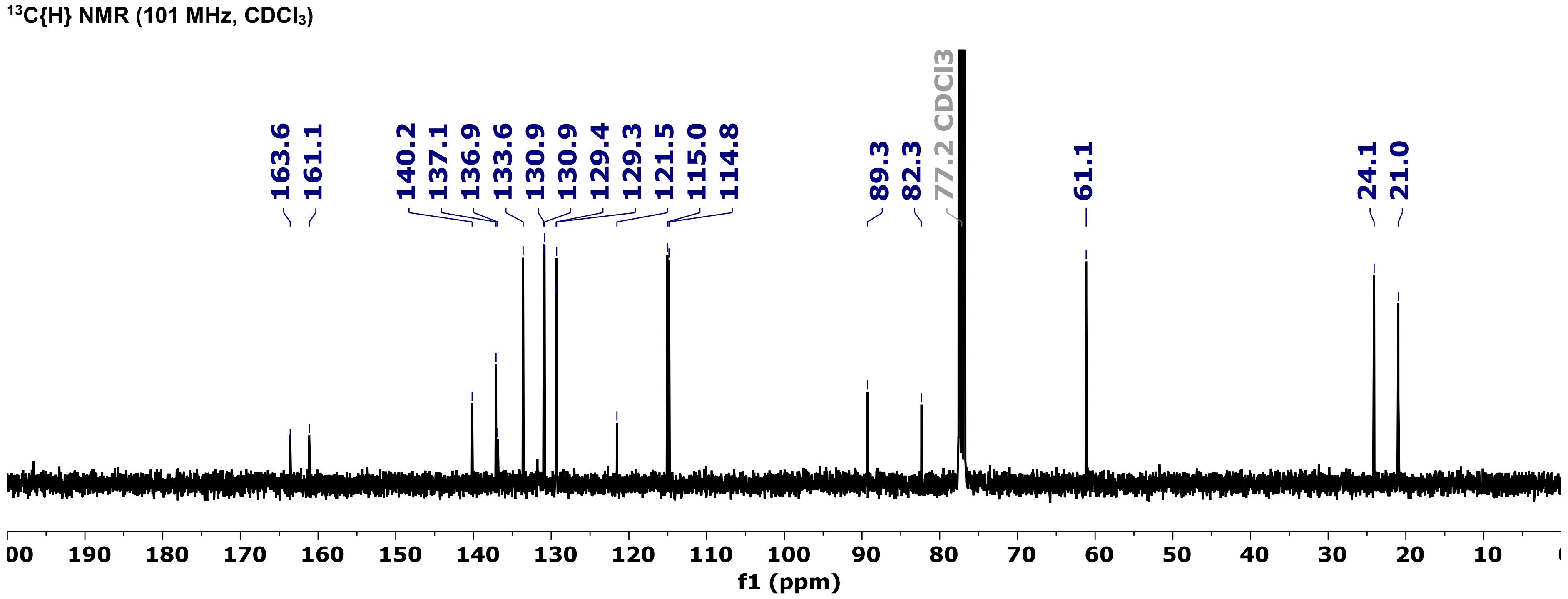

$^{13}C\{H\}$ NMR (101 MHz, $CDCl_3$)
163.6
161.1
140.2
137.1
136.9
133.6
130.9
130.9
129.4
129.3
121.5
115.0
114.8
89.3
82.3
77.2 CDCl3
61.1
24.1
21.0
f1 (ppm)

$^{19}F\{H\}$ NMR (377 MHz, $CDCl_3$)

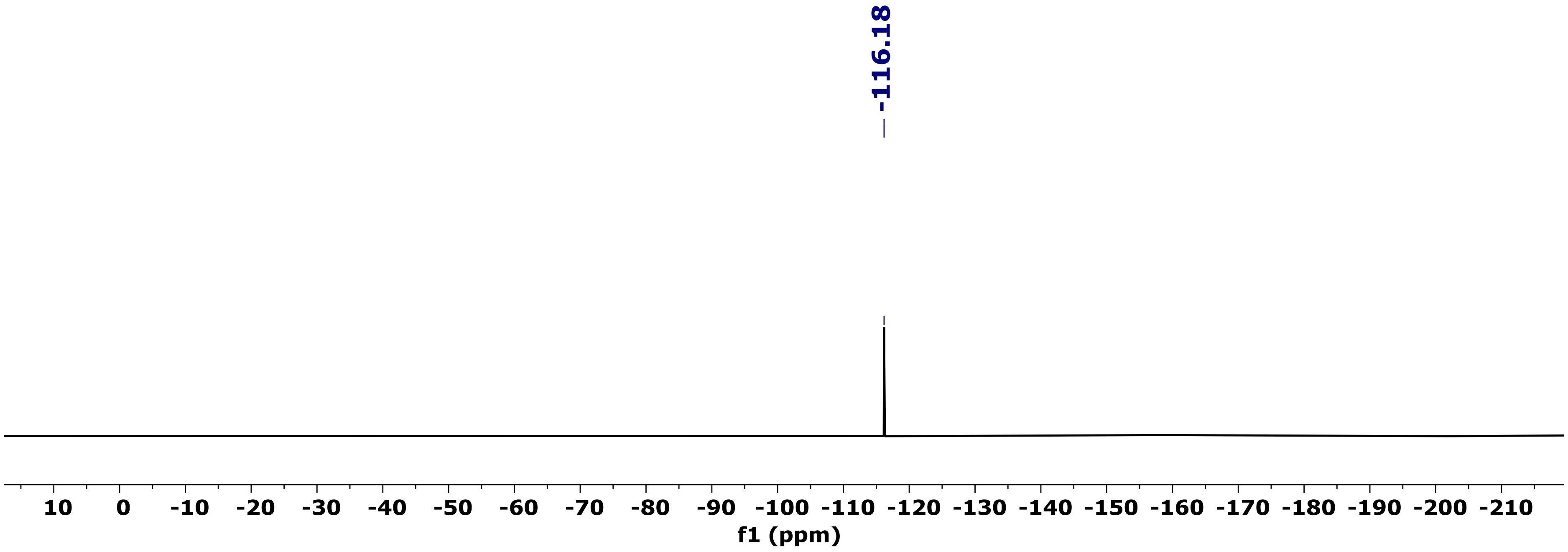

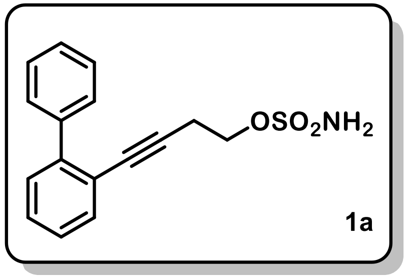

1a

^{1}H NMR (400 MHz, $CDCl_3$)

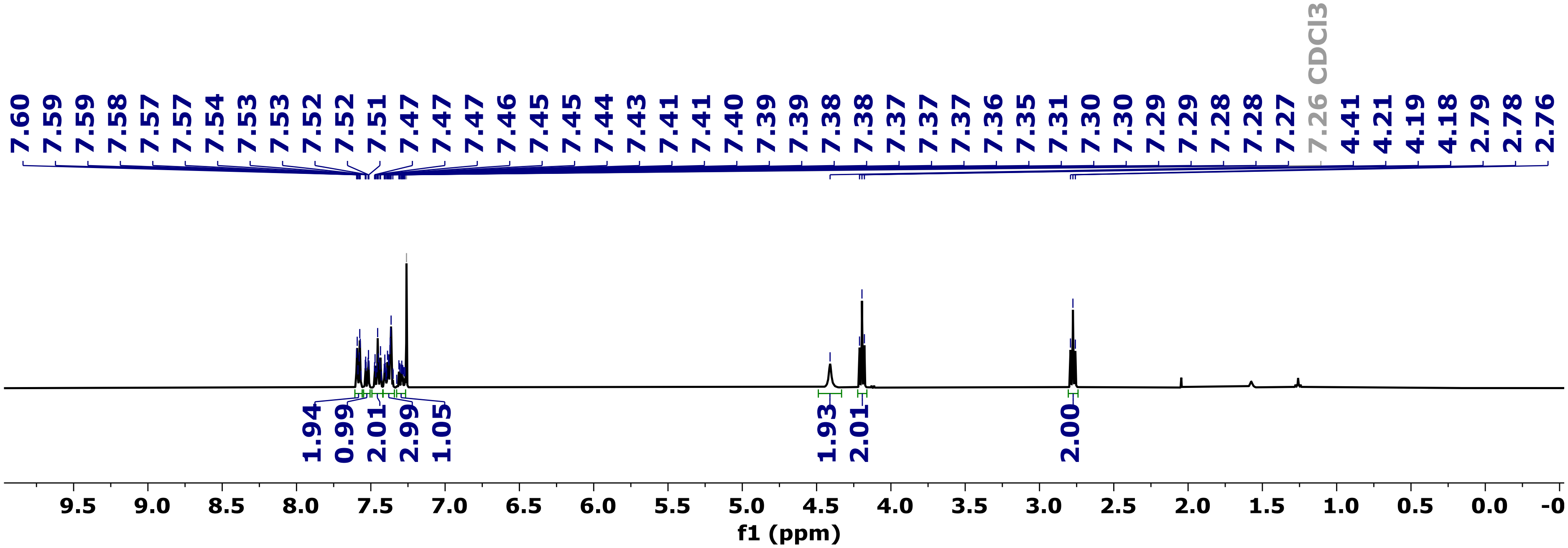

$^{13}C\{H\}$ NMR (101 MHz, $CDCl_3$)

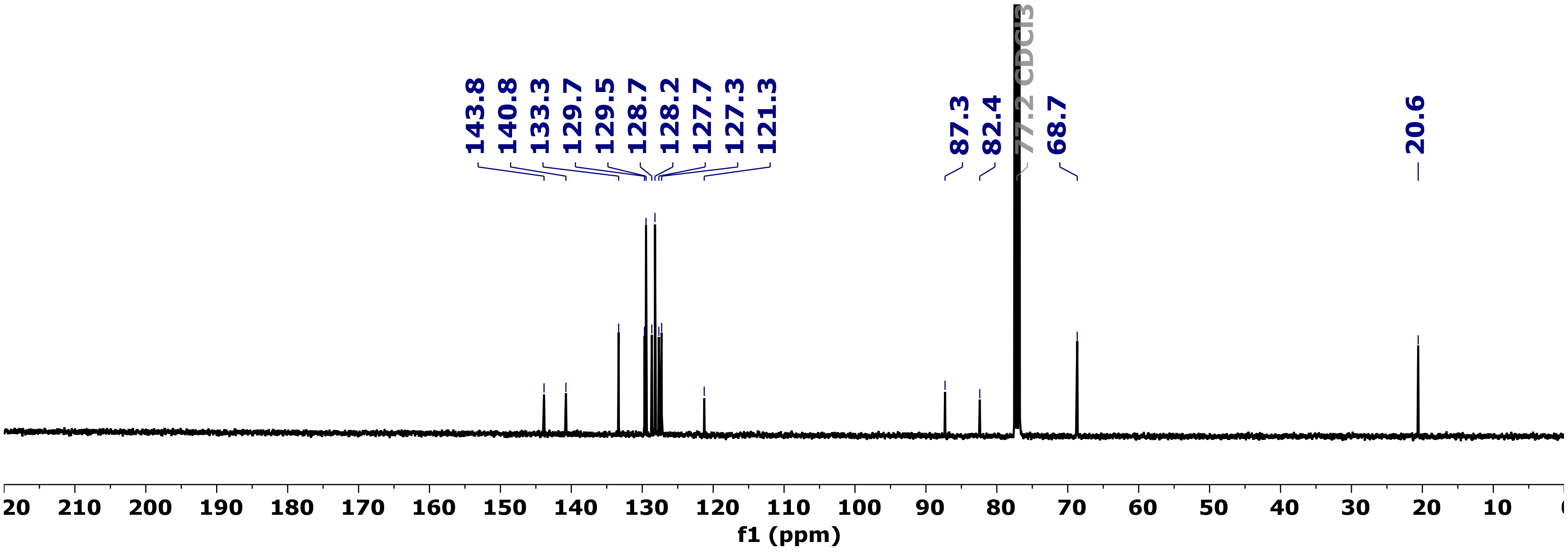

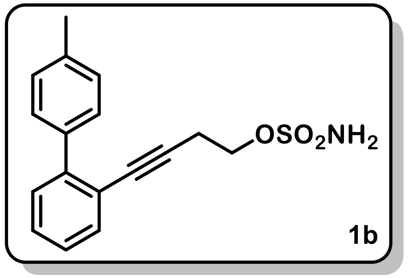

1b

1H NMR (400 MHz, CDCl3)

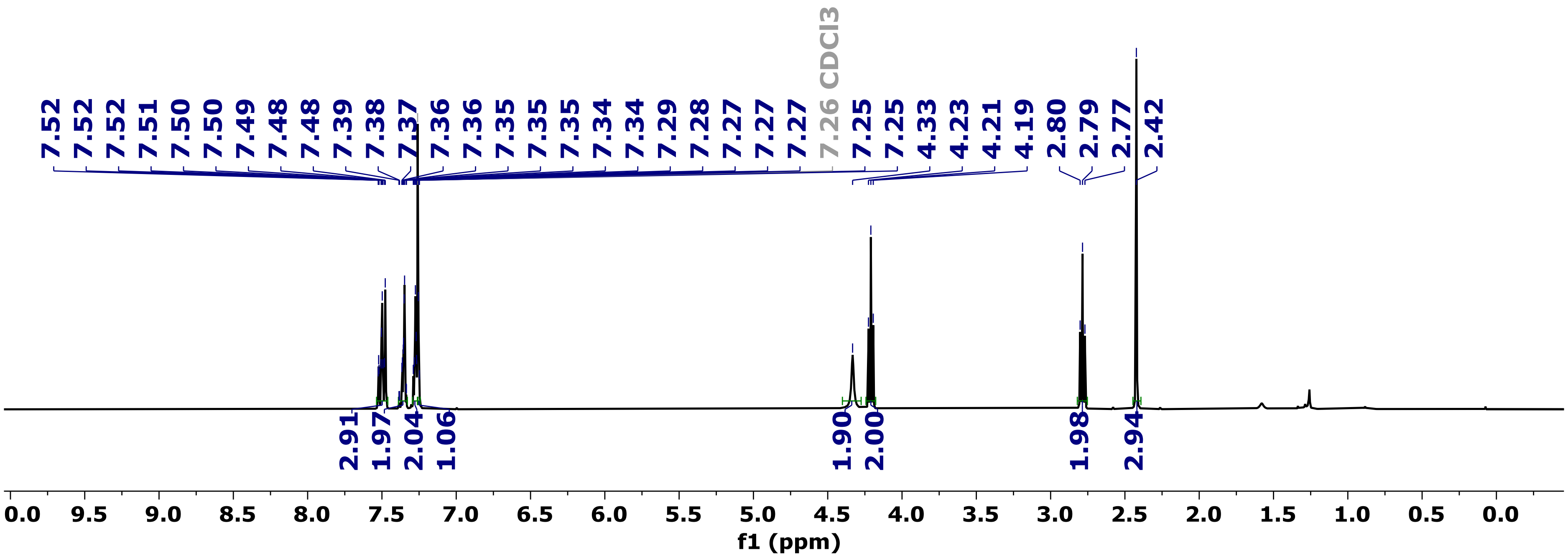

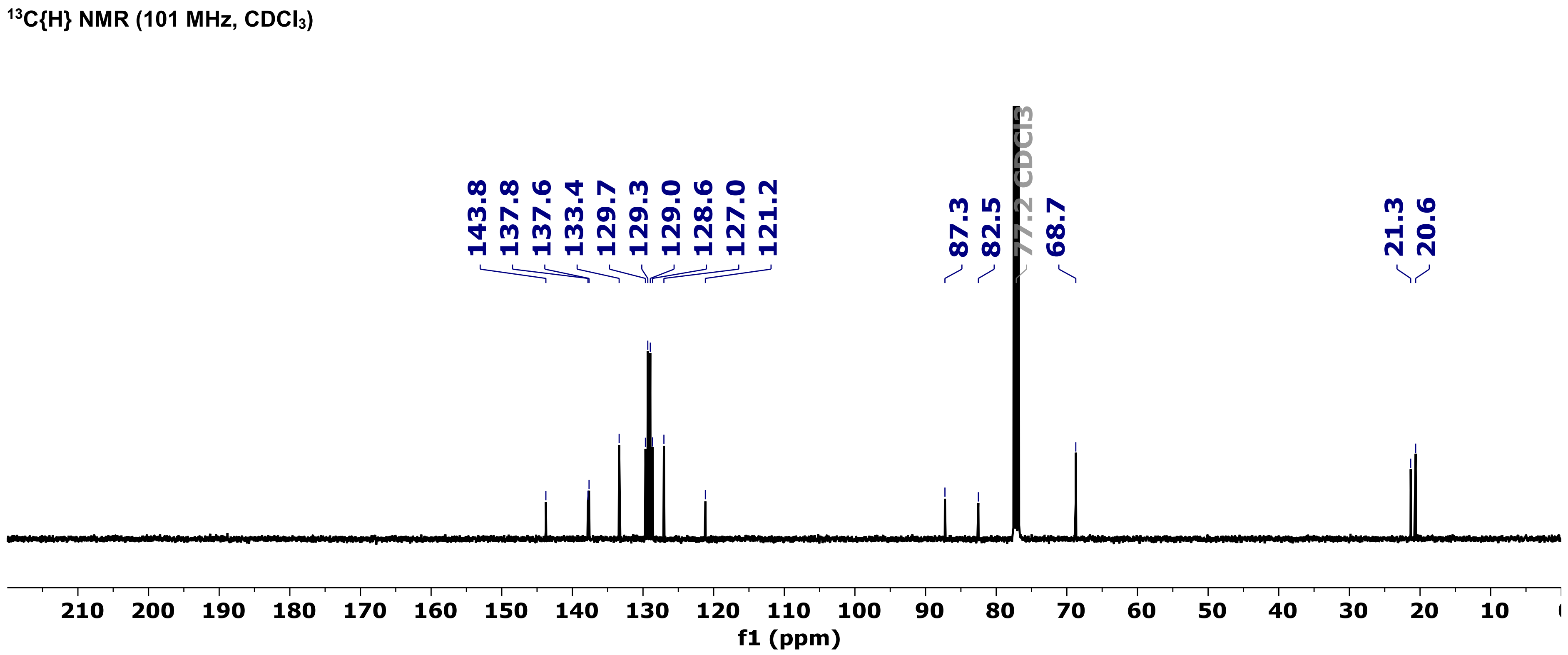
$^{13}C\{H\}$ NMR (101 MHz, $CDCl_3$)
143.8
137.8
137.6
133.4
129.7
129.3
129.0
128.6
127.0
121.2
87.3
82.5
77.2 CDCl3
68.7
21.3
20.6
210 200 190 180 170 160 150 140 130 120 110 100 90 80 70 60 50 40 30 20 10
f1 (ppm)

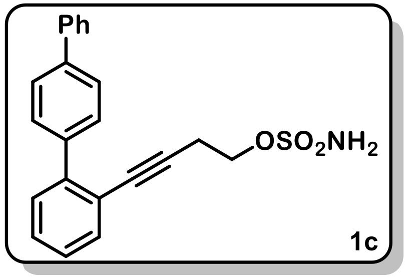

^{1}H NMR (400 MHz, $CDCl_3$)

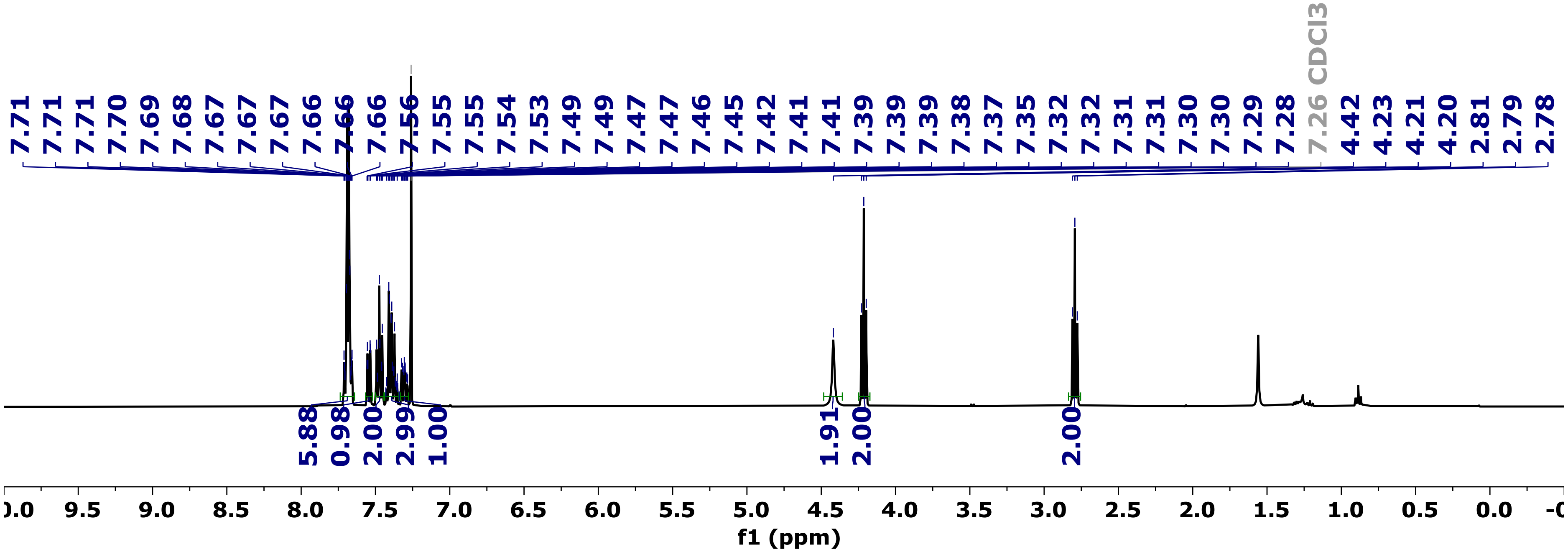

$^{13}C\{H\}$ NMR (101 MHz, $CDCl_3$)

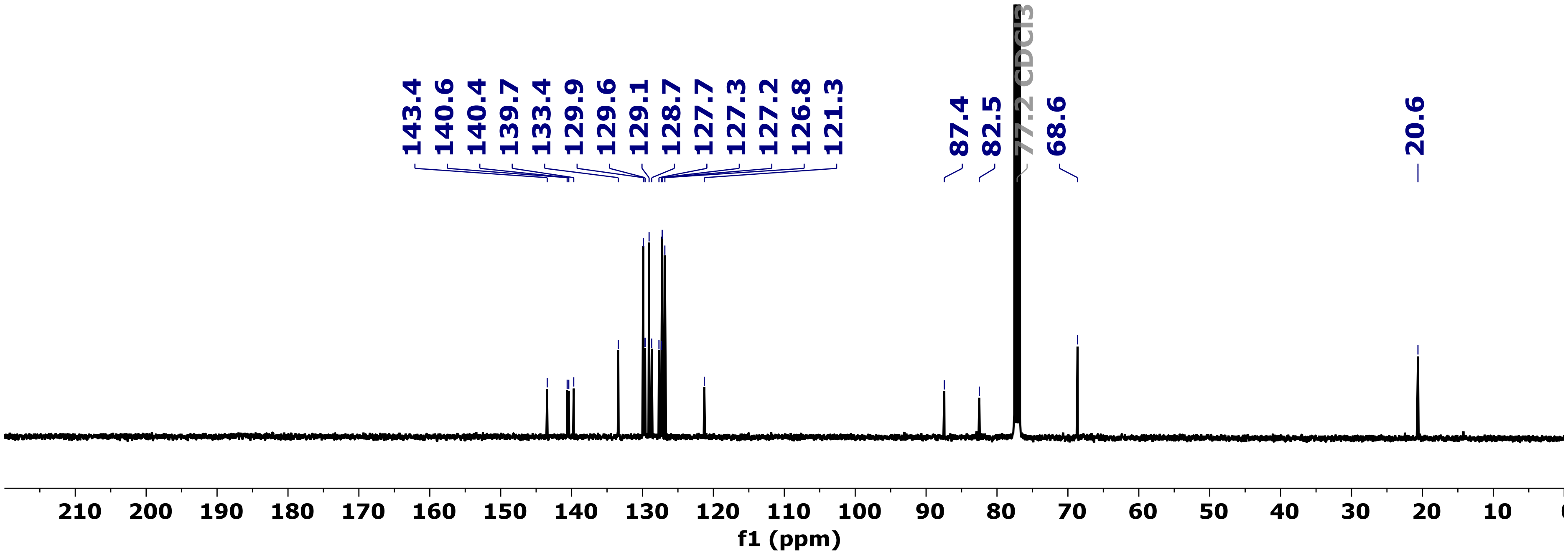

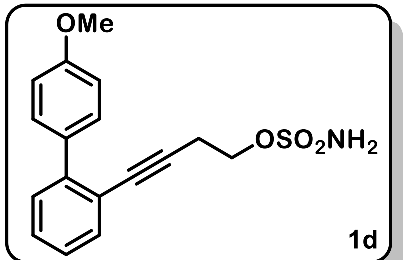

1d

^{1}H NMR (400 MHz, $CDCl_3$)

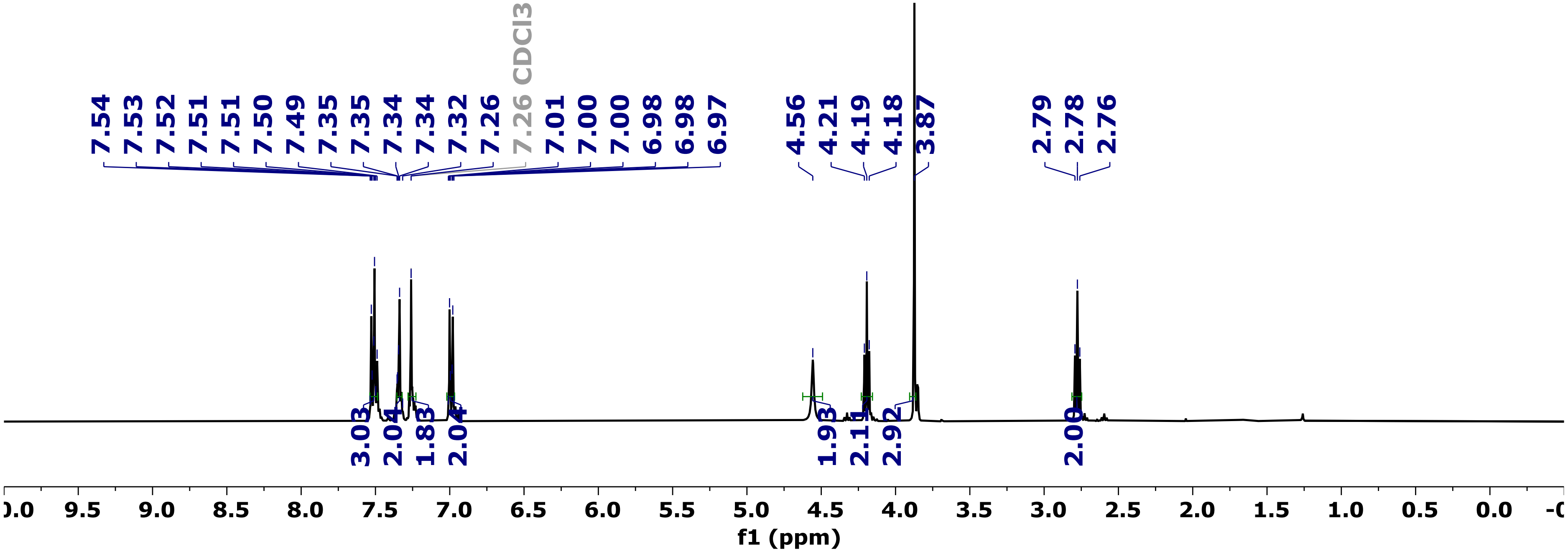

$^{13}C\{H\}$ NMR (101 MHz, $CDCl_3$)

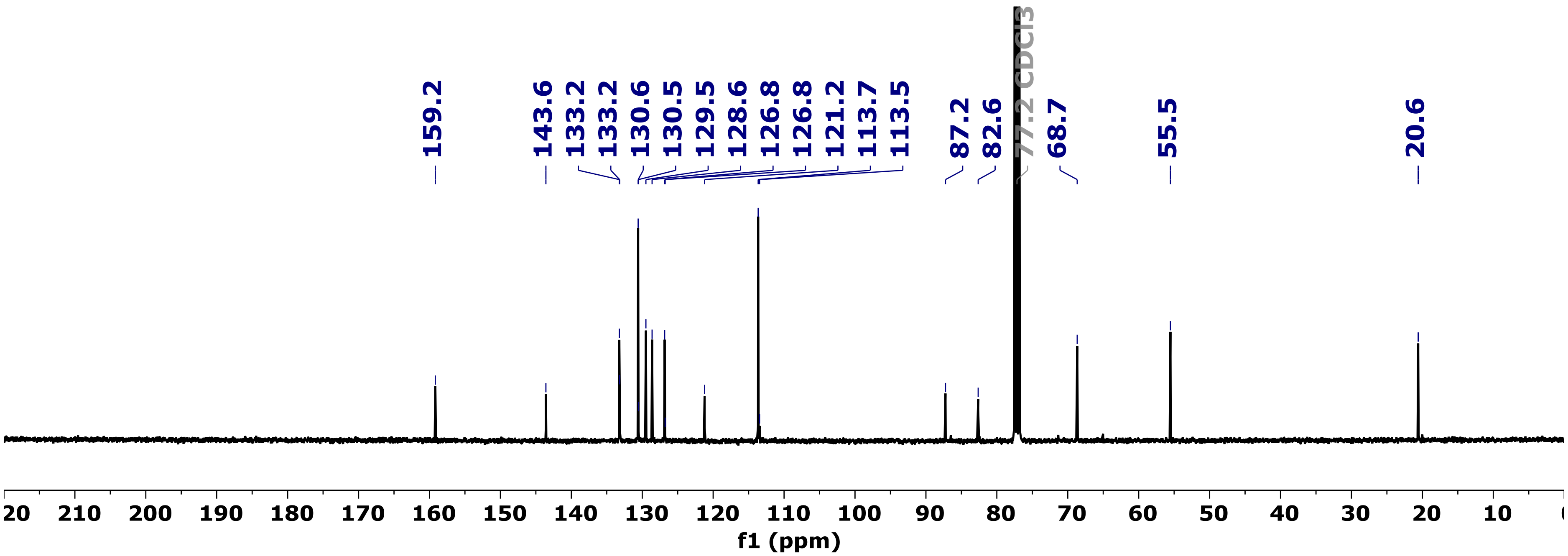

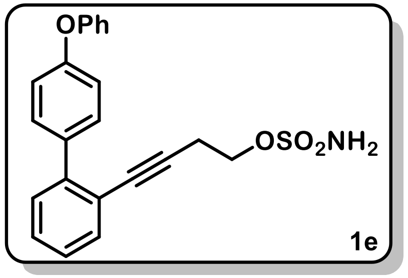

1e

^1H NMR (400 MHz, $CDCl_3$)

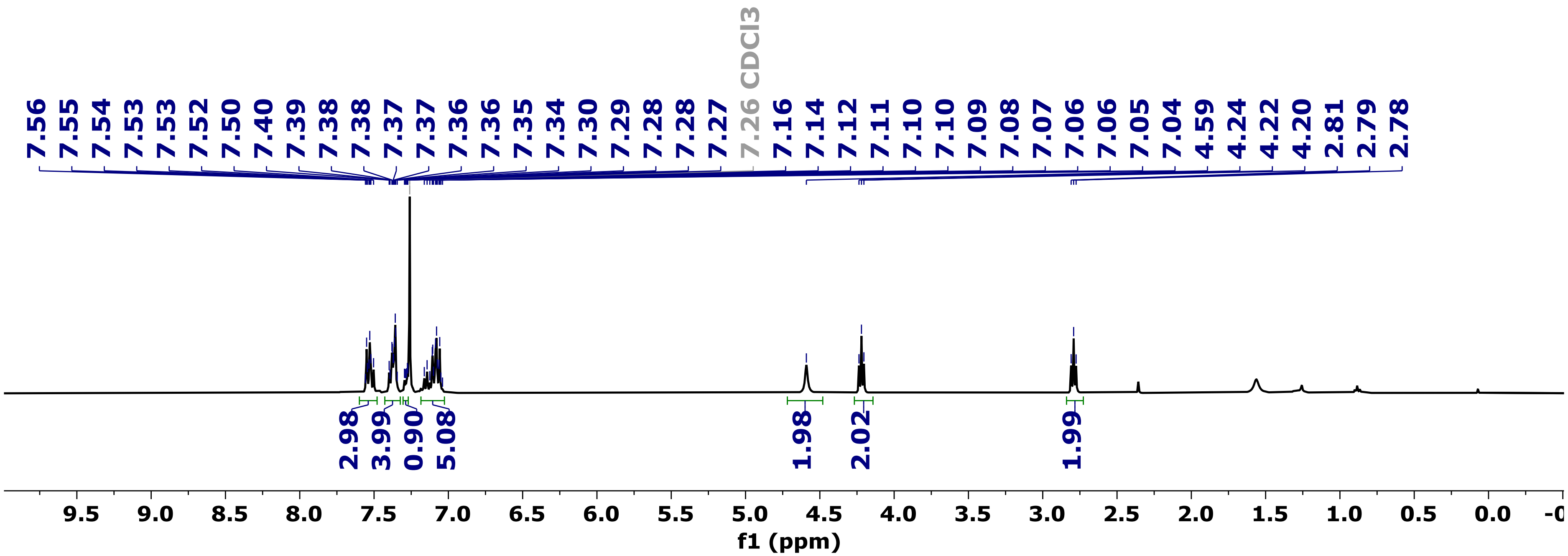

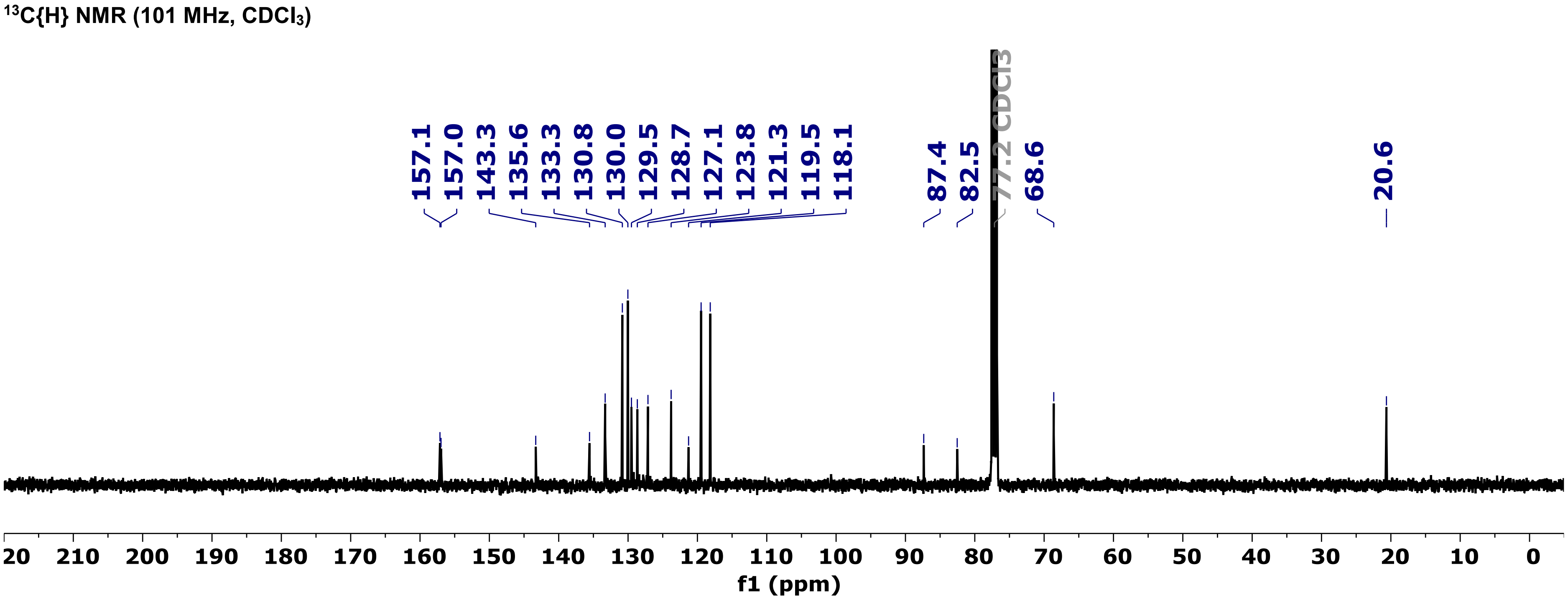

13C{H} NMR (101 MHz, CDCl3)
157.1
157.0
143.3
135.6
133.3
130.8
130.0
129.5
128.7
127.1
123.8
121.3
119.5
118.1
87.4
82.5
77.2 CDCl3
68.6
20.6
f1 (ppm)

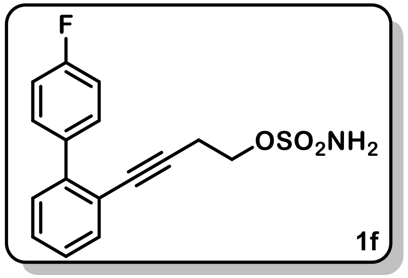

^{1}H NMR (400 MHz, $CDCl_3$)

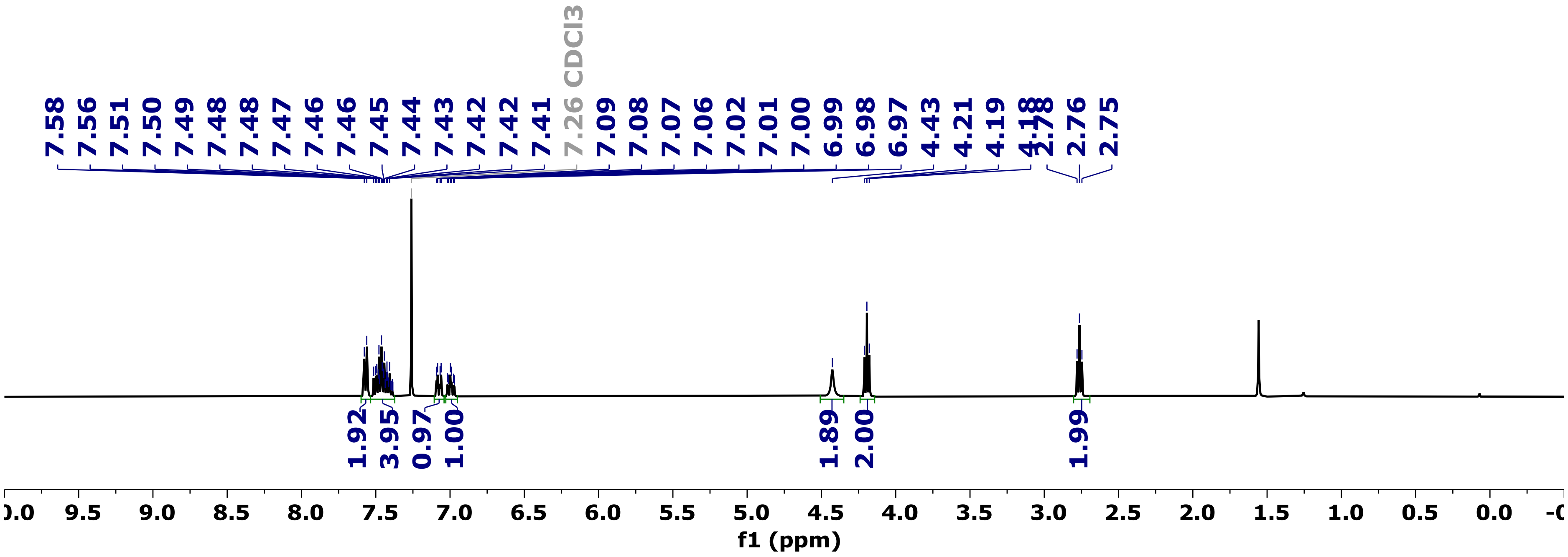

$^{13}C\{H\}$ NMR (101 MHz, $CDCl_3$)

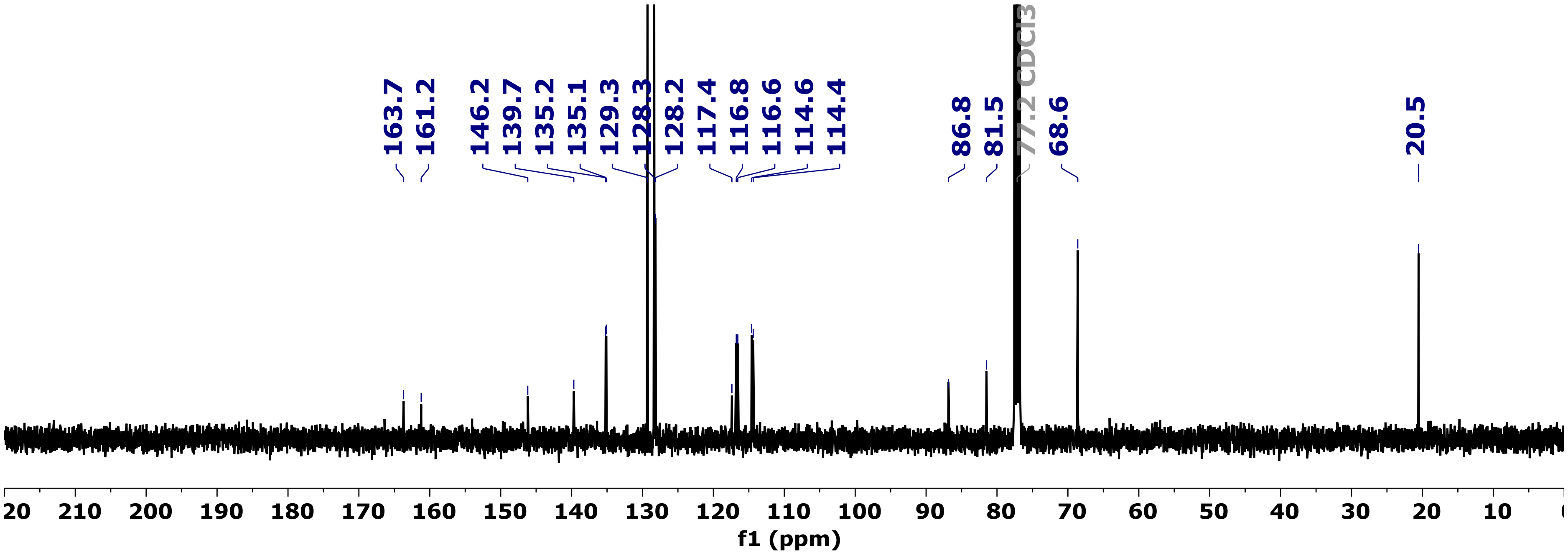

$^{19}F\{H\}$ NMR (377 MHz, $CDCl_3$)

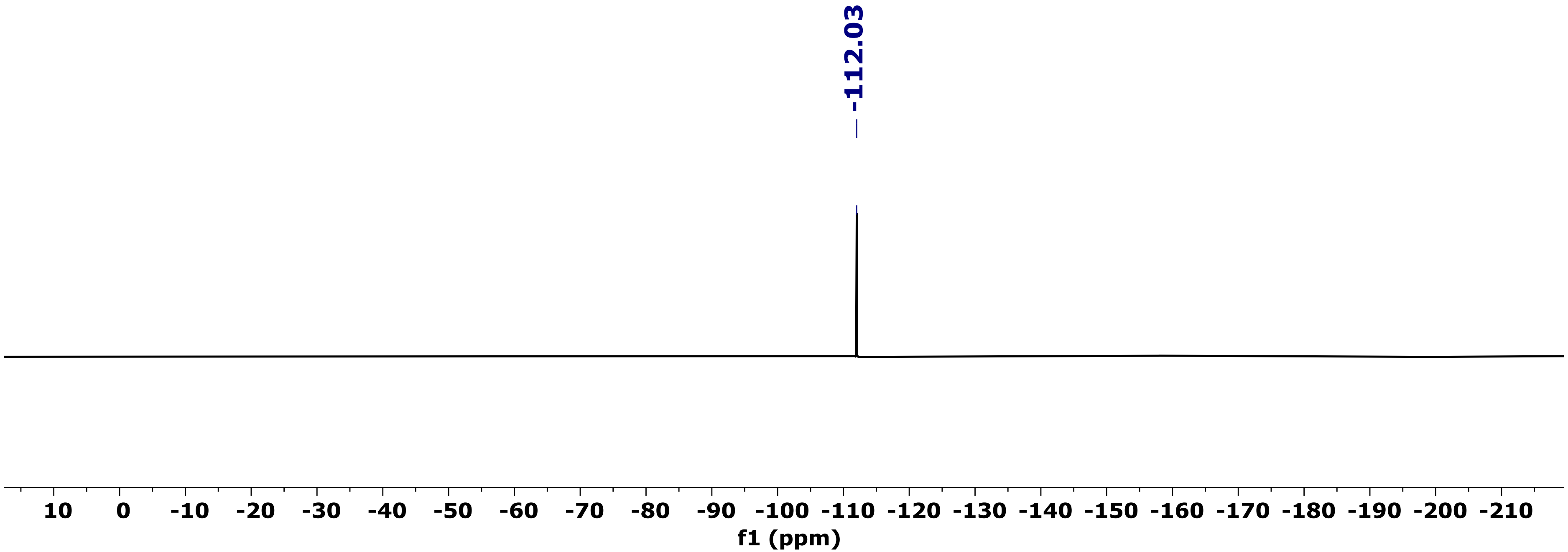

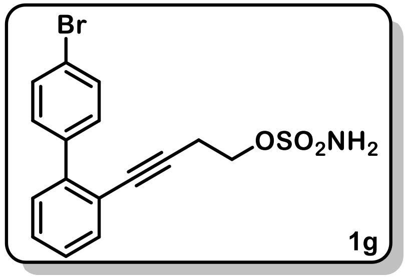

^{1}H NMR (400 MHz, $CDCl_3$)

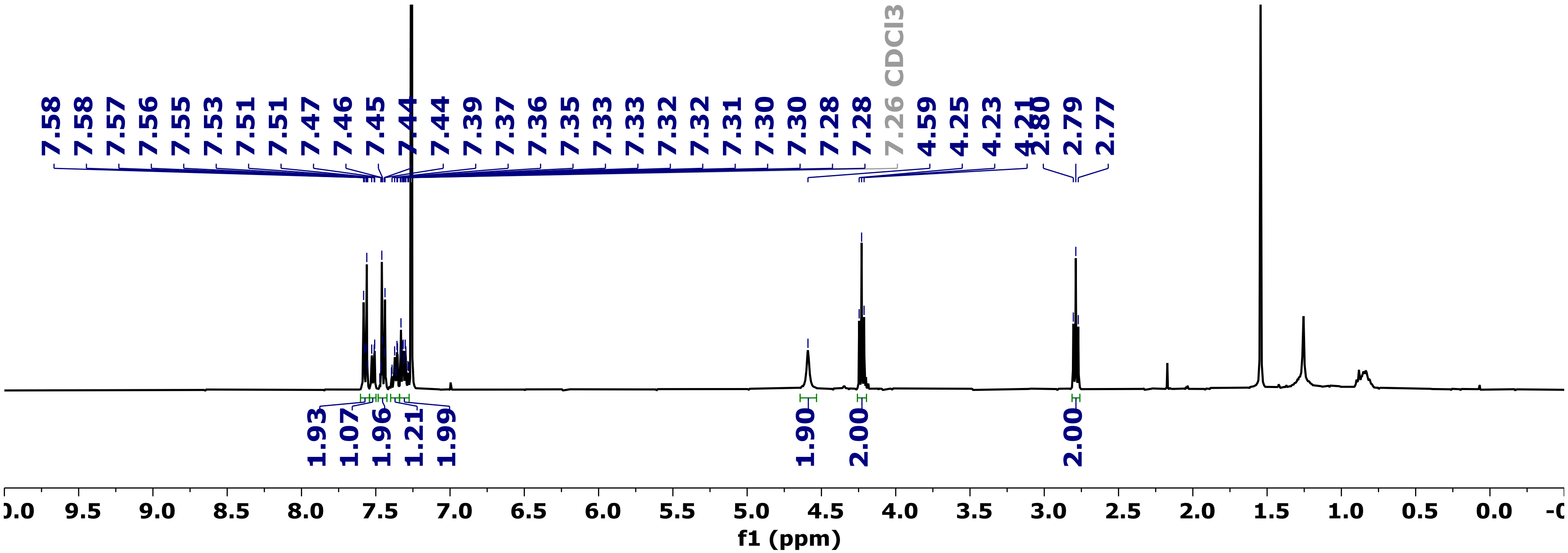

$^{13}C\{H\}$ NMR (101 MHz, $CDCl_3$)

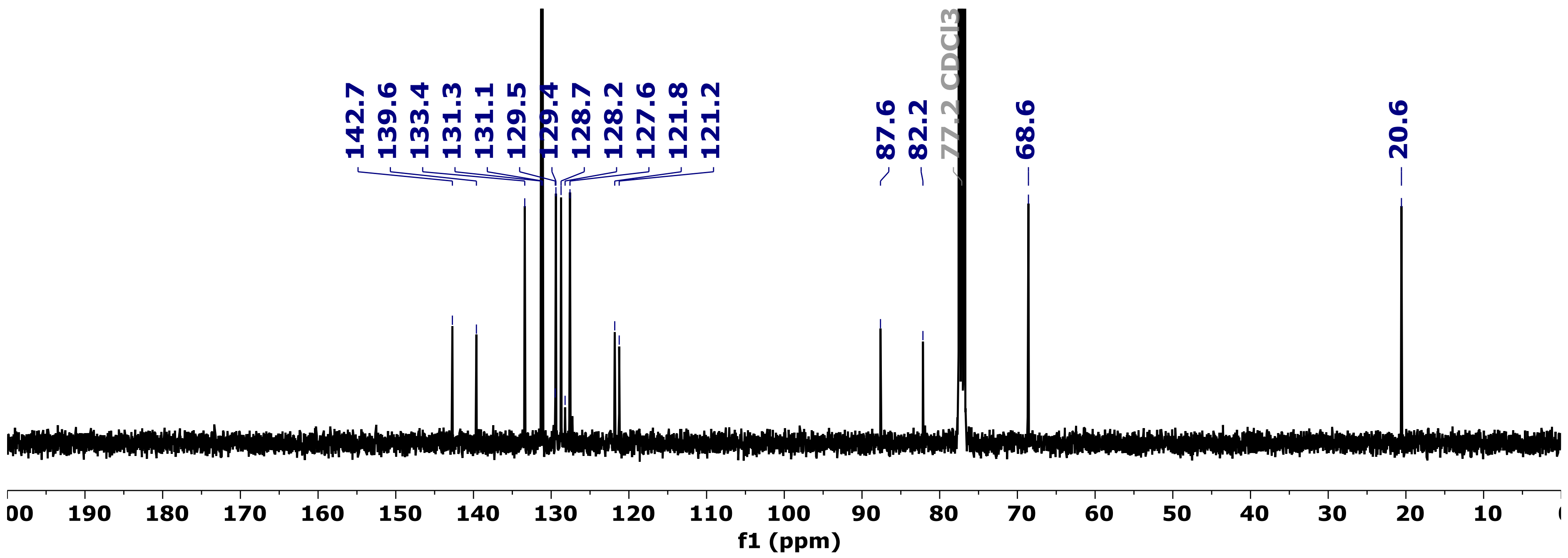

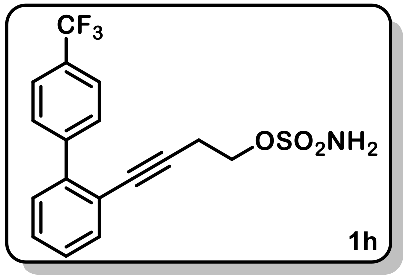

^{1}H NMR (400 MHz, $CDCl_3$)

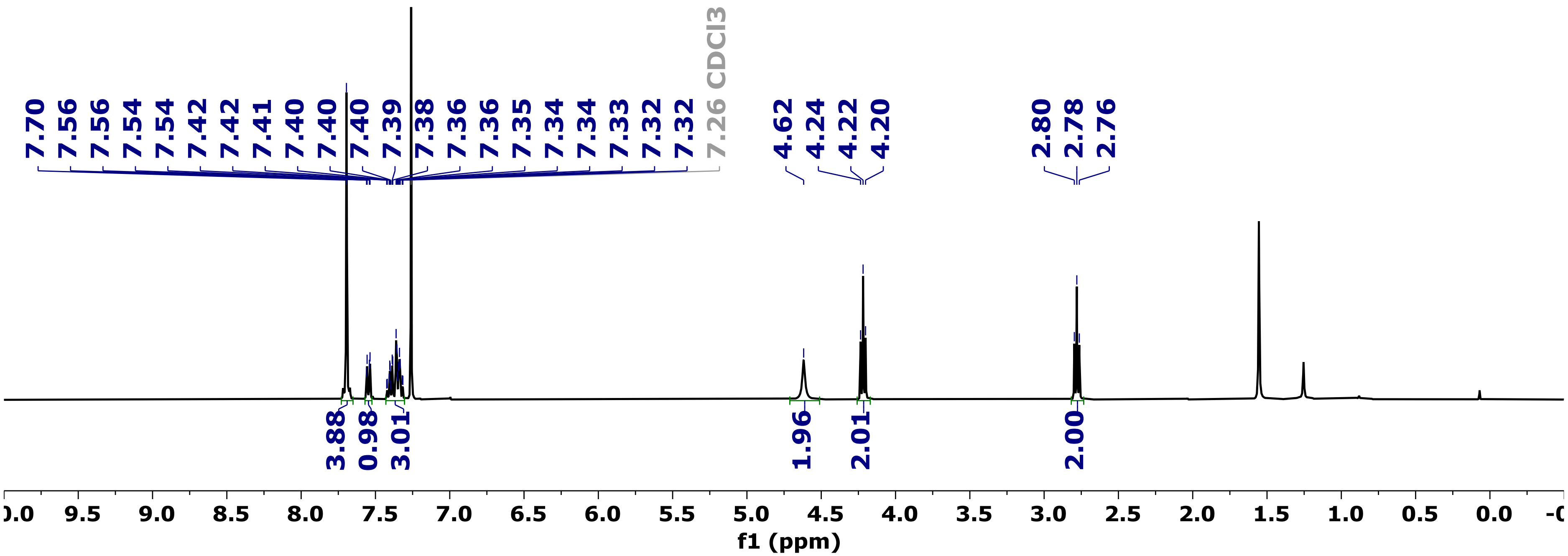

$^{13}C\{H\}$ NMR (101 MHz, $CDCl_3$)

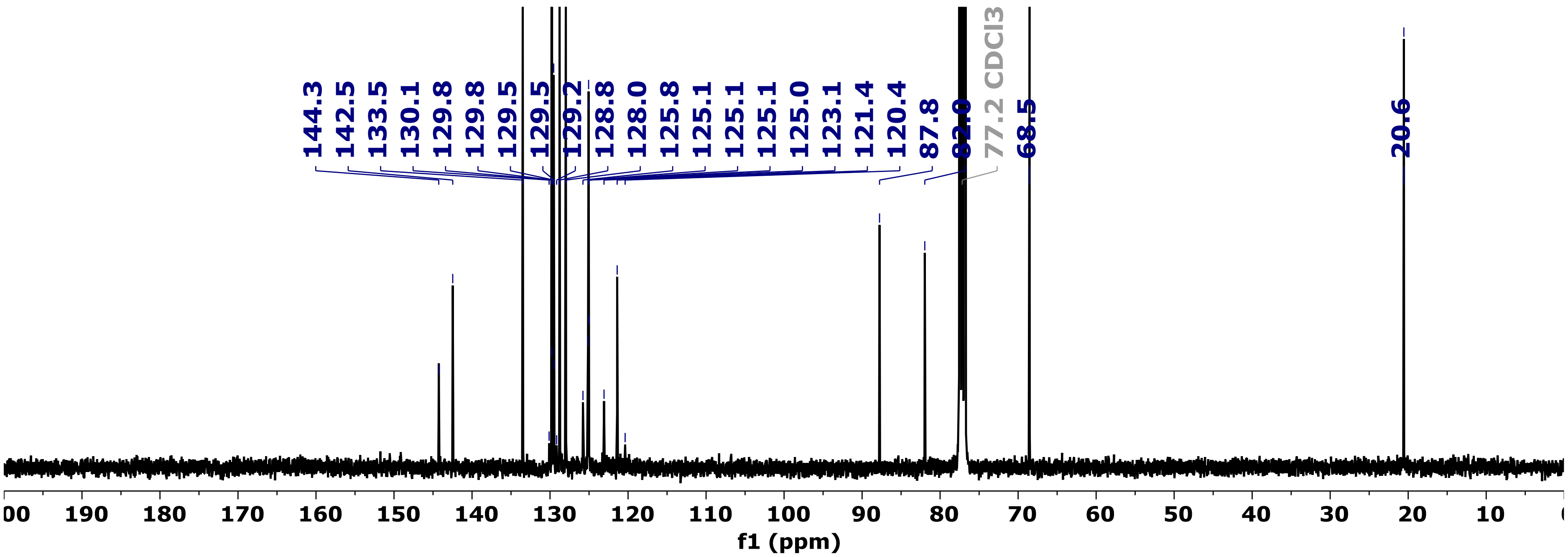

$^{19}F\{H\}$ NMR (377 MHz, $CDCl_3$)

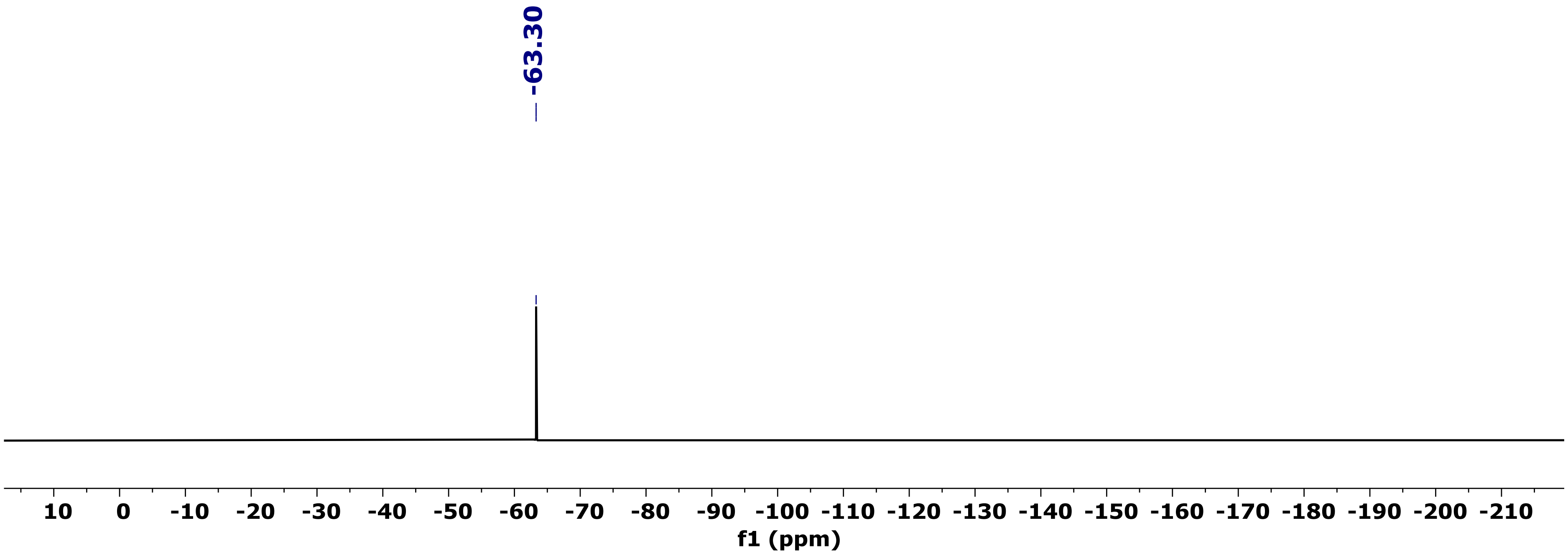

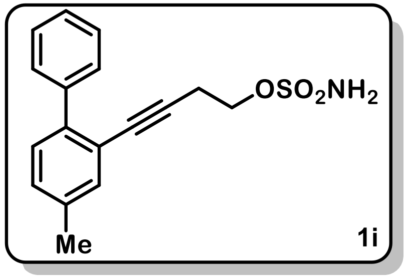

^{1}H NMR (400 MHz, $CDCl_3$)

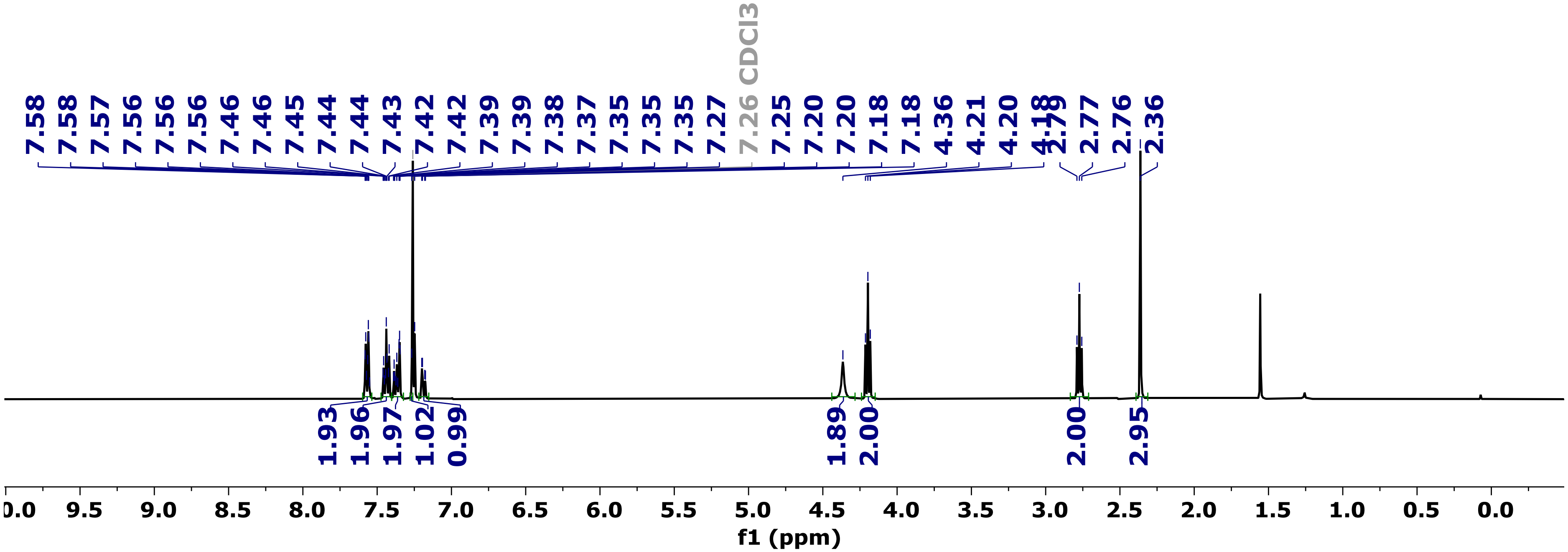

^{13}C{H} NMR (101 MHz, $CDCl_3$)

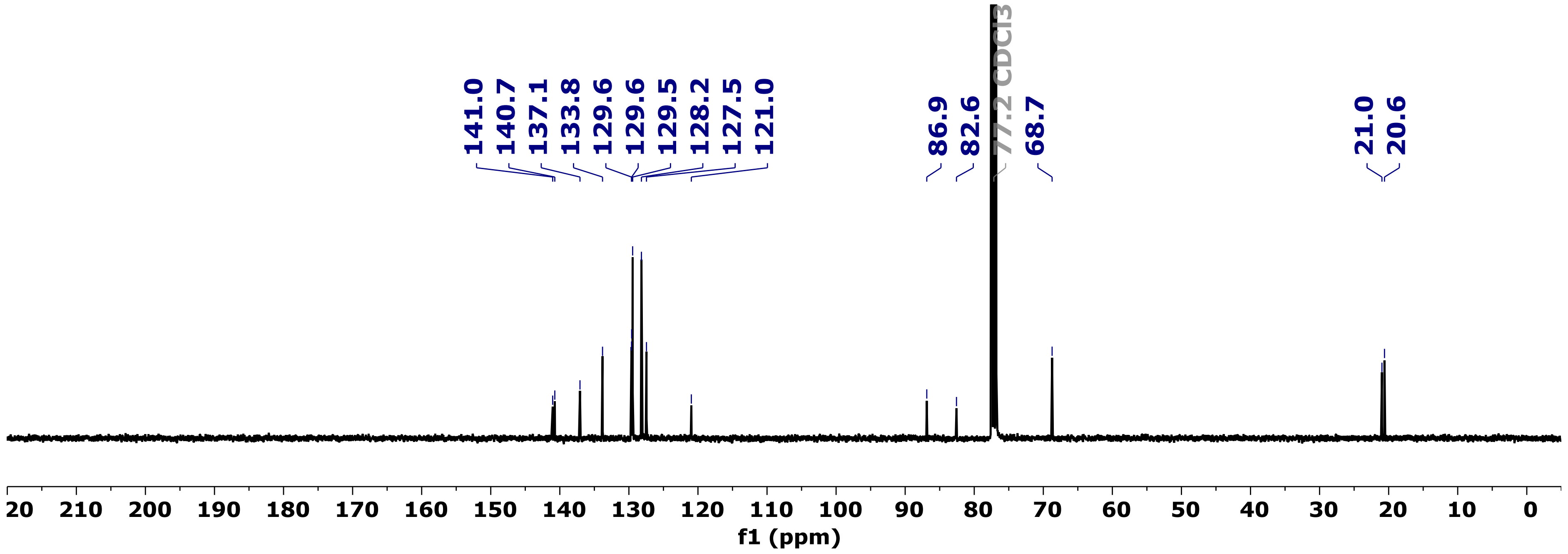

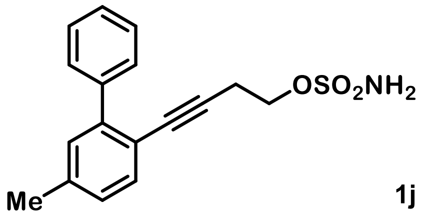

1j

^{1}H NMR (400 MHz, $CDCl_3$)

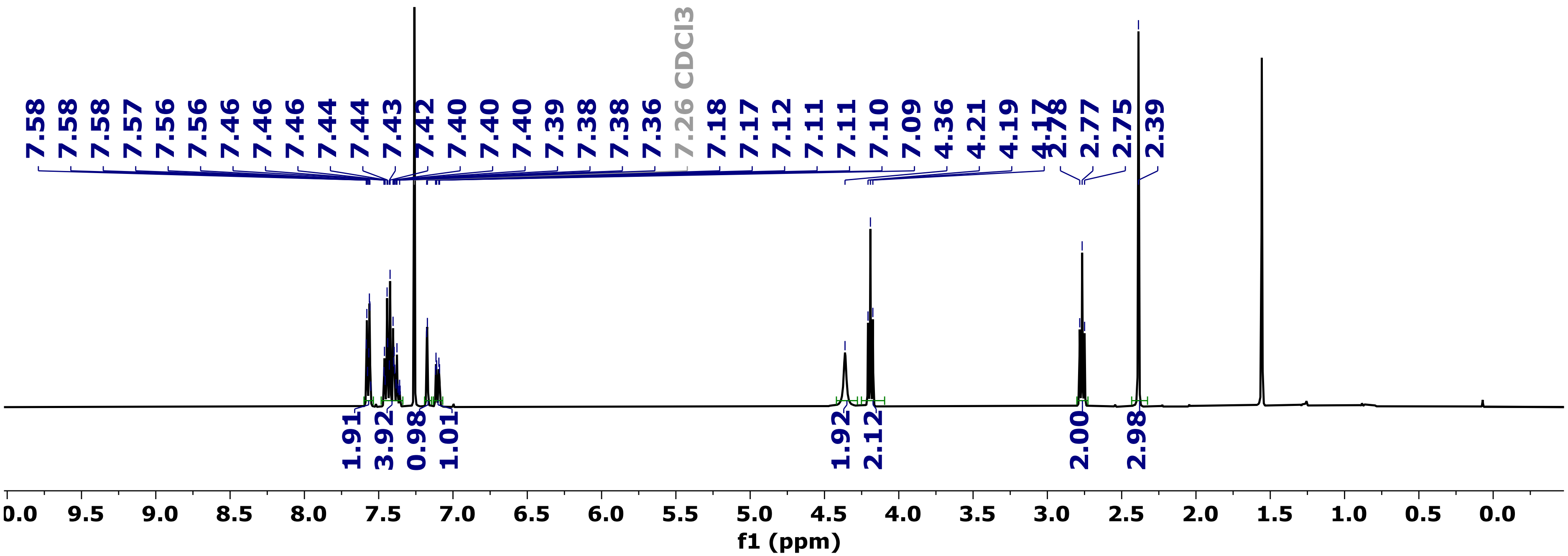

$^{13}C\{H\}$ NMR (101 MHz, $CDCl_3$)

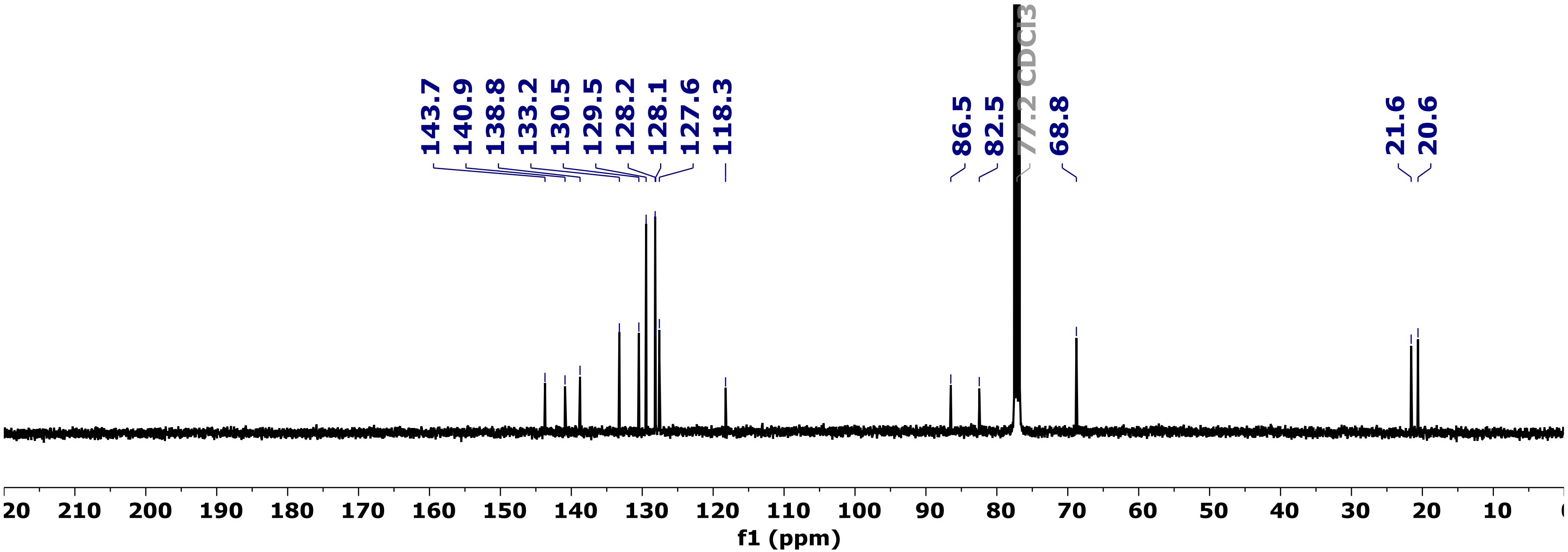

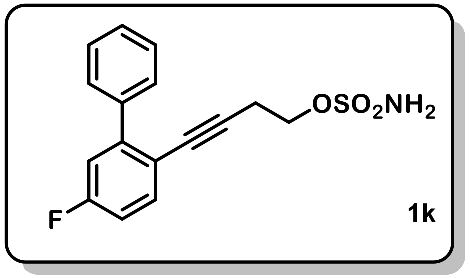

1k

^1H NMR (400 MHz, $CDCl_3$)

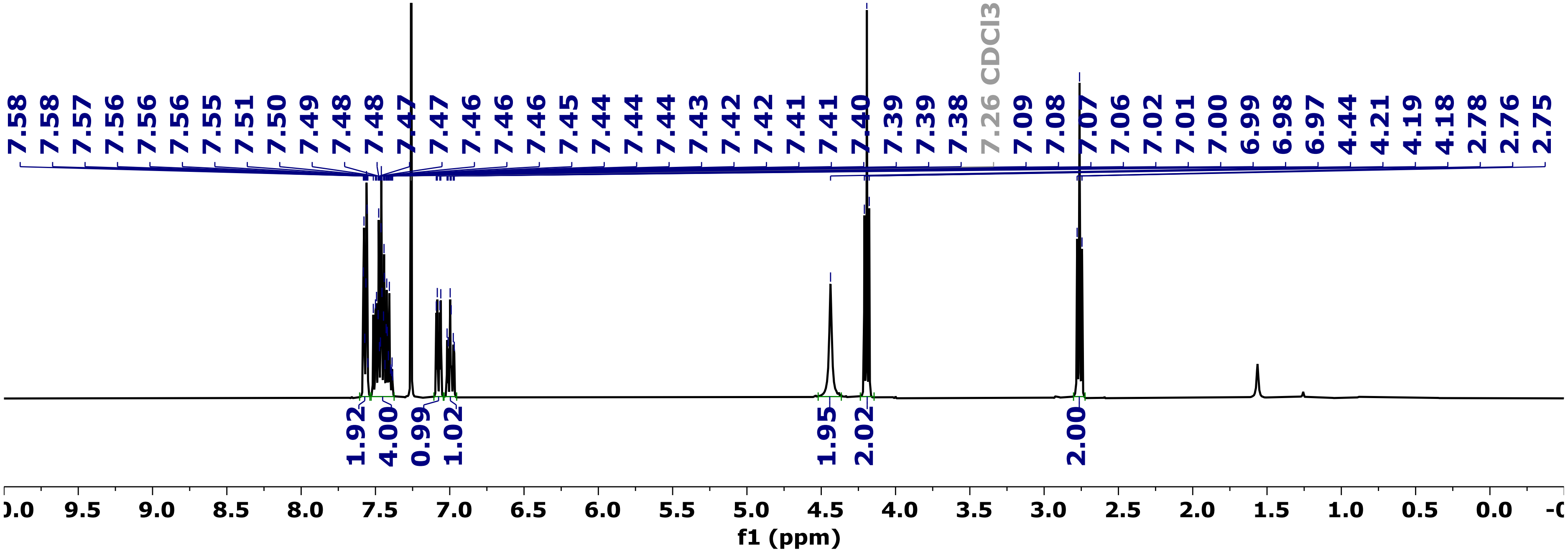

$^{13}C\{H\}$ NMR (101 MHz, $CDCl_3$)

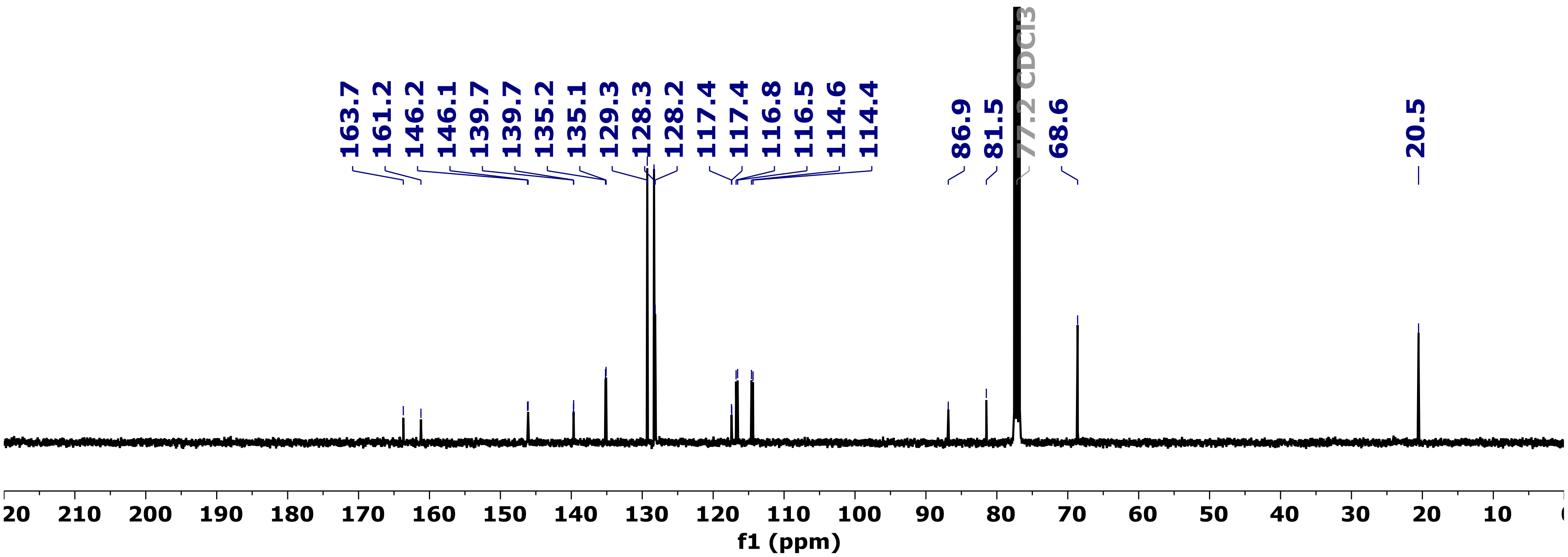

$^{19}F\{H\}$ NMR (377 MHz, $CDCl_3$)

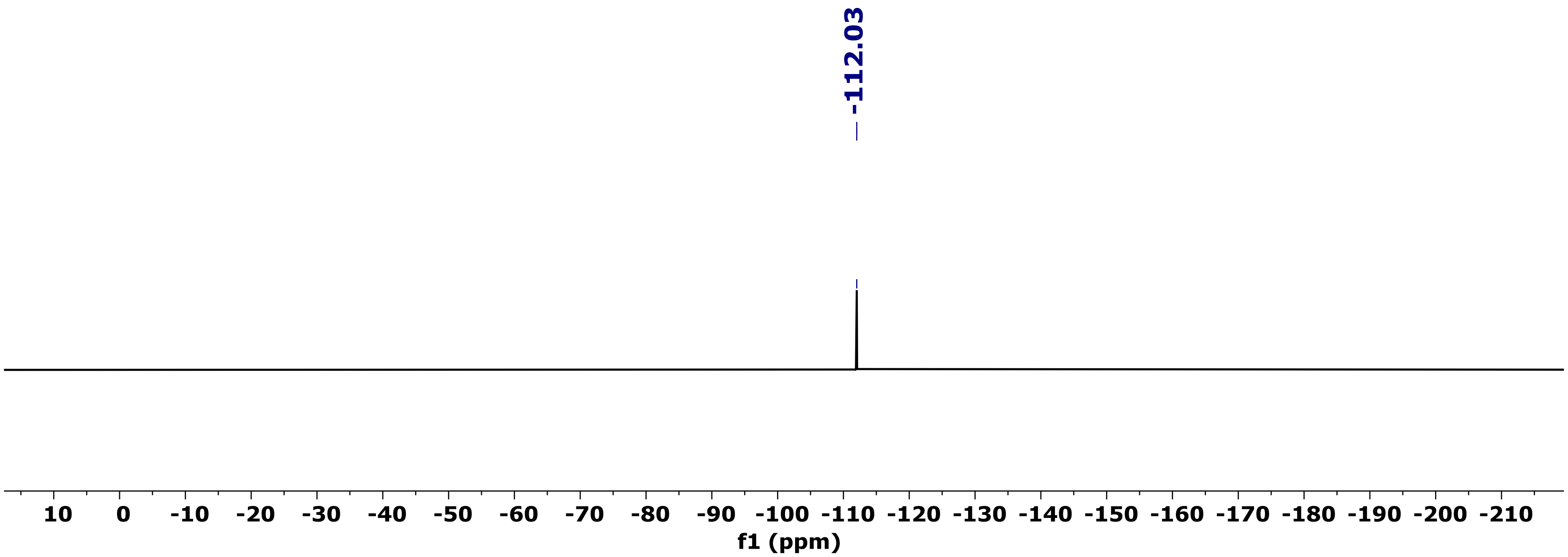

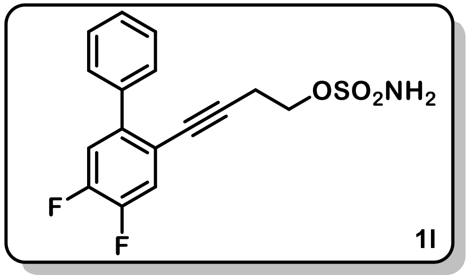

^1H NMR (400 MHz, $CDCl_3$)

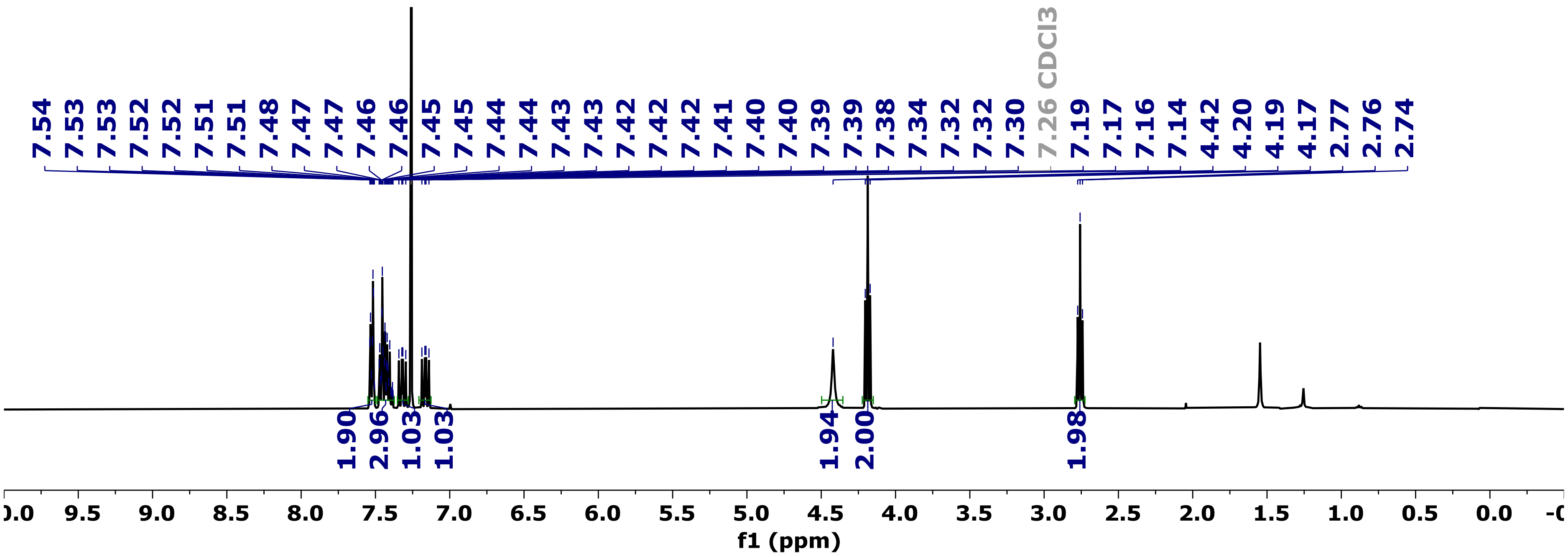

$^{13}C\{H\}$ NMR (101 MHz, $CDCl_3$)

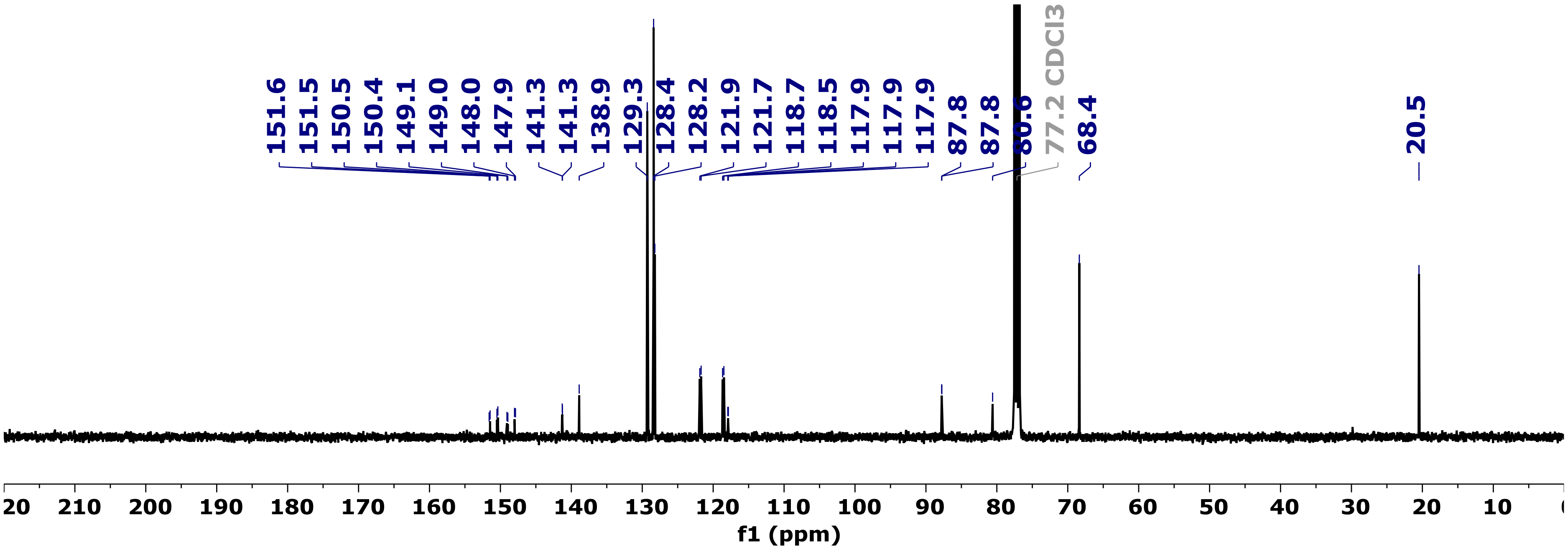

$^{19}F\{H\}$ NMR (377 MHz, $CDCl_3$)

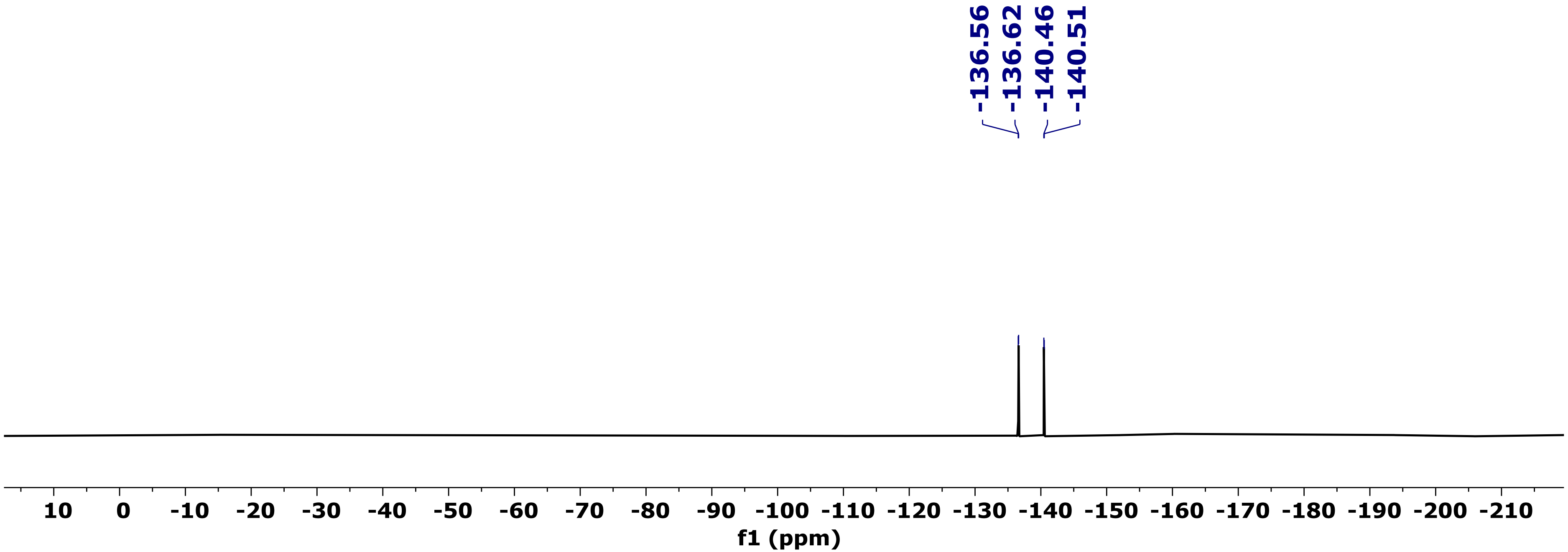

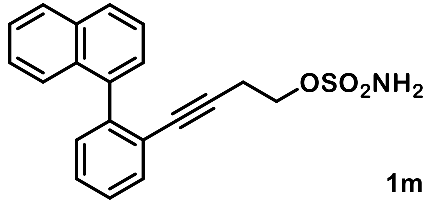

1m

^{1}H NMR (400 MHz, $CDCl_3$)

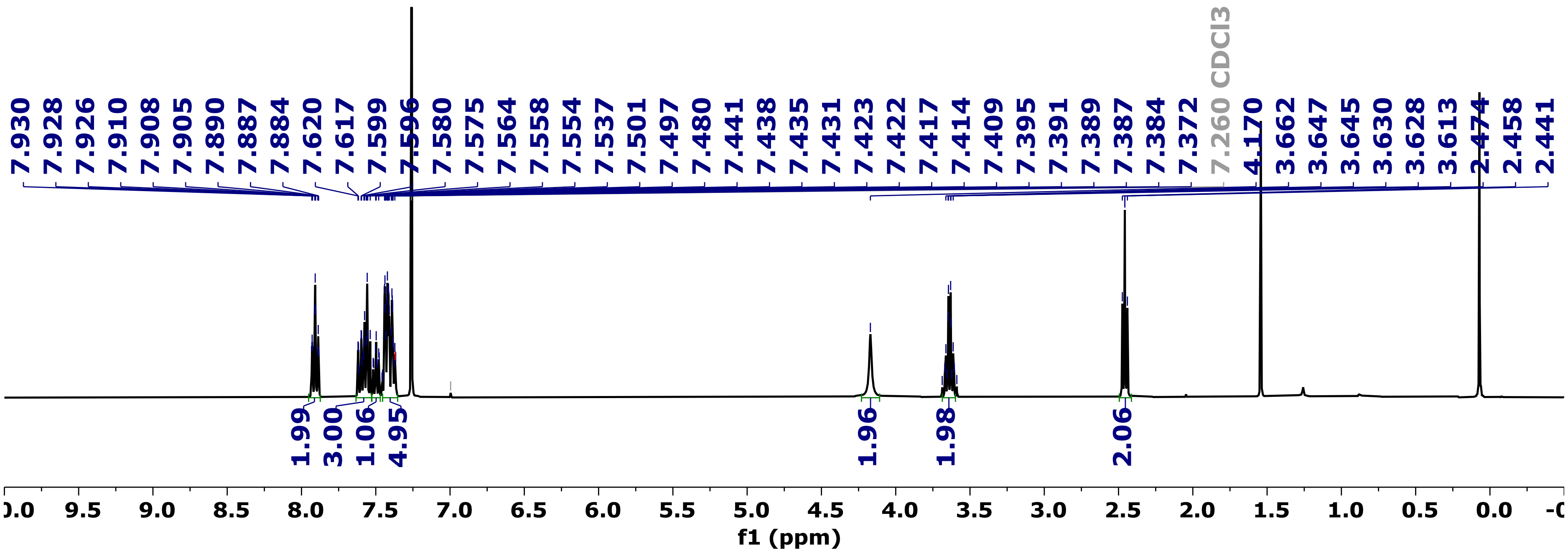

$^{13}C\{H\}$ NMR (101 MHz, $CDCl_3$)

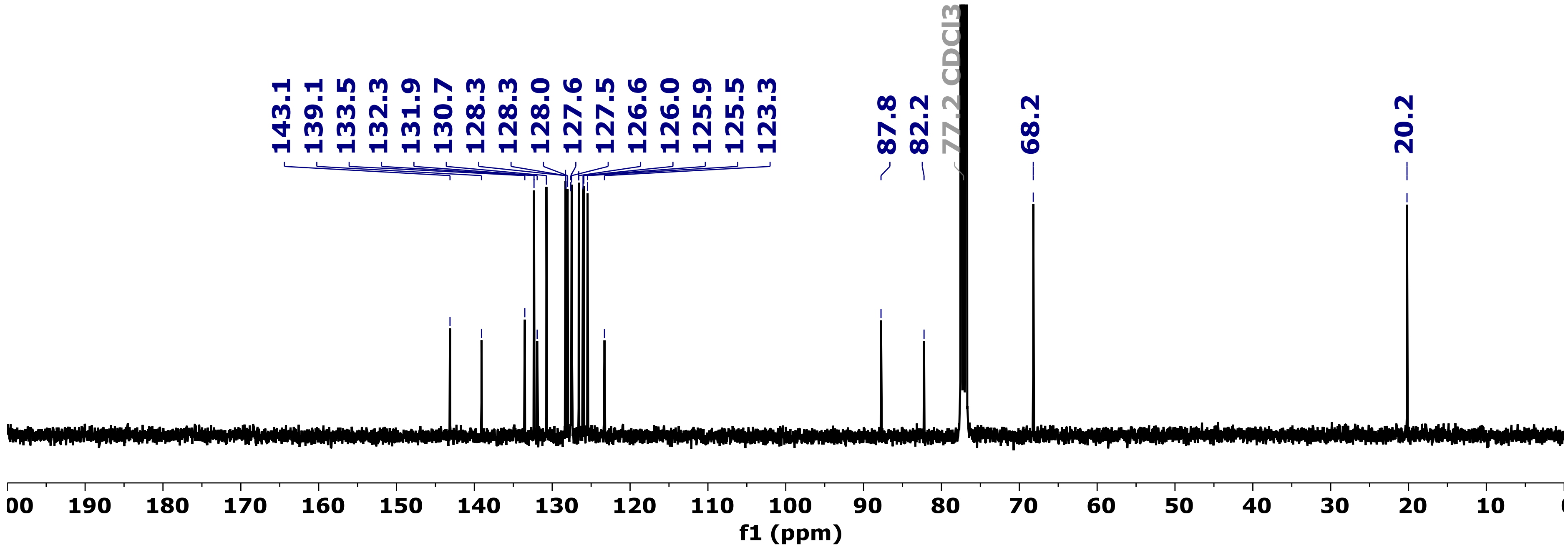

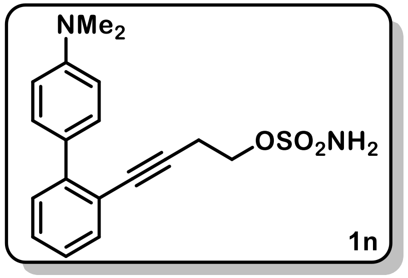

^{1}H NMR (400 MHz, $CDCl_3$)

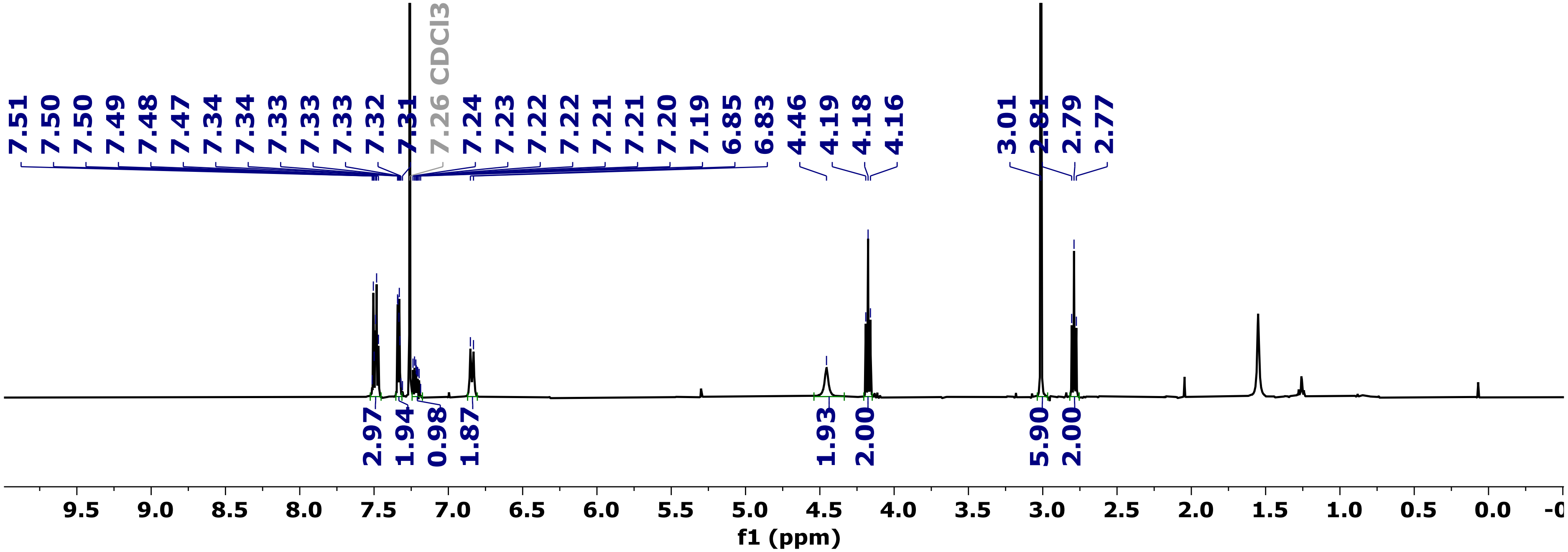

$^{13}C\{H\}$ NMR (101 MHz, $CDCl_3$)

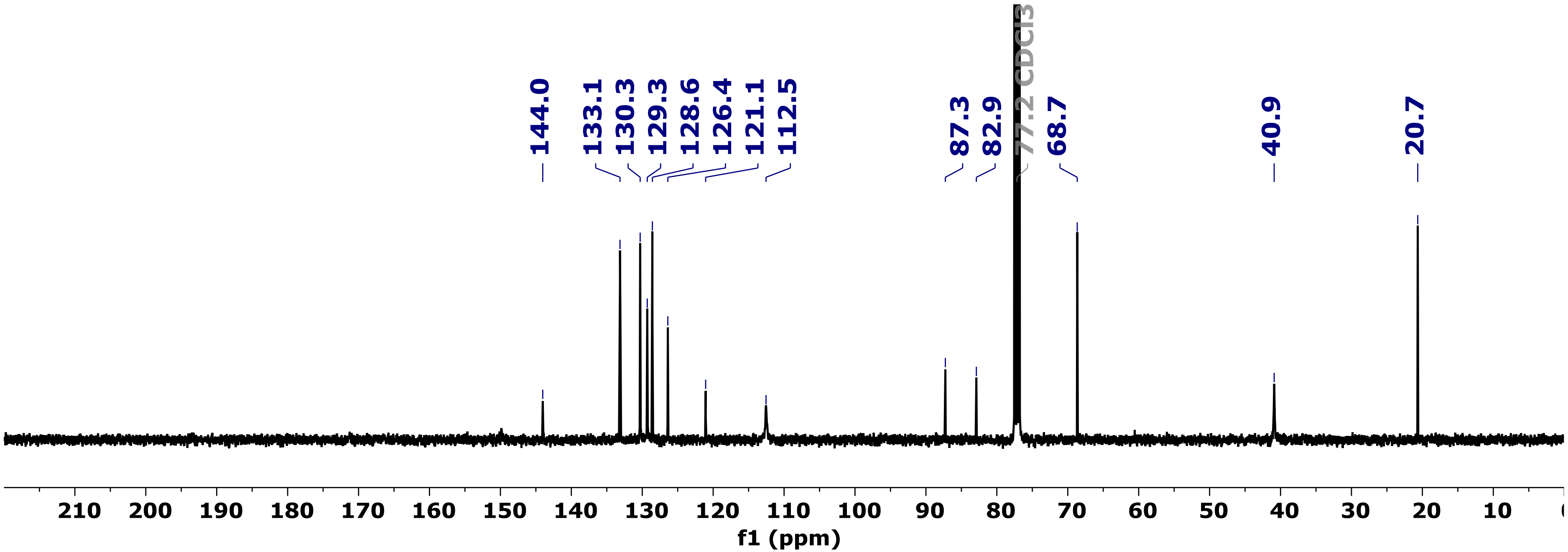

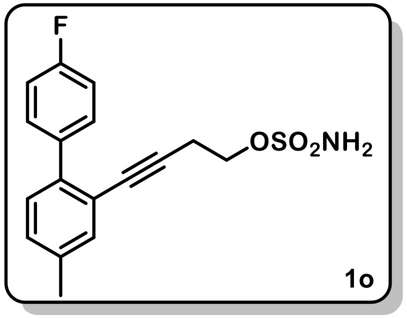

1o

^{1}H NMR (400 MHz, $CDCl_3$)

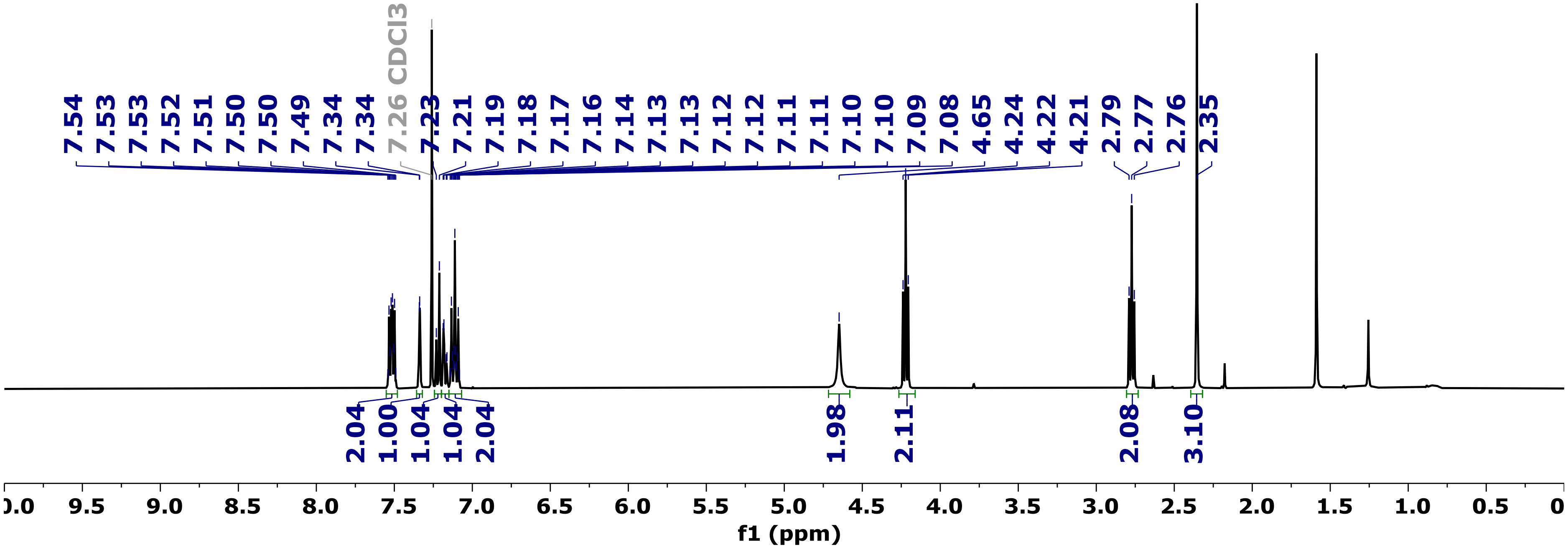

$^{13}C\{H\}$ NMR (101 MHz, $CDCl_3$)

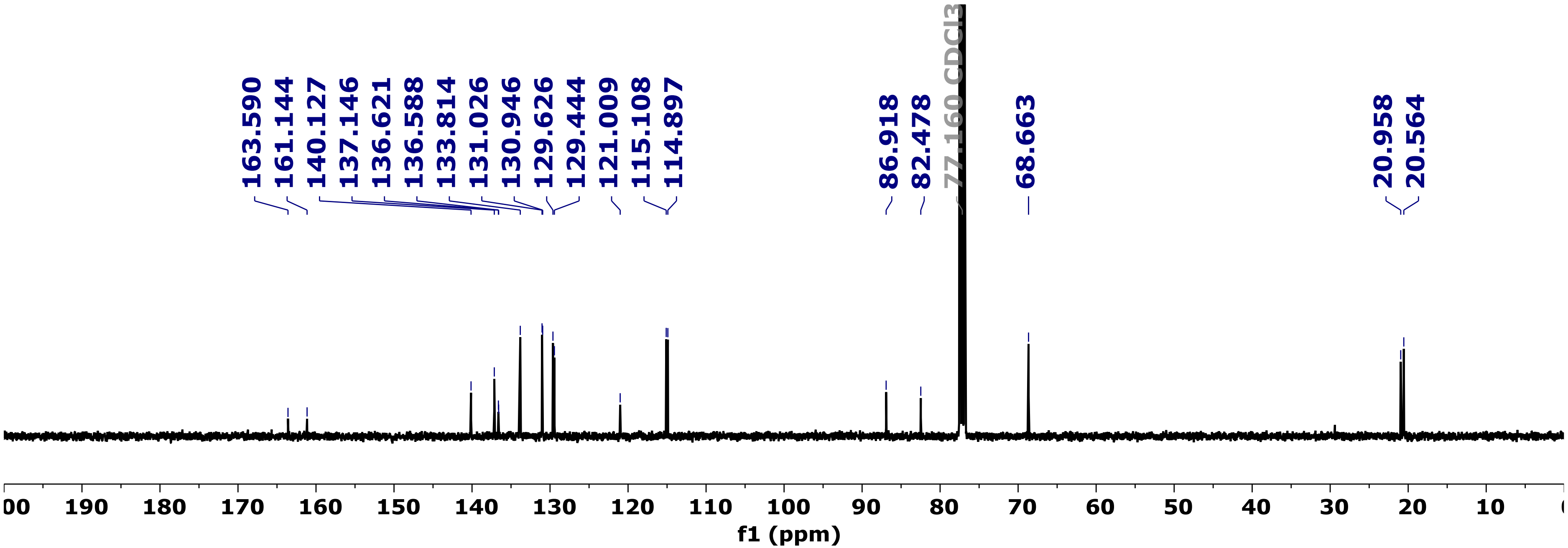

$^{19}F\{H\}$ NMR (377 MHz, $CDCl_3$)

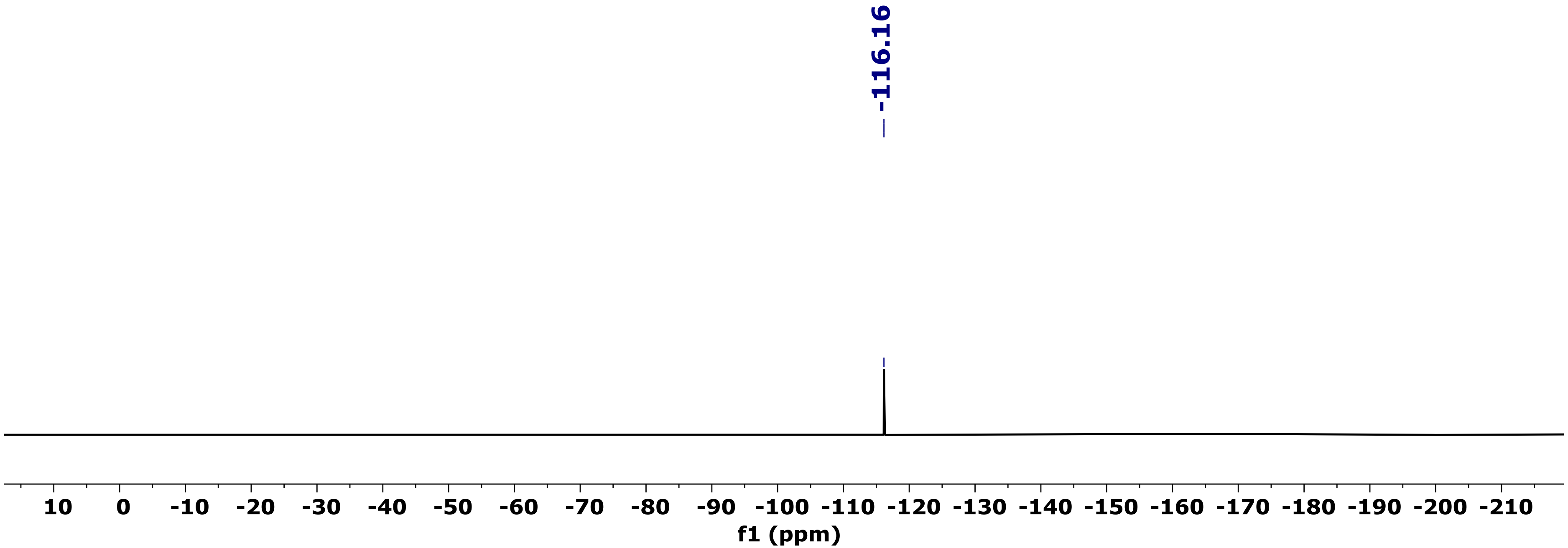

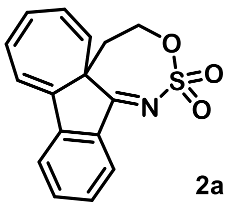

2a

^{1}H NMR (400 MHz, $CDCl_3$)

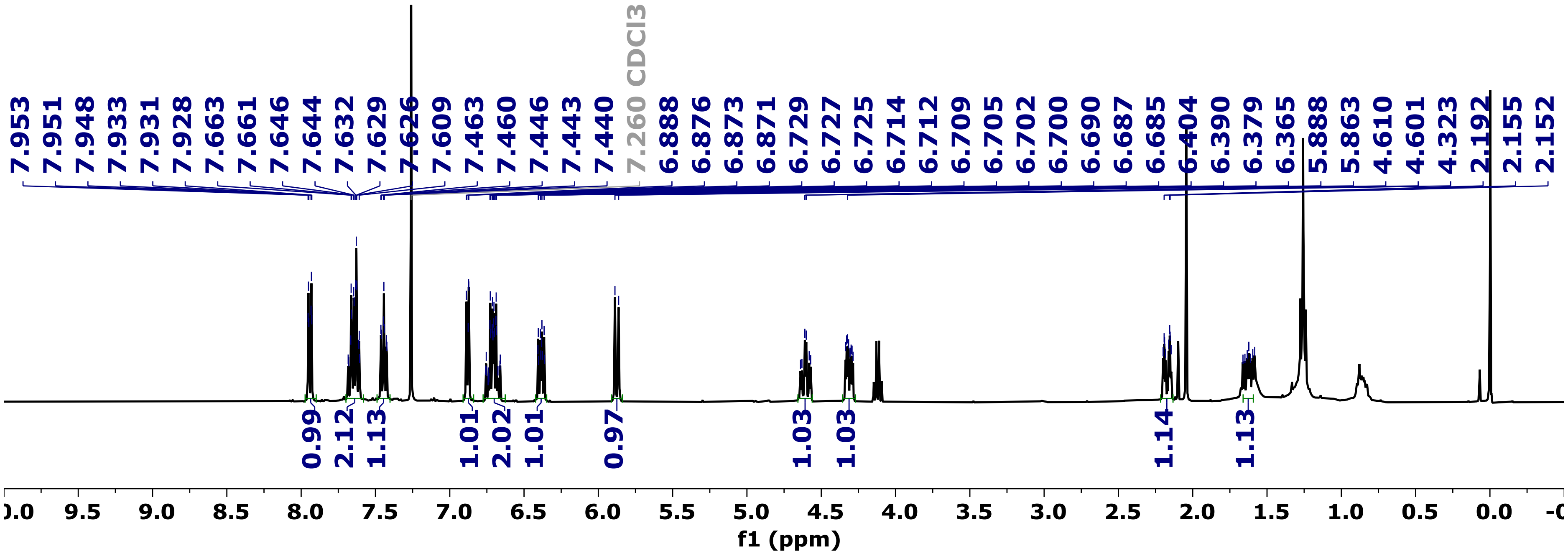

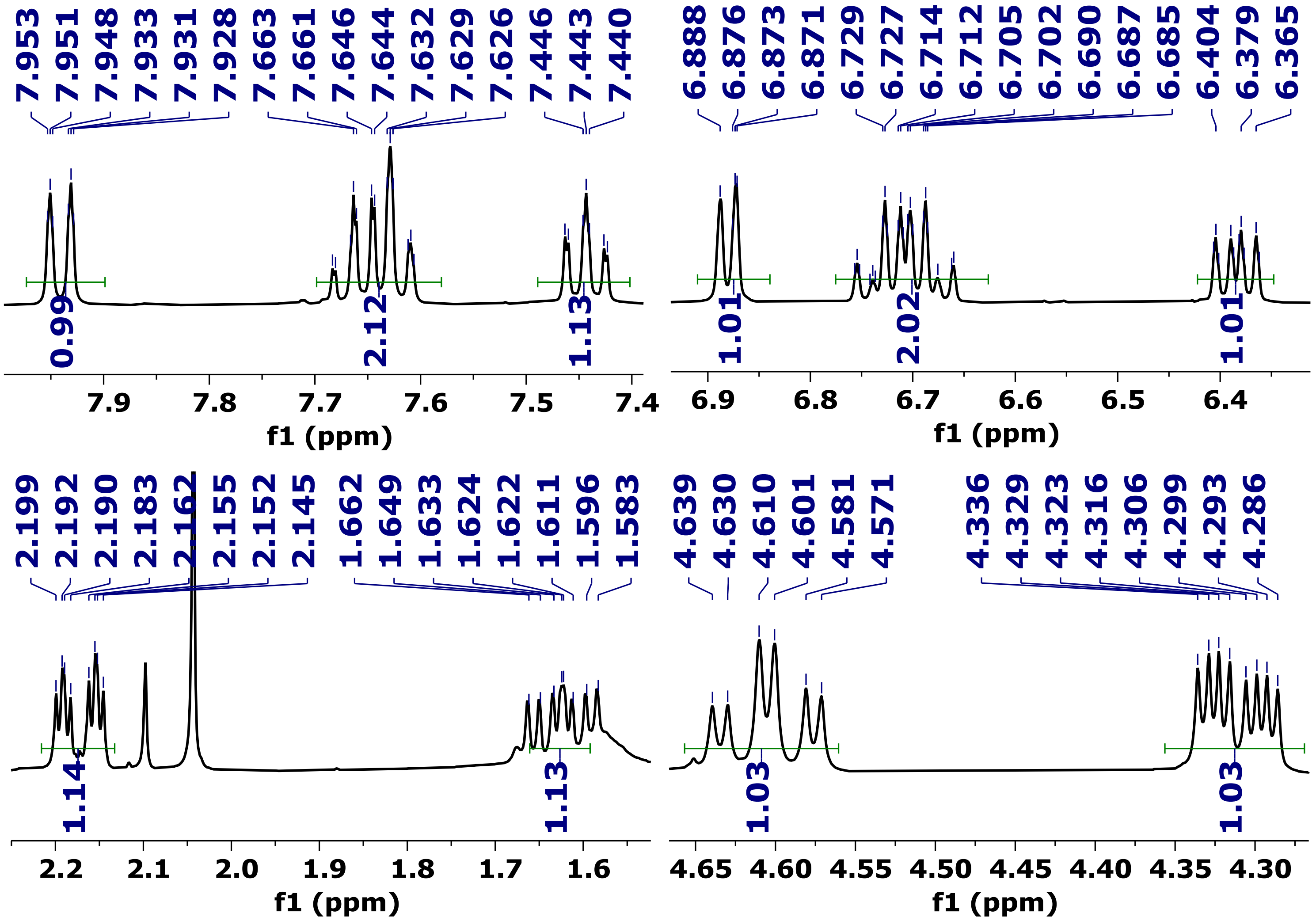
7.953 7.951 7.948 7.933 7.931 7.928 7.663 7.661 7.646 7.644 7.632 7.629 7.626 7.446 7.443 7.440
0.99
2.12
1.13
7.9 7.8 7.7 7.6 7.5 7.4
f1 (ppm)
6.888 6.876 6.873 6.871 6.729 6.727 6.714 6.712 6.705 6.702 6.690 6.687 6.685 6.404 6.379 6.365
1.01
2.02
1.01
6.9 6.8 6.7 6.6 6.5 6.4
f1 (ppm)
2.199 2.192 2.190 2.183 2.162 2.155 2.152 2.145 1.662 1.649 1.633 1.624 1.622 1.611 1.596 1.583
1.14
1.13
2.2 2.1 2.0 1.9 1.8 1.7 1.6
f1 (ppm)
4.639 4.630 4.610 4.601 4.581 4.571 4.336 4.329 4.323 4.316 4.306 4.299 4.293 4.286
1.03
1.03
4.65 4.60 4.55 4.50 4.45 4.40 4.35 4.30
f1 (ppm)

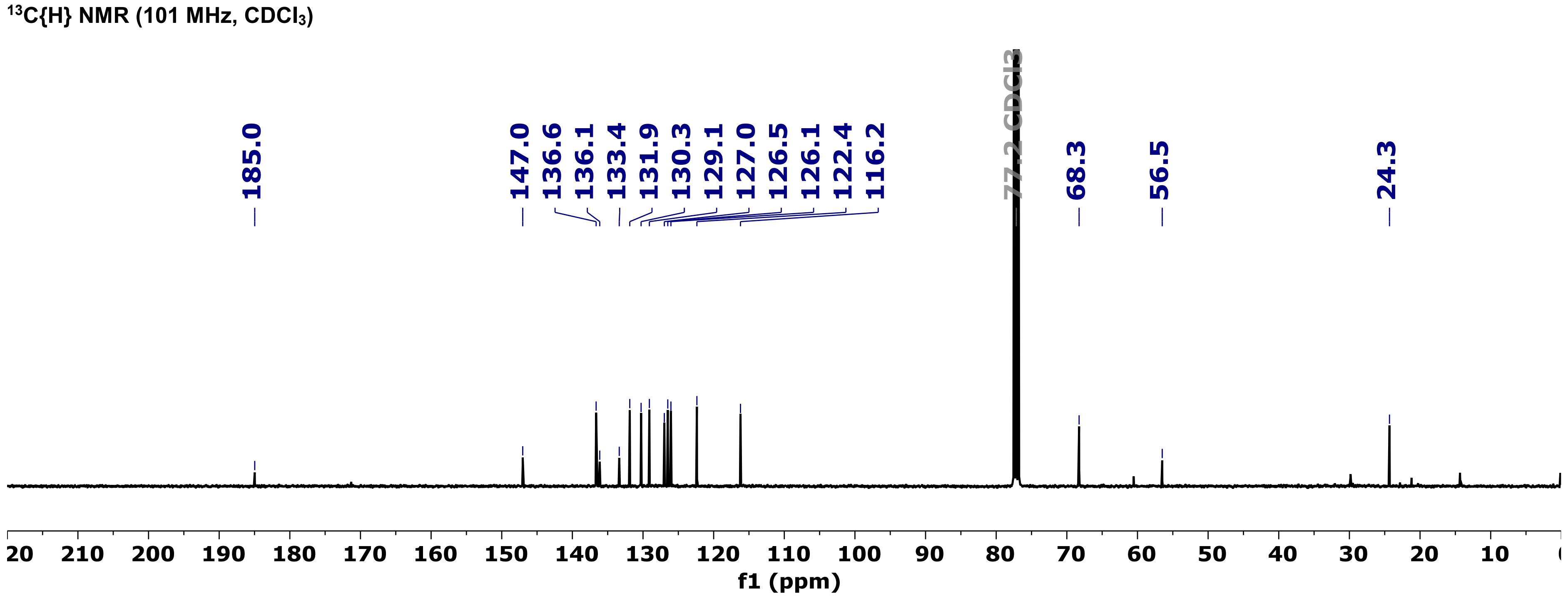

13C{H} NMR (101 MHz, CDCl3)
185.0
147.0
136.6
136.1
133.4
131.9
130.3
129.1
127.0
126.5
126.1
122.4
116.2
77.2 CDCl3
68.3
56.5
24.3
20
210
200
190
180
170
160
150
140
130
120
110
100
90
80
70
60
50
40
30
20
10
f1 (ppm)

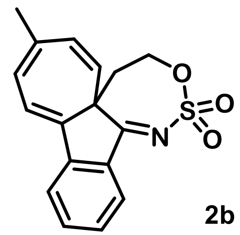

2b

^{1}H NMR (400 MHz, $CDCl_3$)

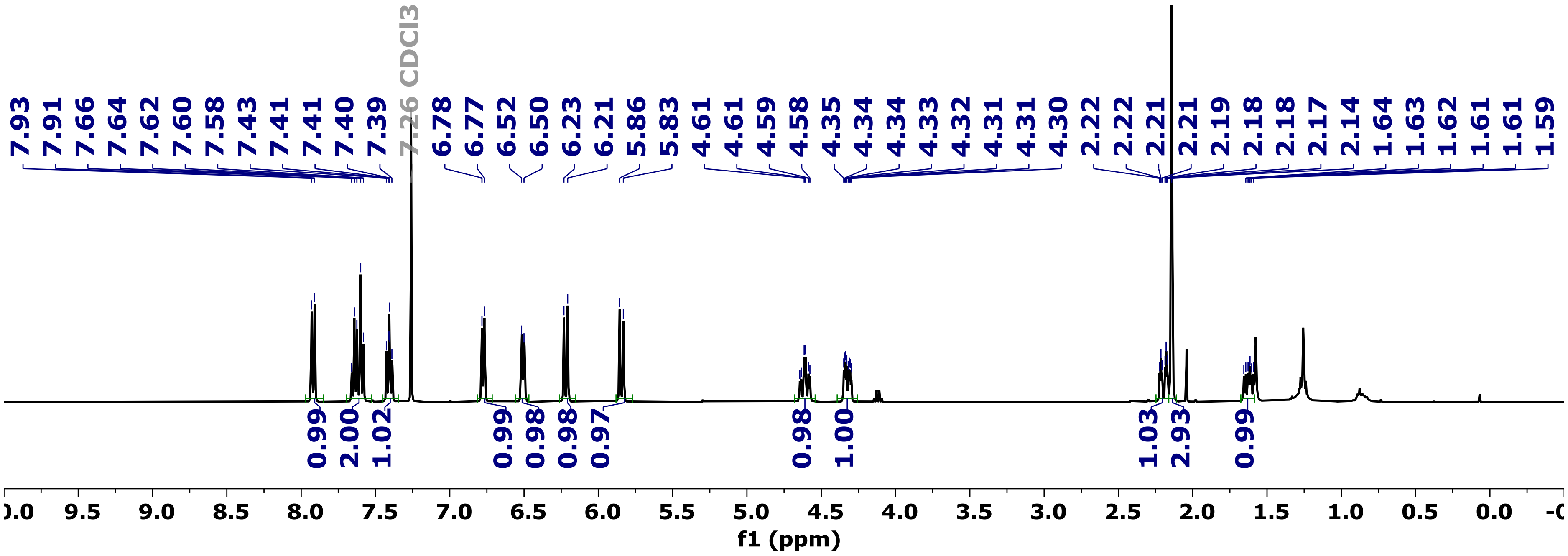

$^{13}C\{H\}$ NMR (101 MHz, $CDCl_3$)

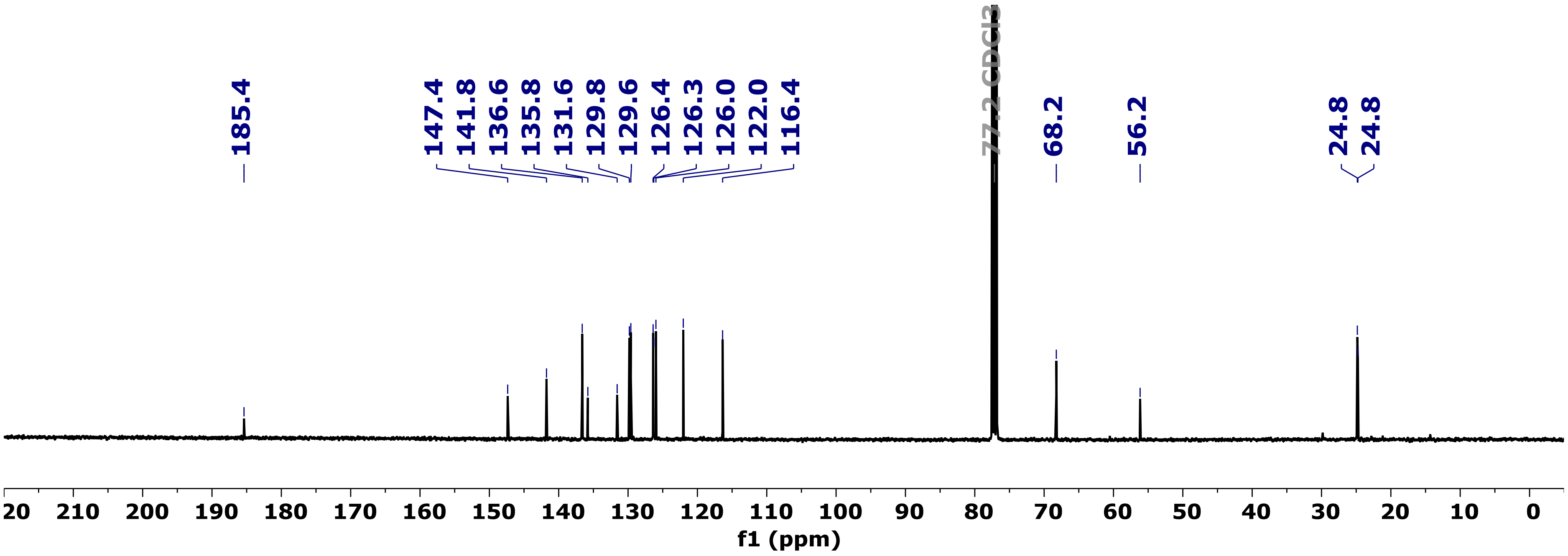

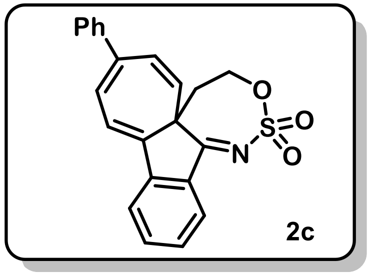

^{1}H NMR (400 MHz, $CDCl_3$)

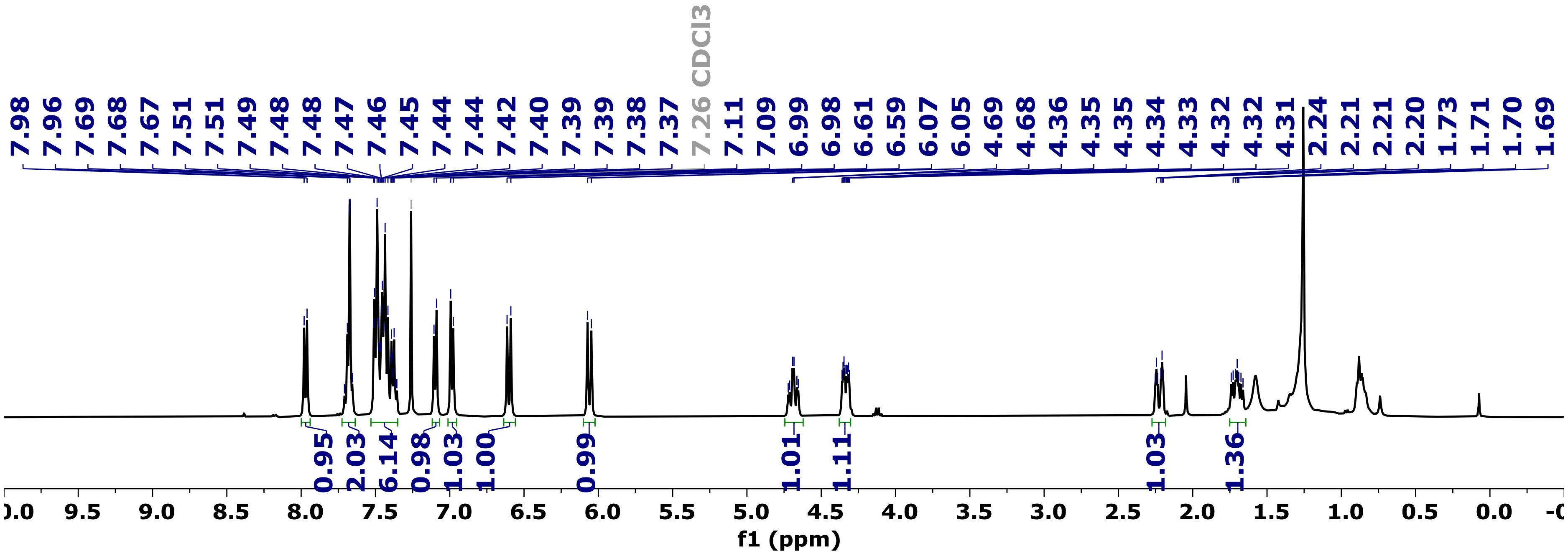

$^{13}C\{H\}$ NMR (101 MHz, $CDCl_3$)

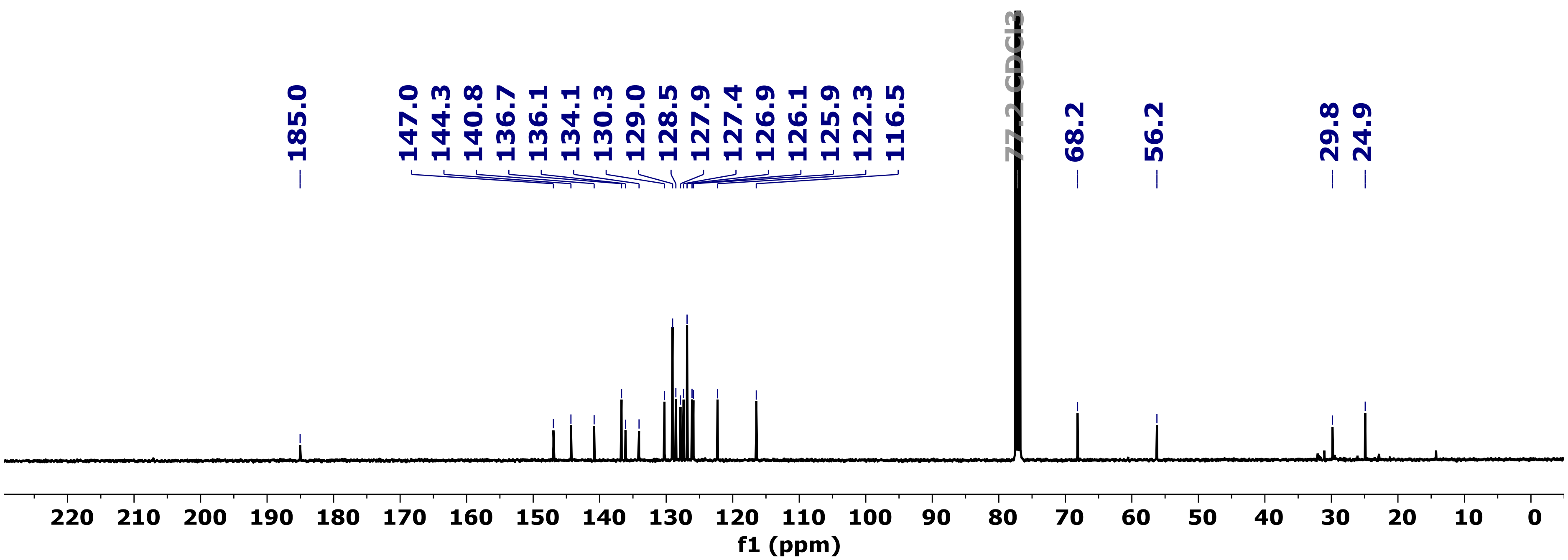

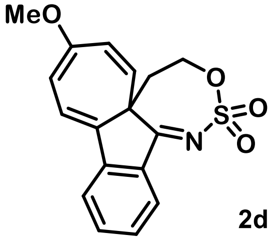

2d

^{1}H NMR (400 MHz, $CDCl_3$)

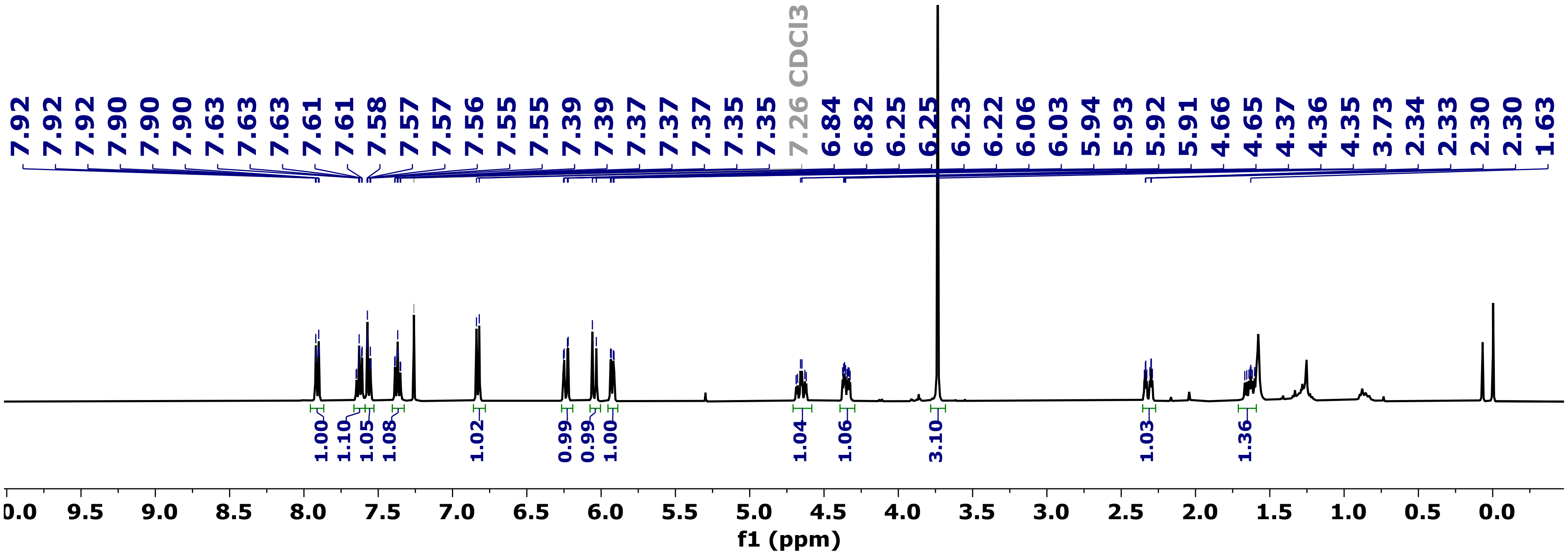

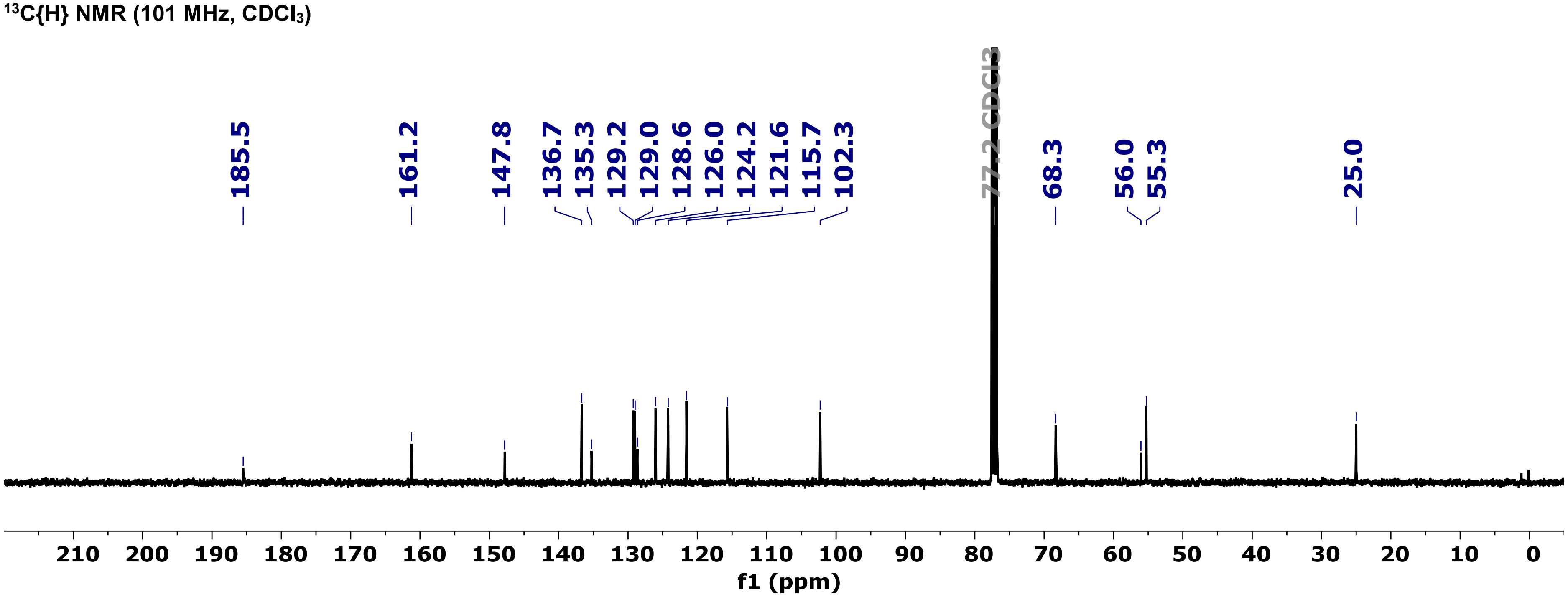

^{13}C{H} NMR (101 MHz, CDCl$_3$)
185.5
161.2
147.8
136.7
135.3
129.2
129.0
128.6
126.0
124.2
121.6
115.7
102.3
77.2 CDCl3
68.3
56.0
55.3
25.0
210 200 190 180 170 160 150 140 130 120 110 100 90 80 70 60 50 40 30 20 10 0
f1 (ppm)

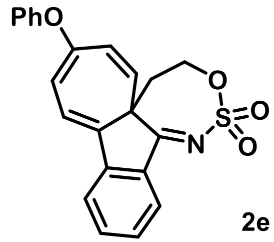

2e

^{1}H NMR (400 MHz, $CDCl_3$)

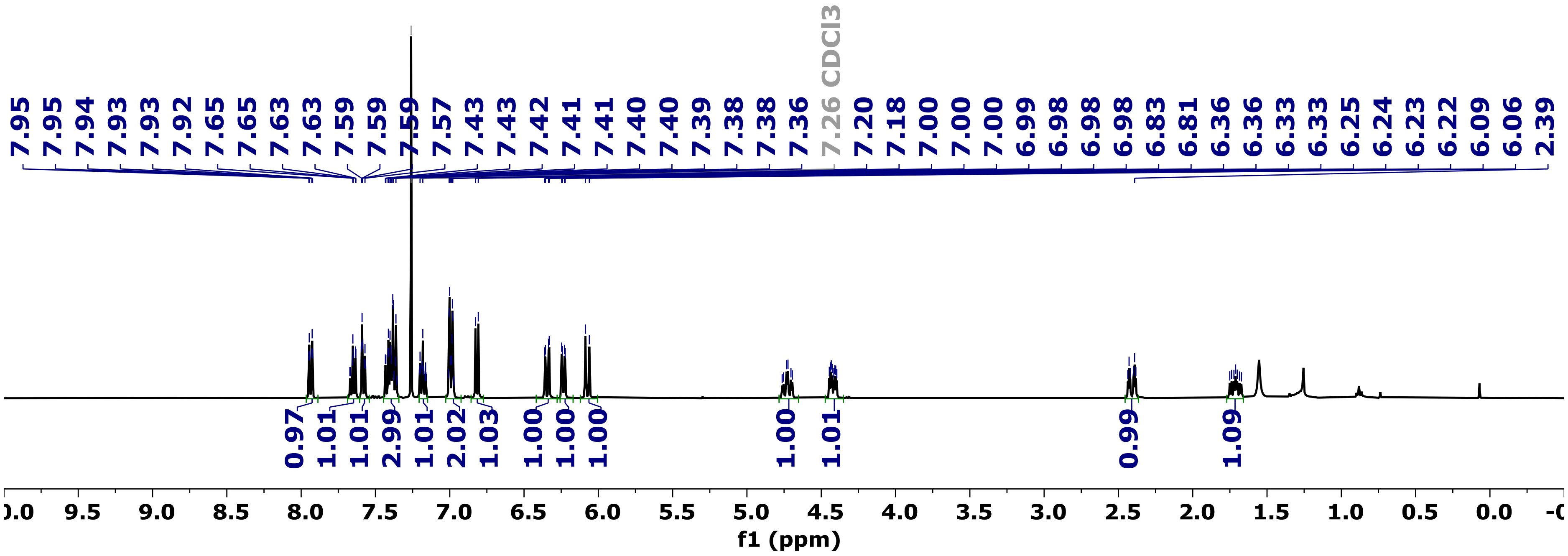

$^{13}C\{H\}$ NMR (101 MHz, $CDCl_3$)

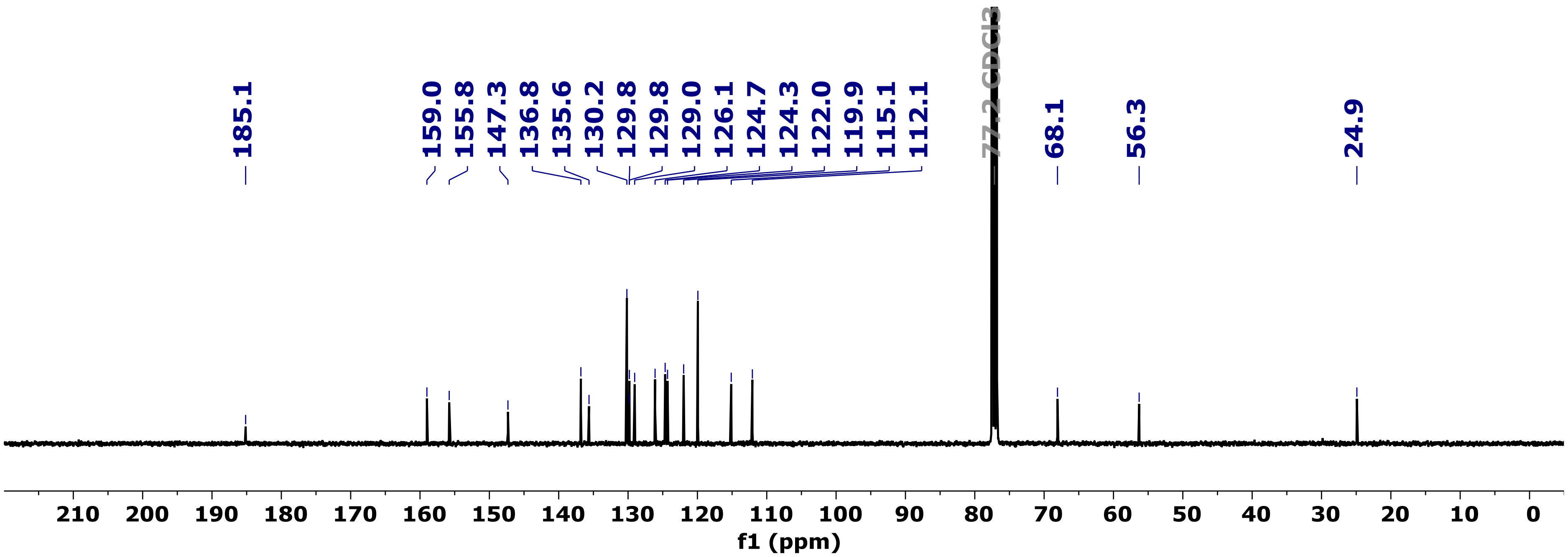

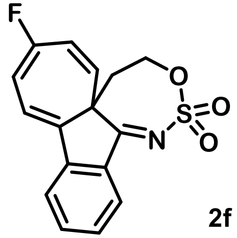

2f

^{1}H NMR (400 MHz, $CDCl_3$)

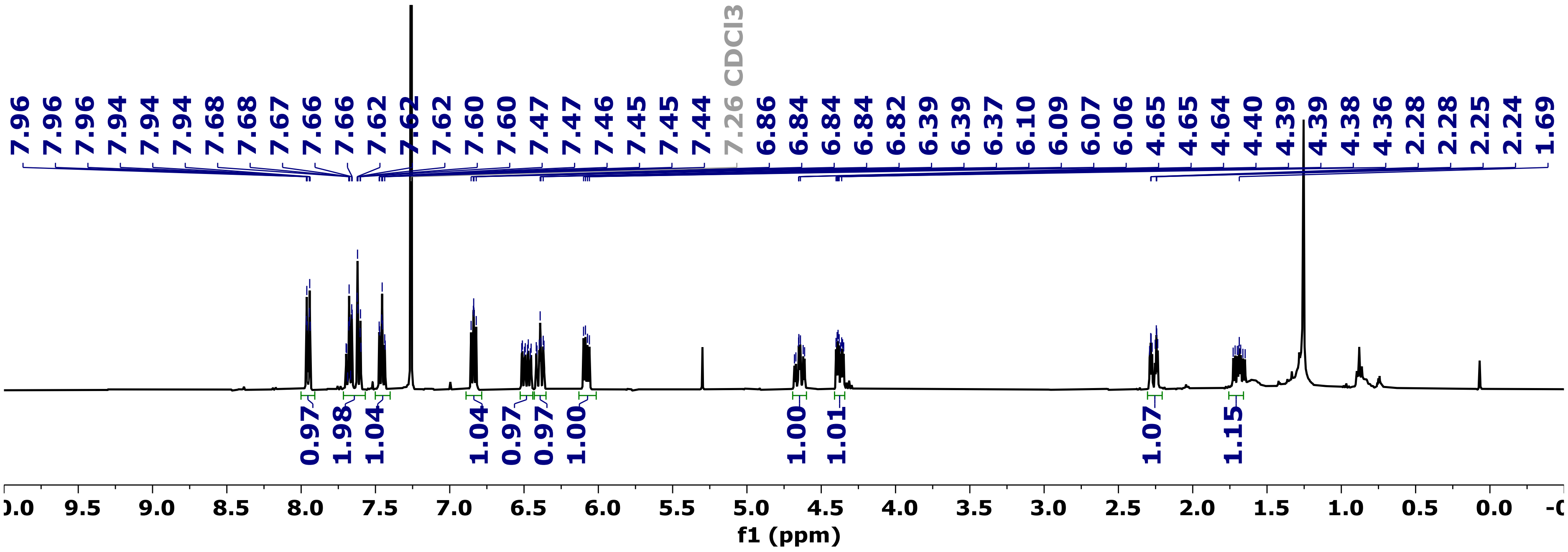

$^{13}C\{H\}$ NMR (101 MHz, $CDCl_3$)

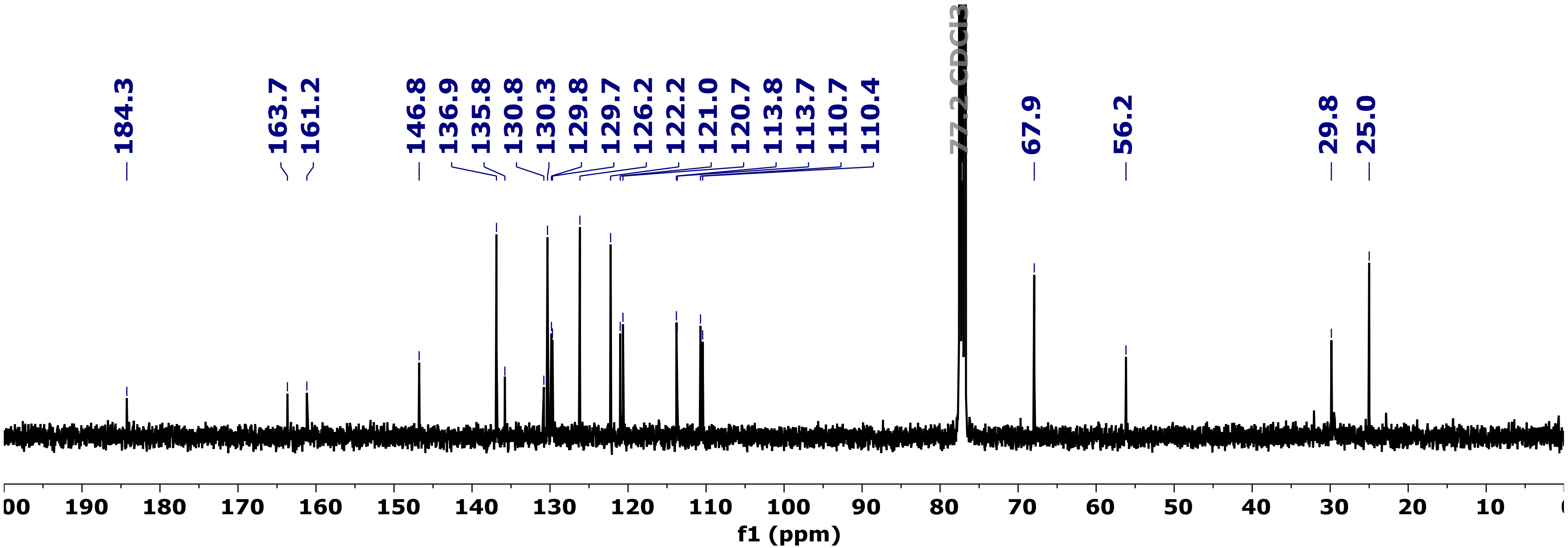

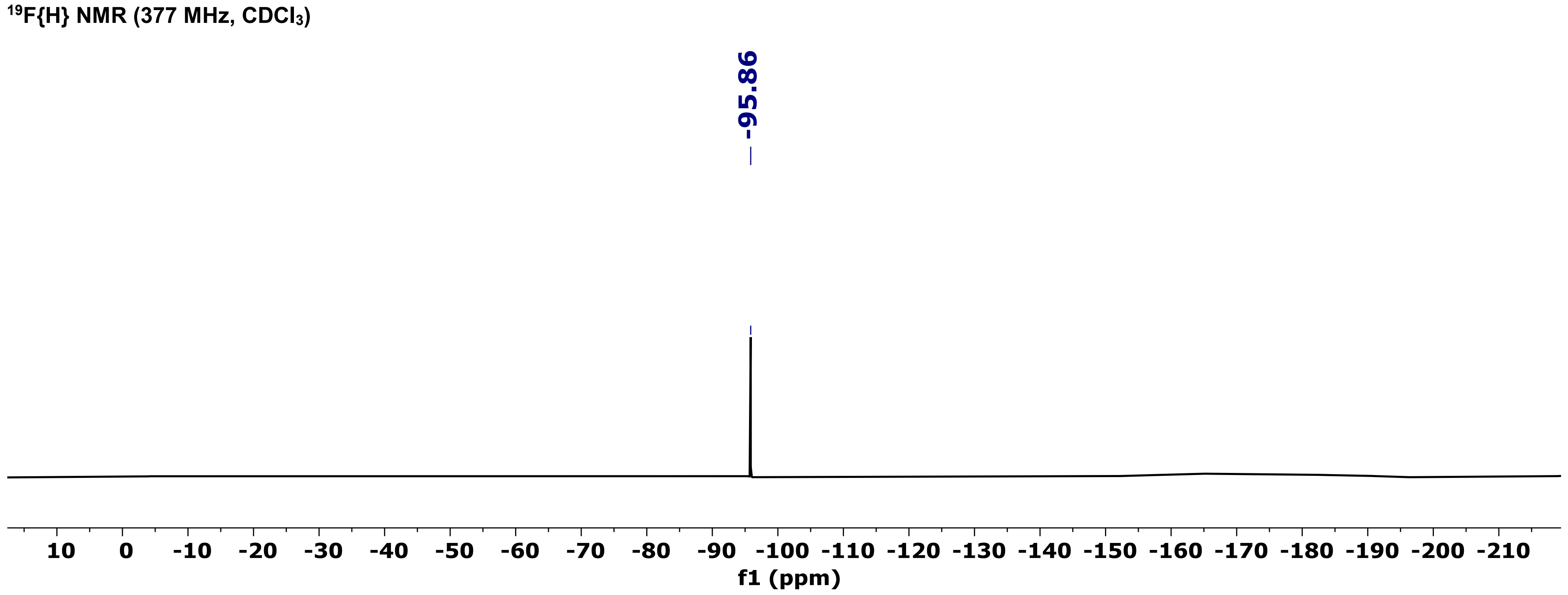

$^{19}F\{H\}$ NMR (377 MHz, $CDCl_3$)
-95.86
10 0 -10 -20 -30 -40 -50 -60 -70 -80 -90 -100 -110 -120 -130 -140 -150 -160 -170 -180 -190 -200 -210
f1 (ppm)

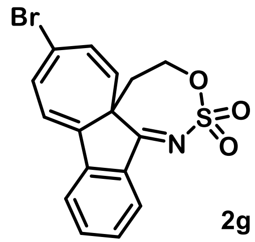

2g

^1H NMR (400 MHz, $CDCl_3$)

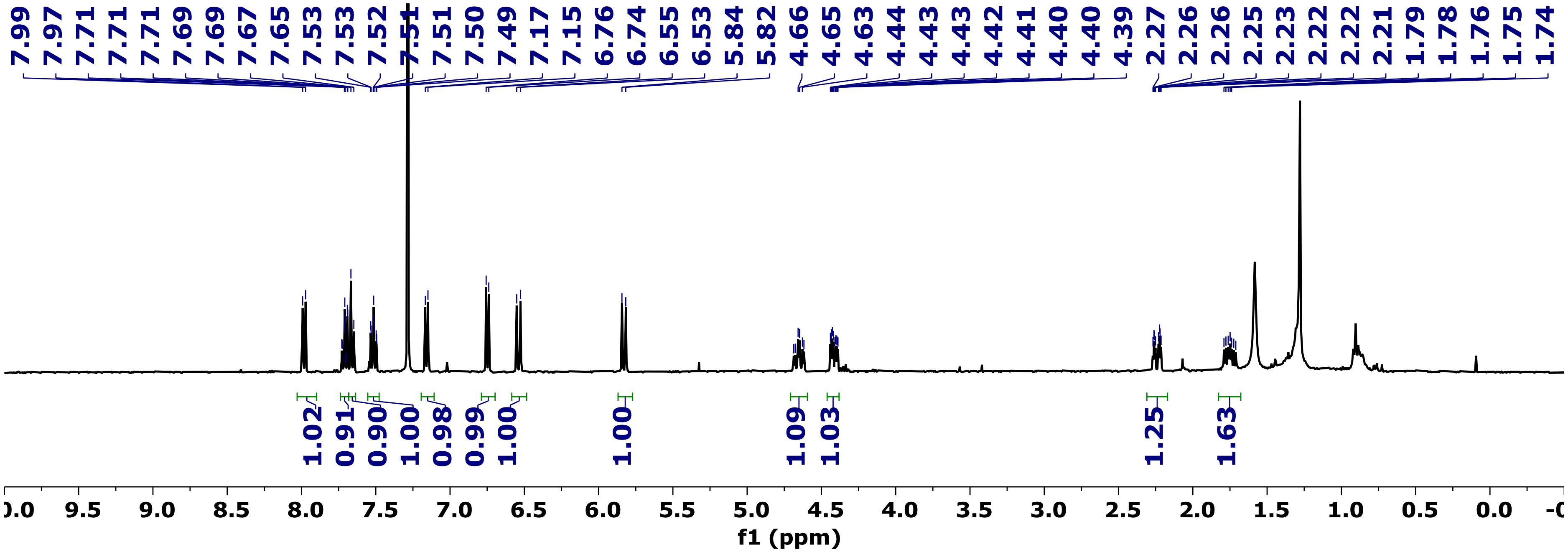

$^{13}C\{H\}$ NMR (101 MHz, $CDCl_3$)

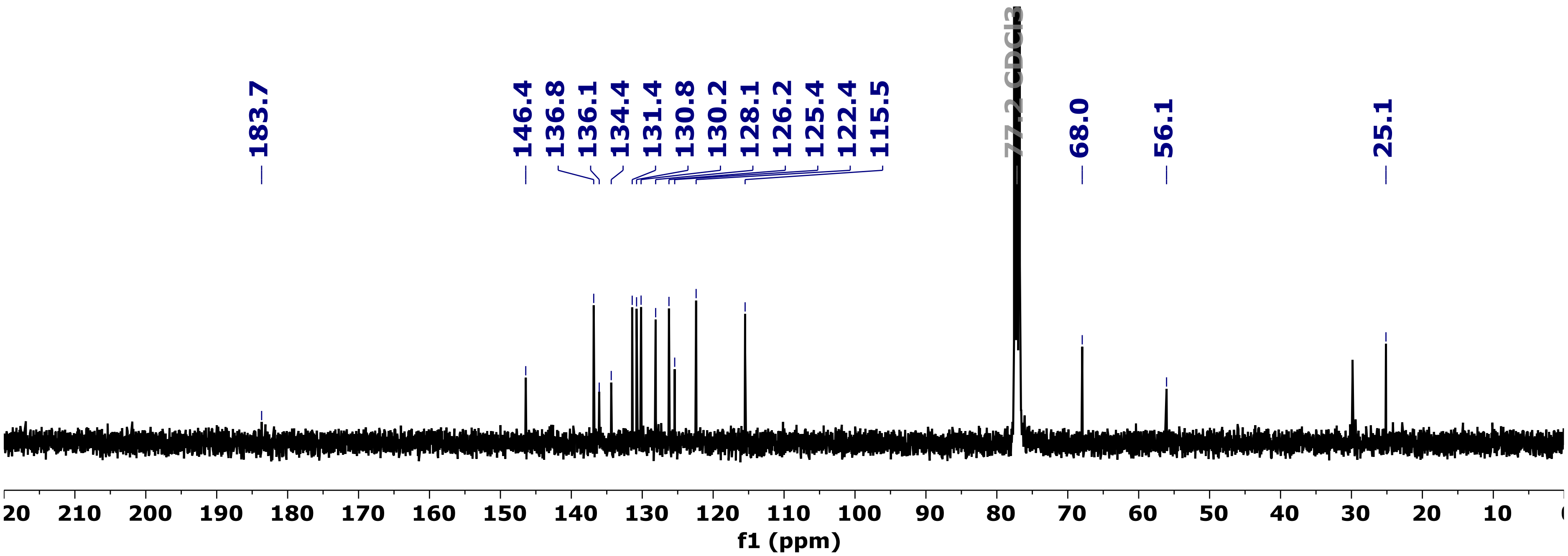

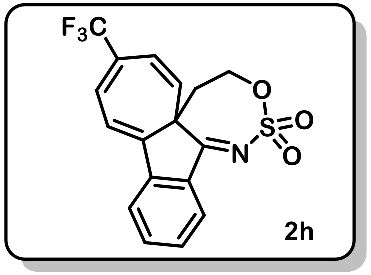

^1H NMR (400 MHz, $CDCl_3$)

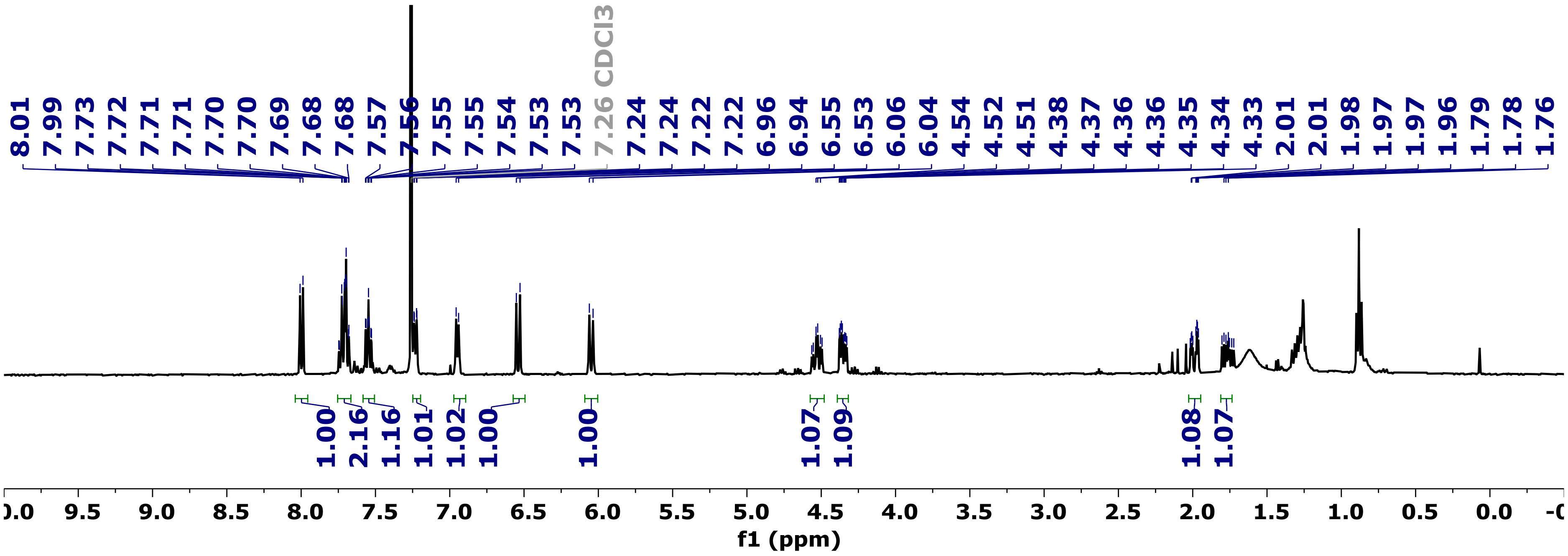

$^{13}C\{H\}$ NMR (101 MHz, $CDCl_3$)

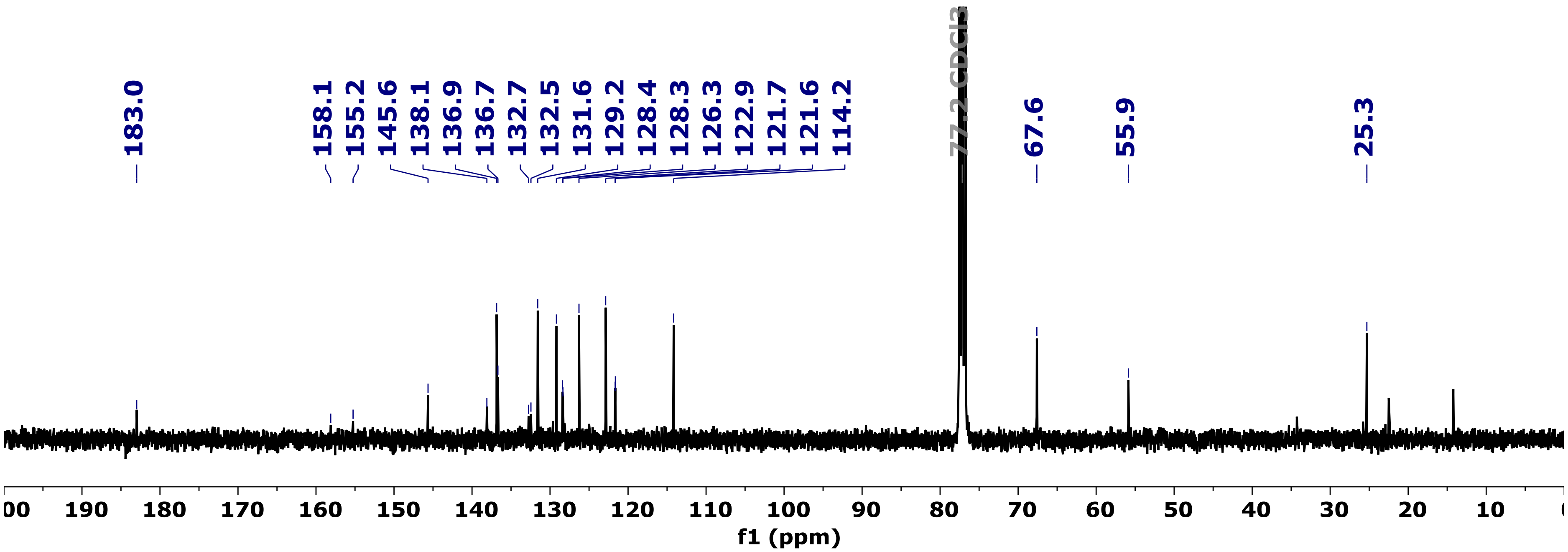

$^{19}F\{H\}$ NMR (377 MHz, $CDCl_3$)

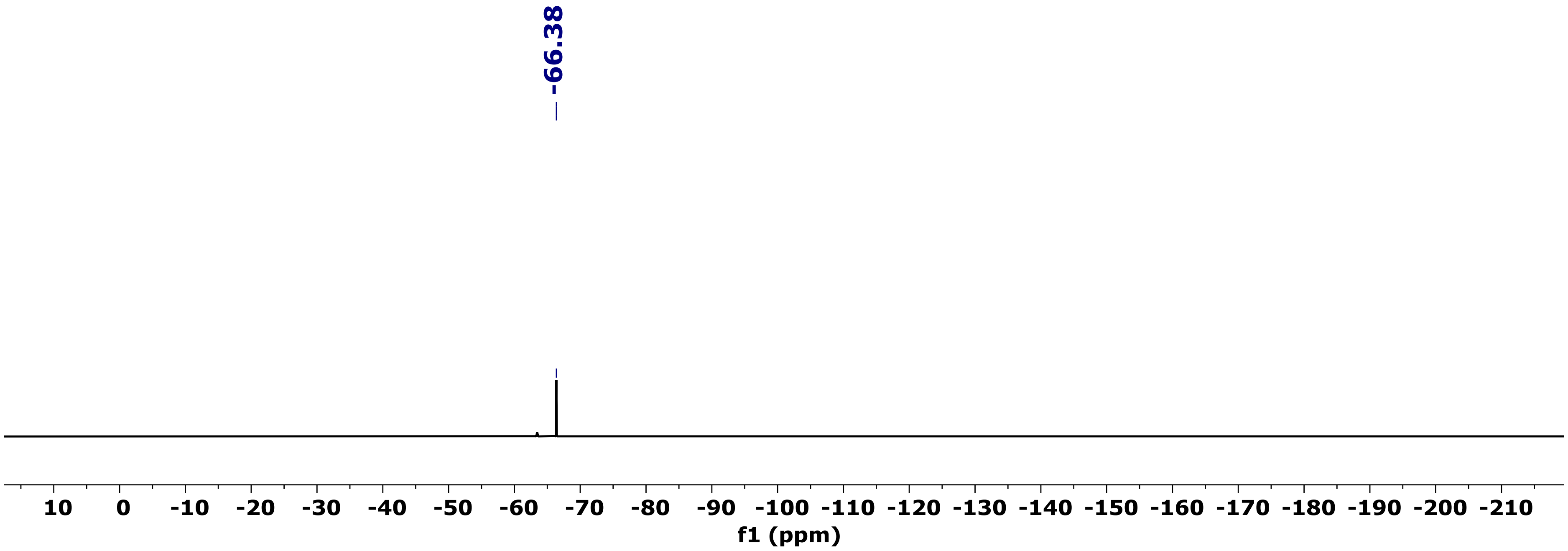

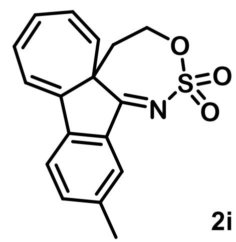

2i

^{1}H NMR (400 MHz, $CDCl_3$)

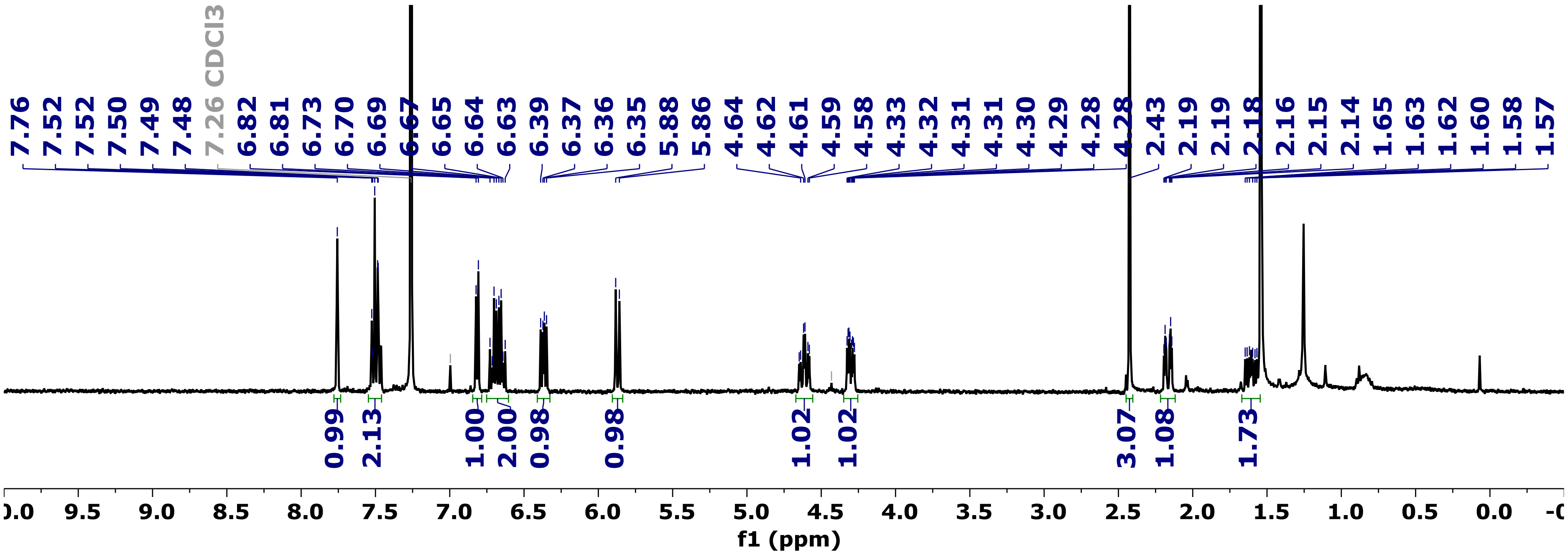

$^{13}C\{H\}$ NMR (101 MHz, $CDCl_3$)

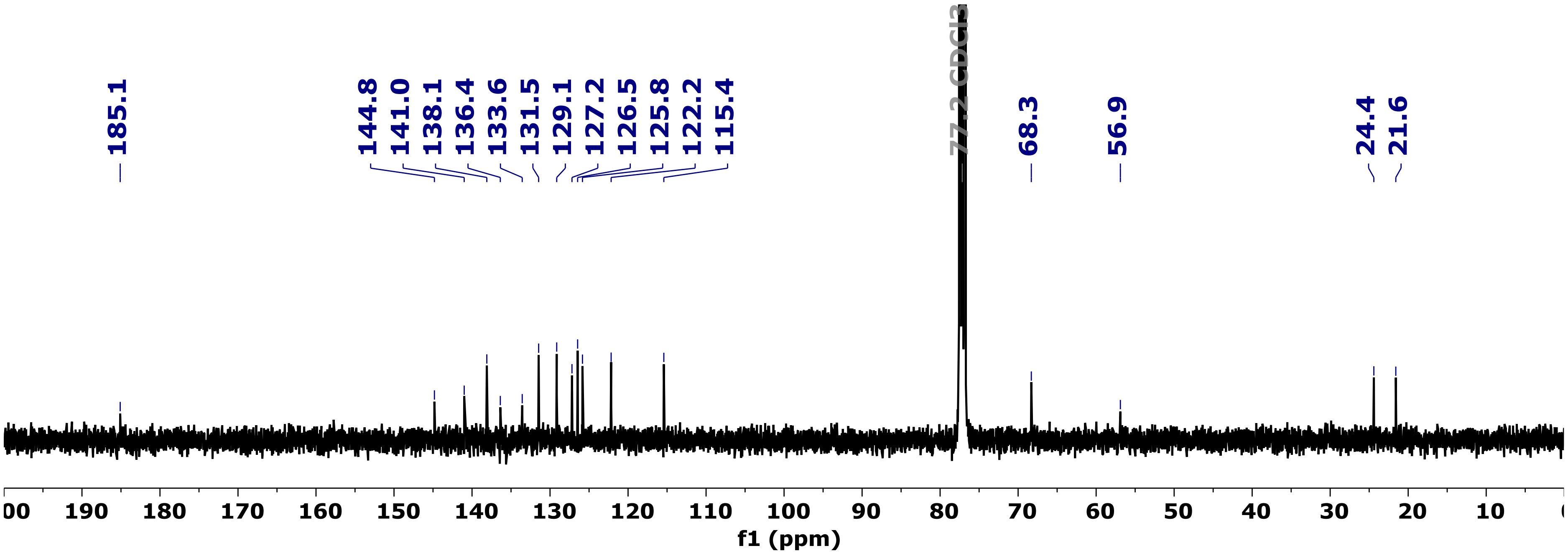

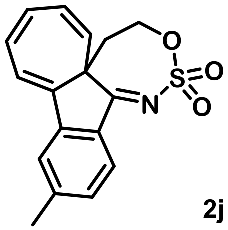

2j

^{1}H NMR (400 MHz, CDCl3)

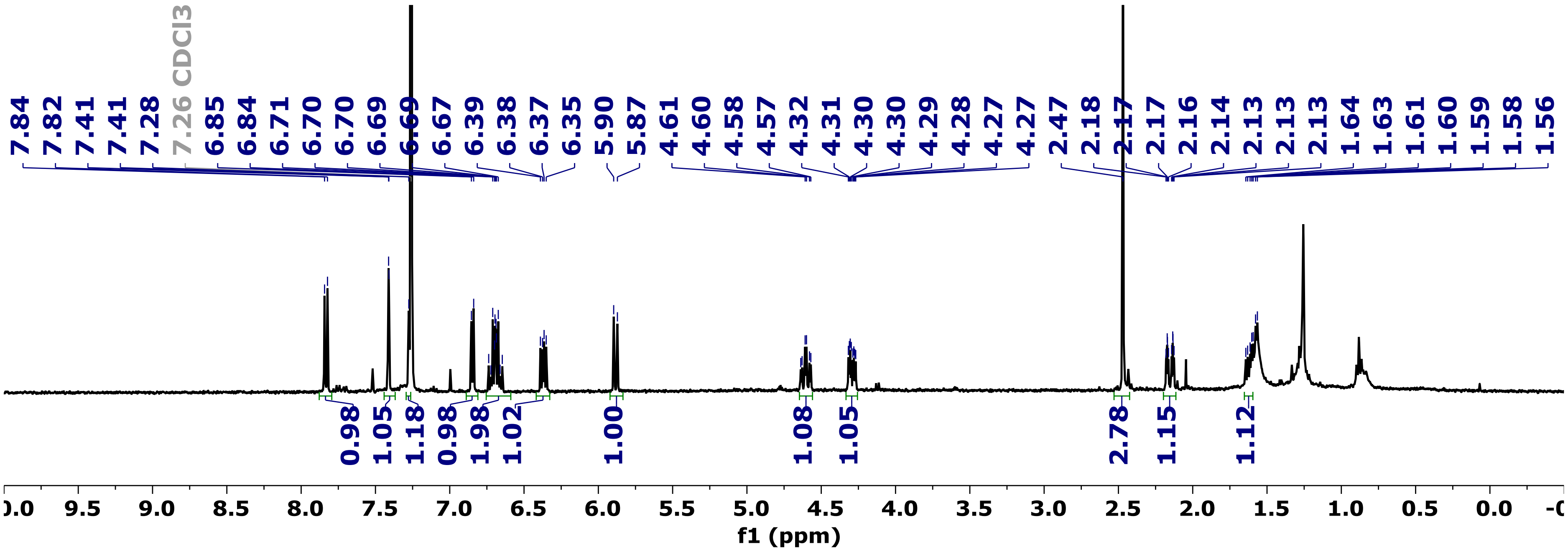

$^{13}C\{H\}$ NMR (101 MHz, $CDCl_3$)

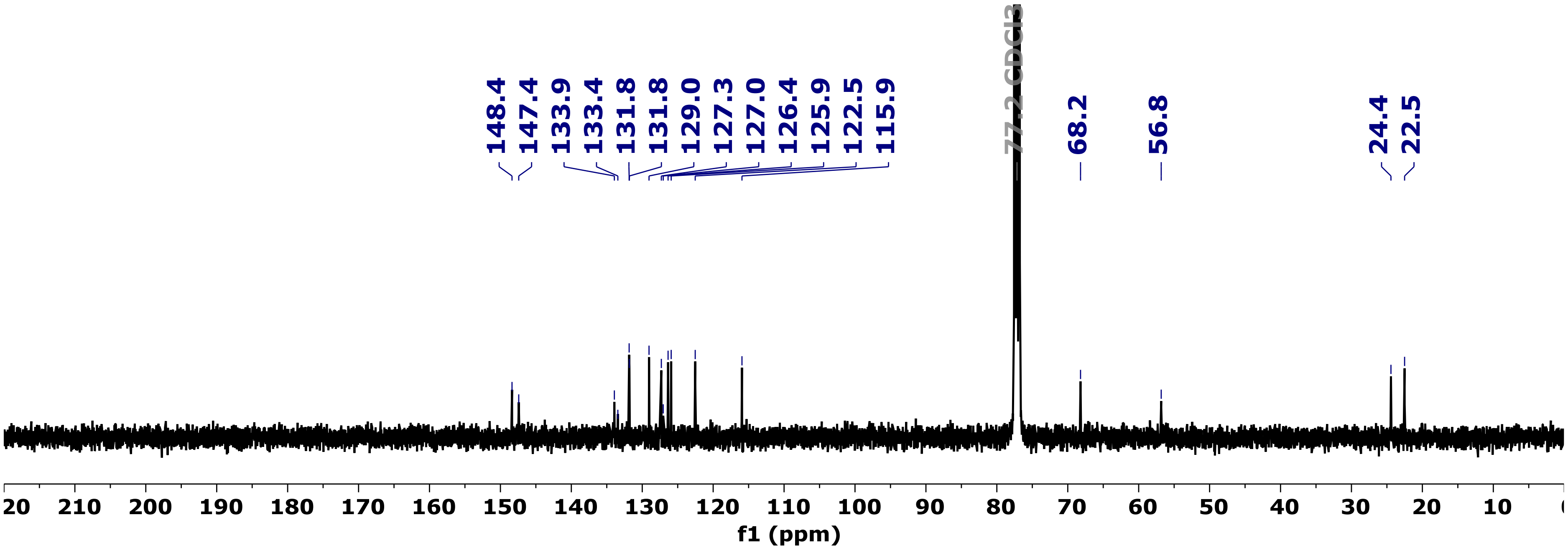

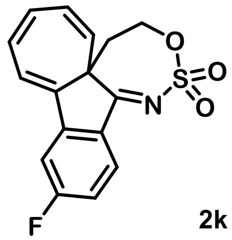

2k

^{1}H NMR (400 MHz, $CDCl_3$)

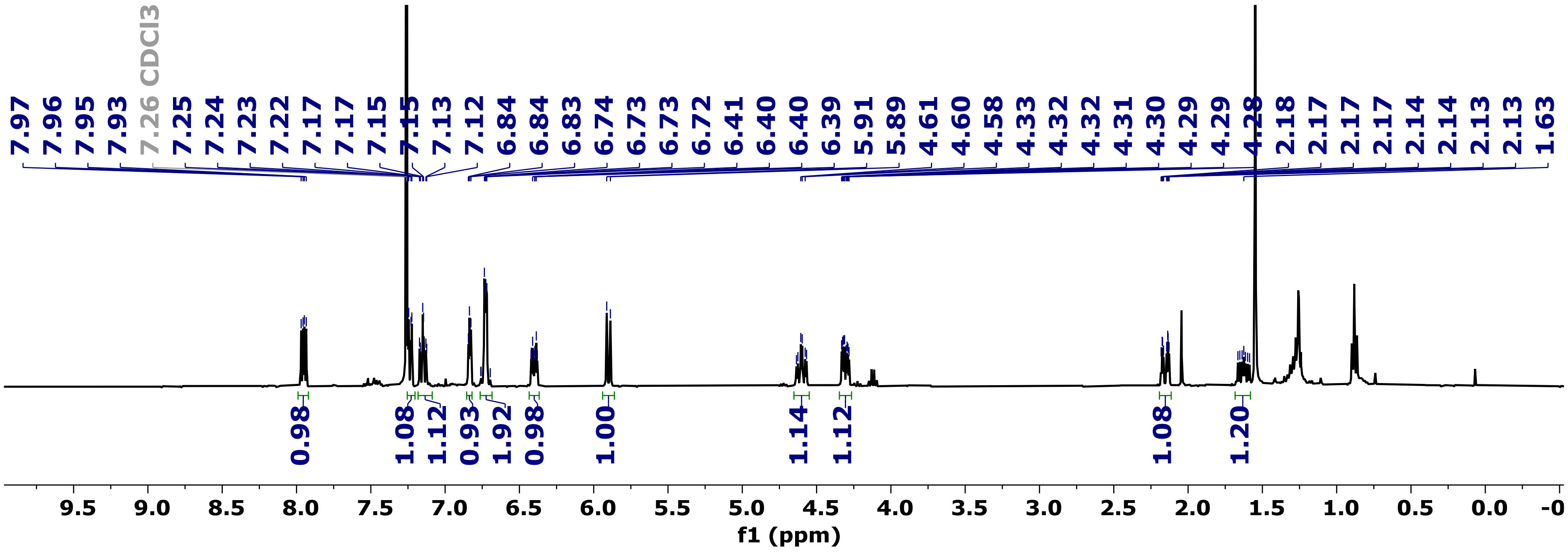

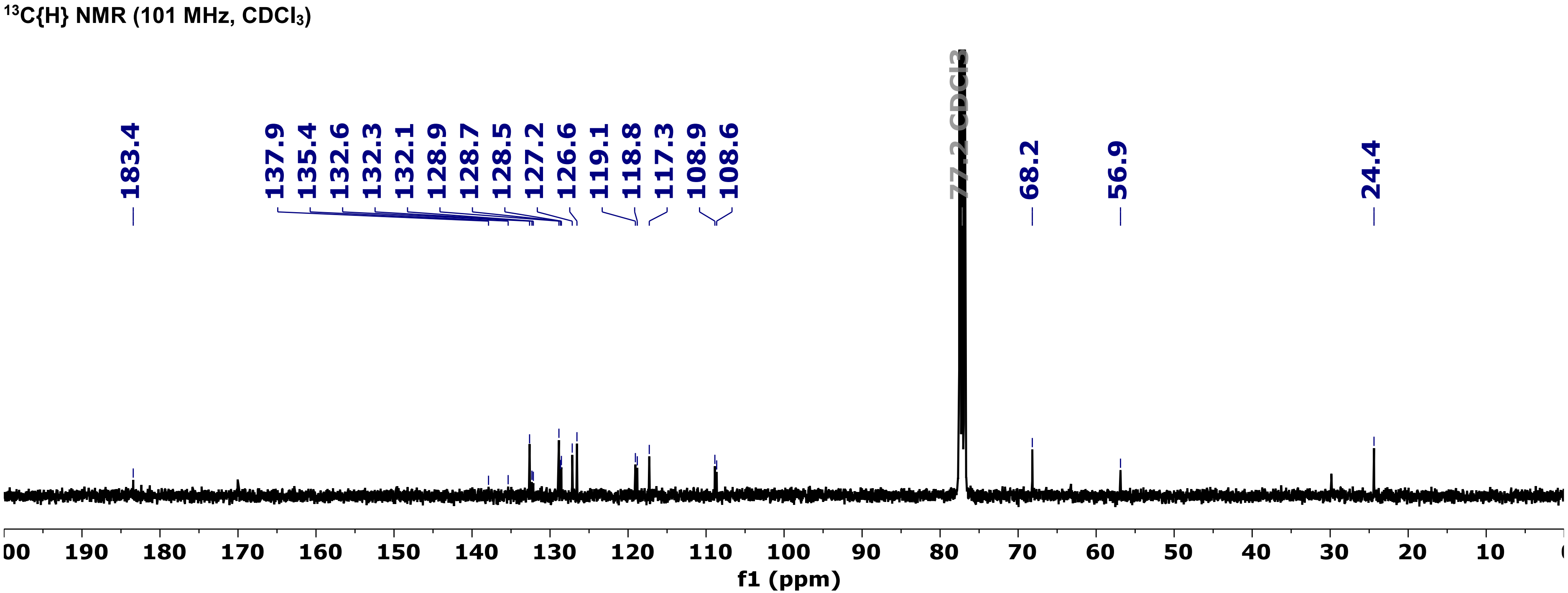

$^{13}C\{H\}$ NMR (101 MHz, $CDCl_3$)
183.4
137.9
135.4
132.6
132.3
132.1
128.9
128.7
128.5
127.2
126.6
119.1
118.8
117.3
108.9
108.6
77.2 CDCl3
68.2
56.9
24.4
f1 (ppm)

^{19}F NMR (377 MHz, $CDCl_3$)

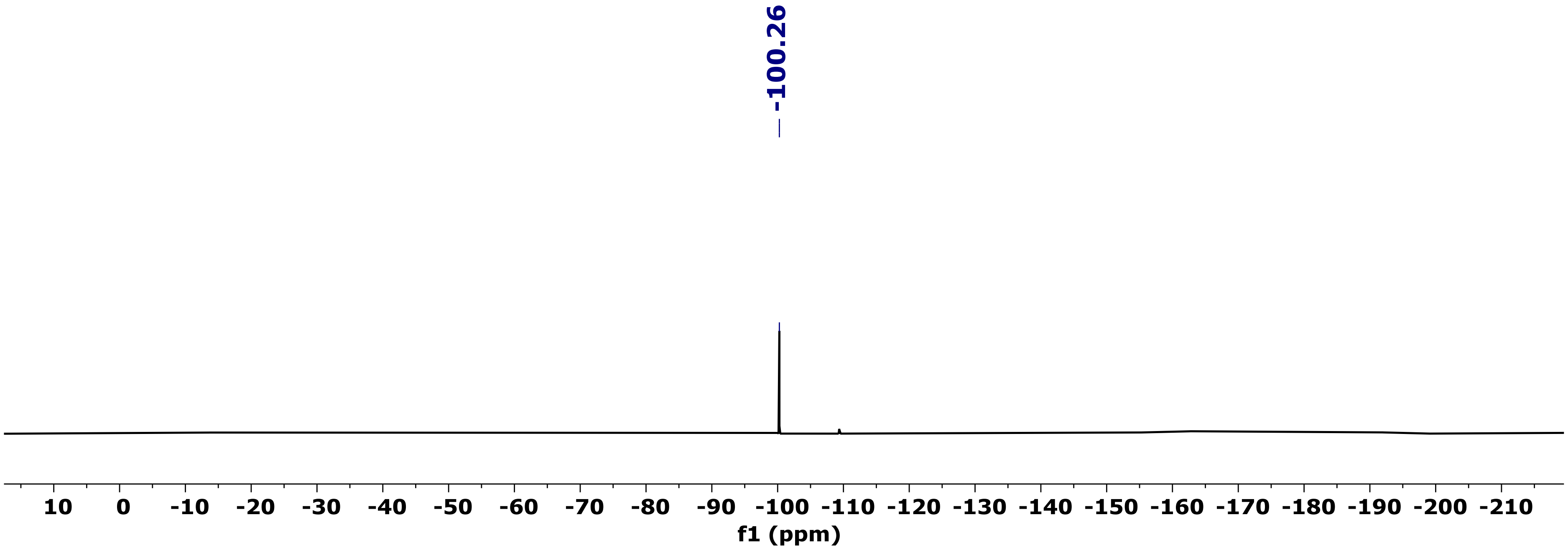

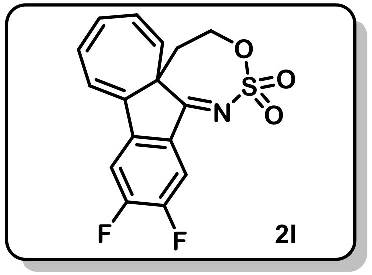

^{1}H NMR (400 MHz, $CDCl_3$)

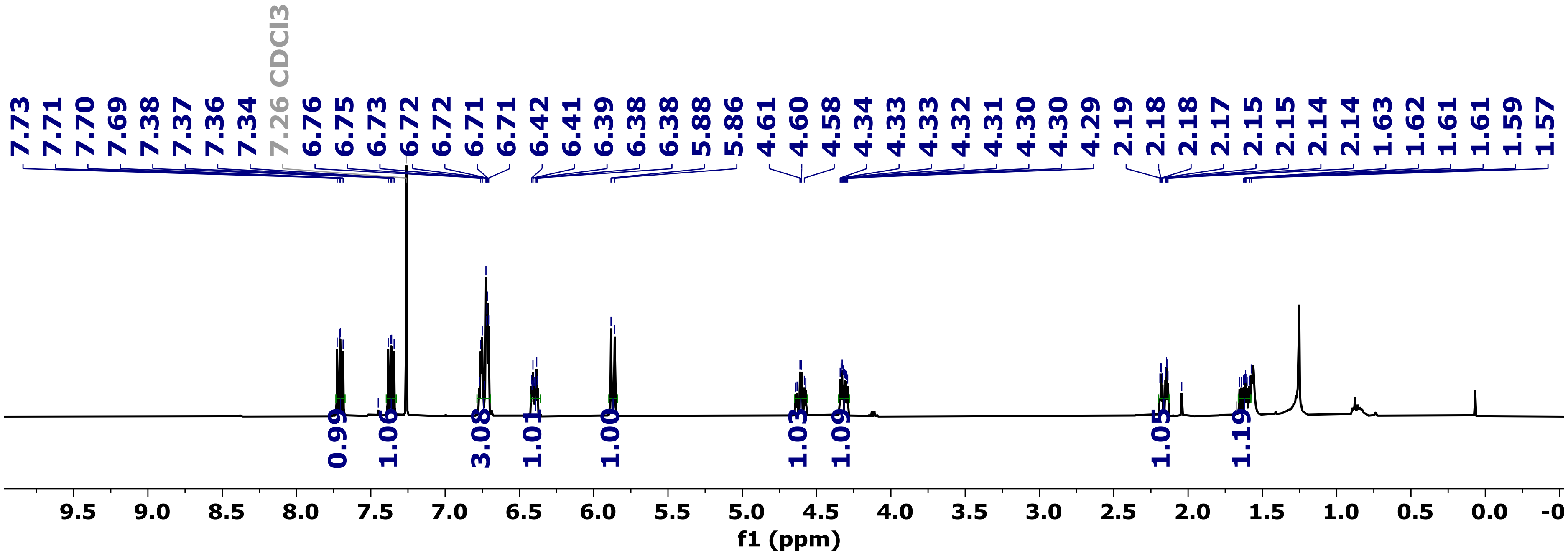

$^{13}C\{H\}$ NMR (101 MHz, $CDCl_3$)

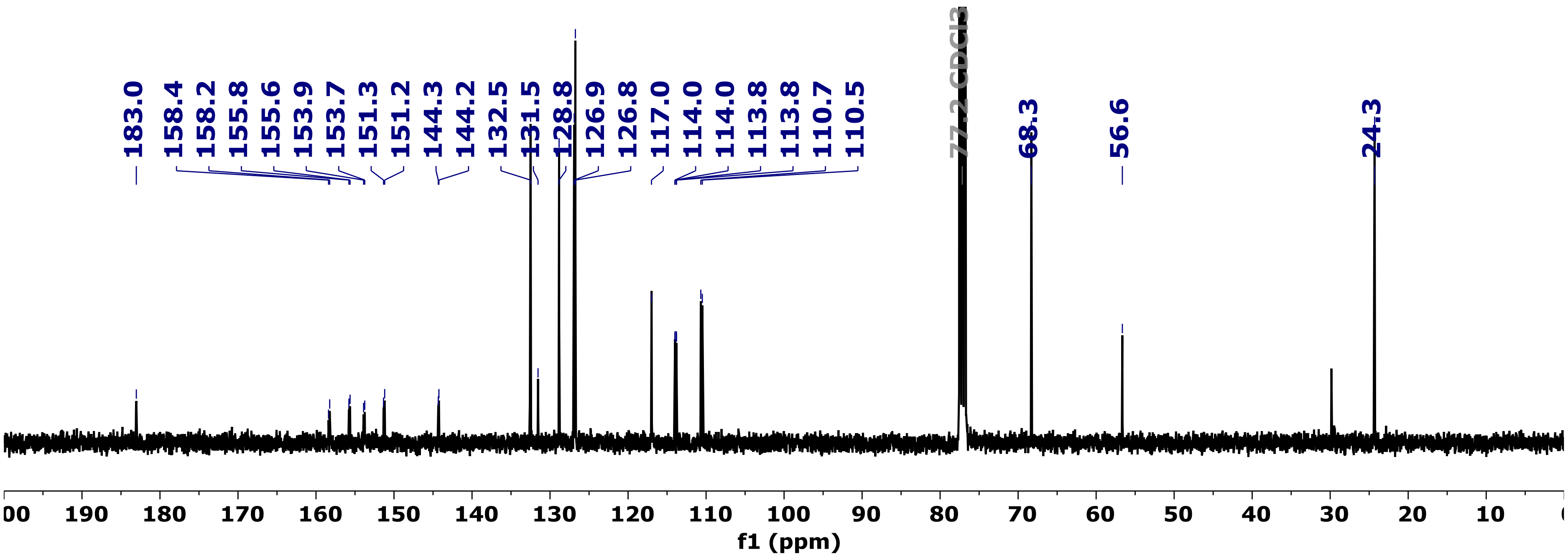

^{19}F NMR (377 MHz, $CDCl_3$)

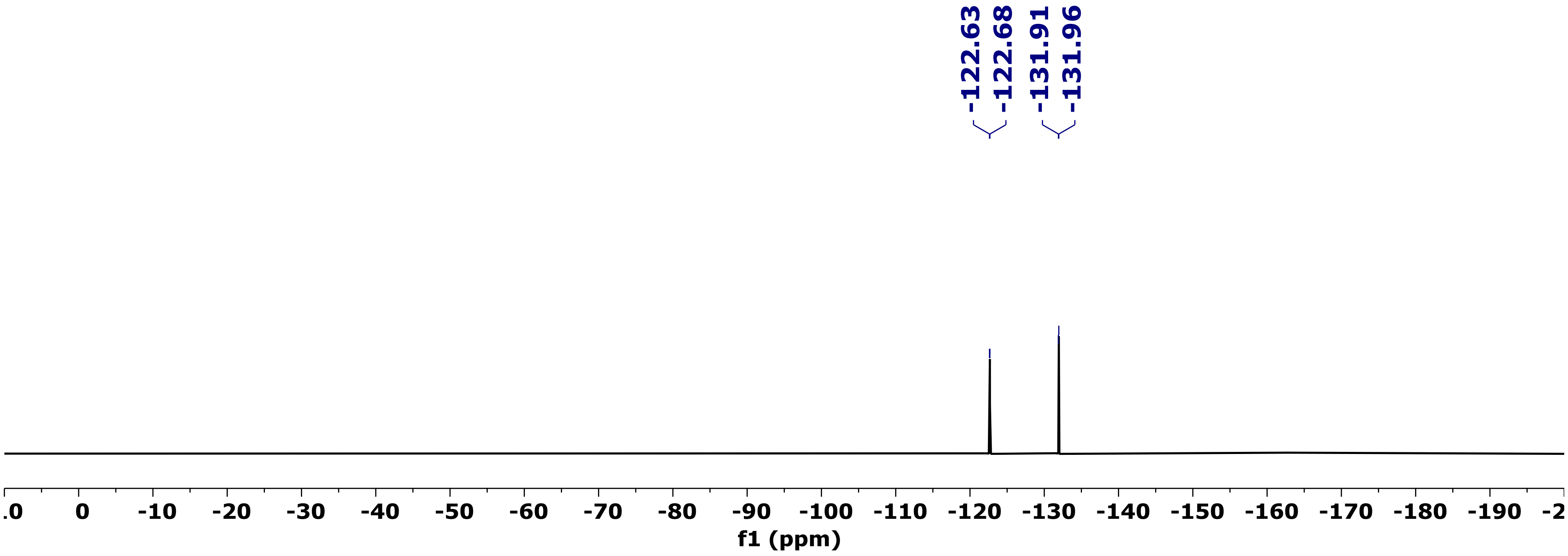

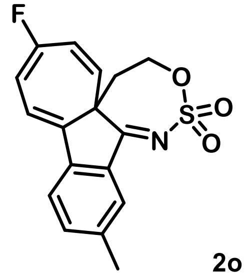

2o

^{1}H NMR (400 MHz, $CDCl_3$)

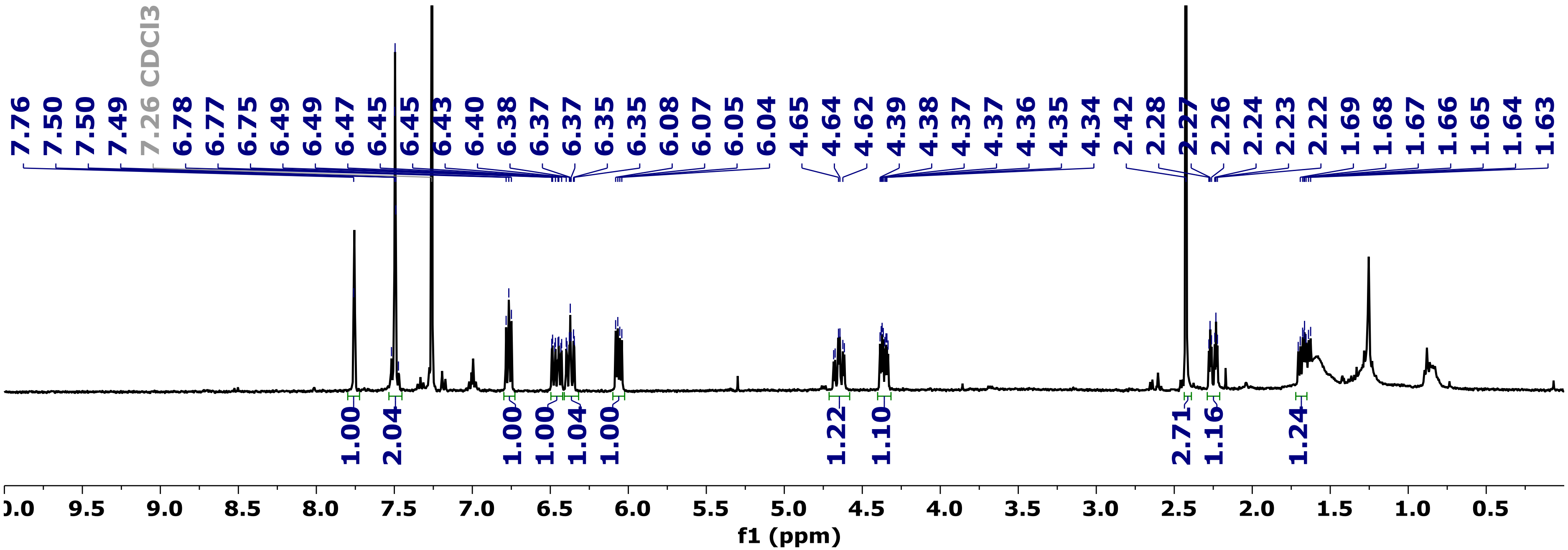

$^{13}C\{H\}$ NMR (101 MHz, $CDCl_3$)

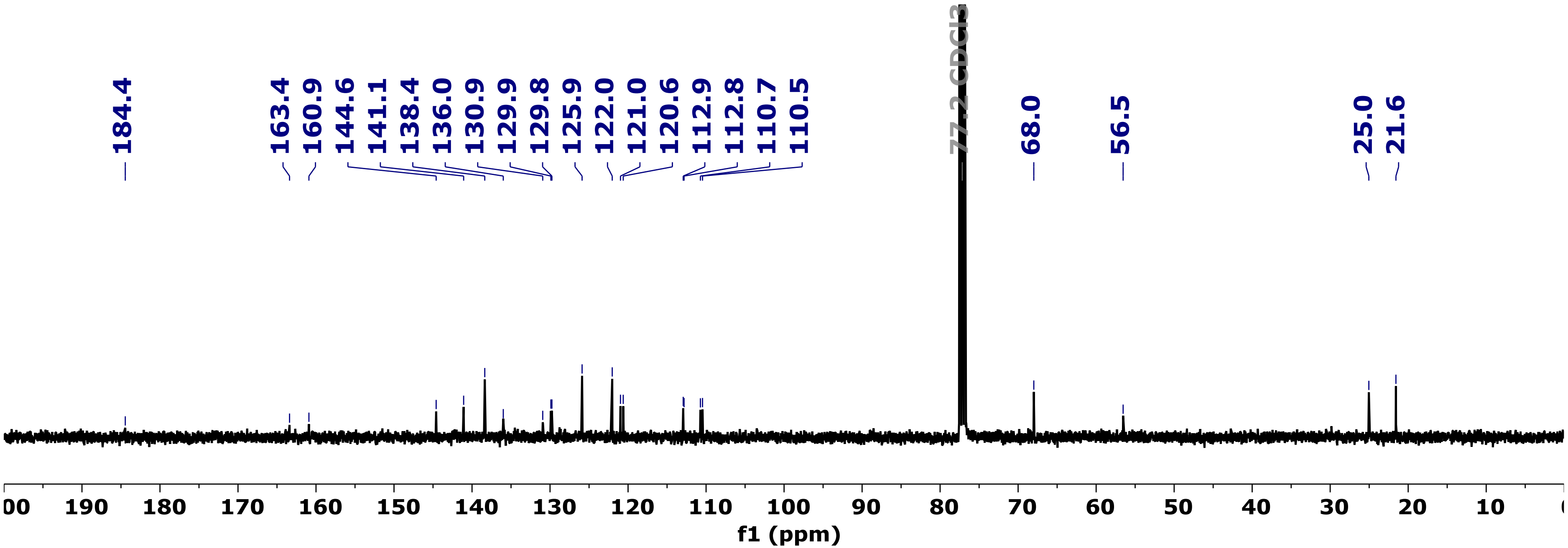

^{19}F NMR (377 MHz, $CDCl_3$)

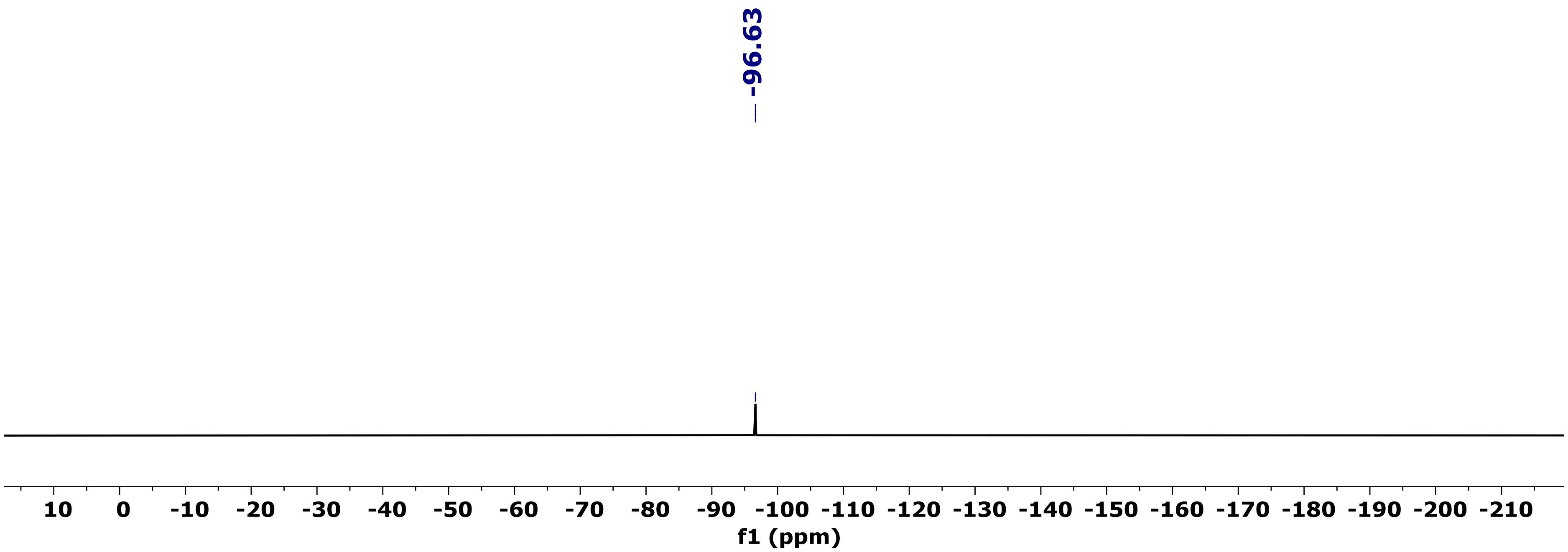

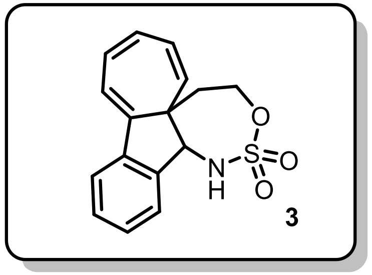

3

^{1}H NMR (400 MHz, $CDCl_3$)

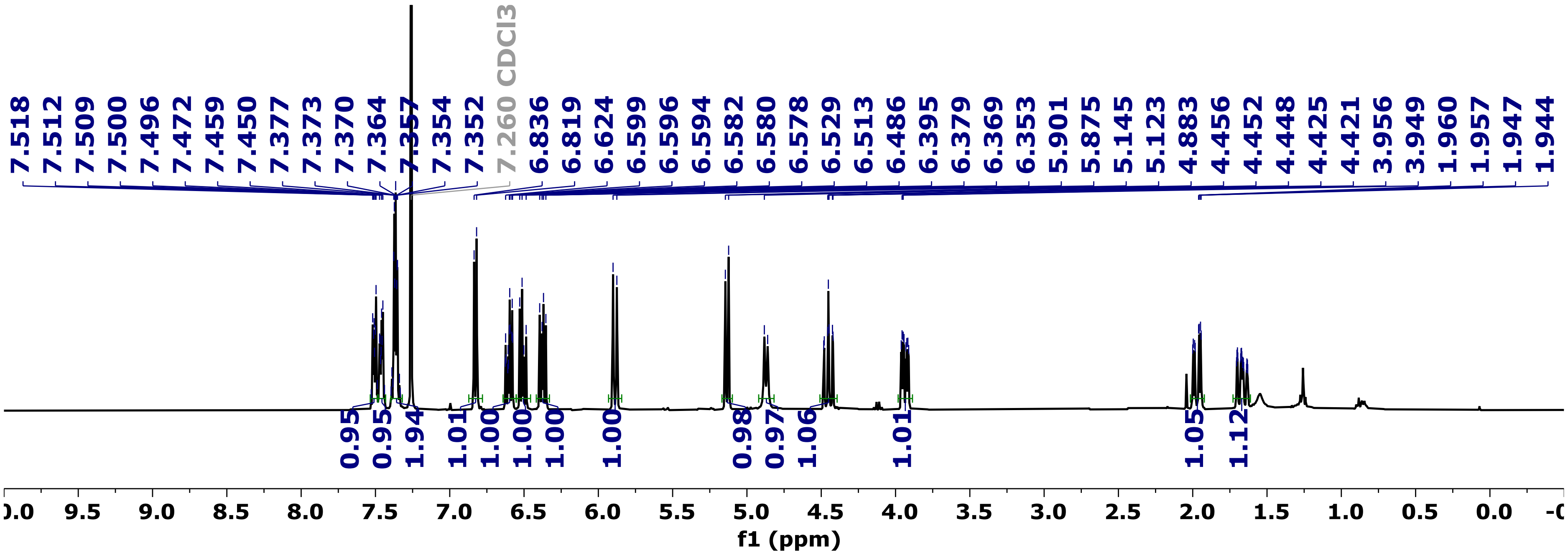

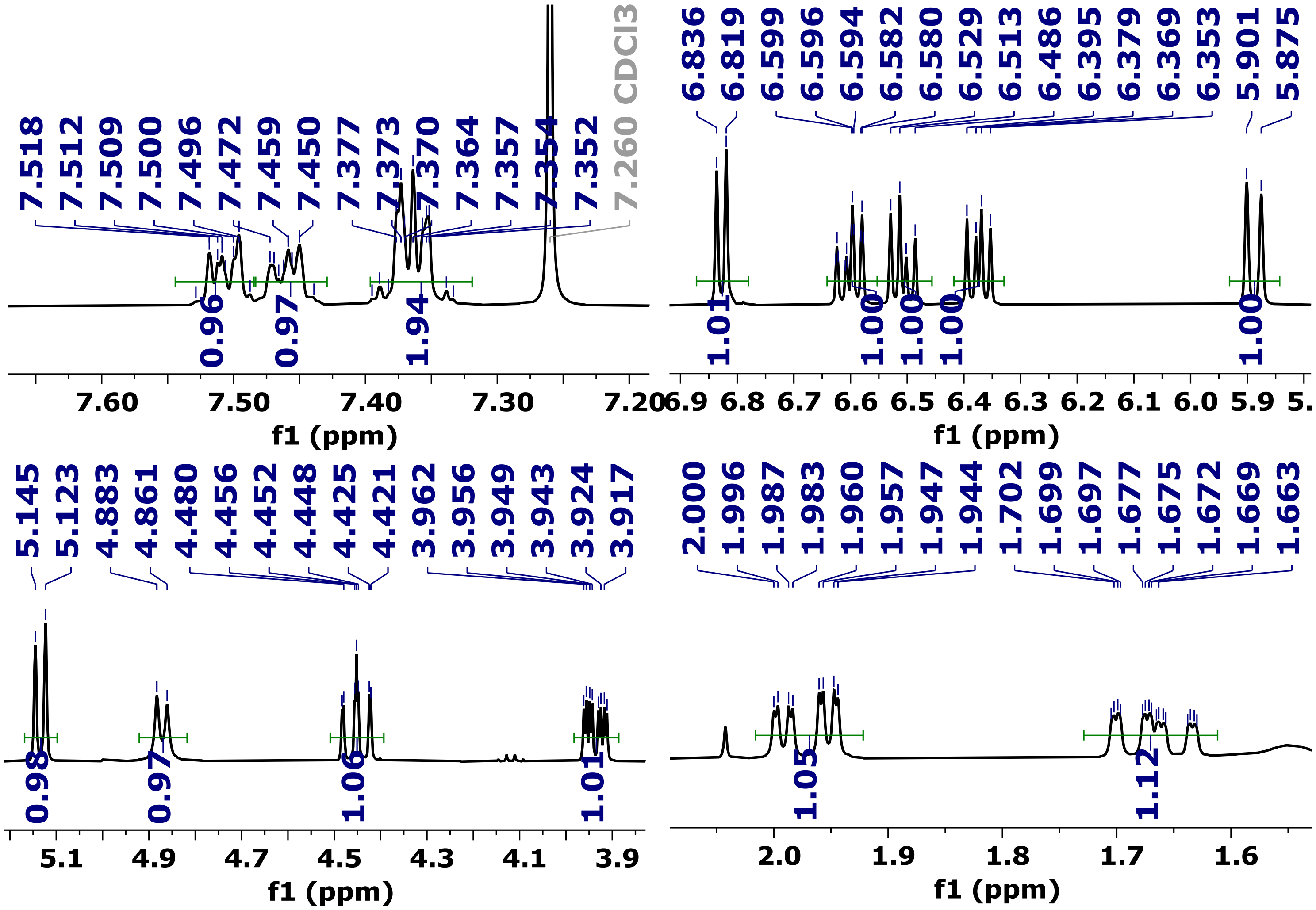

7.518
7.512
7.509
7.500
7.496
7.472
7.459
7.450
7.377
7.373
7.370
7.364
7.357
7.354
7.352
7.260 CDCl3
0.96
0.97
1.94
7.60
7.50
7.40
7.30
7.20
f1 (ppm)
6.836
6.819
6.599
6.596
6.594
6.582
6.580
6.529
6.513
6.486
6.395
6.379
6.369
6.353
5.901
5.875
1.01
1.00
1.00
1.00
1.00
6.9 6.8 6.7 6.6 6.5 6.4 6.3 6.2 6.1 6.0 5.9 5.
f1 (ppm)
5.145
5.123
4.883
4.861
4.480
4.456
4.452
4.448
4.425
4.421
3.962
3.956
3.949
3.943
3.924
3.917
0.98
0.97
1.06
1.01
5.1 4.9 4.7 4.5 4.3 4.1 3.9
f1 (ppm)
2.000
1.996
1.987
1.983
1.960
1.957
1.947
1.944
1.702
1.699
1.697
1.677
1.675
1.672
1.669
1.663
1.05
1.12
2.0 1.9 1.8 1.7 1.6
f1 (ppm)

$^{13}C\{H\}$ NMR (101 MHz, $CDCl_3$)

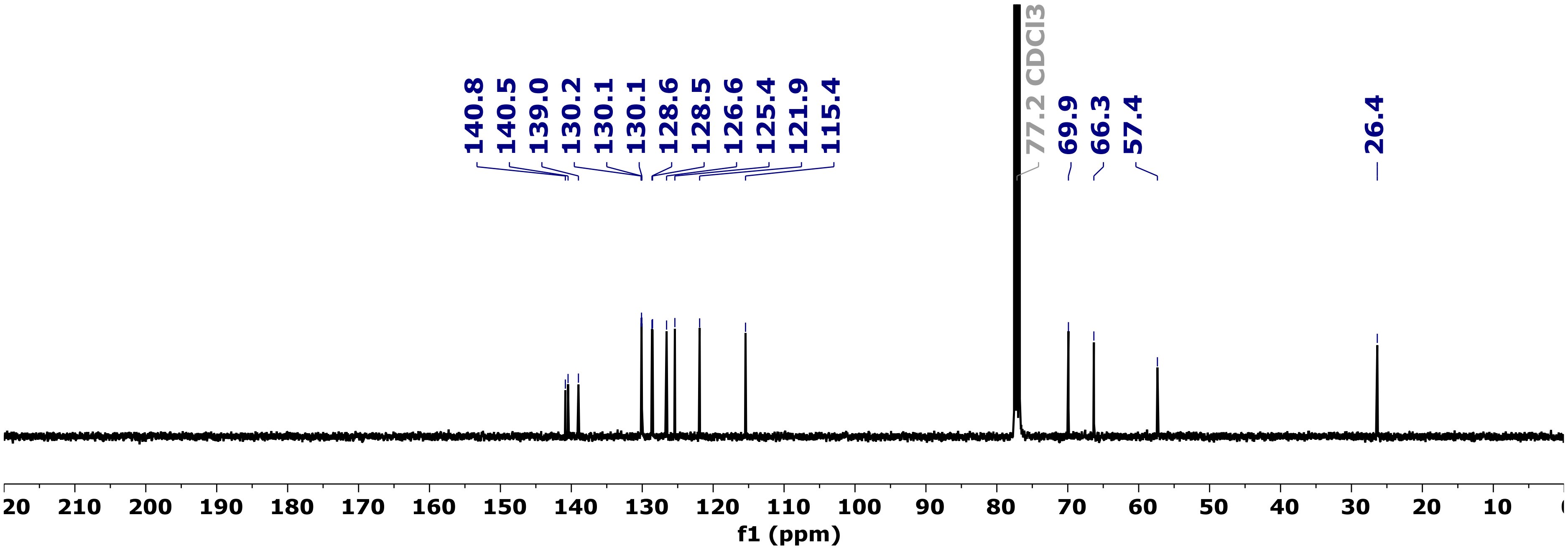

NOESY (101 MHz, CDCl$_3$)

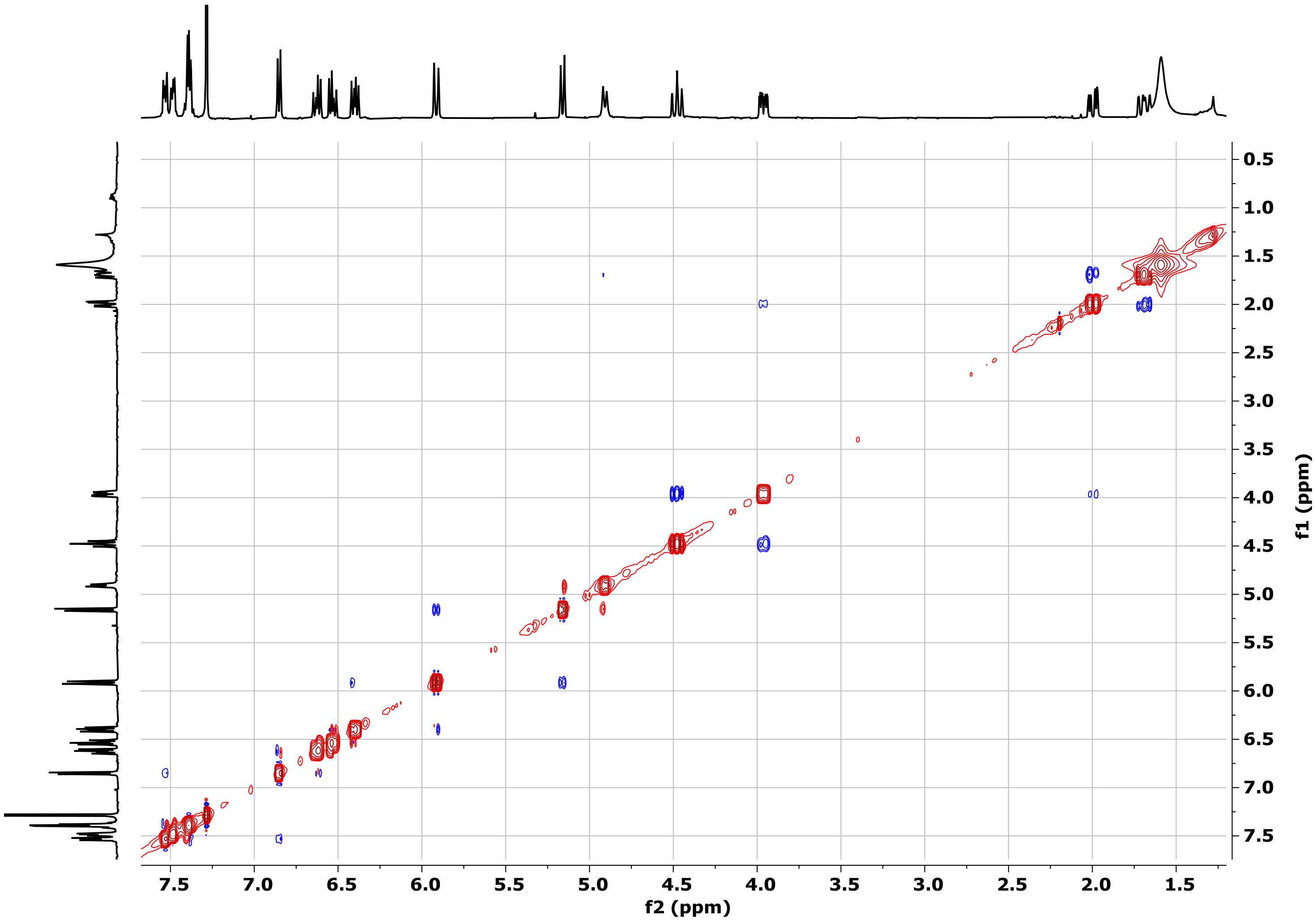

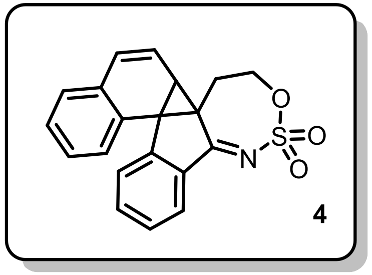

^{1}H NMR (400 MHz, $CDCl_3$)

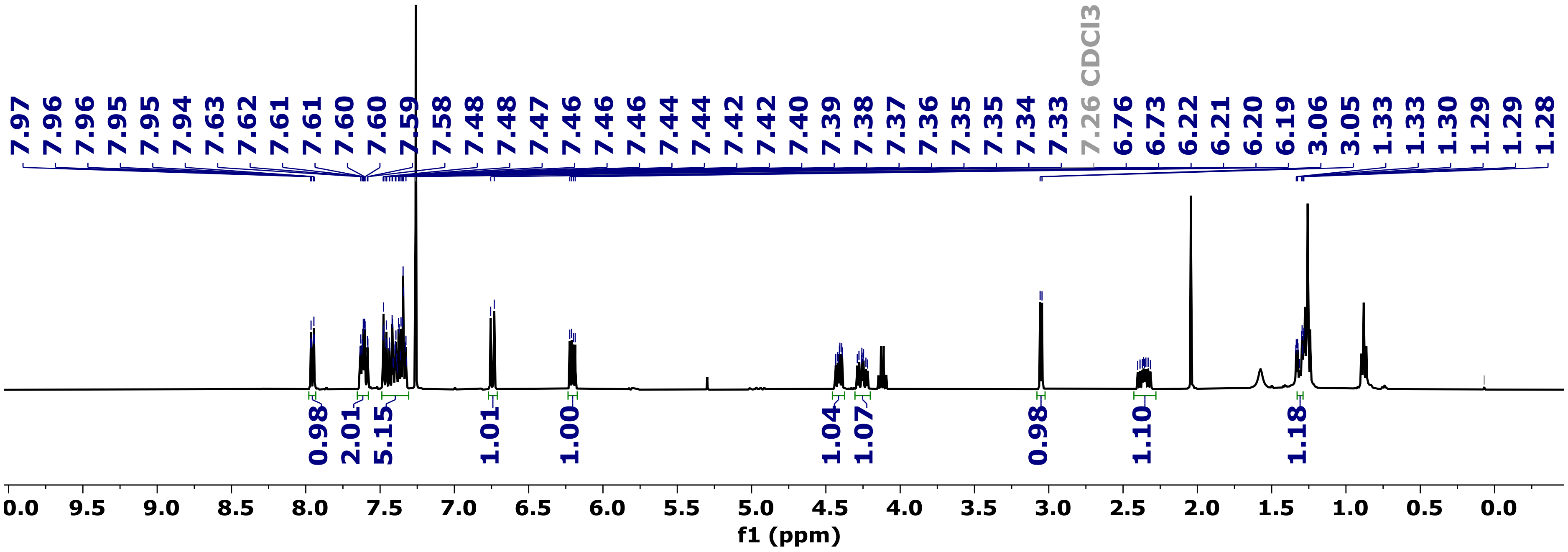

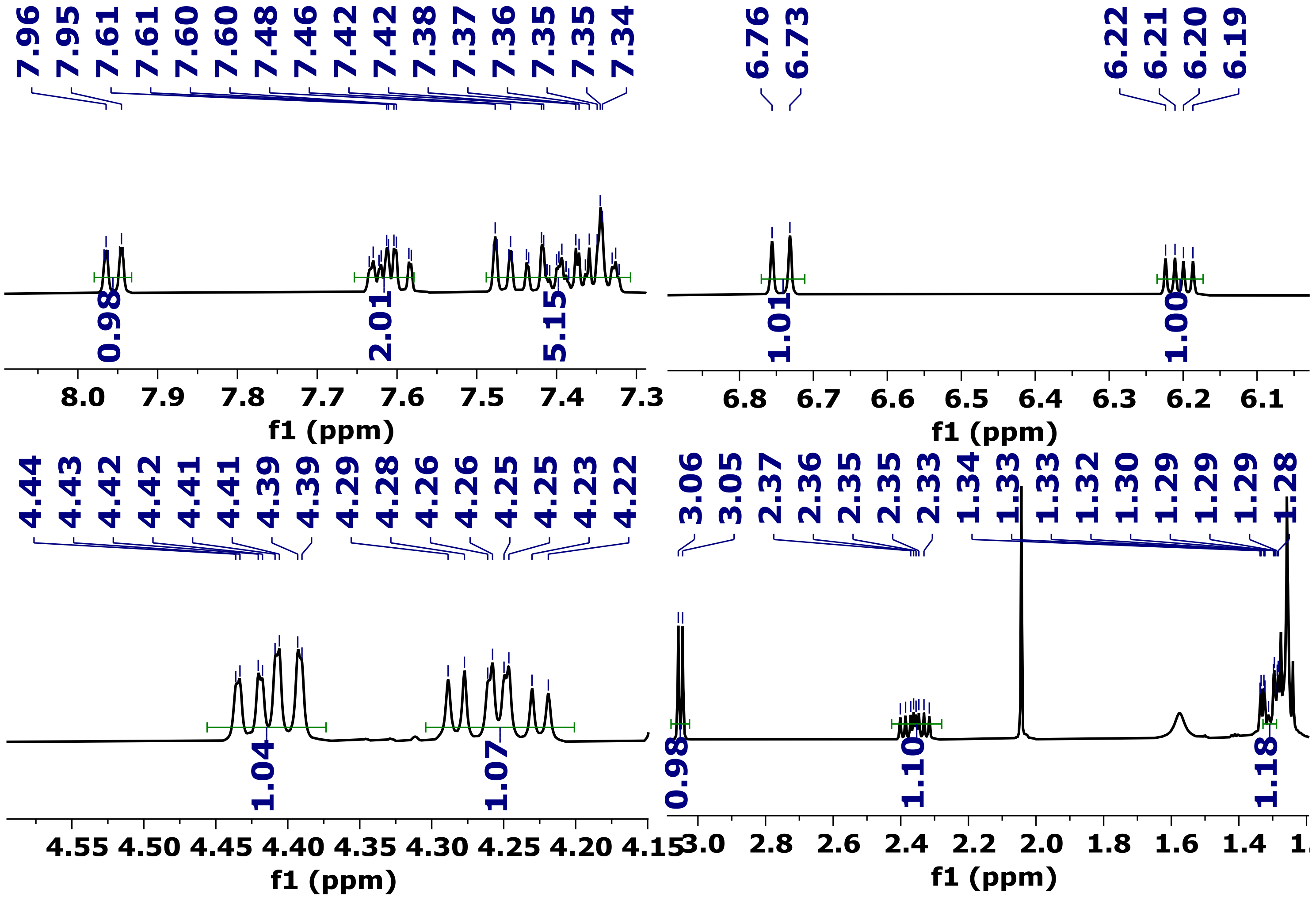
7.96
7.95
7.61
7.61
7.60
7.60
7.48
7.46
7.42
7.42
7.38
7.37
7.36
7.35
7.35
7.34
0.98
2.01
5.15
8.0 7.9 7.8 7.7 7.6 7.5 7.4 7.3
f1 (ppm)
6.76
6.73
6.22
6.21
6.20
6.19
1.01
1.00
6.8 6.7 6.6 6.5 6.4 6.3 6.2 6.1
f1 (ppm)
4.44
4.43
4.42
4.42
4.41
4.41
4.39
4.39
4.29
4.28
4.26
4.26
4.25
4.25
4.23
4.22
1.04
1.07
4.55 4.50 4.45 4.40 4.35 4.30 4.25 4.20 4.15
f1 (ppm)
3.06
3.05
2.37
2.36
2.35
2.35
2.33
1.34
1.33
1.33
1.32
1.30
1.29
1.29
1.29
1.28
0.98
1.10
1.18
3.0 2.8 2.6 2.4 2.2 2.0 1.8 1.6 1.4
f1 (ppm)

13C{H} NMR (101 MHz, CDCl3)

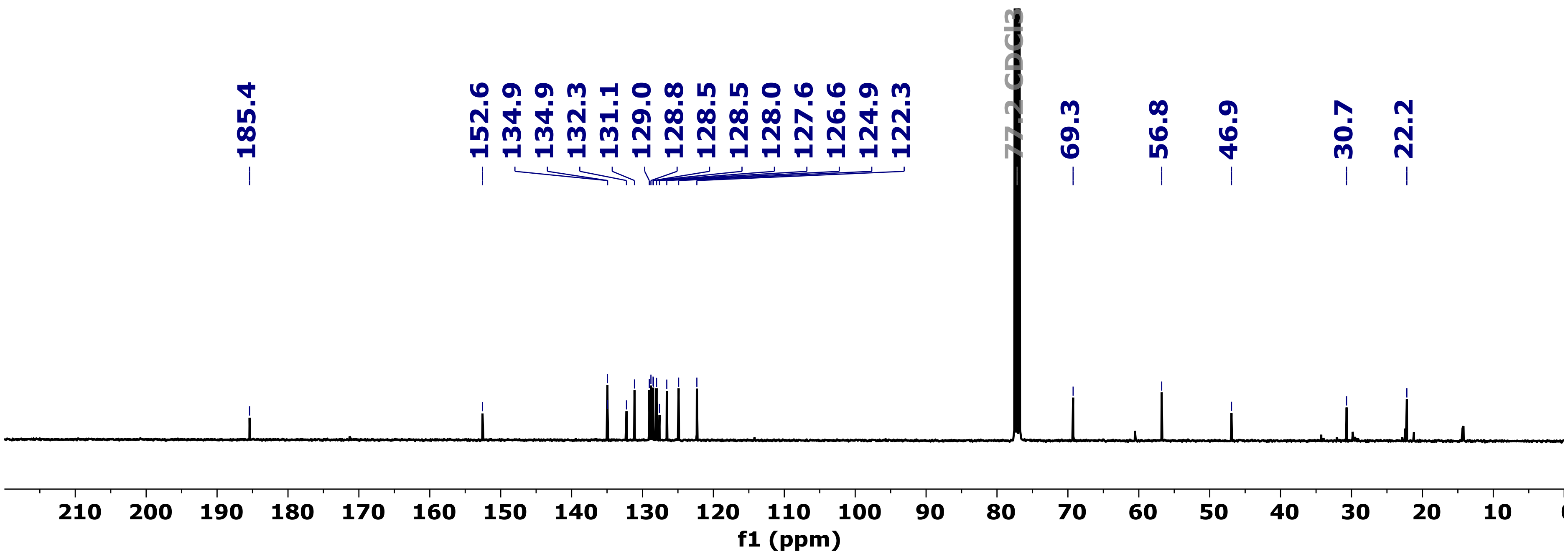

Supplement: Supplementary file 1 [file cs5c06179_si_001.pdf]
